# Supplementary material for: NHC-catalyzed atropoenantioselective synthesis of axially chiral biaryl amino alcohols via a cooperative strategy
Source: Nat Commun. 2019 Jul 11;10:3062. doi: 10.1038/s41467-019-10878-7 (PMC6624290; doi:10.1038/s41467-019-10878-7)
Supplement: Supplementary file 1 — Supplementary Information [file 41467_2019_10878_MOESM1_ESM.pdf]

NHC-catalyzed Atropoenantioselective Synthesis of Axially Chiral Biaryl Amino Alcohols via a Cooperative Strategy

Yang *et al.*

Supplementary Information

Supplementary Figures

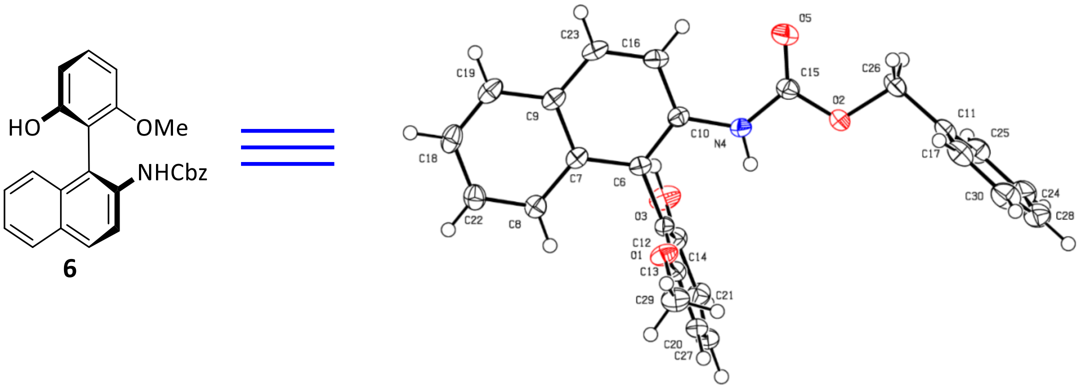

Supplementary Figure 1. X-ray crystal structure of 6

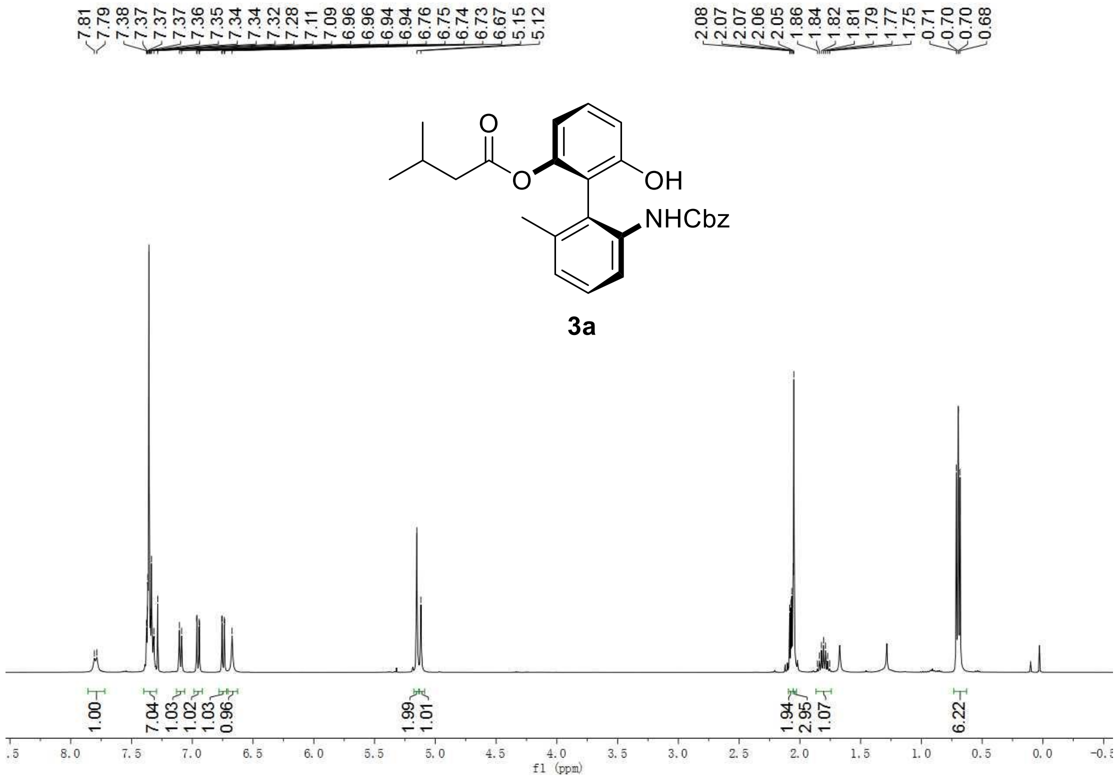

Supplementary Figure 2. ^{1}H NMR Spectrum of 3a.

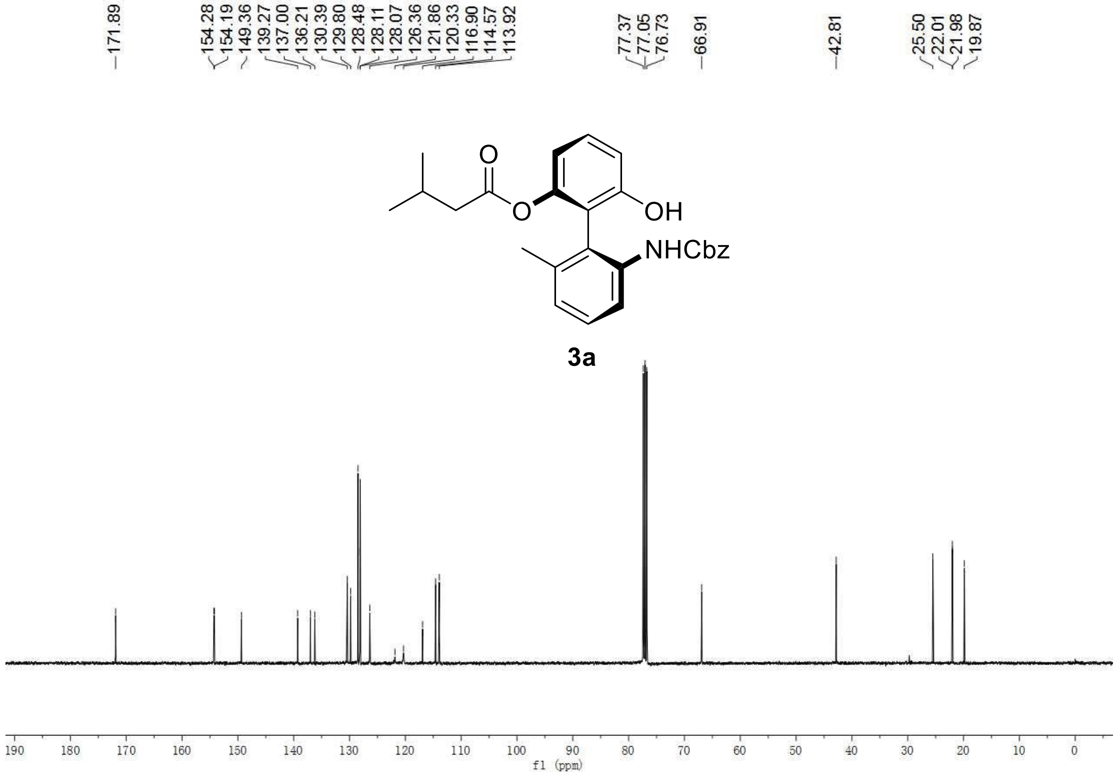

Supplementary Figure 3. ^{13}C NMR Spectrum of 3a.

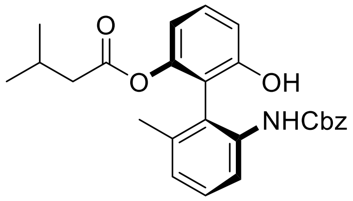

3a

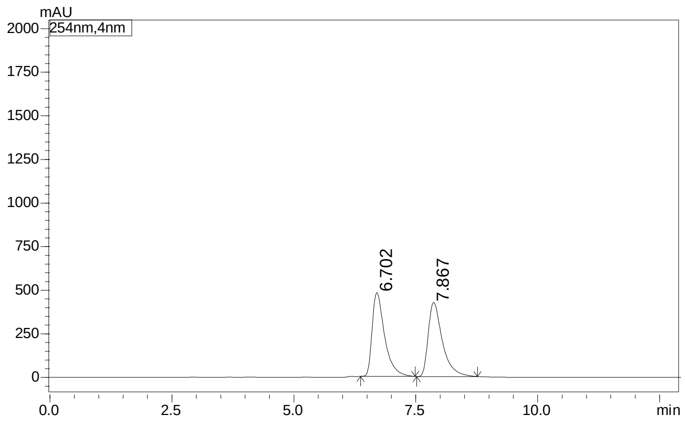

Peak	Ret. Time	Area	Height	Area%	Height%
1	6.702	8564833	480128	49.904	53.067
2	7.867	8597818	424628	50.096	46.933
Tolal		17162651	904755	100.000	100.000

Supplementary Figure 4. HPLC Spectrum of racemic 3a.

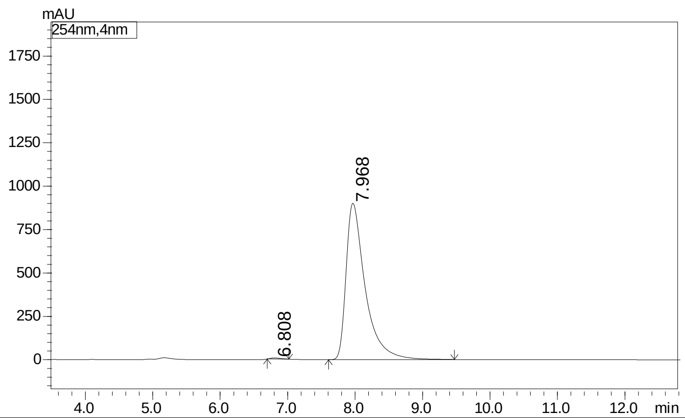

Peak	Ret. Time	Area	Height	Area%	Height%
1	6.808	69909	6699	0.393	0.738
2	7.968	17711048	901286	99.607	99.262
Tolal		17780956	907985	100.000	100.000

Supplementary Figure 5. HPLC Spectrum of 3a.

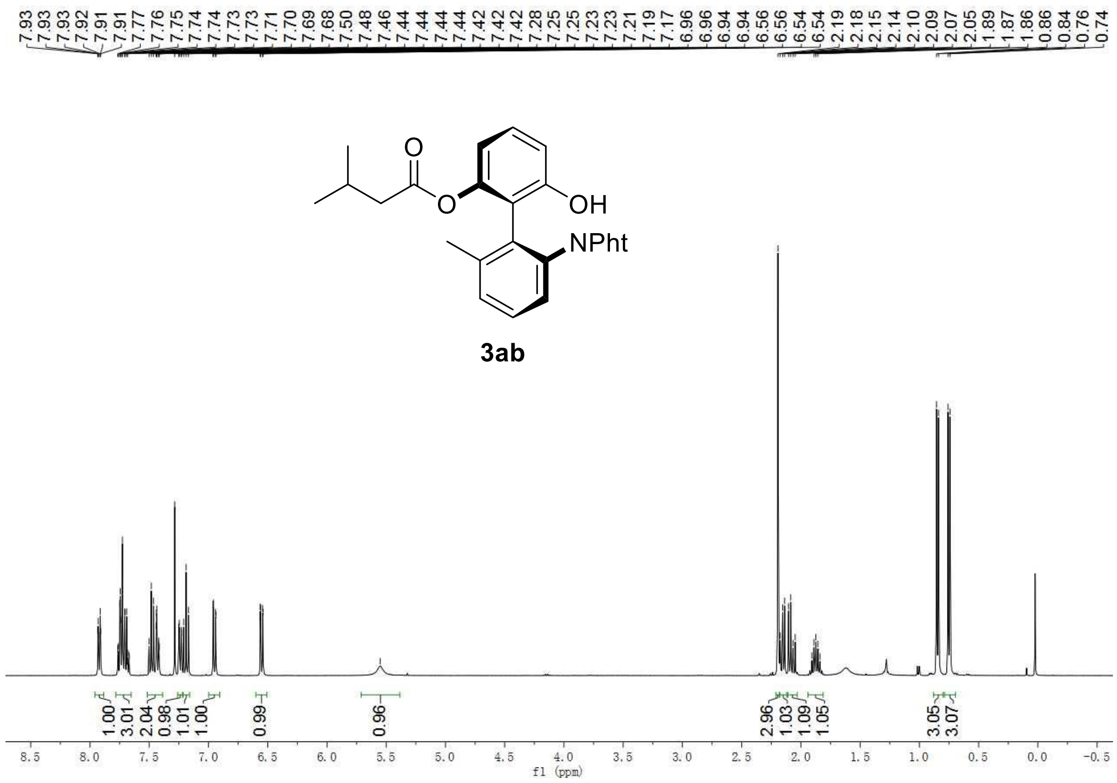

Supplementary Figure 6. ^{1}H NMR Spectrum of 3ab.

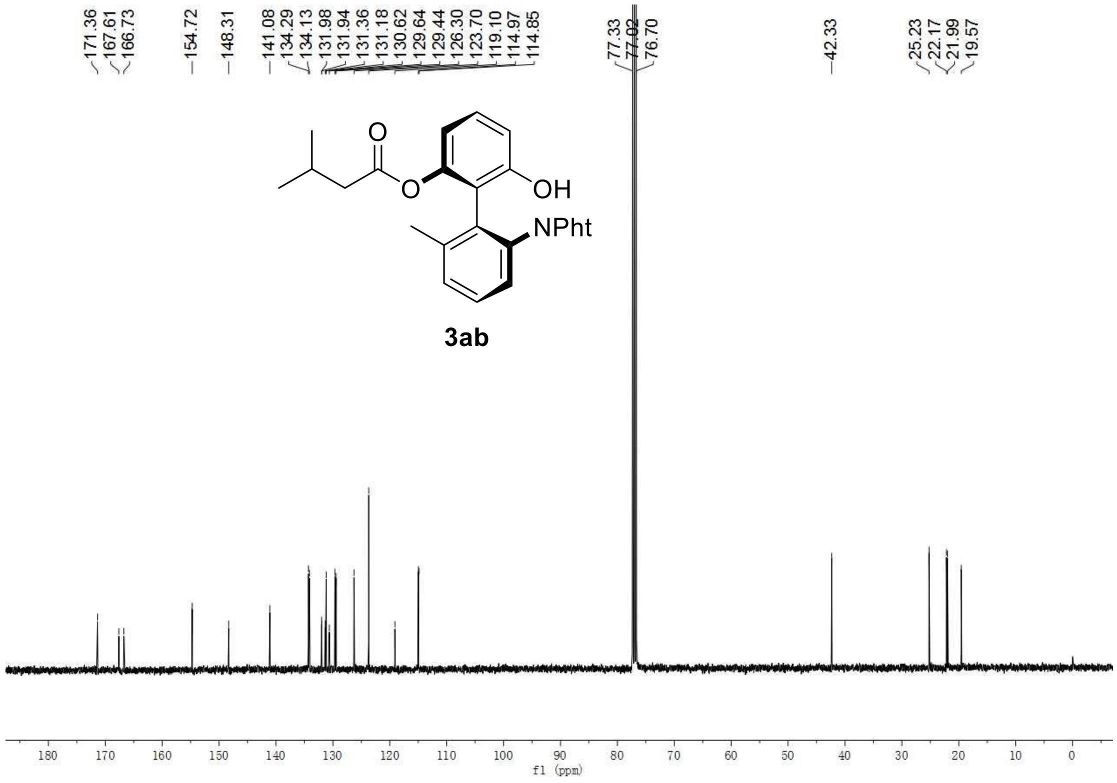

Supplementary Figure 7. ^{13}C NMR Spectrum of 3ab.

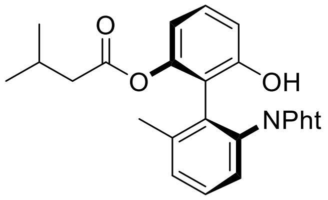

3ab

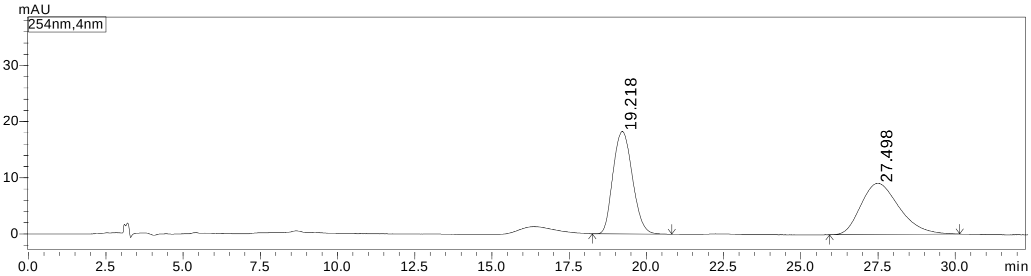

Peak	Ret. time	Area	Height	Area%	Height%
1	19.218	799241	18251	50.342	66.681
2	27.498	788381	9120	49.658	33.319
Total		1587622	27371	100.000	100.000

Supplementary Figure 8. HPLC Spectrum of racemic 3ab.

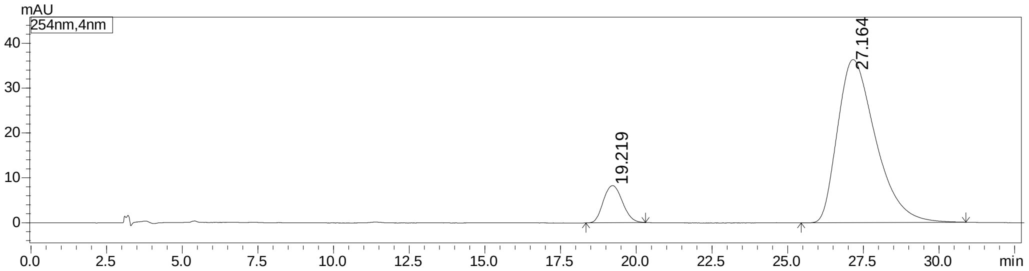

Peak	Ret. time	Area	Height	Area%	Height%
1	19.219	362926	8279	10.253	18.553
2	27.164	3176740	36345	89.747	81.447
Total		3539666	44624	100.000	100.000

Supplementary Figure 9. HPLC Spectrum of 3ab.

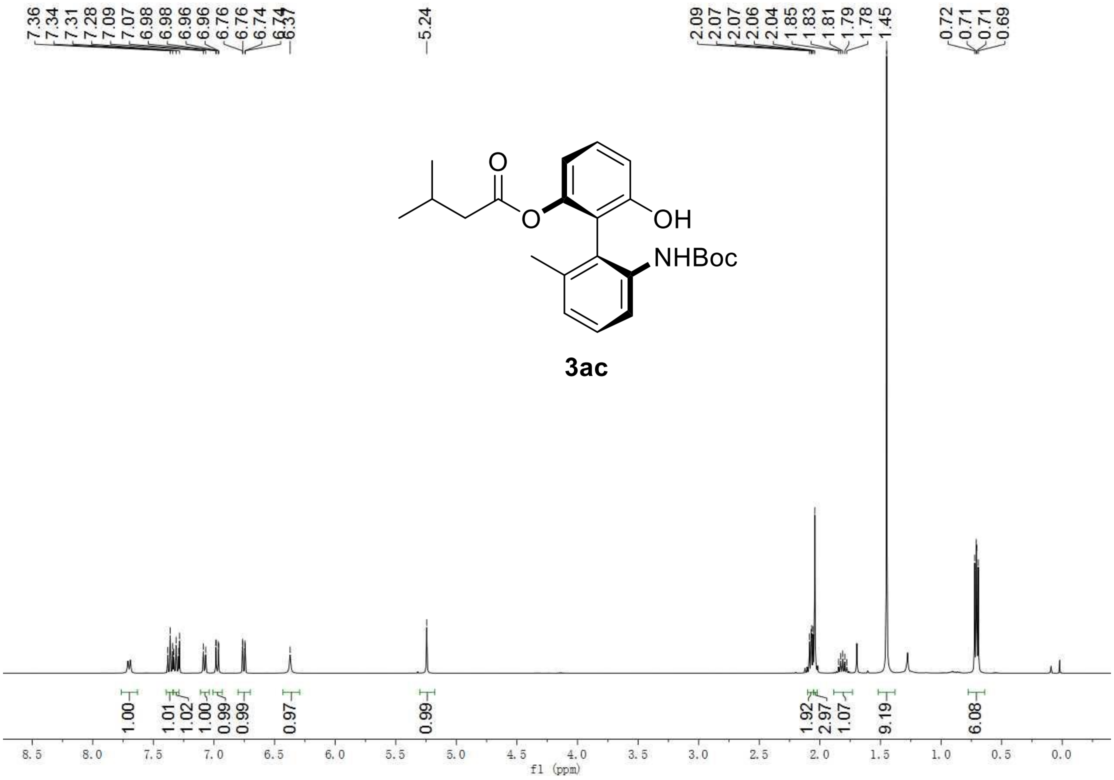

Supplementary Figure 10. ^{1}H NMR Spectrum of 3ac.

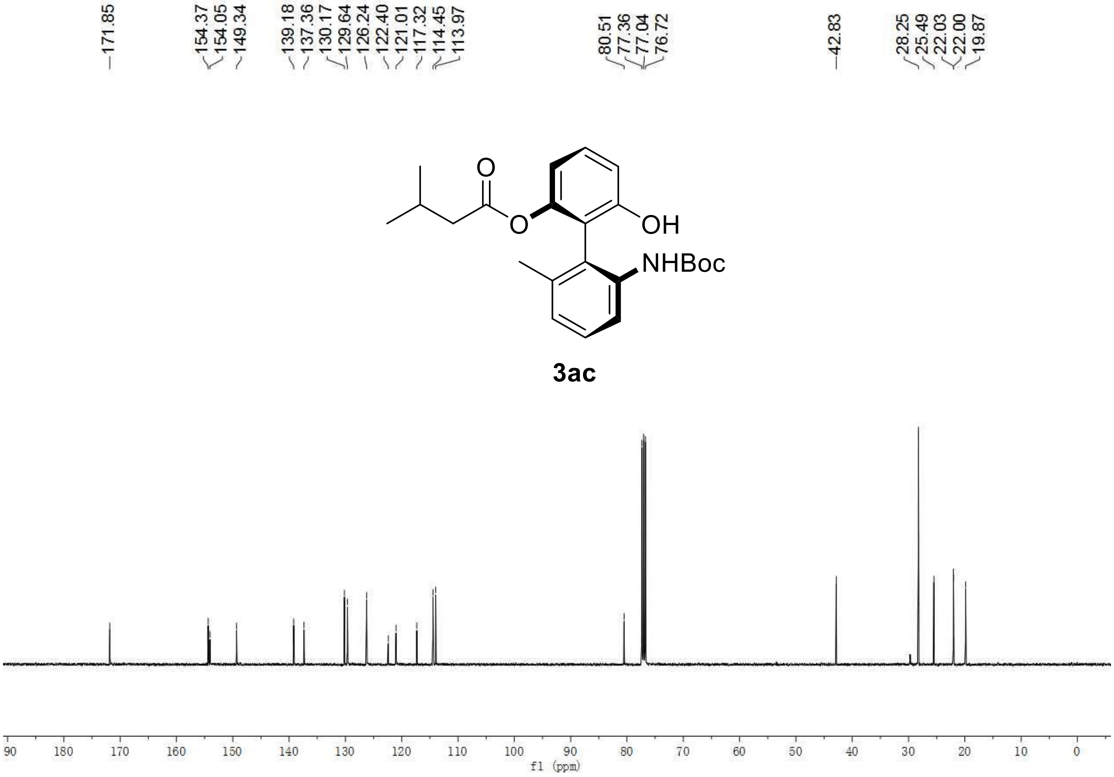

Supplementary Figure 11. ^{13}C NMR Spectrum of 3ac.

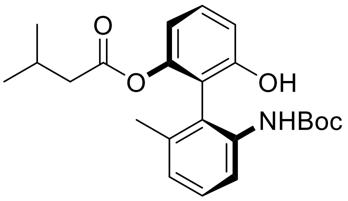

3ac

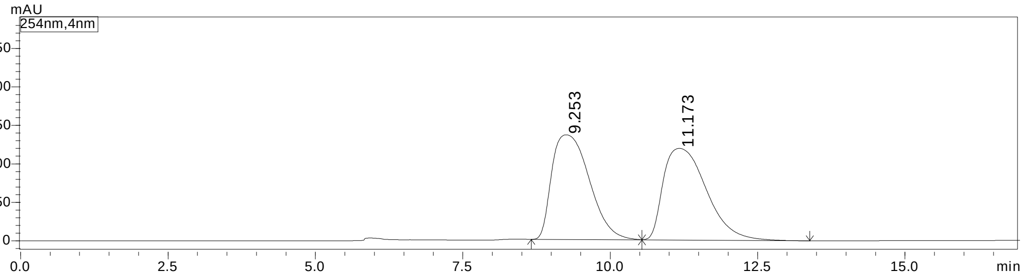

峰号	保留时间	面积	高度	面积%	高度%
1	9.253	6060127	136051	49.800	53.304
2	11.173	6108882	119187	50.200	46.696
总计		12169009	255238	100.000	100.000

Supplementary Figure 12. HPLC Spectrum of racemic 3ac.

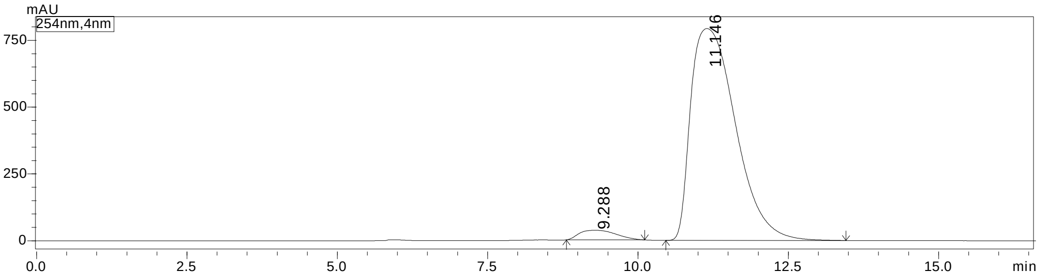

峰号	保留时间	面积	高度	面积%	高度%
1	9.288	1517144	35832	3.553	4.325
2	11.146	41182294	792628	96.447	95.675
总计		42699438	828460	100.000	100.000

Supplementary Figure 13. HPLC Spectrum of 3ac.

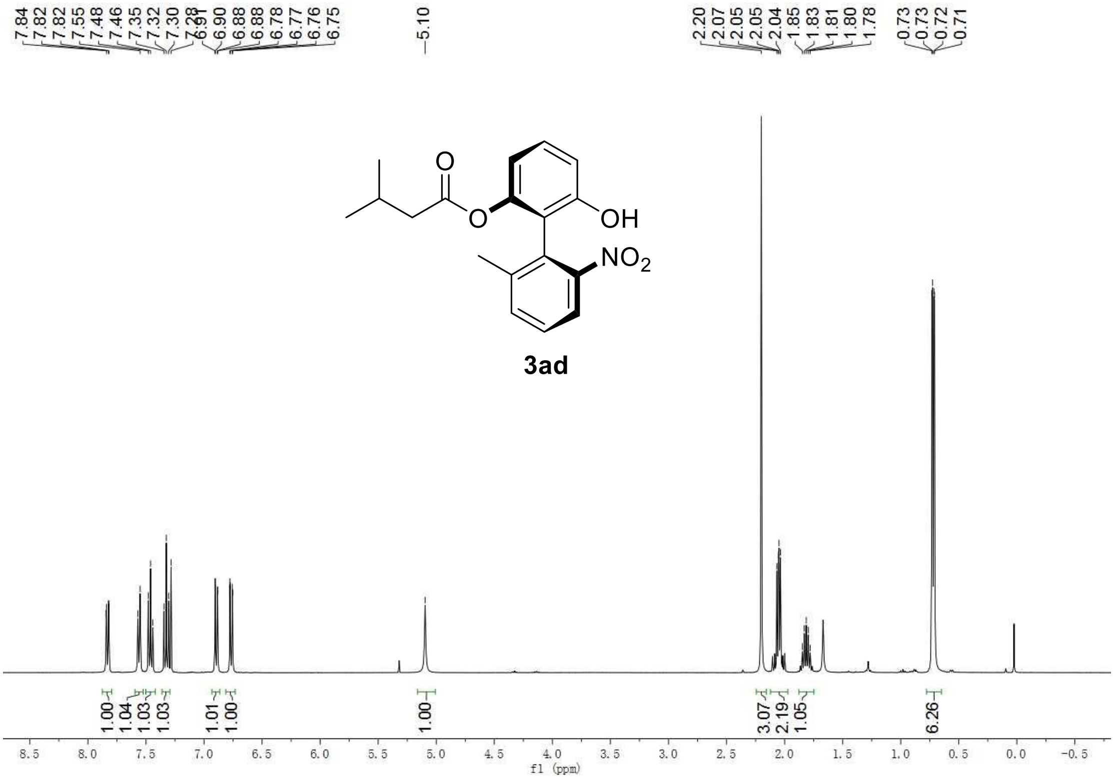

Supplementary Figure 14. ^{1}H NMR Spectrum of 3ad.

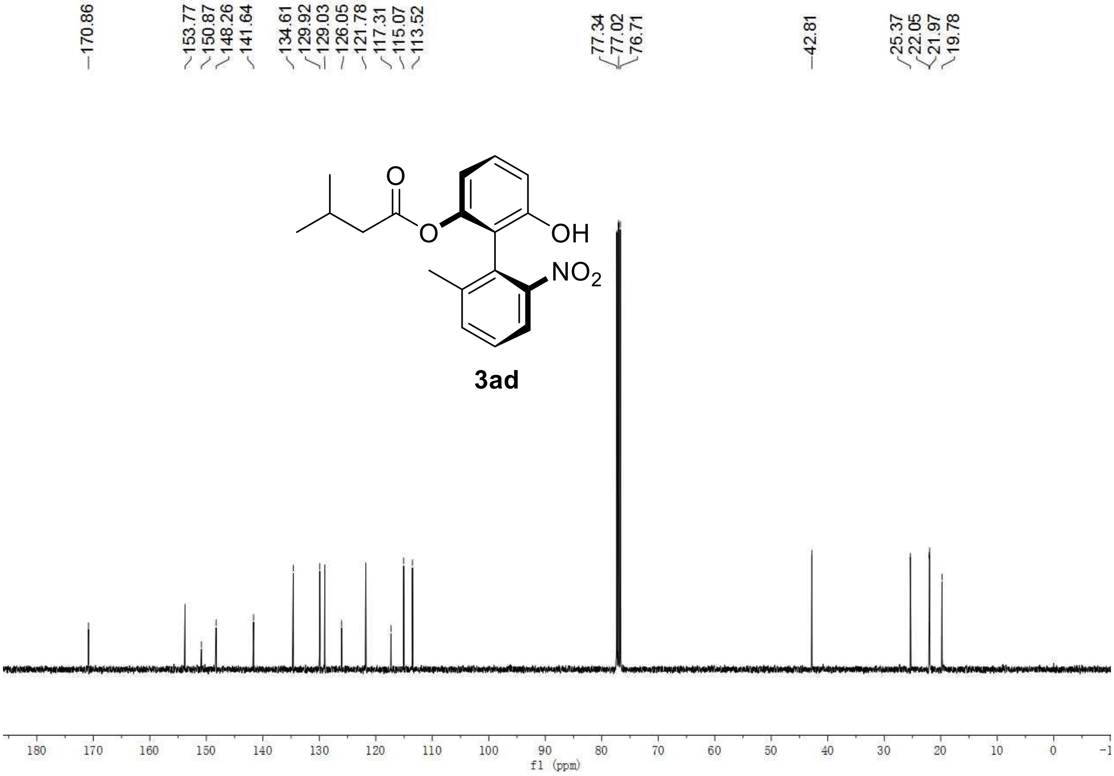

Supplementary Figure 15. ^{13}C NMR Spectrum of 3ad.

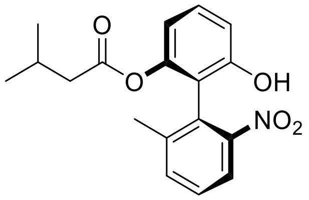

3ad

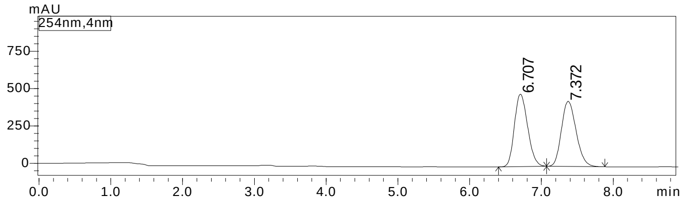

Peak	Ret. Time	Area	Height	Area%	Height%
1	6.707	6217409	484362	49.980	52.640
2	7.372	6222407	435784	50.020	47.360
Tolal		12439815	920146	100.000	100.000

Supplementary Figure 16. HPLC Spectrum of racemic 3ad.

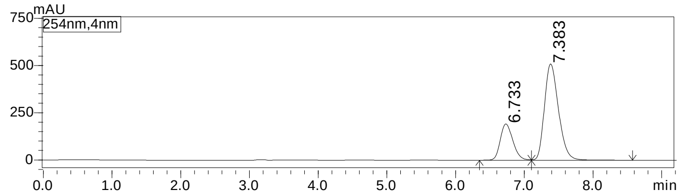

Peak	Ret. Time	Area	Height	Area%	Height%
1	6.733	2450888	191886	25.270	27.354
2	7.383	7247911	509593	74.730	72.646
Tolal		9698799	701480	100.000	100.000

Supplementary Figure 17. HPLC Spectrum of 3ad.

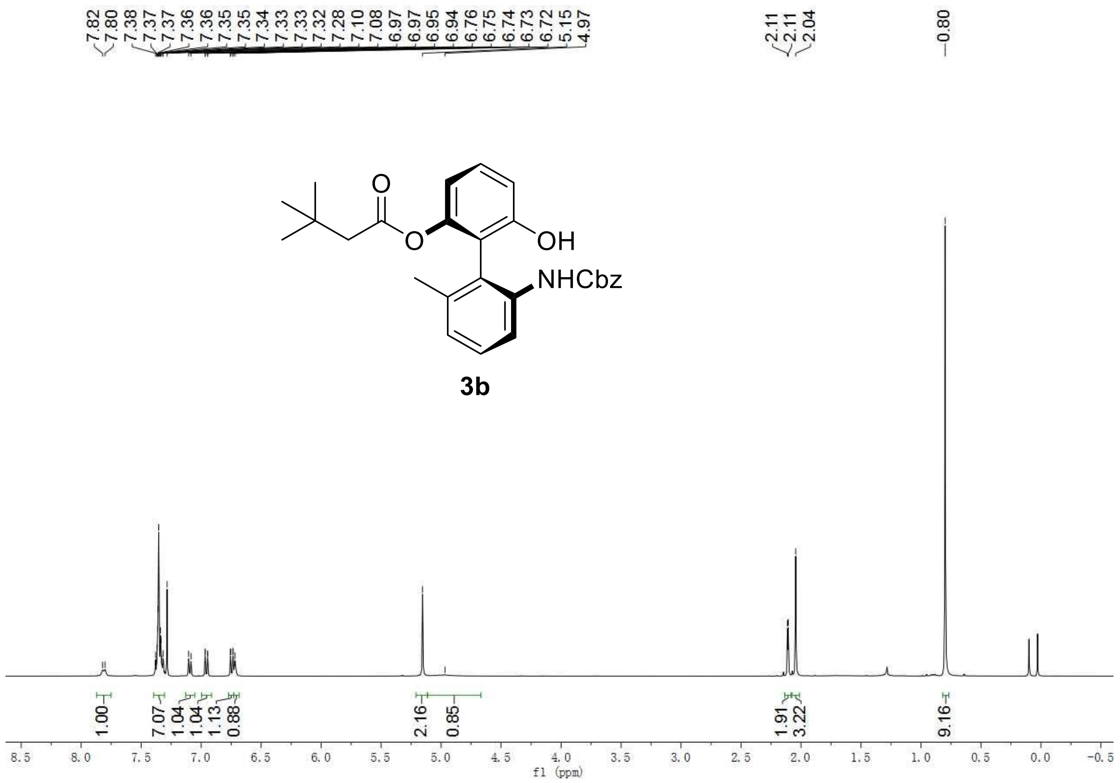

Supplementary Figure 18. ^{1}H NMR Spectrum of 3b.

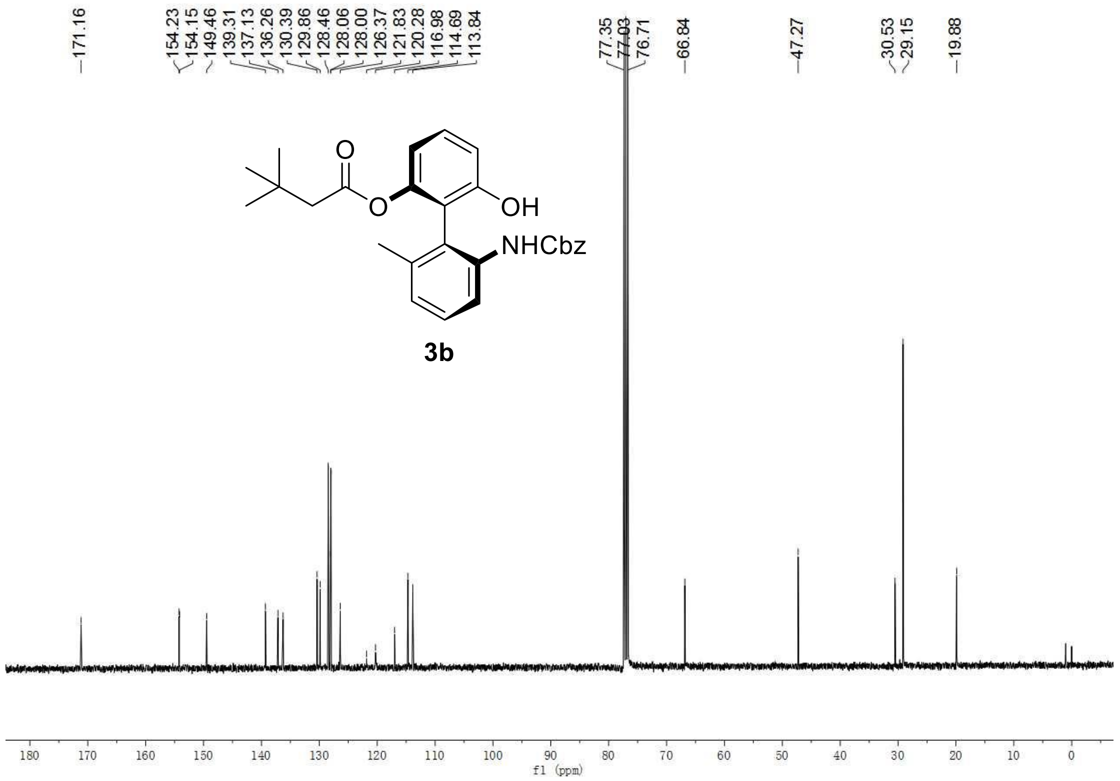

Supplementary Figure 19. ^{13}C NMR Spectrum of 3b.

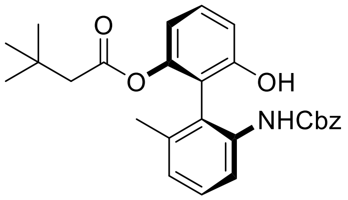

3b

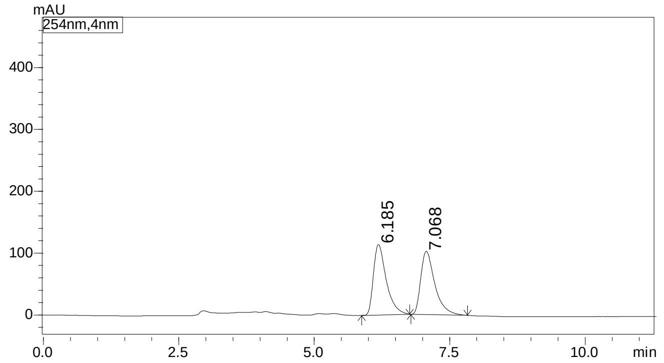

Peak	Ret. Time	Area	Height	Area%	Height%
1	6.185	1883049	114096	50.325	52.681
2	7.068	1858697	102483	49.675	47.319
Tolal		3741746	216579	100.000	100.000

Supplementary Figure 20. HPLC Spectrum of racemic 3b.

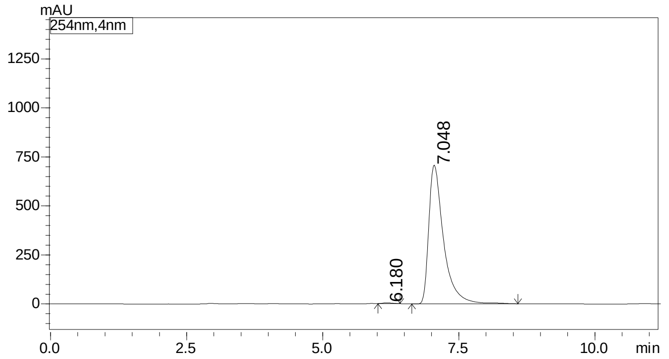

Peak	Ret. Time	Area	Height	Area%	Height%
1	6.180	53195	4271	0.395	0.600
2	7.048	13403035	706995	99.605	99.400
Tolal		13456229	711266	100.000	100.000

Supplementary Figure 21. HPLC Spectrum of 3b.

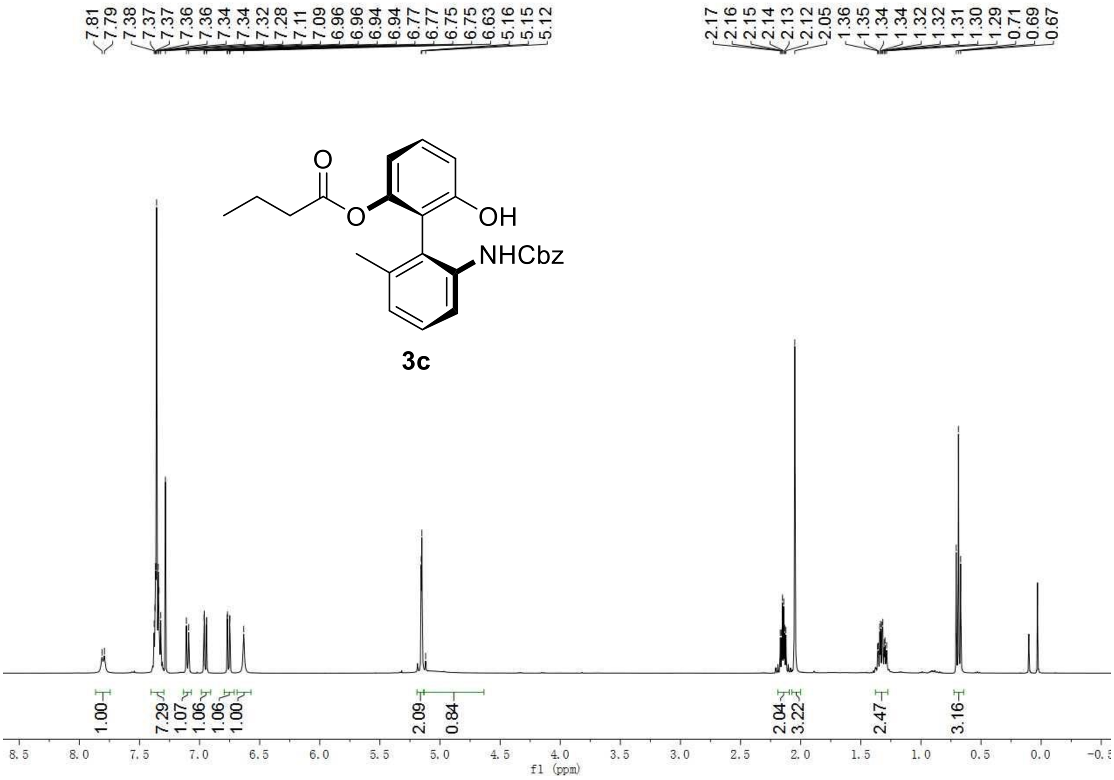

Supplementary Figure 22. ^{1}H NMR Spectrum of 3c.

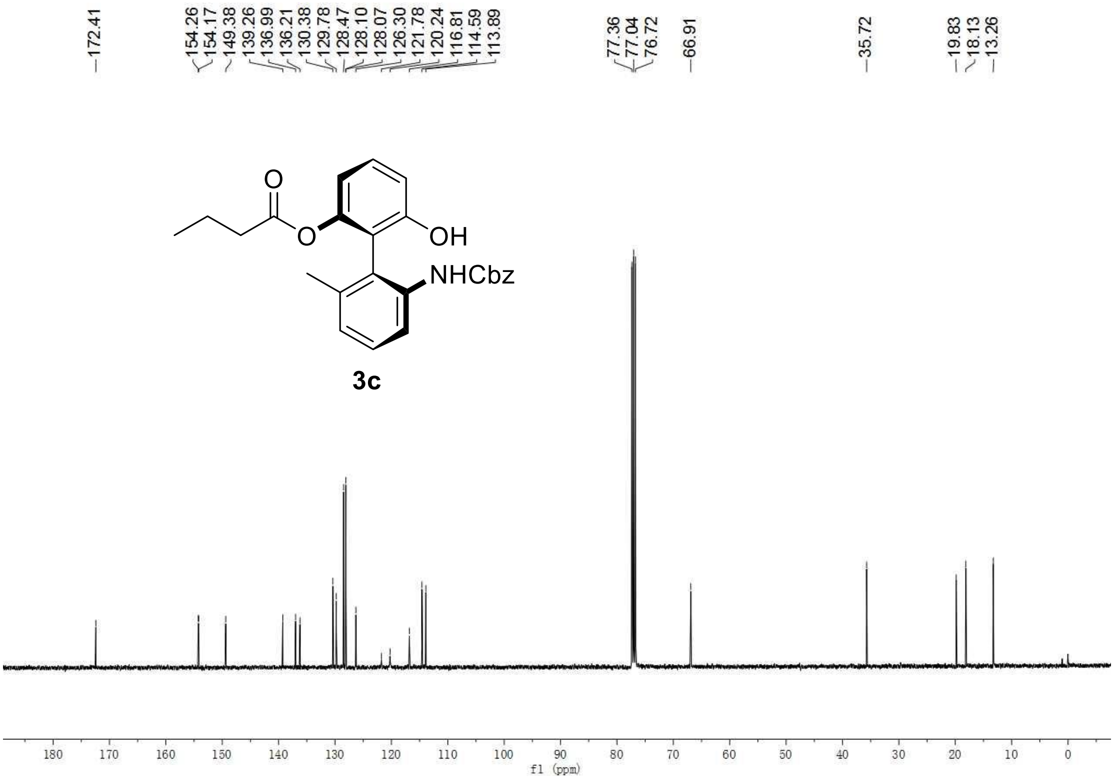

Supplementary Figure 23. ^{13}C NMR Spectrum of 3c.

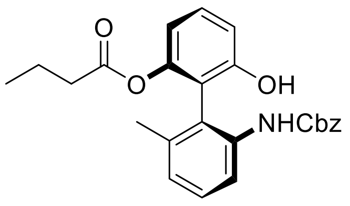

3c

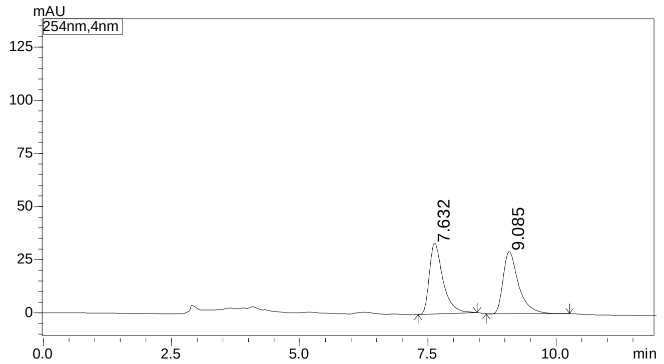

Peak	Ret. Time	Area	Height	Area%	Height%
1	7.632	618564	33284	50.380	53.253
2	9.085	609238	29217	49.620	46.747
Tolal		1227802	62501	100.000	100.000

Supplementary Figure 24. HPLC Spectrum of racemic 3c.

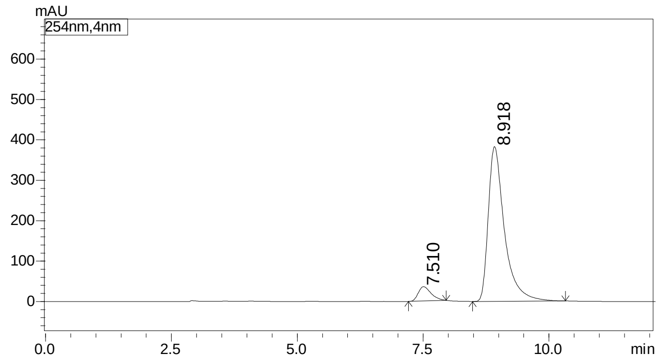

Peak	Ret. Time	Area	Height	Area%	Height%
1	7.510	626934	35457	6.832	8.470
2	8.918	8550070	383144	93.168	91.530
Tolal		9177003	418601	100.000	100.000

Supplementary Figure 25. HPLC Spectrum of 3c.

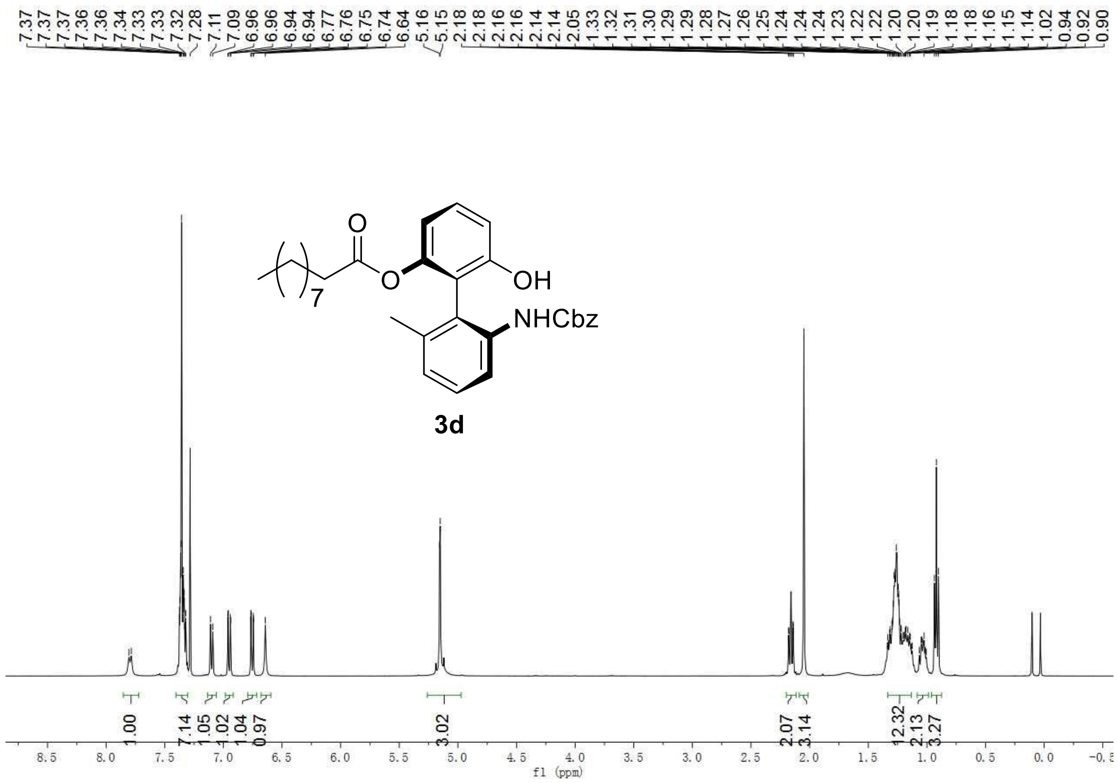

Supplementary Figure 26. ^{1}H NMR Spectrum of 3d.

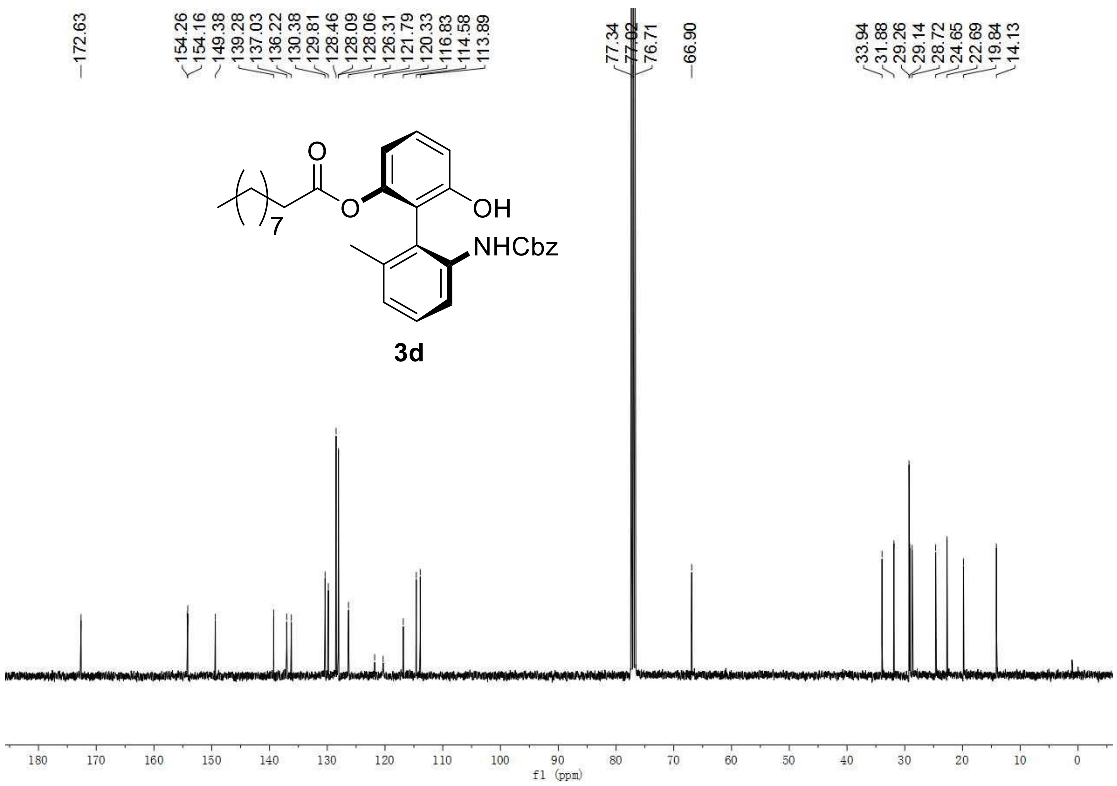

Supplementary Figure 27. ^{13}C NMR Spectrum of 3d.

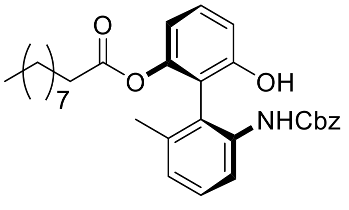

3d

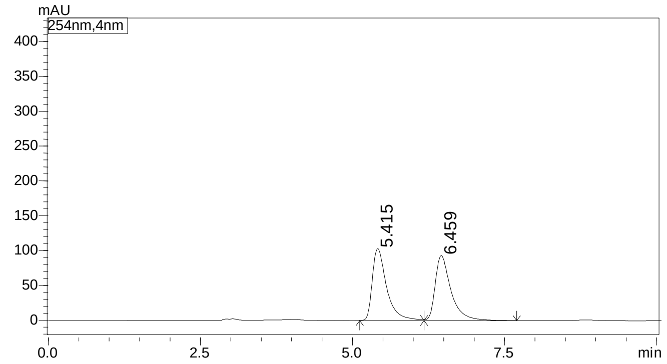

Peak	Ret. Time	Area	Height	Area%	Height%
1	5.415	1689667	103120	50.209	52.542
2	6.459	1675612	93141	49.791	47.458
Tolal		3365278	196261	100.000	100.000

Supplementary Figure 28. HPLC Spectrum of racemic 3d.

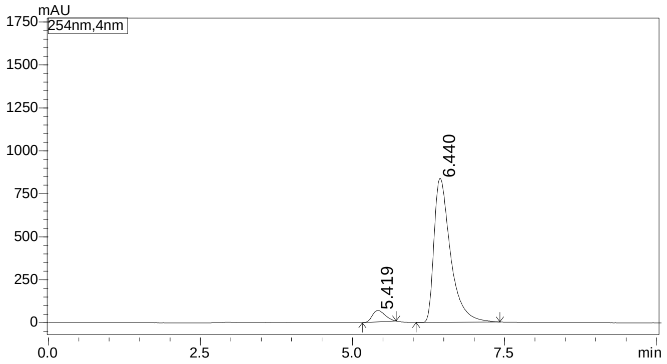

Peak	Ret. Time	Area	Height	Area%	Height%
1	5.419	966010	65899	5.772	7.289
2	6.440	15771495	838220	94.228	92.711
Tolal		16737505	904119	100.000	100.000

Supplementary Figure 29. HPLC Spectrum of 3d.

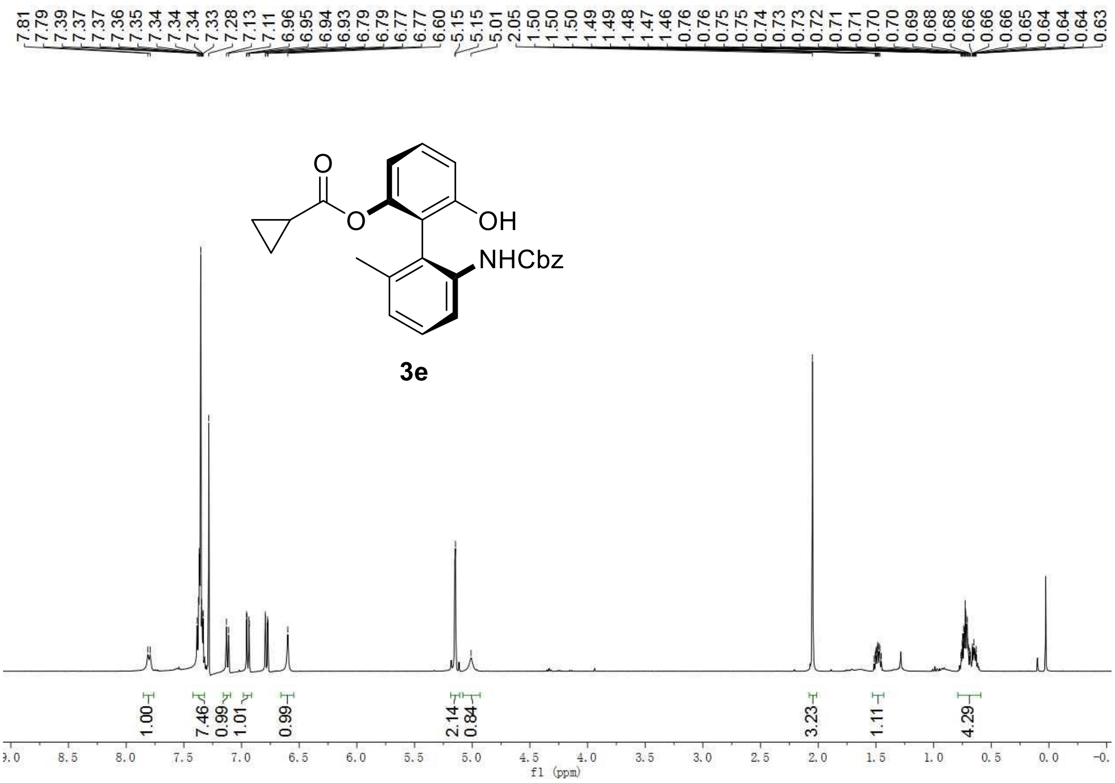

Supplementary Figure 30. ^{1}H NMR Spectrum of 3e.

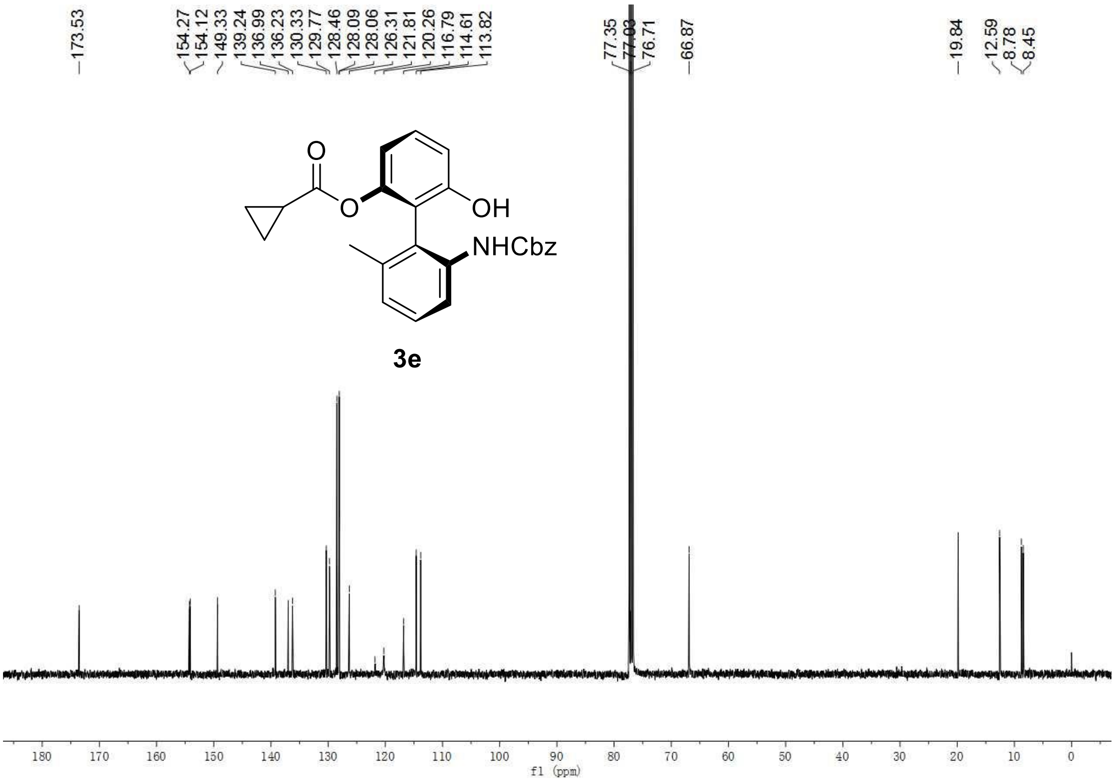

Supplementary Figure 31. ^{13}C NMR Spectrum of 3e.

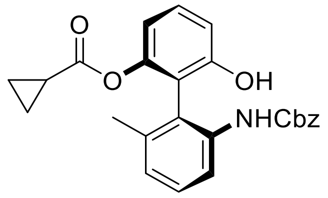

3e

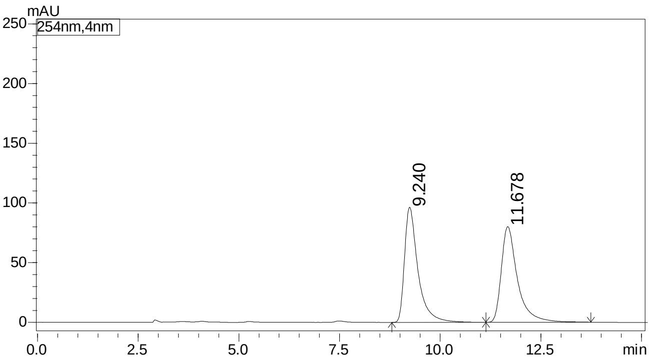

Peak	Ret. Time	Area	Height	Area%	Height%
1	9.240	2243485	96481	50.068	54.627
2	11.678	2237398	80138	49.932	45.373
Tolal		4480884	176619	100.000	100.000

Supplementary Figure 32. HPLC Spectrum of racemic 3e.

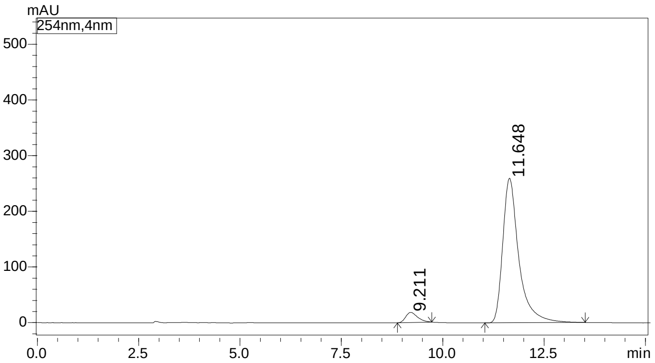

Peak	Ret. Time	Area	Height	Area%	Height%
1	9.211	368429	18104	4.889	6.519
2	11.648	7166898	259611	95.111	93.481
Tolal		7535327	277714	100.000	100.000

Supplementary Figure 33. HPLC Spectrum of 3e.

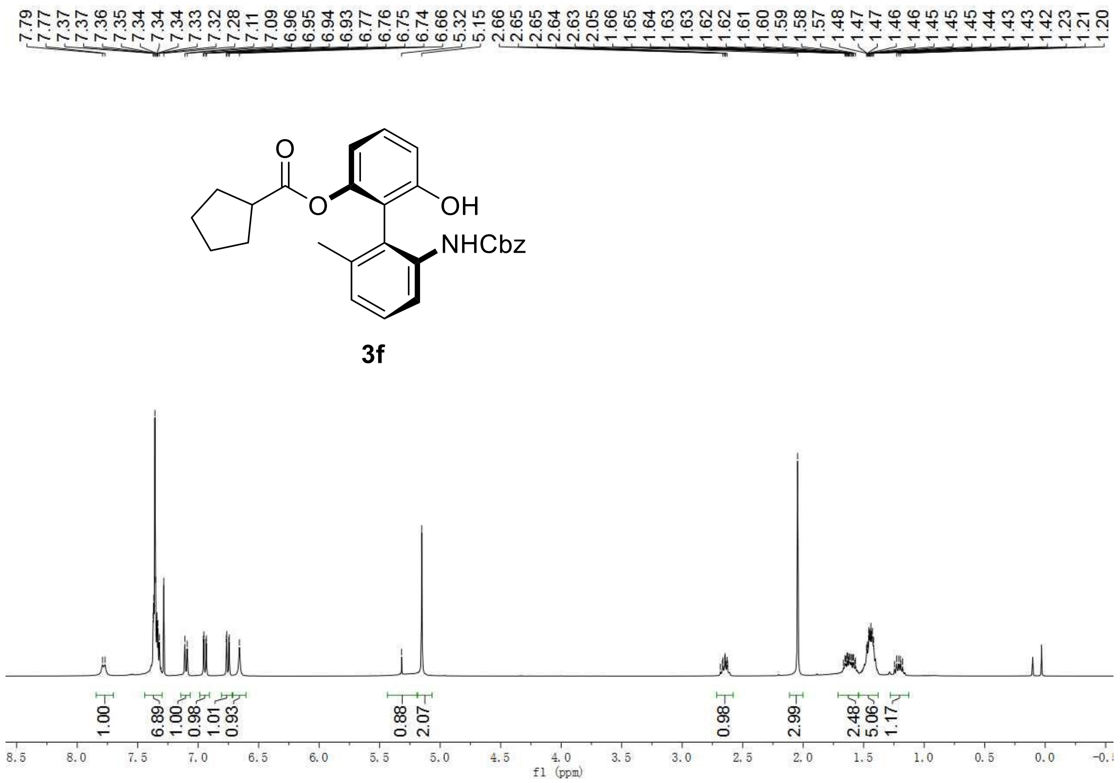

Supplementary Figure 34. ^{1}H NMR Spectrum of 3f.

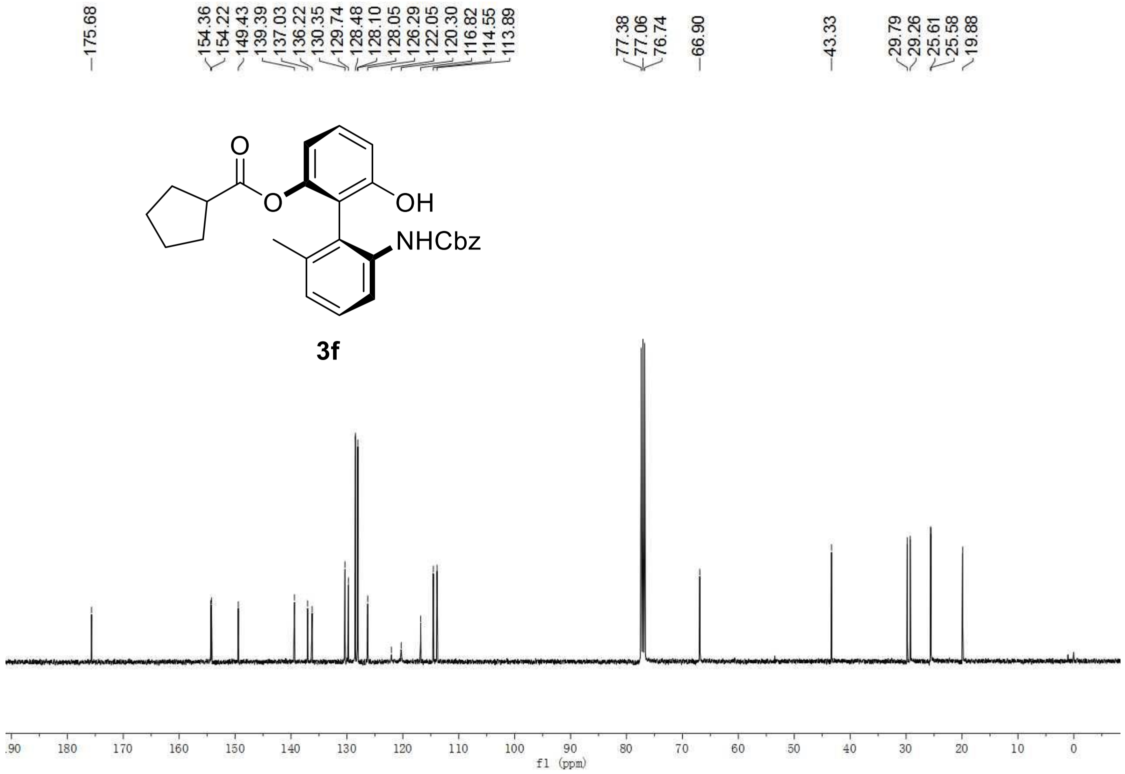

Supplementary Figure 35. ^{13}C NMR Spectrum of 3f.

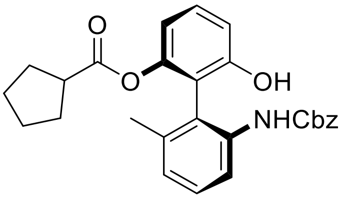

3f

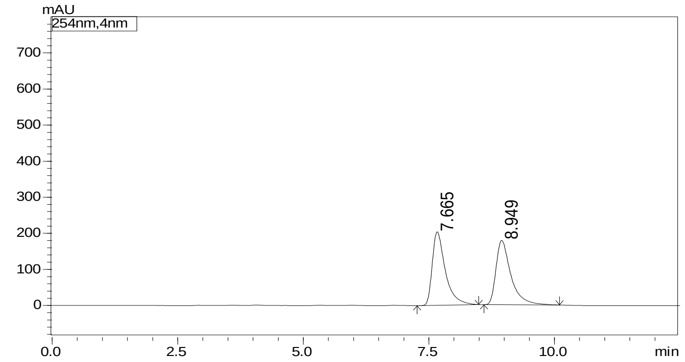

Peak	Ret. Time	Area	Height	Area%	Height%
1	7.665	3684050	203435	50.022	53.269
2	8.949	3680874	178467	49.978	46.731
Tolal		7364924	381902	100.000	100.000

Supplementary Figure 36. HPLC Spectrum of racemic 3f.

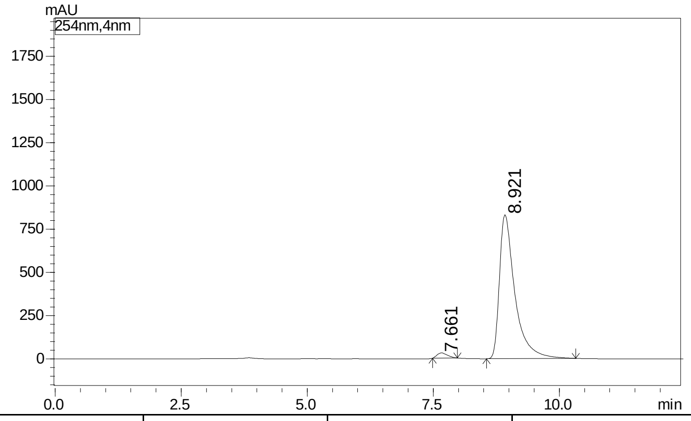

Peak	Ret. Time	Area	Height	Area%	Height%
1	7.661	423115	29664	2.330	3.445
2	8.921	17737664	831375	97.670	96.555
Tolal		18160780	861039	100.000	100.000

Supplementary Figure 37. HPLC Spectrum of 3f.

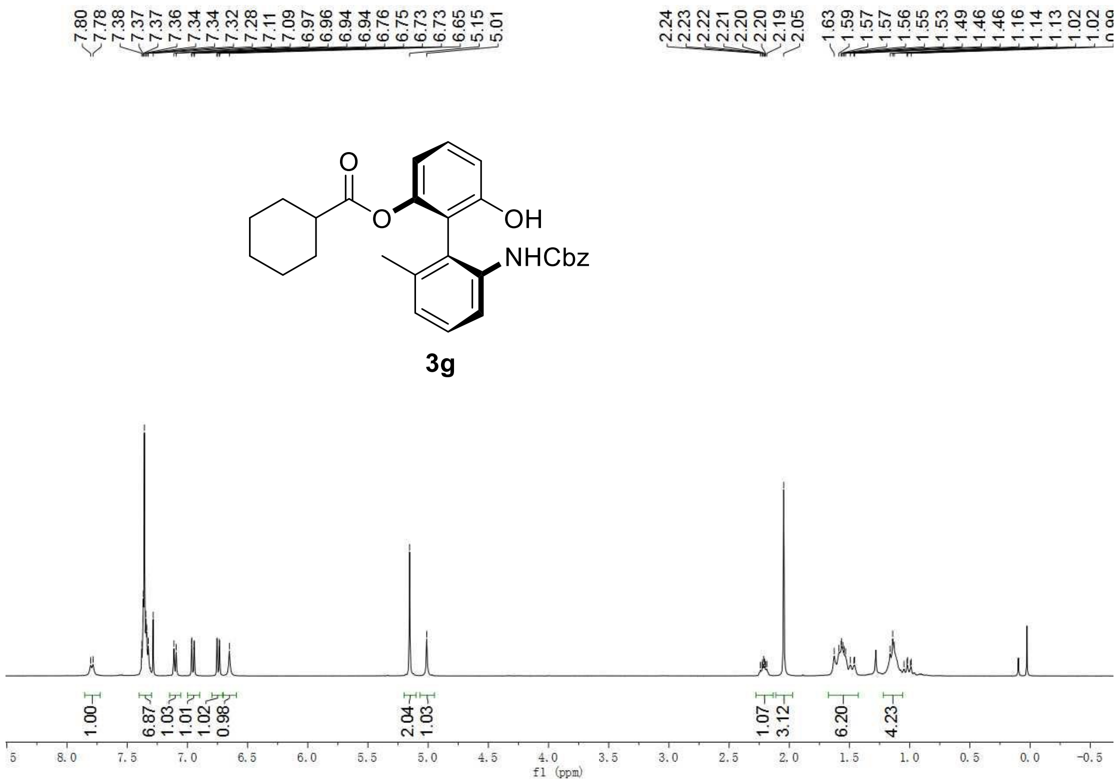

Supplementary Figure 38. ^{1}H NMR Spectrum of 3g.

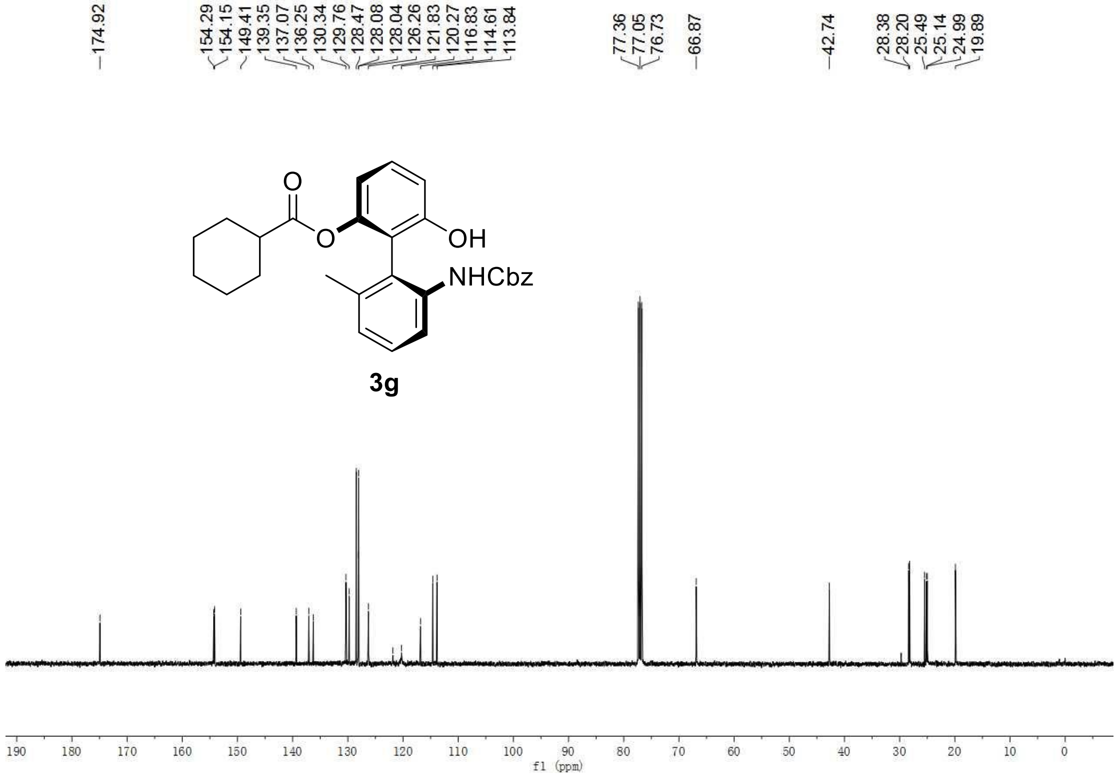

Supplementary Figure 39. ^{13}C NMR Spectrum of 3g.

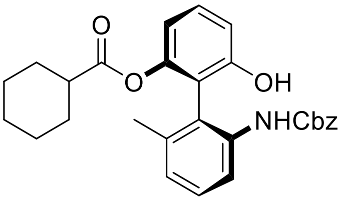

3g

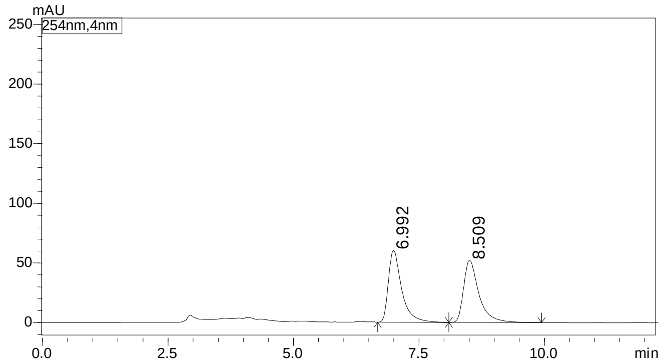

Peak	Ret. Time	Area	Height	Area%	Height%
1	6.992	1103095	60142	50.135	53.629
2	8.509	1097174	52003	49.865	46.371
Tolal		2200269	112144	100.000	100.000

Supplementary Figure 40. HPLC Spectrum of racemic 3g.

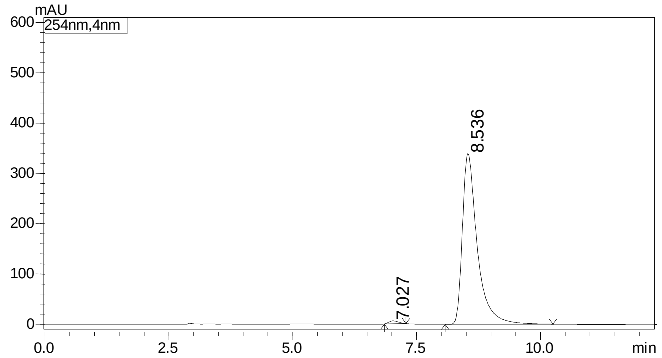

Peak	Ret. Time	Area	Height	Area%	Height%
1	7.027	77530	5778	1.084	1.675
2	8.536	7074858	339183	98.916	98.325
Tolal		7152388	344961	100.000	100.000

Supplementary Figure 41. HPLC Spectrum of 3g.

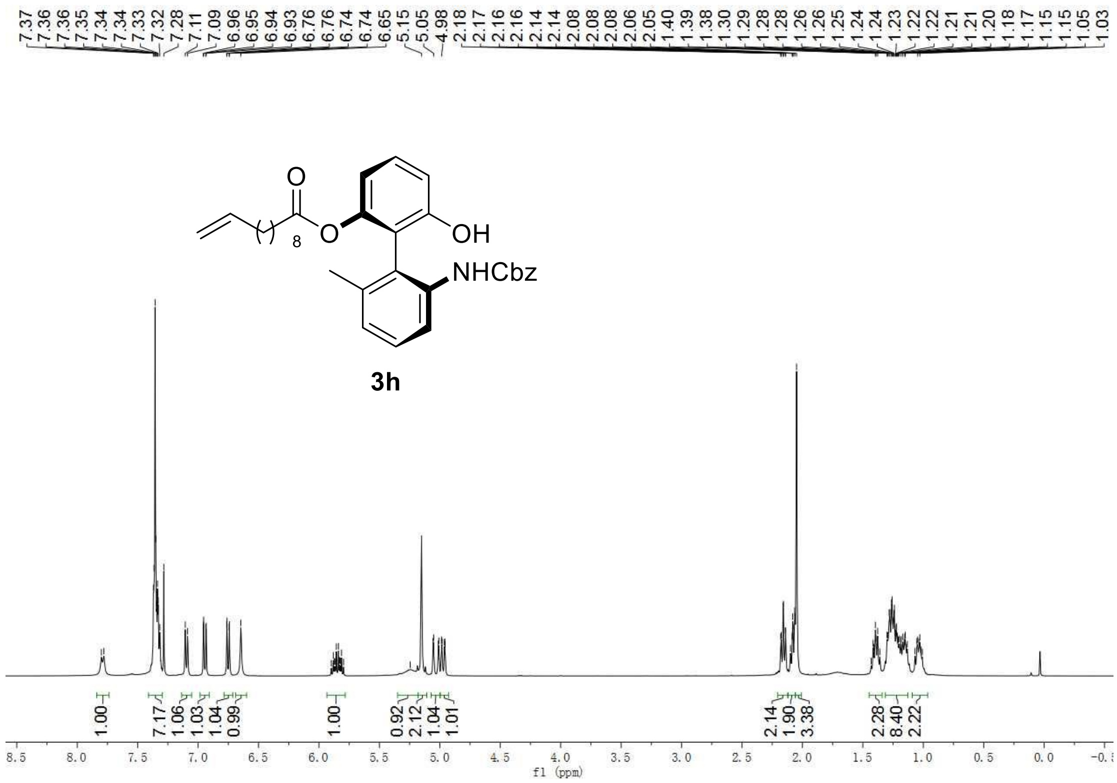

Supplementary Figure 42. ^{1}H NMR Spectrum of 3h.

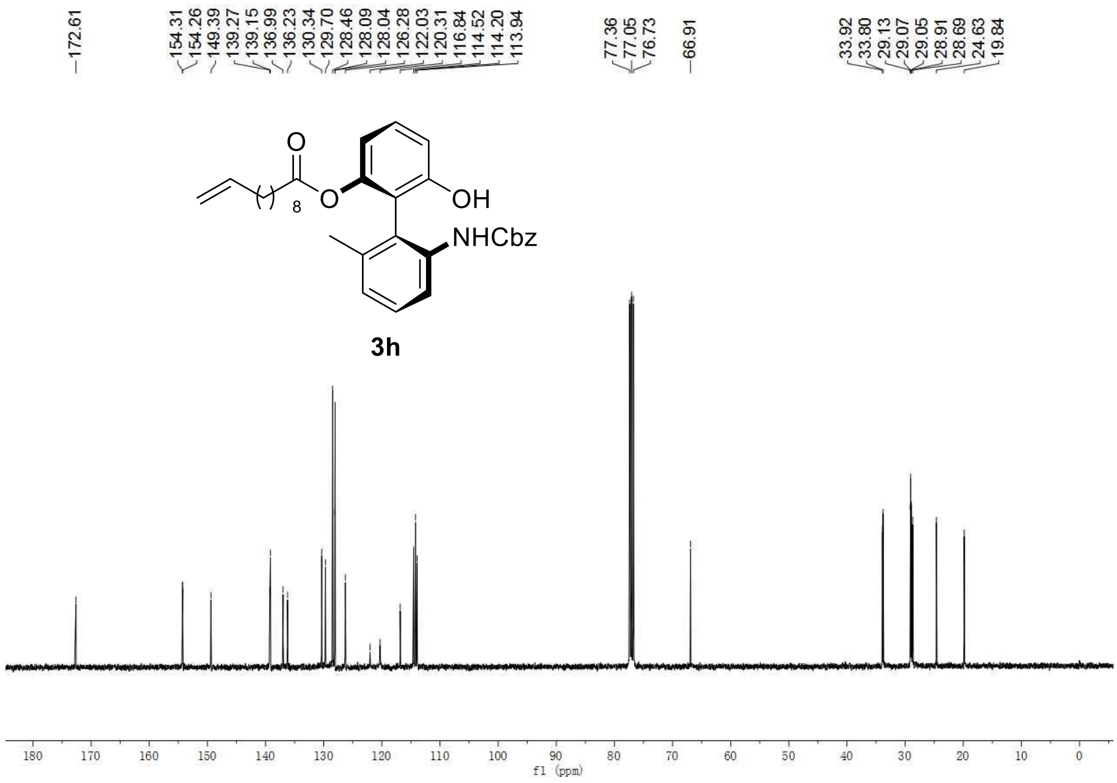

Supplementary Figure 43. ^{13}C NMR Spectrum of 3h.

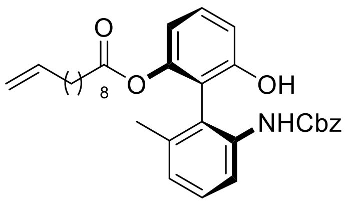

3h

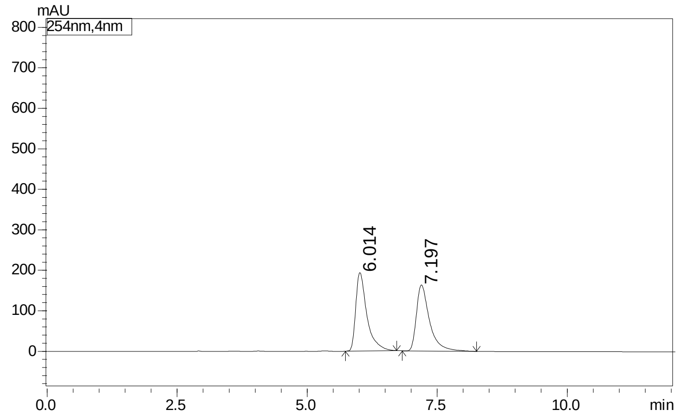

Peak	Ret. Time	Area	Height	Area%	Height%
1	6.014	2887287	193847	50.299	54.325
2	7.197	2853005	162982	49.701	45.675
Tolal		5740292	356829	100.000	100.000

Supplementary Figure 44. HPLC Spectrum of racemic 3h.

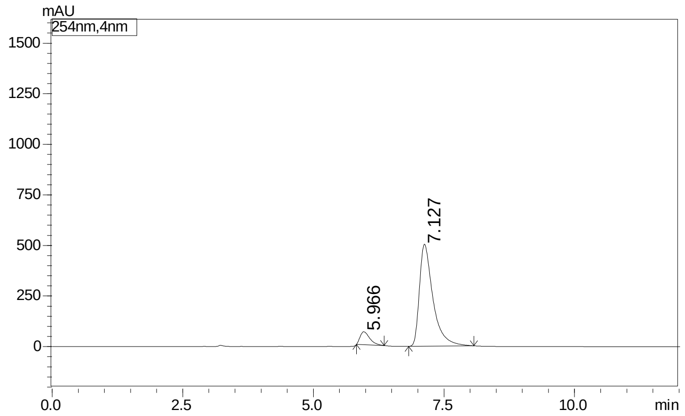

Peak	Ret. Time	Area	Height	Area%	Height%
1	5.966	819397	64025	8.272	11.264
2	7.127	9086092	504353	91.728	88.736
Tolal		9905489	568378	100.000	100.000

Supplementary Figure 45. HPLC Spectrum of 3h.

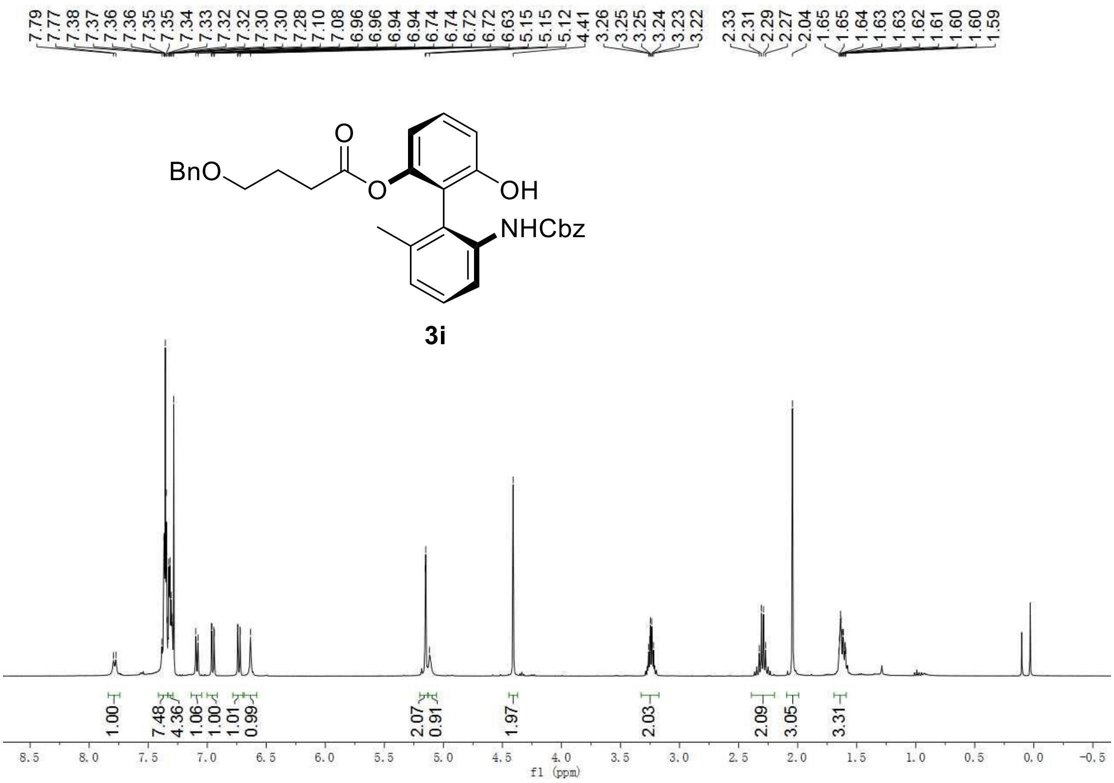

Supplementary Figure 46. ^{1}H NMR Spectrum of 3i.

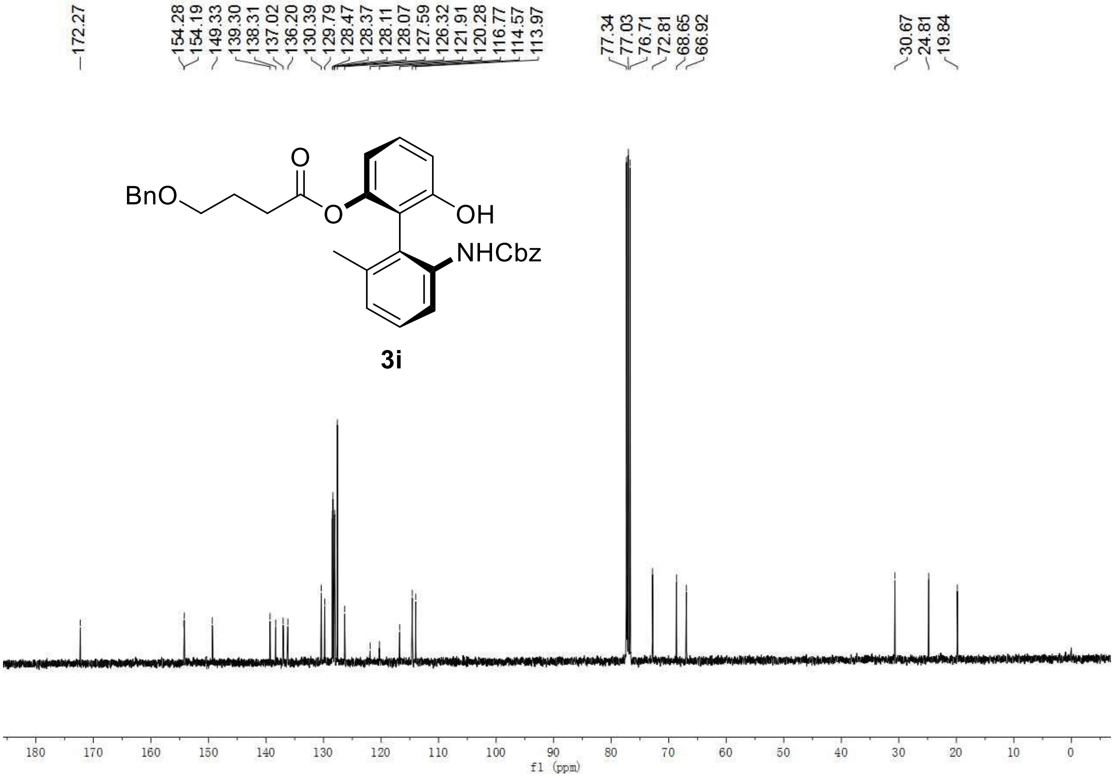

Supplementary Figure 47. ^{13}C NMR Spectrum of 3i.

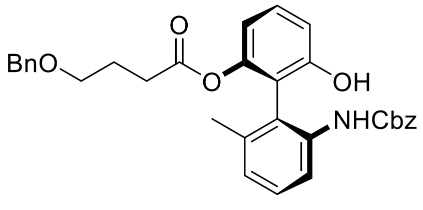

3i

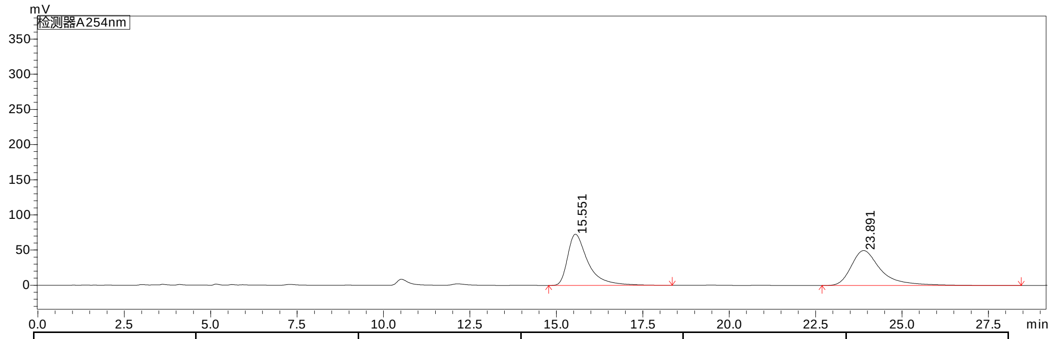

Peak	Ret. Time	Area	Height	Area%	Height%
1	15.551	2990139	72818	50.041	59.441
2	23.891	2985255	49686	49.959	40.559
Tolal		5975394	122503	100.000	100.000

Supplementary Figure 48. HPLC Spectrum of racemic 3i.

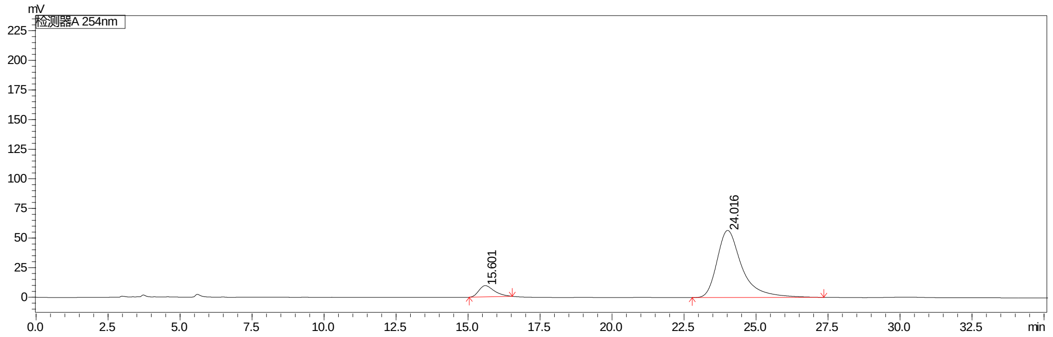

Peak	Ret. Time	Area	Height	Area%	Height%
1	15.601	354562	9536	9.411	14.400
2	24.016	3413169	56686	90.589	85.600
Tolal		3767731	66222	100.000	100.000

Supplementary Figure 49. HPLC Spectrum of 3i.

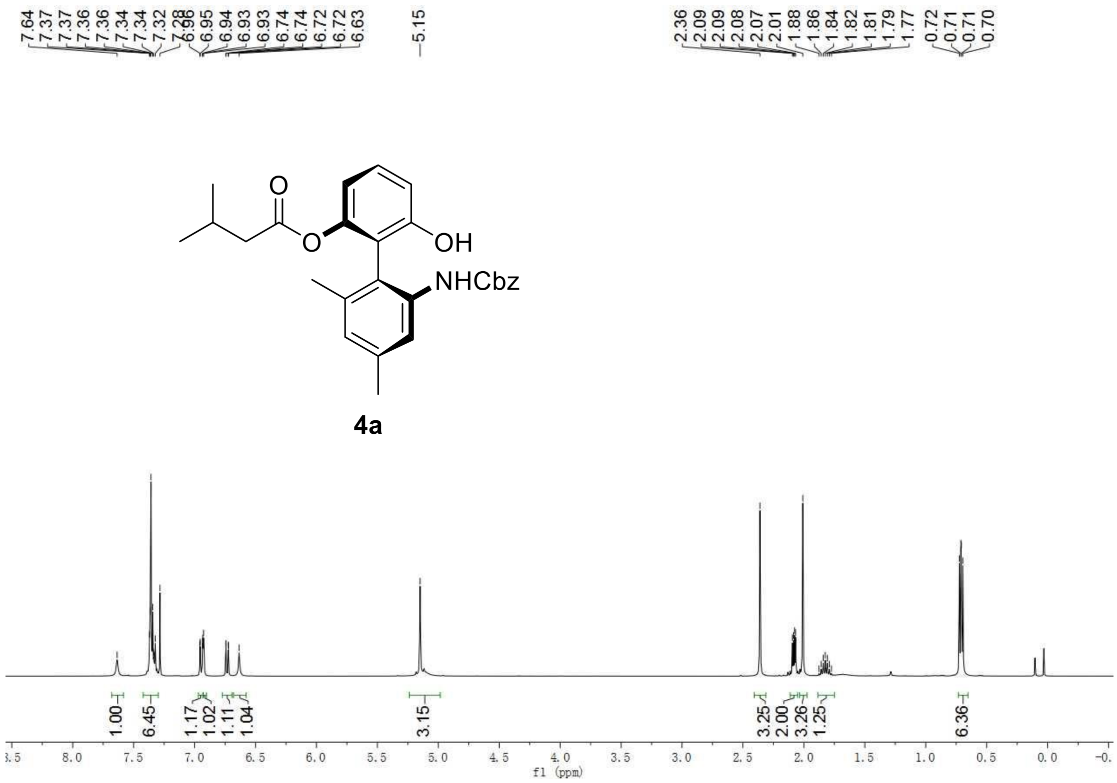

Supplementary Figure 50. ^{1}H NMR Spectrum of 4a.

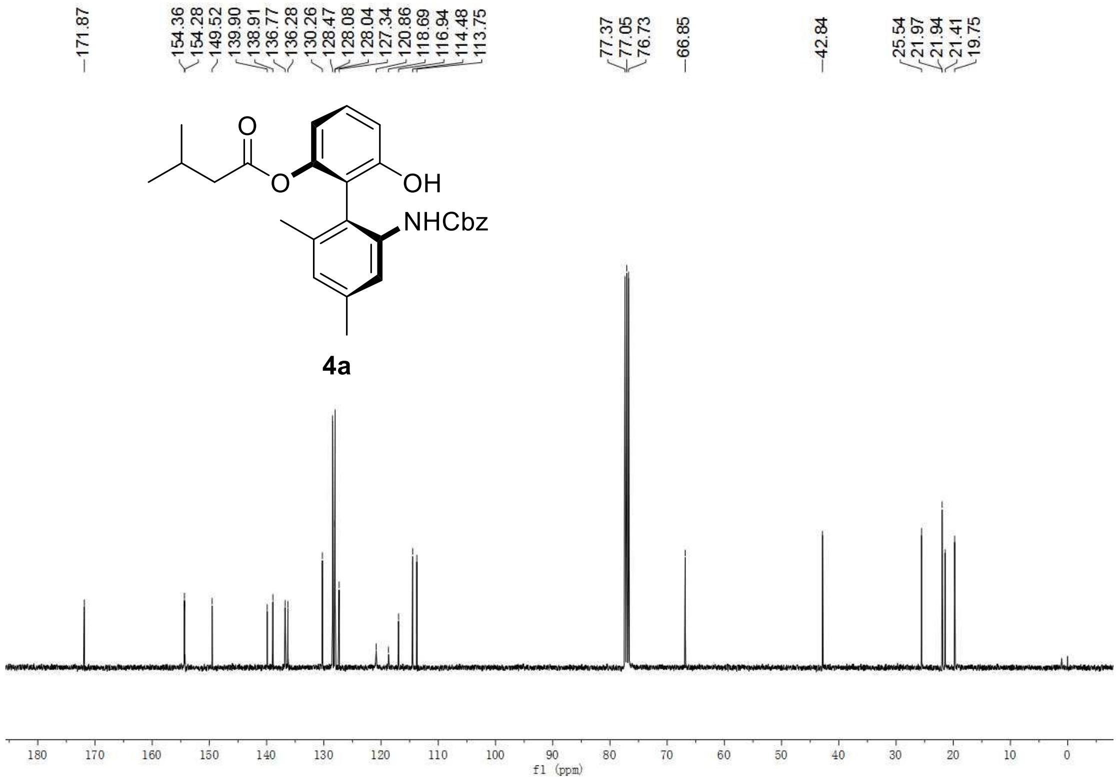

Supplementary Figure 51. ^{13}C NMR Spectrum of 4a.

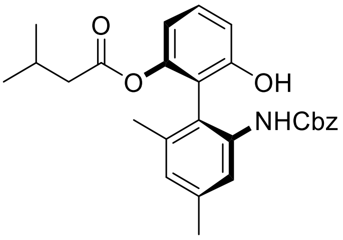

4a

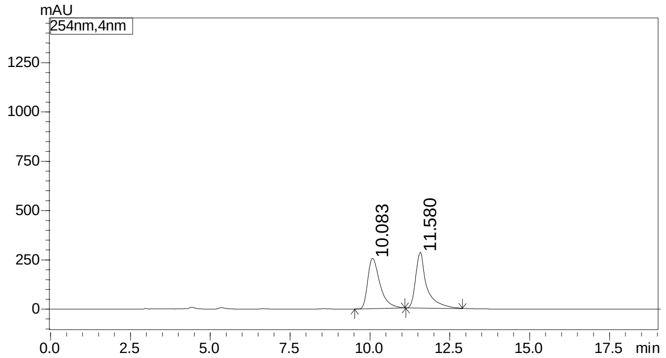

Peak	Ret. Time	Area	Height	Area%	Height%
1	10.083	6789386	254991	49.986	47.441
2	11.580	6793313	282503	50.014	52.559
Tolal		13582698	537494	100.000	100.000

Supplementary Figure 52. HPLC Spectrum of racemic 4a.

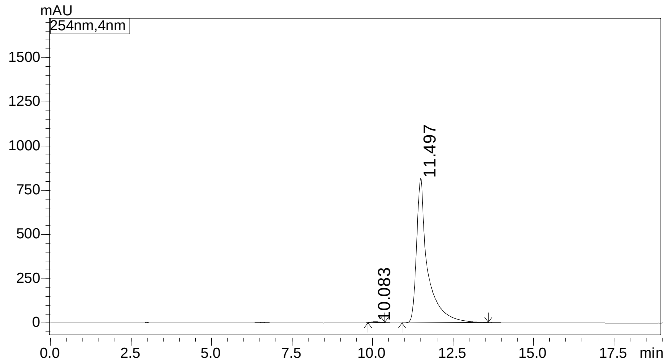

Peak	Ret. Time	Area	Height	Area%	Height%
1	10.083	88322	4812	0.450	0.586
2	11.497	19549165	815728	99.550	99.414
Tolal		19637486	820540	100.000	100.000

Supplementary Figure 53. HPLC Spectrum of 4a.

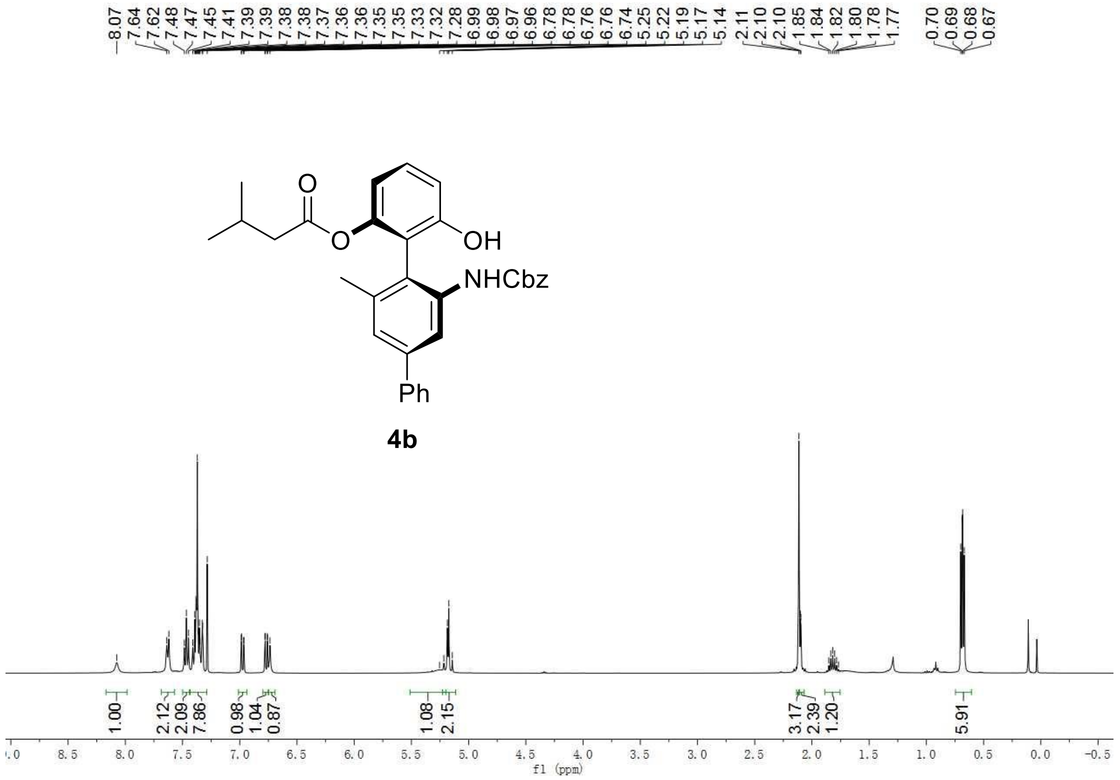

Supplementary Figure 54. ^{1}H NMR Spectrum of 4b.

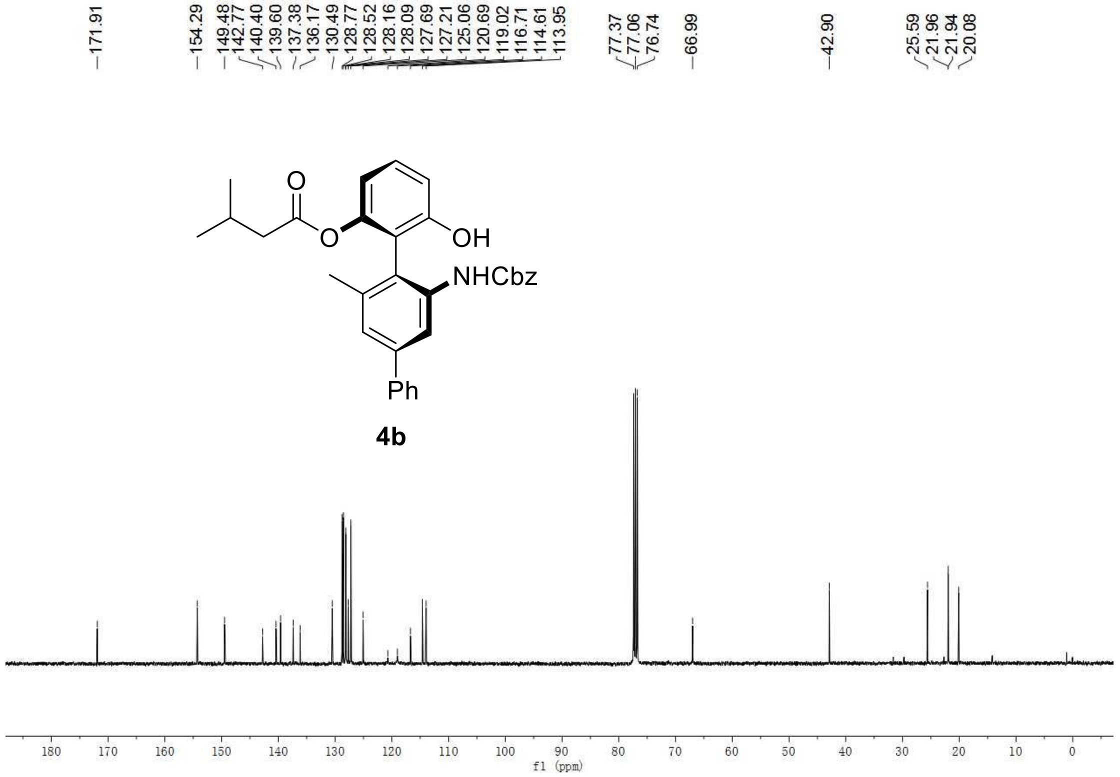

Supplementary Figure 55. ^{13}C NMR Spectrum of 4b.

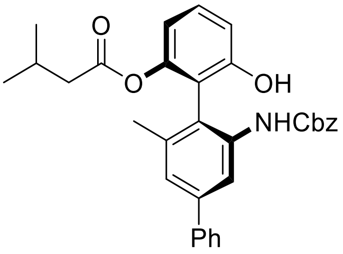

4b

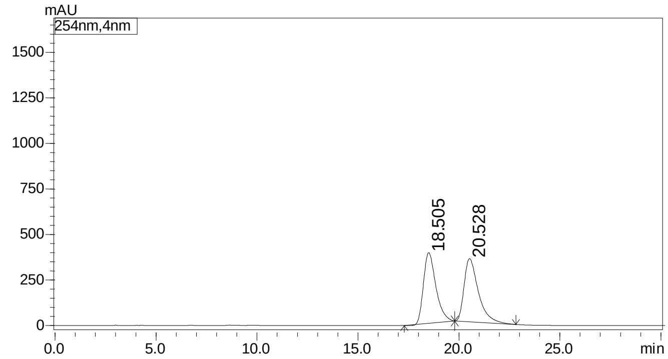

Peak	Ret. Time	Area	Height	Area%	Height%
1	18.505	16645281	388195	49.353	52.795
2	20.528	17081625	347086	50.647	47.205
Tolal		33726906	735281	100.000	100.000

Supplementary Figure 56. HPLC Spectrum of racemic 4b.

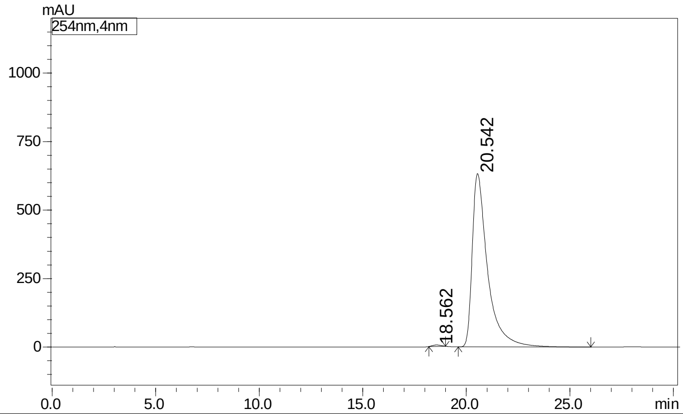

Peak	Ret. Time	Area	Height	Area%	Height%
1	18.562	145588	5285	0.444	0.828
2	20.542	32649615	632674	99.556	99.172
Tolal		32795202	637959	100.000	100.000

Supplementary Figure 57. HPLC Spectrum of 4b.

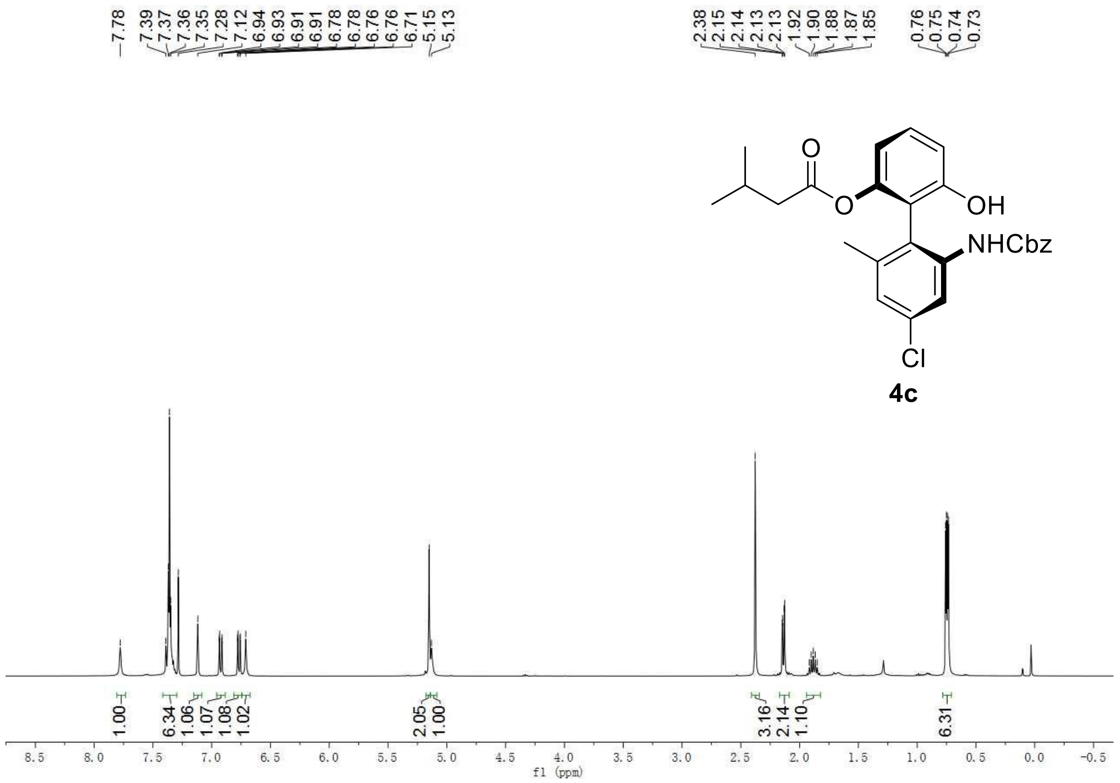

Supplementary Figure 58. ^{1}H NMR Spectrum of 4c.

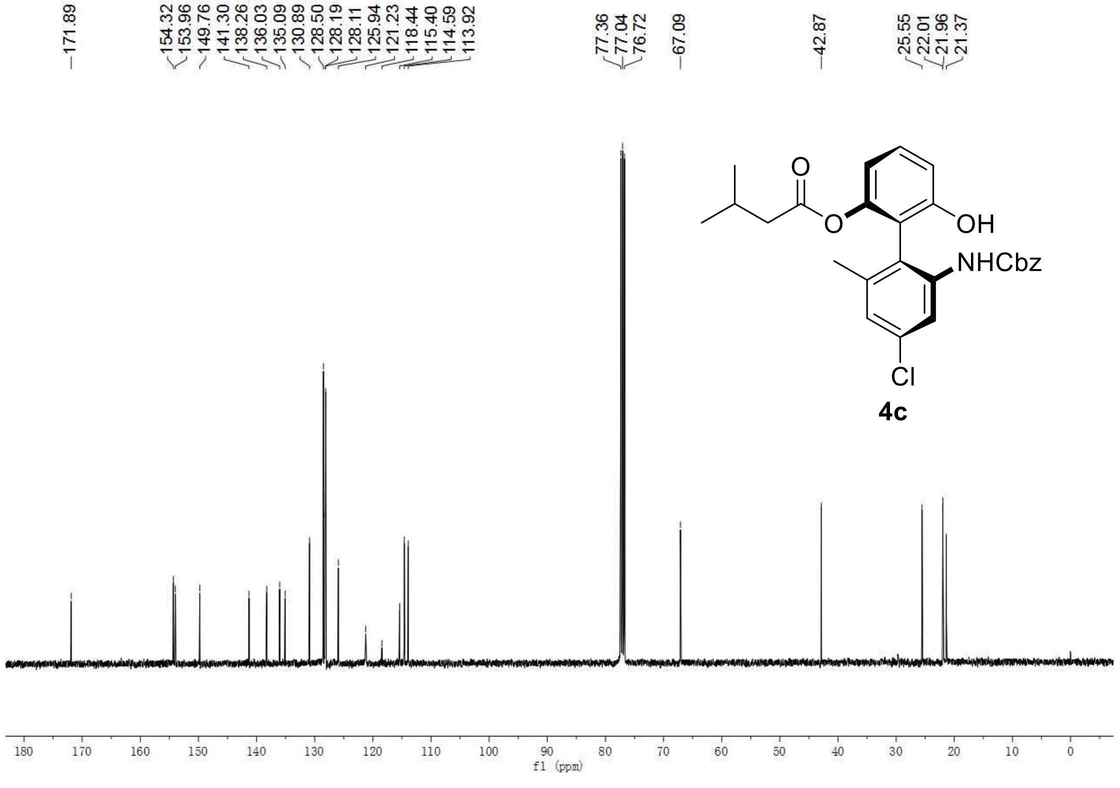

Supplementary Figure 59. ^{13}C NMR Spectrum of 4c.

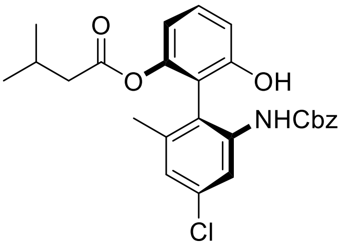

4c

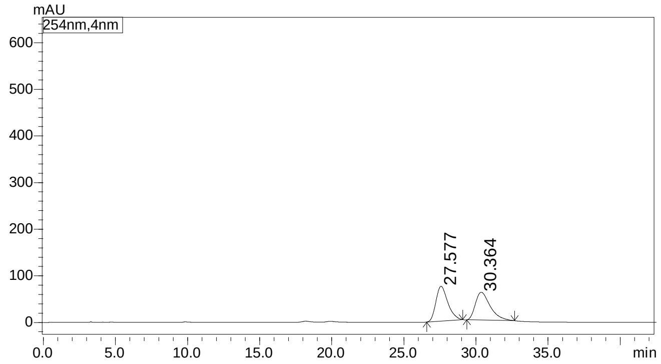

Peak	Ret. Time	Area	Height	Area%	Height%
1	27.577	4302296	74623	50.133	55.543
2	30.364	4279548	59729	49.867	44.457
Tolal		8581844	134352	100.000	100.000

Supplementary Figure 60. HPLC Spectrum of racemic 4c.

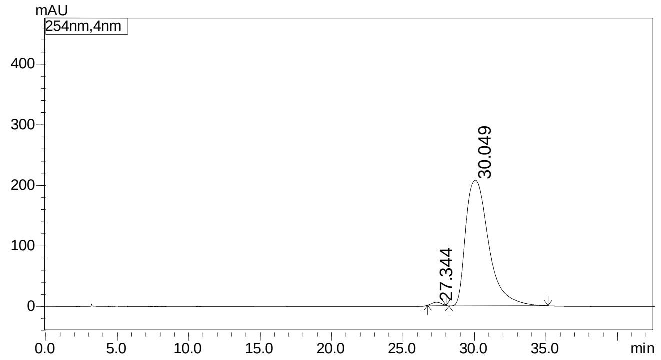

Peak	Ret. Time	Area	Height	Area%	Height%
1	27.344	224047	5127	0.952	2.411
2	30.049	23322419	207516	99.048	97.589
Tolal		23546466	212642	100.000	100.000

Supplementary Figure 61. HPLC Spectrum of 4c.

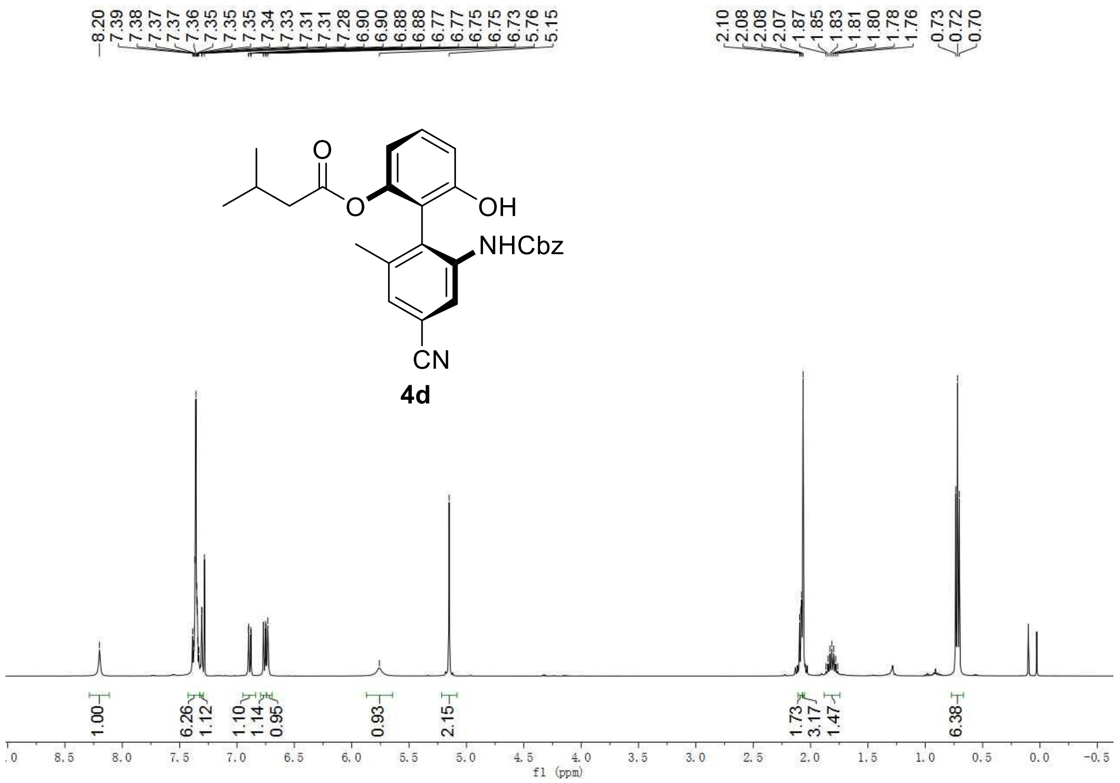

Supplementary Figure 62. ^{1}H NMR Spectrum of 4d.

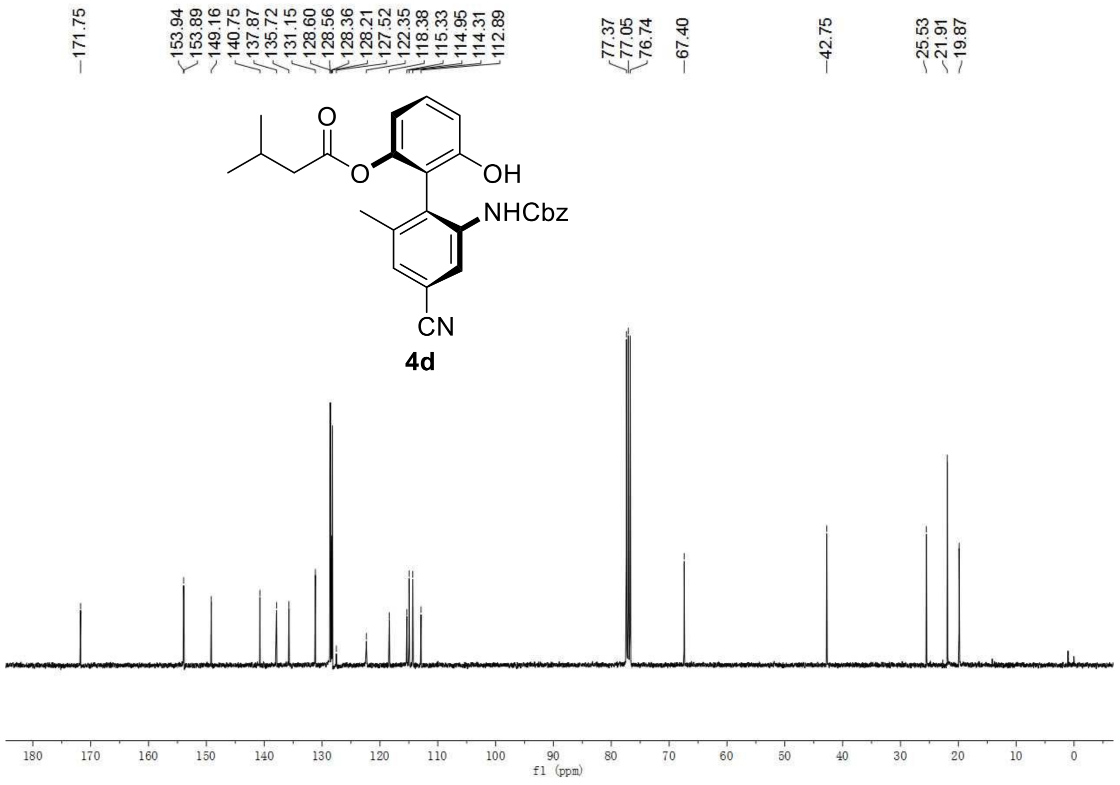

Supplementary Figure 63. ^{13}C NMR Spectrum of 4d.

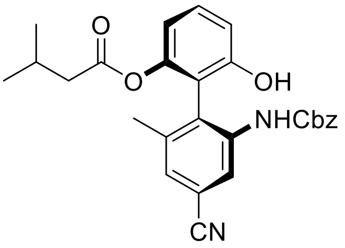

4d

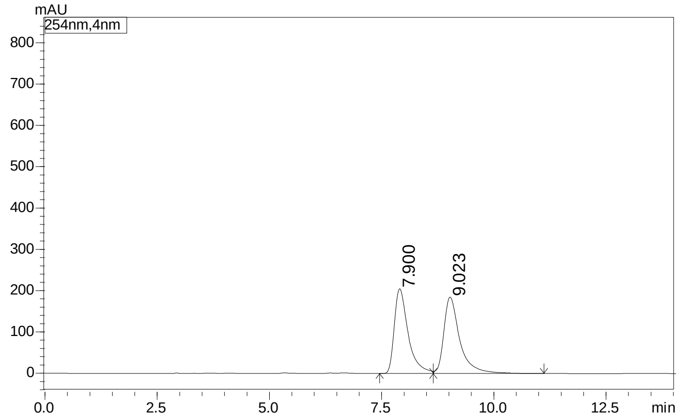

Peak	Ret. Time	Area	Height	Area%	Height%
1	7.900	4405560	205058	49.008	52.564
2	9.023	4583879	185053	50.992	47.436
Tolal		8989439	390111	100.000	100.000

Supplementary Figure 64. HPLC Spectrum of racemic 4d.

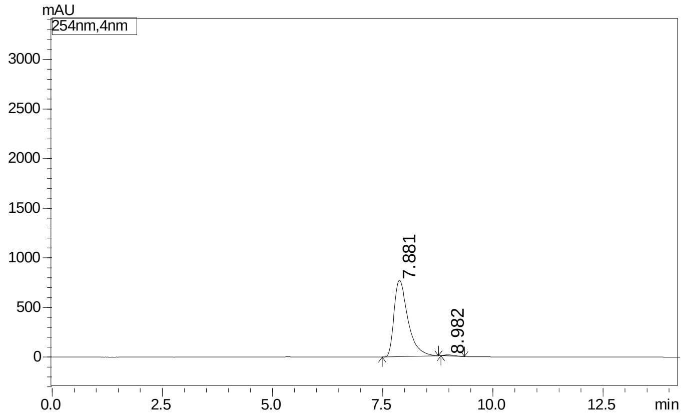

Peak	Ret. Time	Area	Height	Area%	Height%
1	7.881	16848697	769474	99.023	98.687
2	8.982	166239	10236	0.977	1.313
Tolal		17014935	779710	100.000	100.000

Supplementary Figure 65. HPLC Spectrum of 4d.

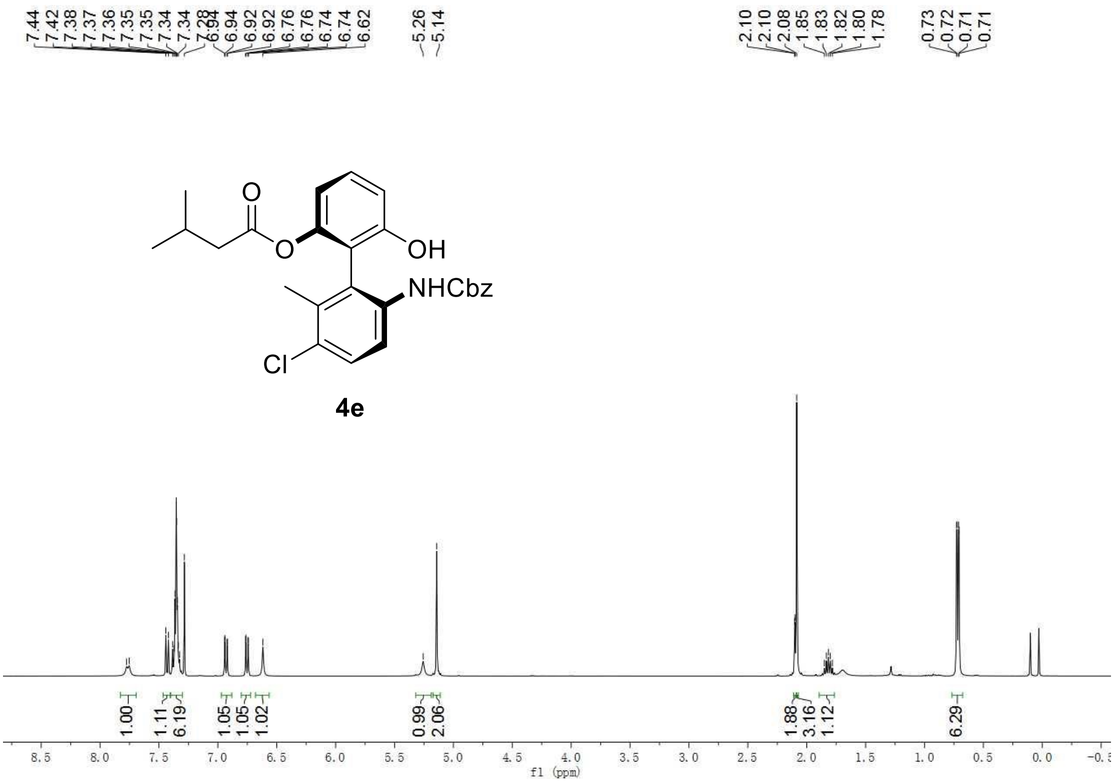

Supplementary Figure 66. ^{1}H NMR Spectrum of 4e.

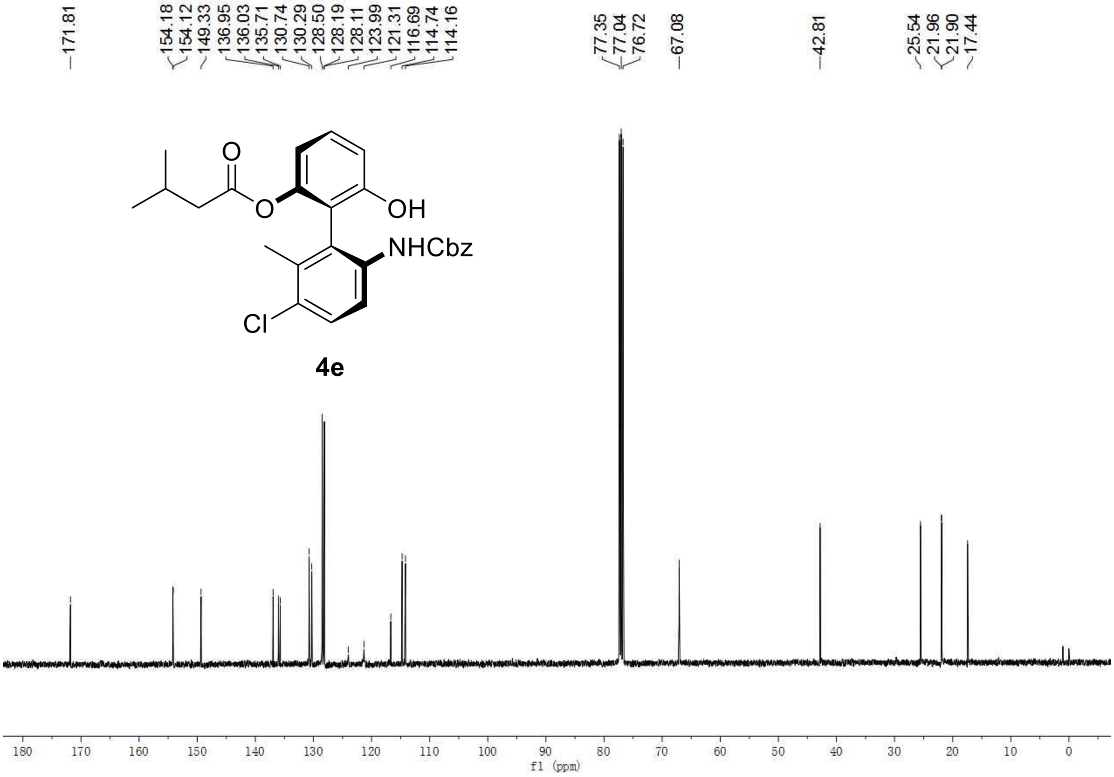

Supplementary Figure 67. ^{13}C NMR Spectrum of 4e.

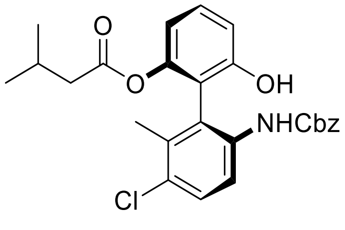

4e

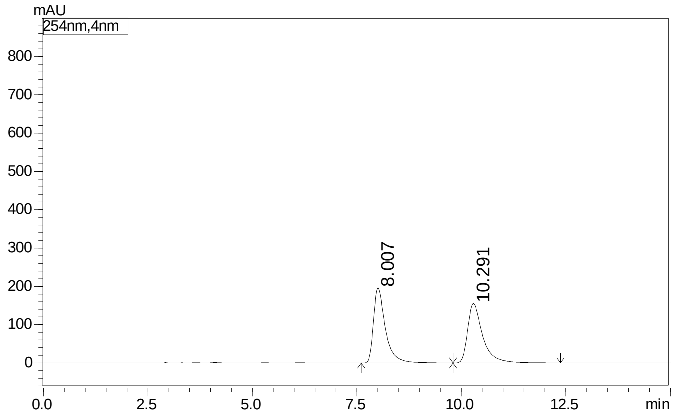

Peak	Ret. Time	Area	Height	Area%	Height%
1	8.007	3905675	196282	50.001	55.809
2	10.291	3905563	155423	49.999	44.191
Tolal		7811238	351705	100.000	100.000

Supplementary Figure 68. HPLC Spectrum of racemic 4e.

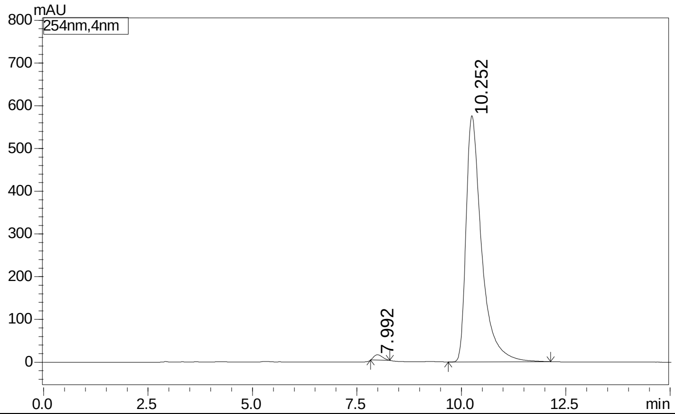

Peak	Ret. Time	Area	Height	Area%	Height%
1	7.992	179977	12454	1.192	2.116
2	10.252	14924771	576182	98.808	97.884
Tolal		15104748	588636	100.000	100.000

Supplementary Figure 69. HPLC Spectrum of 4e.

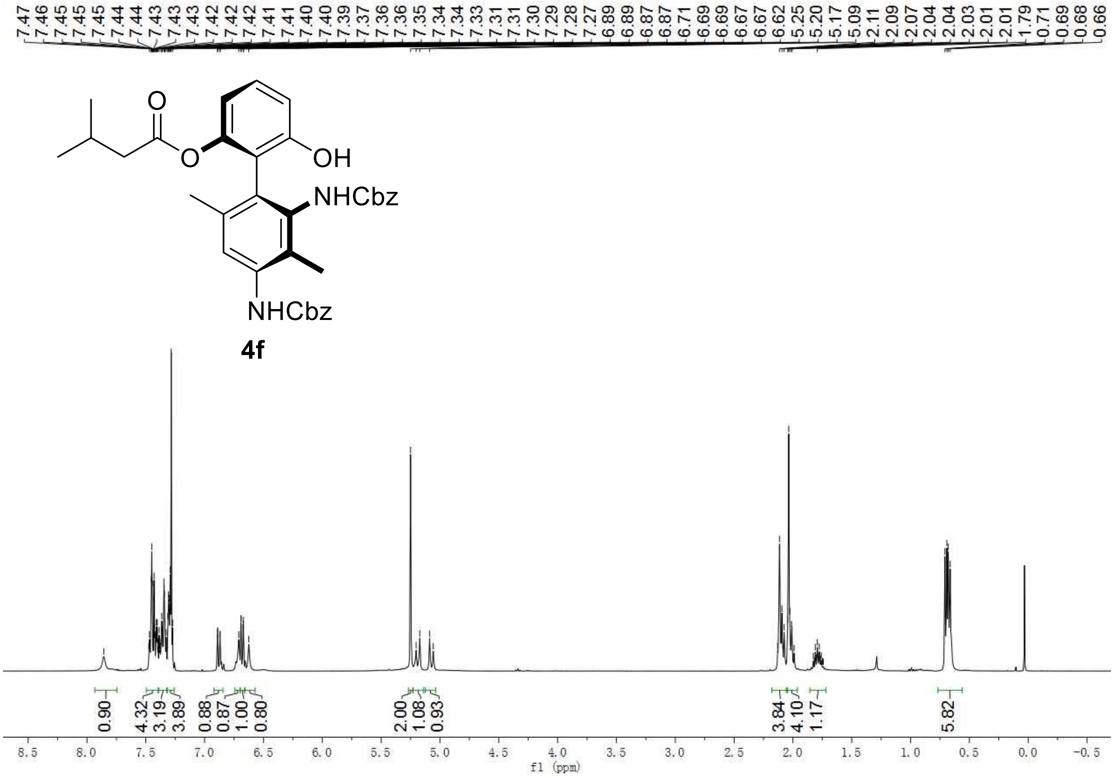

Supplementary Figure 70. ^{1}H NMR Spectrum of 4f.

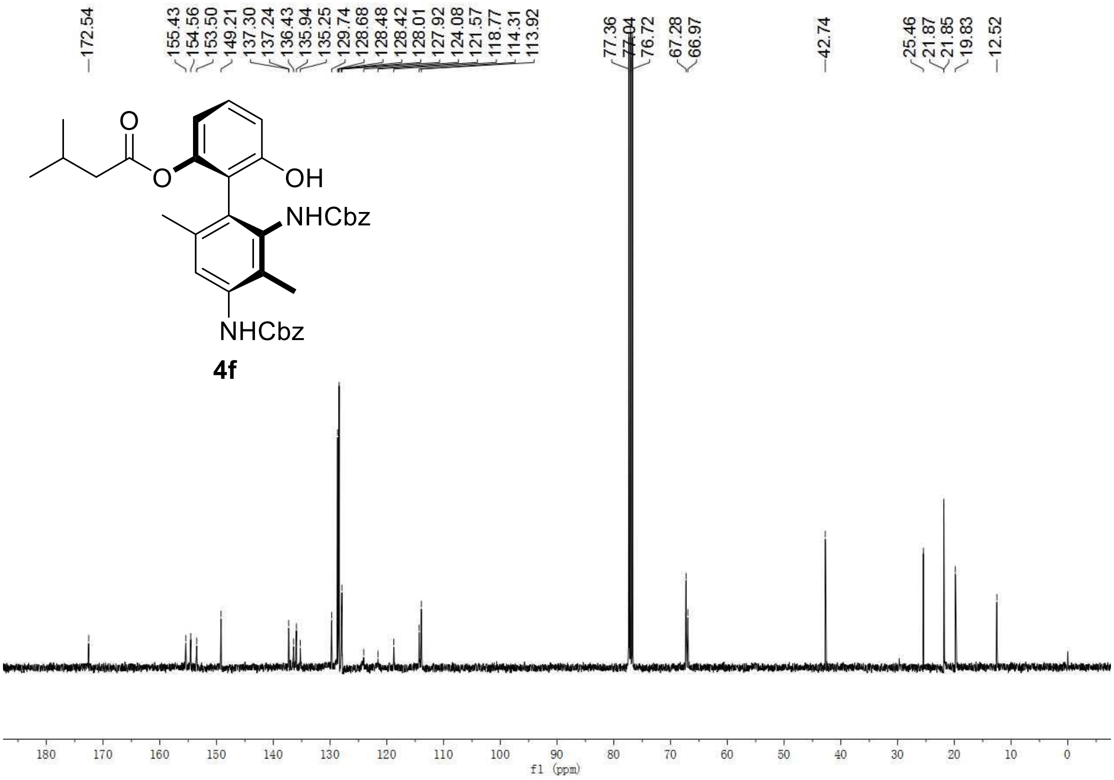

Supplementary Figure 71. ^{13}C NMR Spectrum of 4f.

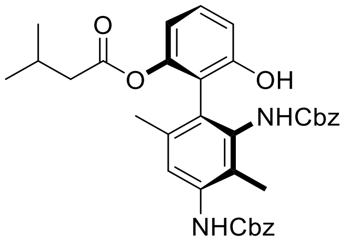

4f

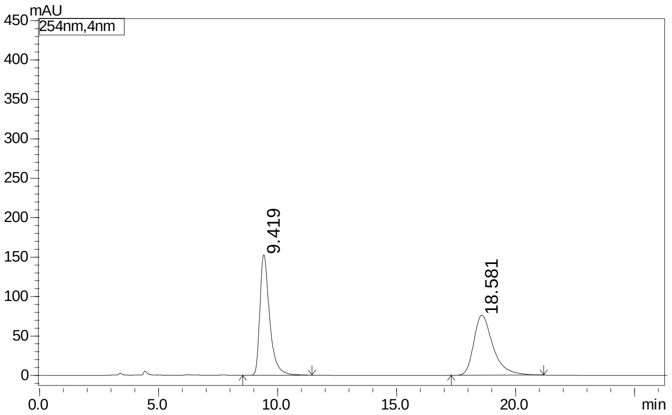

Peak	Ret. Time	Area	Height	Area%	Height%
1	9.419	4328677	152608	50.331	66.712
2	18.581	4271703	76149	49.669	33.288
Tolal		8600380	228757	100.000	100.000

Supplementary Figure 72. HPLC Spectrum of racemic 4f.

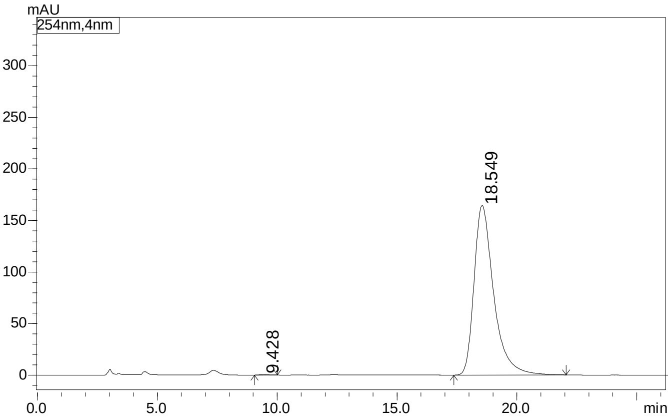

Peak	Ret. Time	Area	Height	Area%	Height%
1	9.428	13148	507	0.140	0.307
2	18.549	9369328	164503	99.860	99.693
Tolal		9382476	165010	100.000	100.000

Supplementary Figure 73. HPLC Spectrum of 4f.

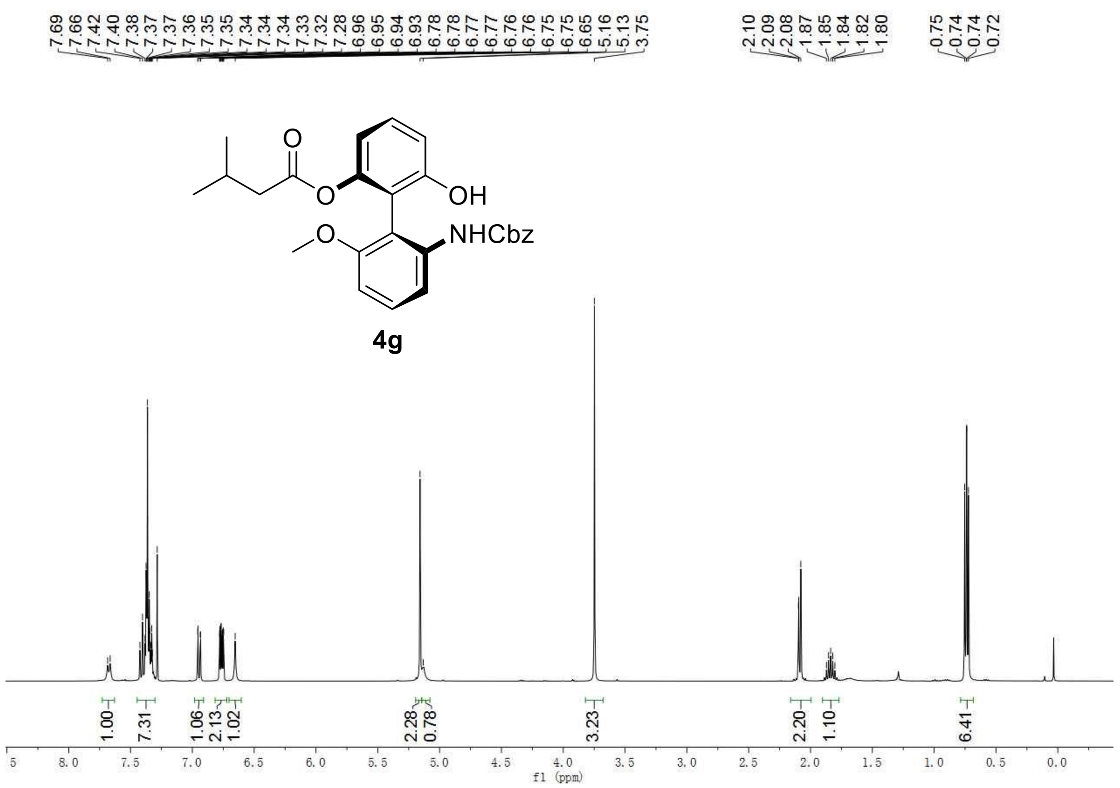

Supplementary Figure 74. ^{1}H NMR Spectrum of 4g.

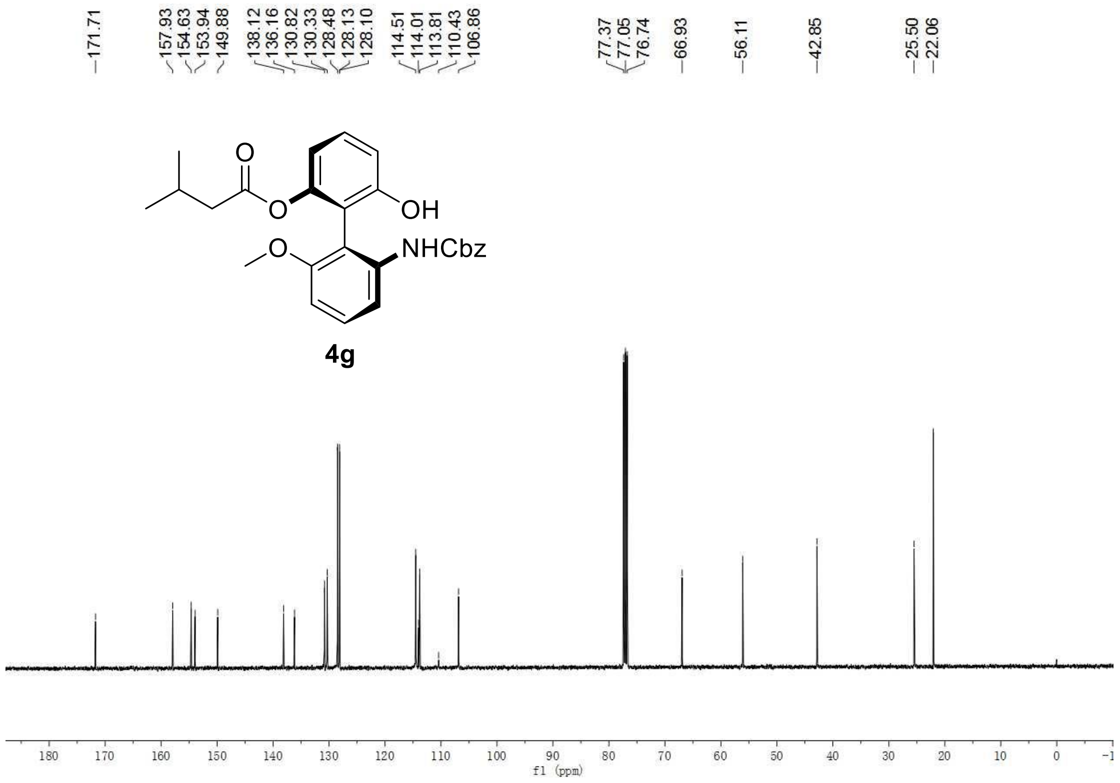

Supplementary Figure 75. ^{13}C NMR Spectrum of 4g.

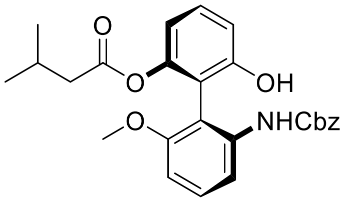

4g

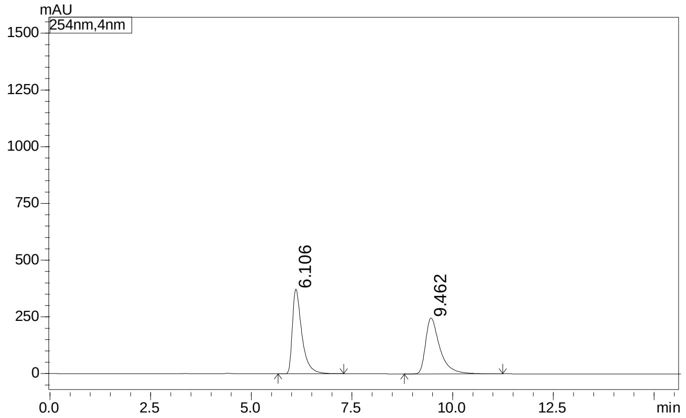

Peak	Ret. Time	Area	Height	Area%	Height%
1	6.106	6110953	373239	50.314	60.259
2	9.462	6034706	246150	49.686	39.741
Tolal		12145659	619390	100.000	100.000

Supplementary Figure 76. HPLC Spectrum of racemic 4g.

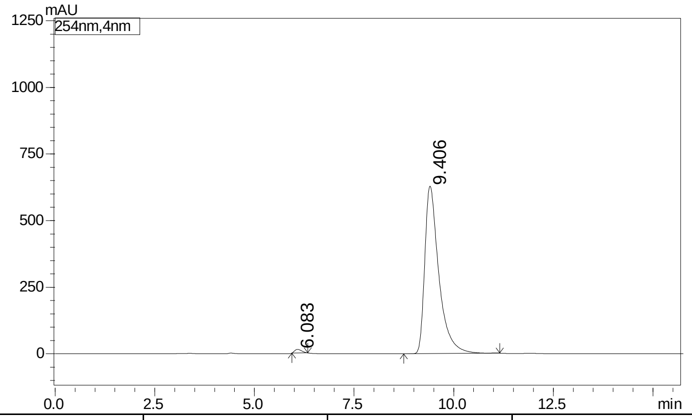

Peak	Ret. Time	Area	Height	Area%	Height%
1	6.083	165944	13660	1.065	2.125
2	9.406	15422869	629251	98.935	97.875
Tolal		15588813	642911	100.000	100.000

Supplementary Figure 77. HPLC Spectrum of 4g.

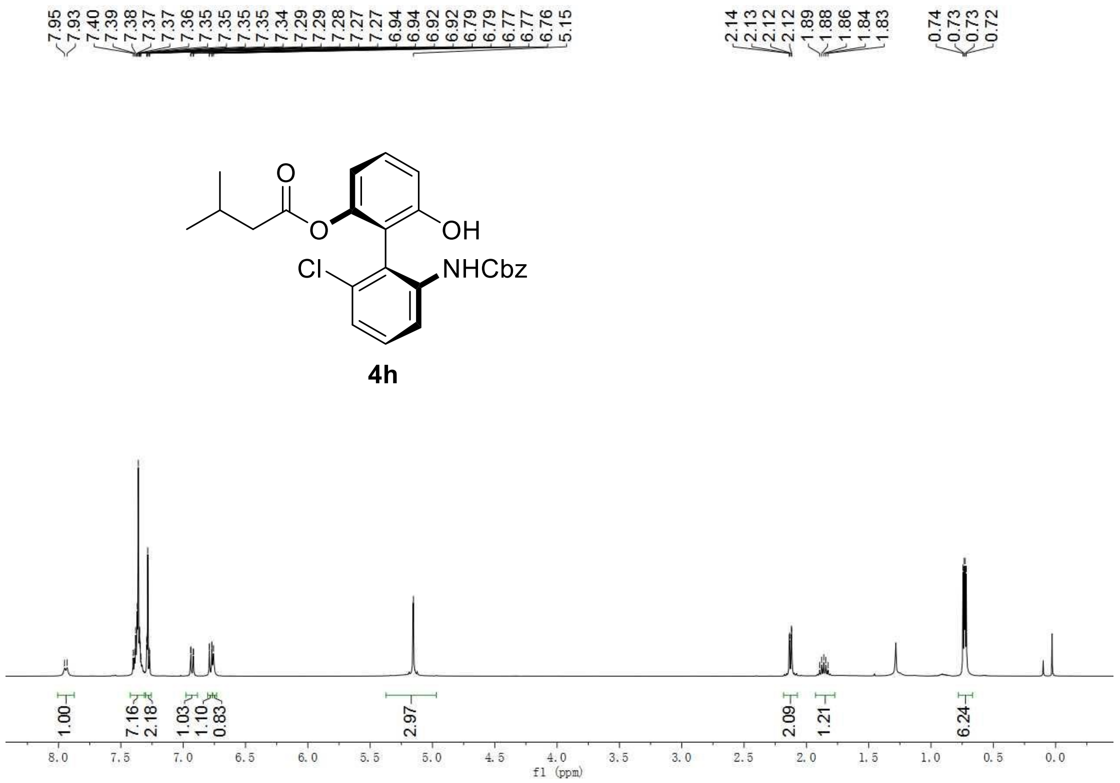

Supplementary Figure 78. ^{1}H NMR Spectrum of 4h.

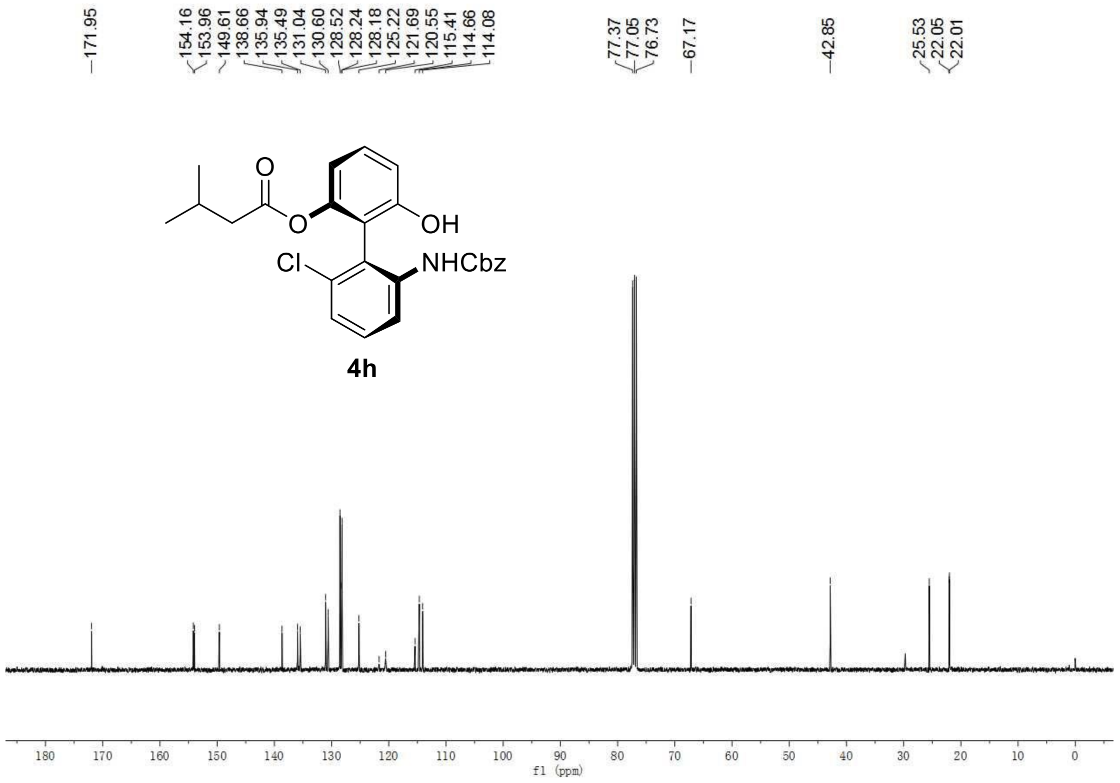

Supplementary Figure 79. ^{13}C NMR Spectrum of 4h.

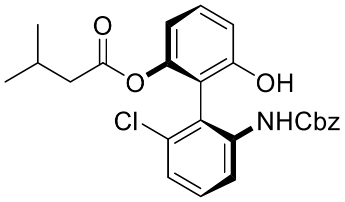

4h

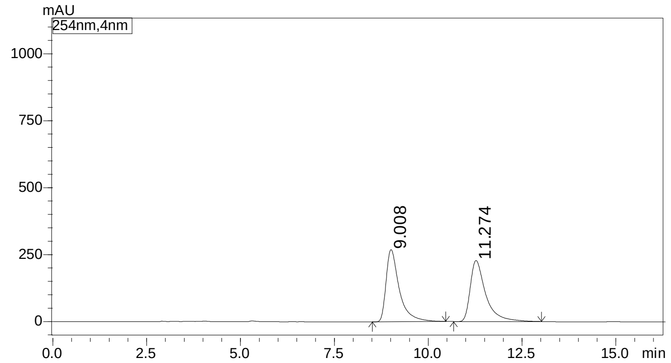

Peak	Ret. Time	Area	Height	Area%	Height%
1	9.008	6588178	269405	50.170	54.156
2	11.274	6543554	228053	49.830	45.844
Tolal		13131733	497458	100.000	100.000

Supplementary Figure 80. HPLC Spectrum of racemic 4h.

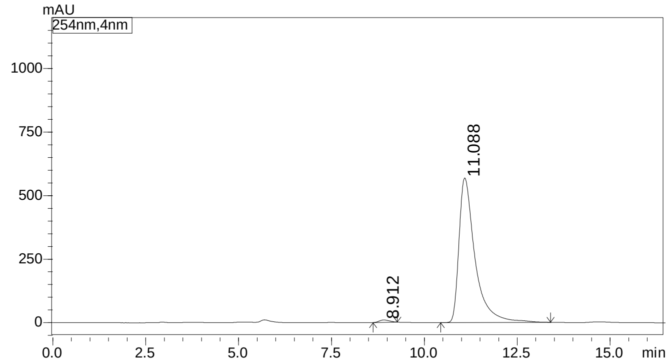

Peak	Ret. Time	Area	Height	Area%	Height%
1	8.912	180529	9407	1.045	1.629
2	11.088	17089923	568209	98.955	98.371
Tolal		17270452	577616	100.000	100.000

Supplementary Figure 81. HPLC Spectrum of 4h.

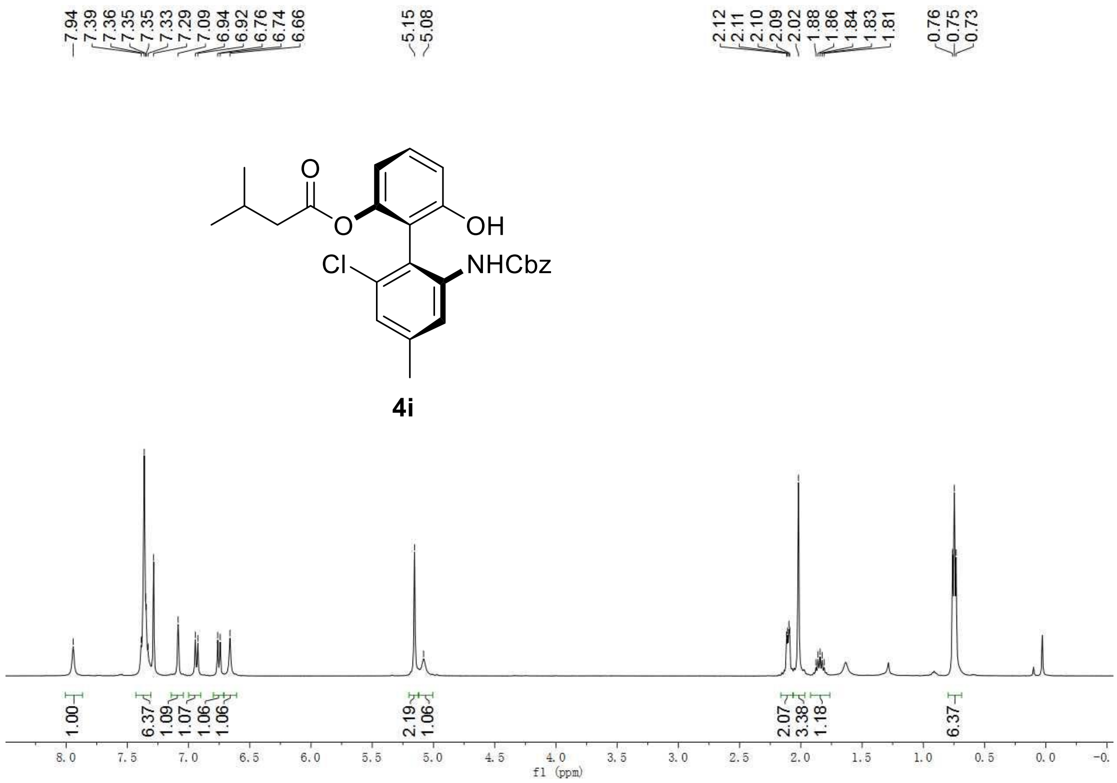

Supplementary Figure 82. ^{1}H NMR Spectrum of 4i.

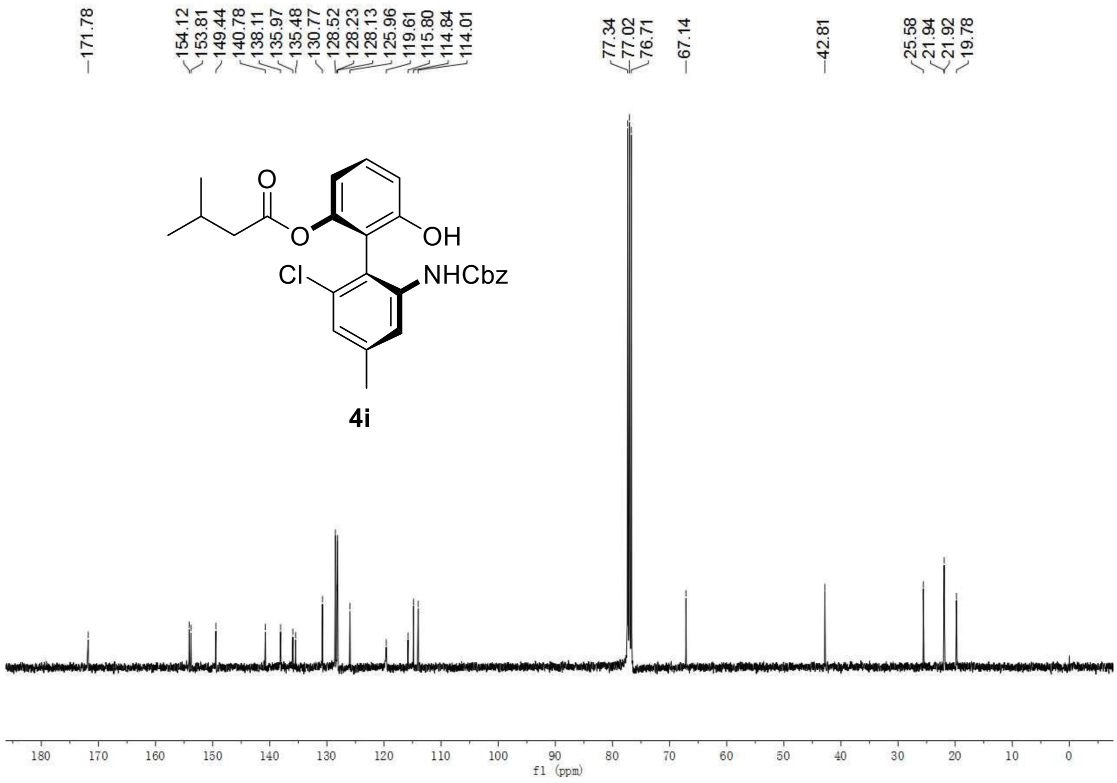

Supplementary Figure 83. ^{13}C NMR Spectrum of 4i.

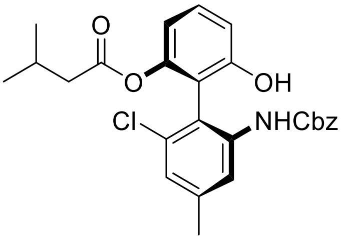

4i

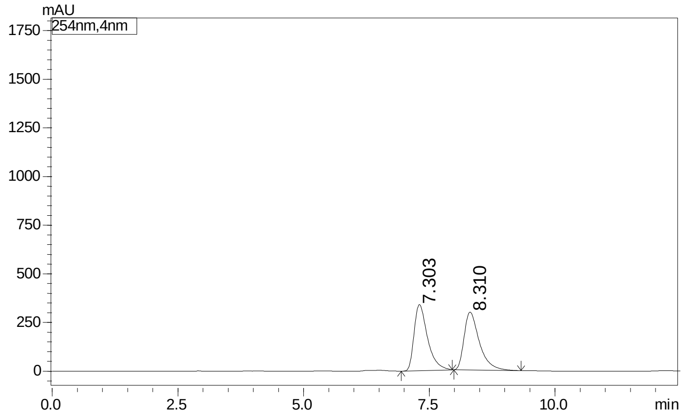

Peak	Ret. Time	Area	Height	Area%	Height%
1	7.303	6294841	339453	50.057	53.313
2	8.310	6280391	297268	49.943	46.687
Tolal		12575232	636722	100.000	100.000

Supplementary Figure 84. HPLC Spectrum of racemic 4i.

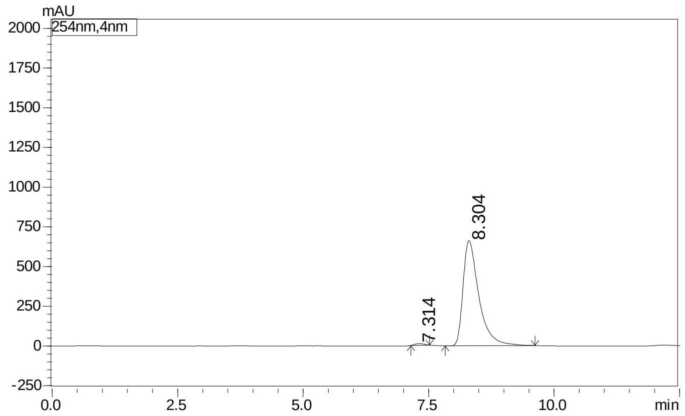

Peak	Ret. Time	Area	Height	Area%	Height%
1	7.314	141781	11076	0.969	1.647
2	8.304	14496845	661237	99.031	98.353
Tolal		14638626	672313	100.000	100.000

Supplementary Figure 85. HPLC Spectrum of 4i.

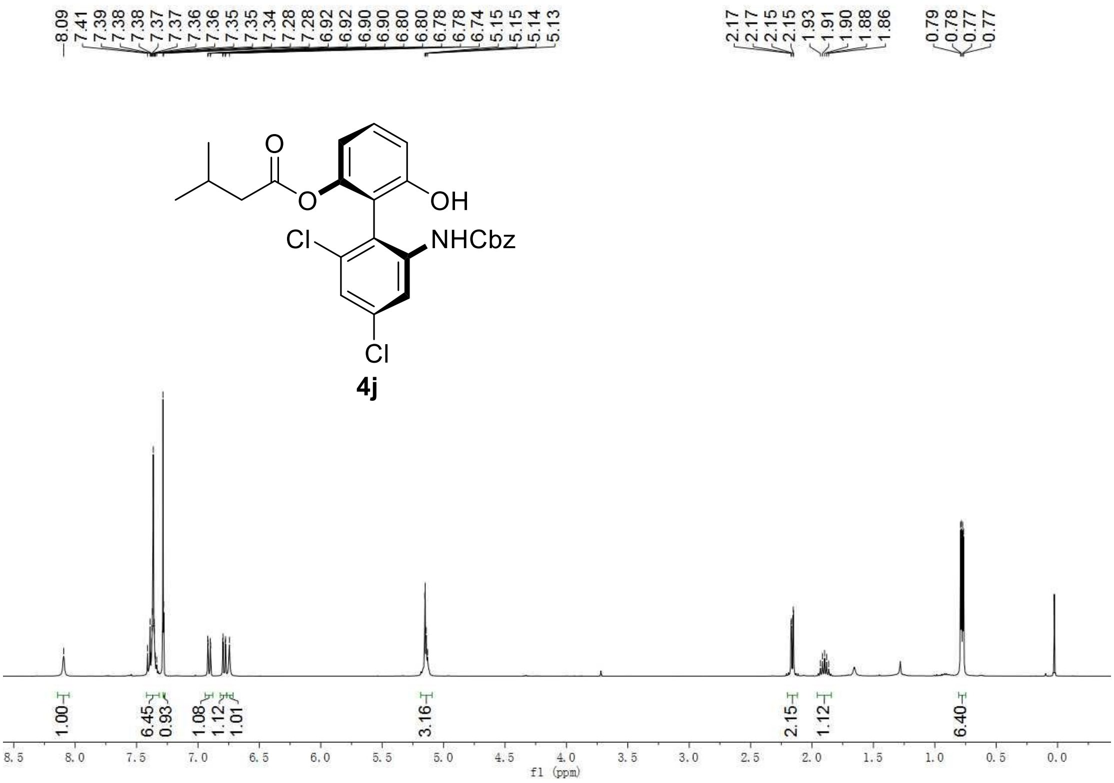

Supplementary Figure 86. ^{1}H NMR Spectrum of 4j.

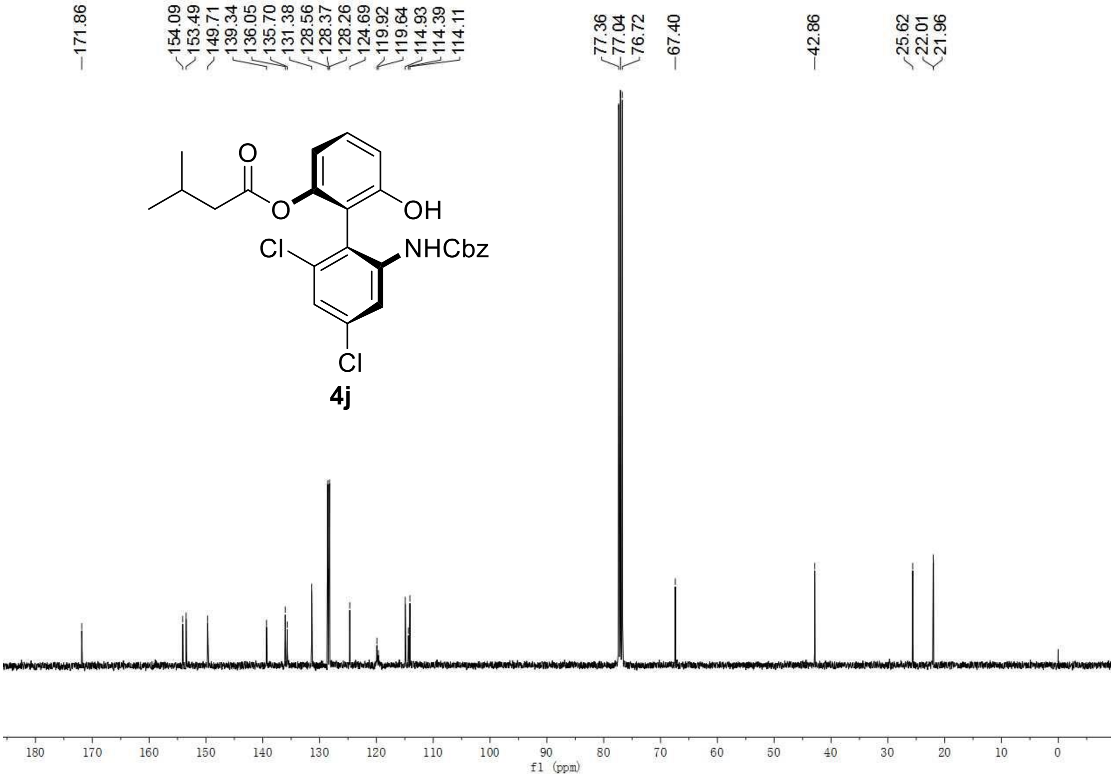

Supplementary Figure 87. ^{13}C NMR Spectrum of 4j.

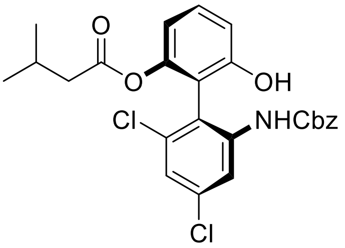

4j

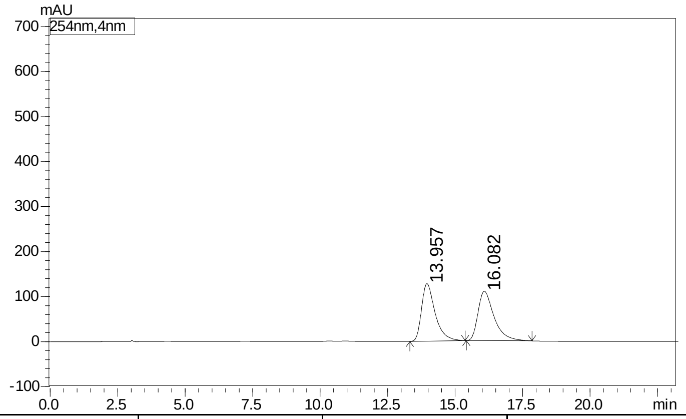

Peak	Ret. Time	Area	Height	Area%	Height%
1	13.957	4478759	127607	49.591	53.734
2	16.082	4552639	109872	50.409	46.266
Tolal		9031397	237479	100.000	100.000

Supplementary Figure 88. HPLC Spectrum of racemic 4j.

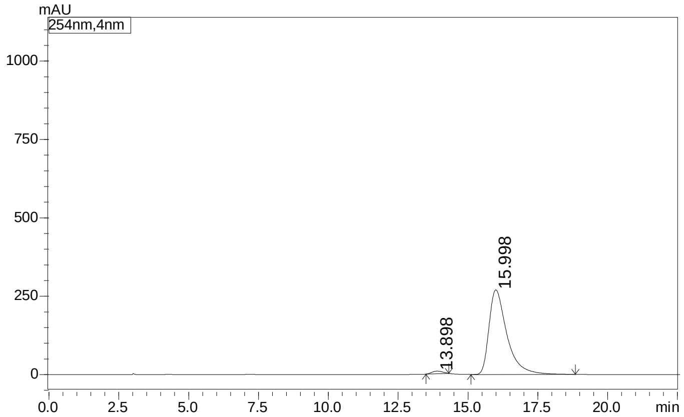

Peak	Ret. Time	Area	Height	Area%	Height%
1	13.898	228581	8460	1.862	3.038
2	15.998	12049105	270014	98.138	96.962
Tolal		12277685	278474	100.000	100.000

Supplementary Figure 89. HPLC Spectrum of 4j.

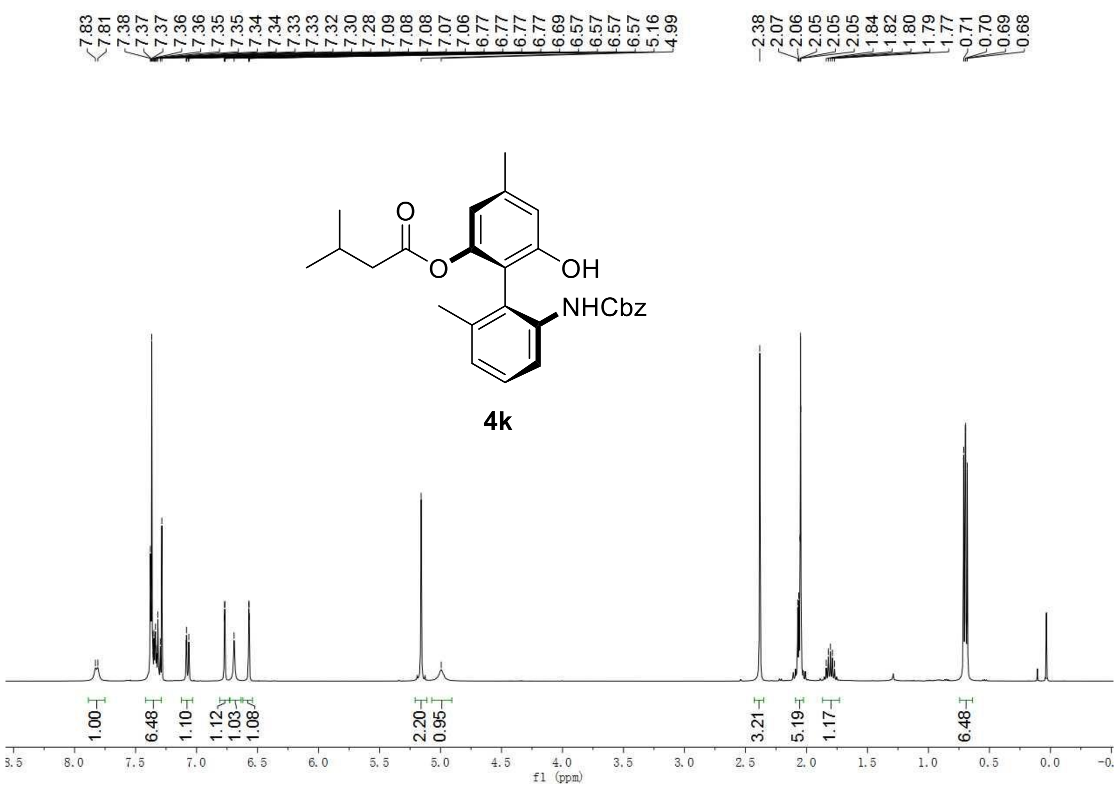

Supplementary Figure 90. ^{1}H NMR Spectrum of 4k.

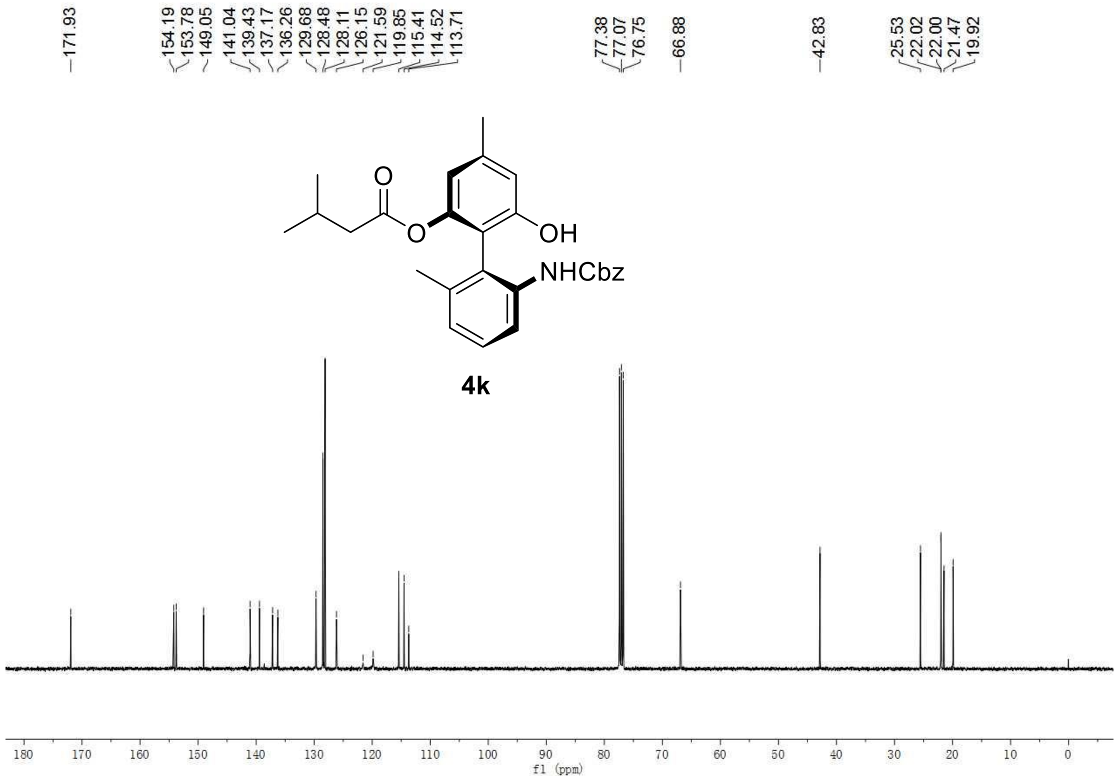

Supplementary Figure 91. ^{13}C NMR Spectrum of 4k.

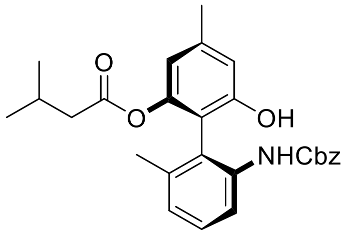

4k

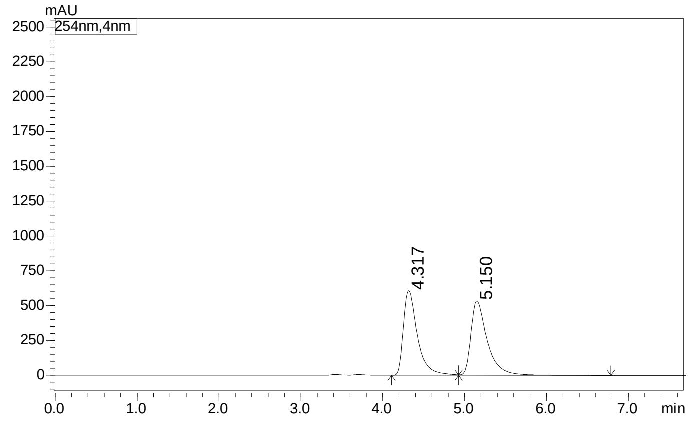

Peak	Ret. Time	Area	Height	Area%	Height%
1	4.317	7244224	607652	49.380	53.182
2	5.150	7426235	534944	50.620	46.818
Tolal		14670460	1142596	100.000	100.000

Supplementary Figure 92. HPLC Spectrum of racemic 4k.

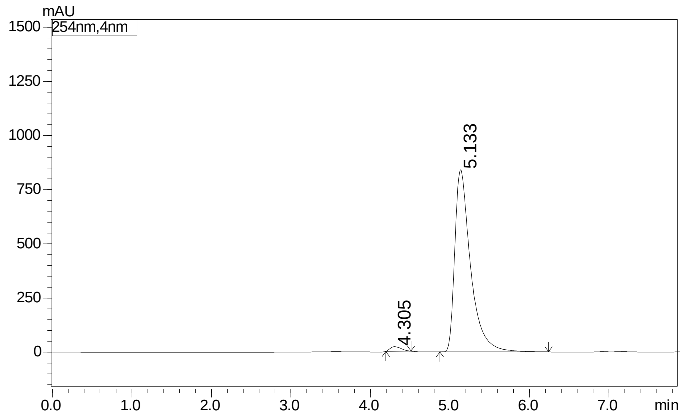

Peak	Ret. Time	Area	Height	Area%	Height%
1	4.305	209781	21789	1.776	2.526
2	5.133	11601994	840744	98.224	97.474
Tolal		11811776	862533	100.000	100.000

Supplementary Figure 93. HPLC Spectrum of 4k.

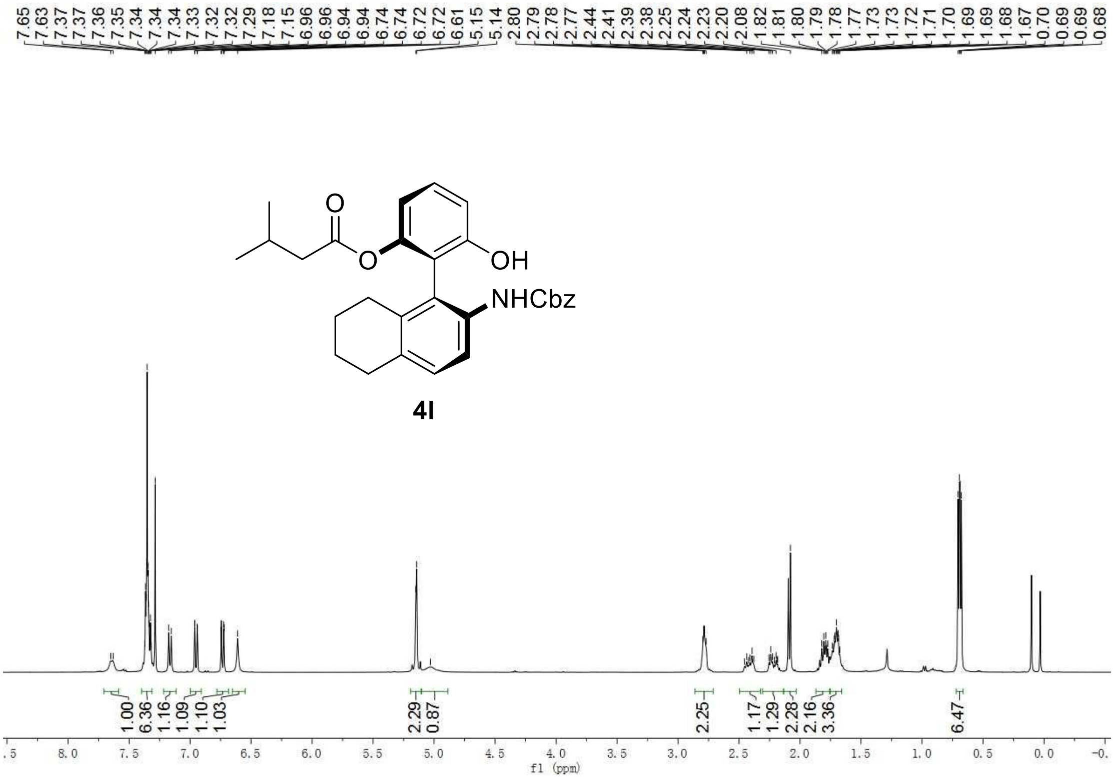

Supplementary Figure 94. ^{1}H NMR Spectrum of 4l.

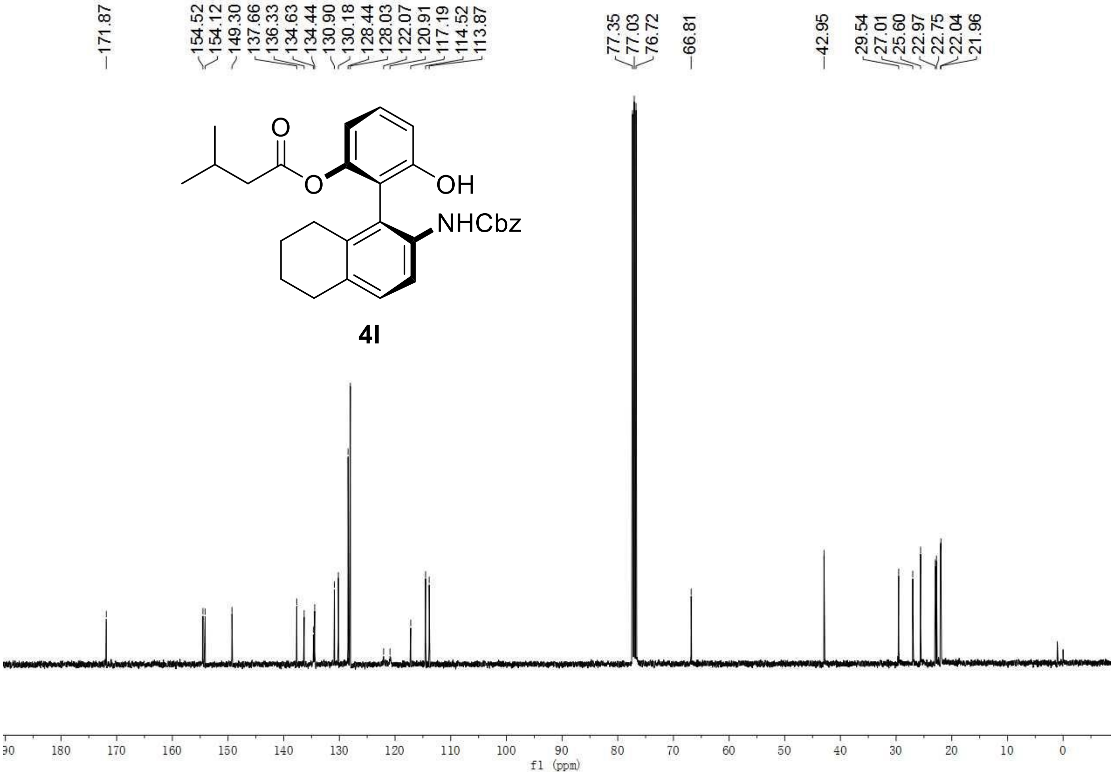

Supplementary Figure 95. ^{13}C NMR Spectrum of 4l.

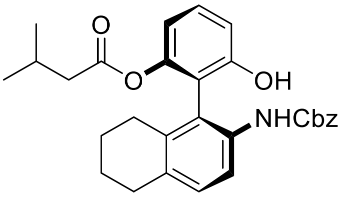

4l

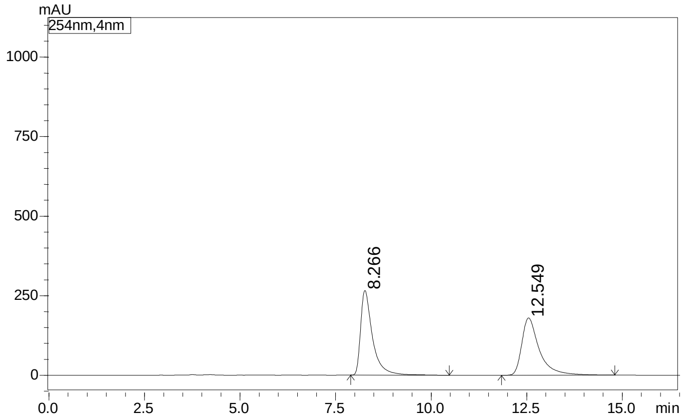

Peak	Ret. Time	Area	Height	Area%	Height%
1	8.266	5775633	266344	50.133	59.670
2	12.549	5744982	180014	49.867	40.330
Tolal		11520615	446358	100.000	100.000

Supplementary Figure 96. HPLC Spectrum of racemic 4l.

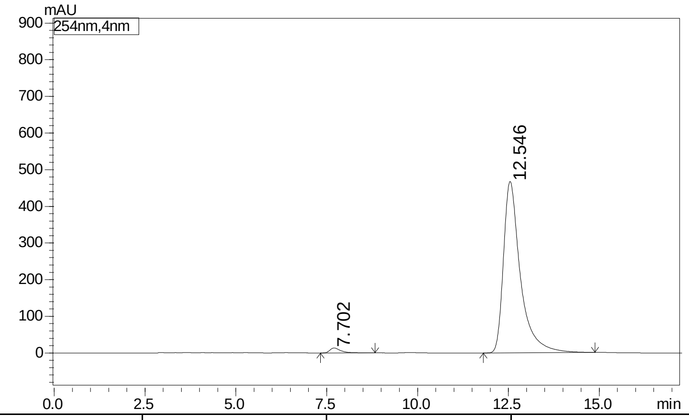

Peak	Ret. Time	Area	Height	Area%	Height%
1	7.702	288656	13692	1.870	2.844
2	12.546	15143935	467736	98.130	97.156
Tolal		15432591	481428	100.000	100.000

Supplementary Figure 97. HPLC Spectrum of 4l.

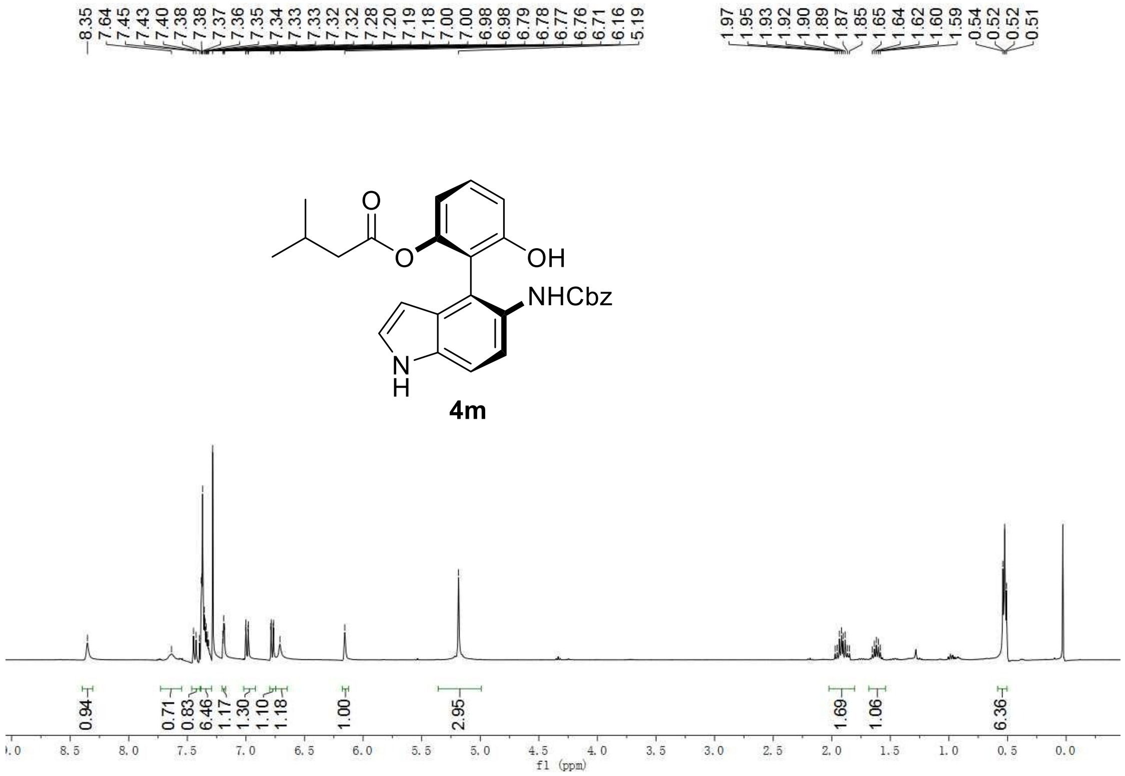

Supplementary Figure 98. ^{1}H NMR Spectrum of 4m.

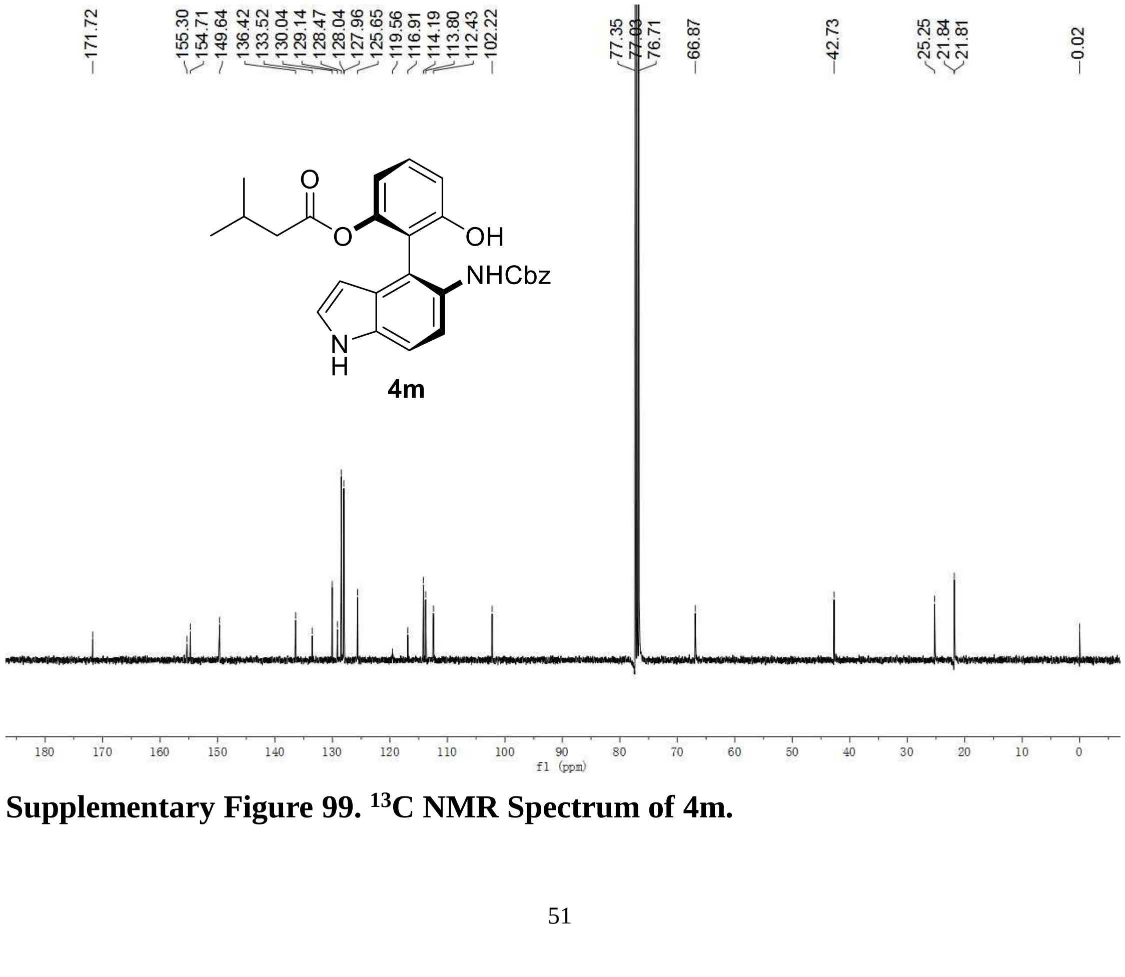

Supplementary Figure 99. ^{13}C NMR Spectrum of 4m.

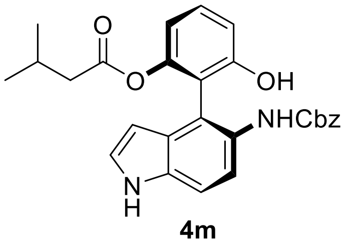

4m

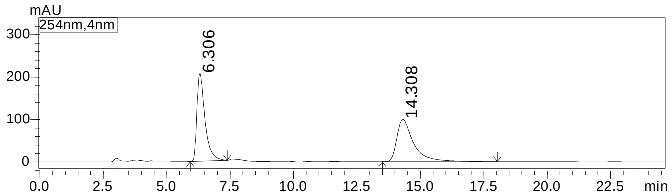

Peak	Ret. Time	Area	Height	Area%	Height%
1	6.306	4585973	206629	49.568	67.346
2	14.308	4665849	100187	50.432	32.654
Tolal		9251822	306816	100.000	100.000

Supplementary Figure 100. HPLC Spectrum of racemic 4m.

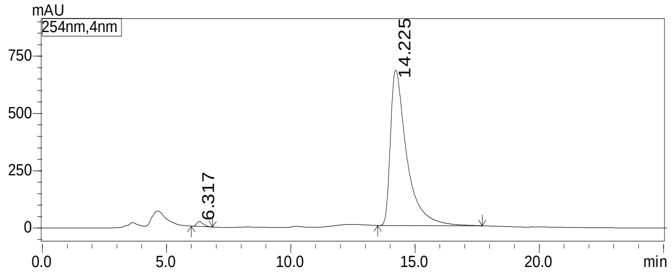

Peak	Ret. Time	Area	Height	Area%	Height%
1	6.317	395851	21525	1.261	3.073
2	14.225	31002693	679017	98.739	96.927
Tolal		31398544	700541	100.000	100.000

Supplementary Figure 101. HPLC Spectrum of 4m.

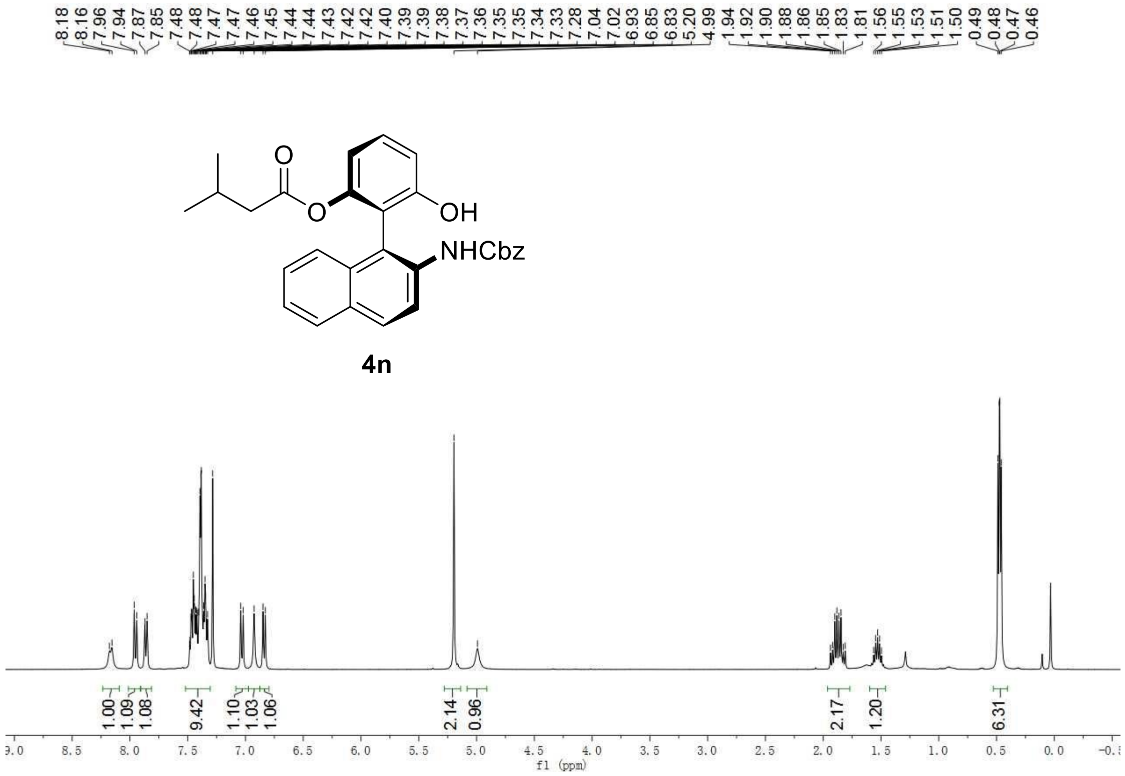

Supplementary Figure 102. ^{1}H NMR Spectrum of 4n.

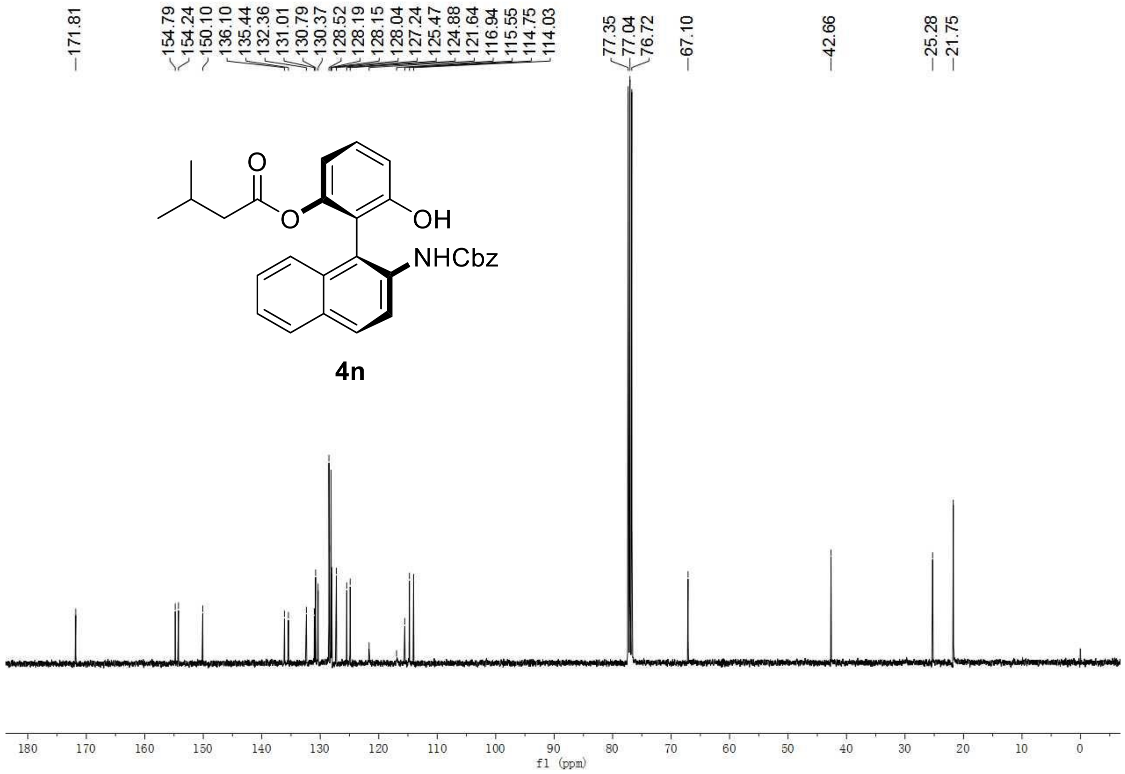

Supplementary Figure 103. ^{13}C NMR Spectrum of 4n.

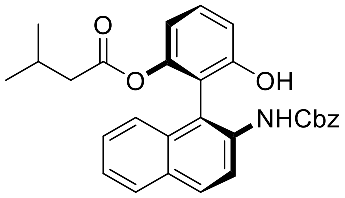

4n

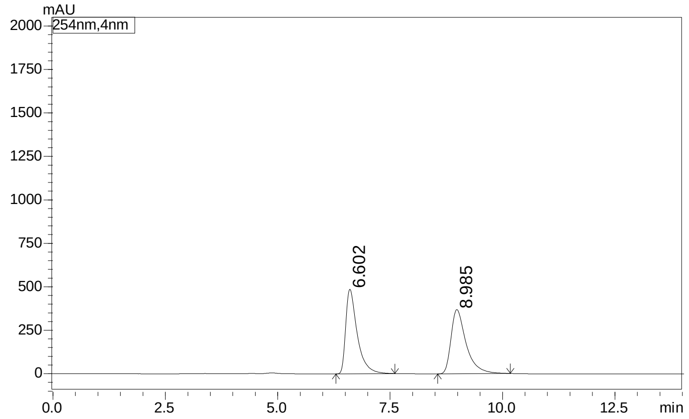

Peak	Ret. Time	Area	Height	Area%	Height%
1	6.602	8657697	486914	50.174	56.869
2	8.985	8597522	369290	49.826	43.131
Tolal		17255219	856204	100.000	100.000

Supplementary Figure 104. HPLC Spectrum of racemic 4n.

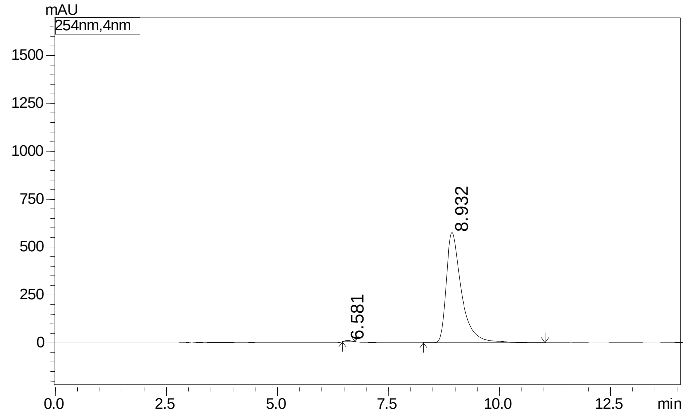

Peak	Ret. Time	Area	Height	Area%	Height%
1	6.581	68533	6782	0.496	1.165
2	8.932	13741913	575498	99.504	98.835
Tolal		13810446	582280	100.000	100.000

Supplementary Figure 105. HPLC Spectrum of 4n.

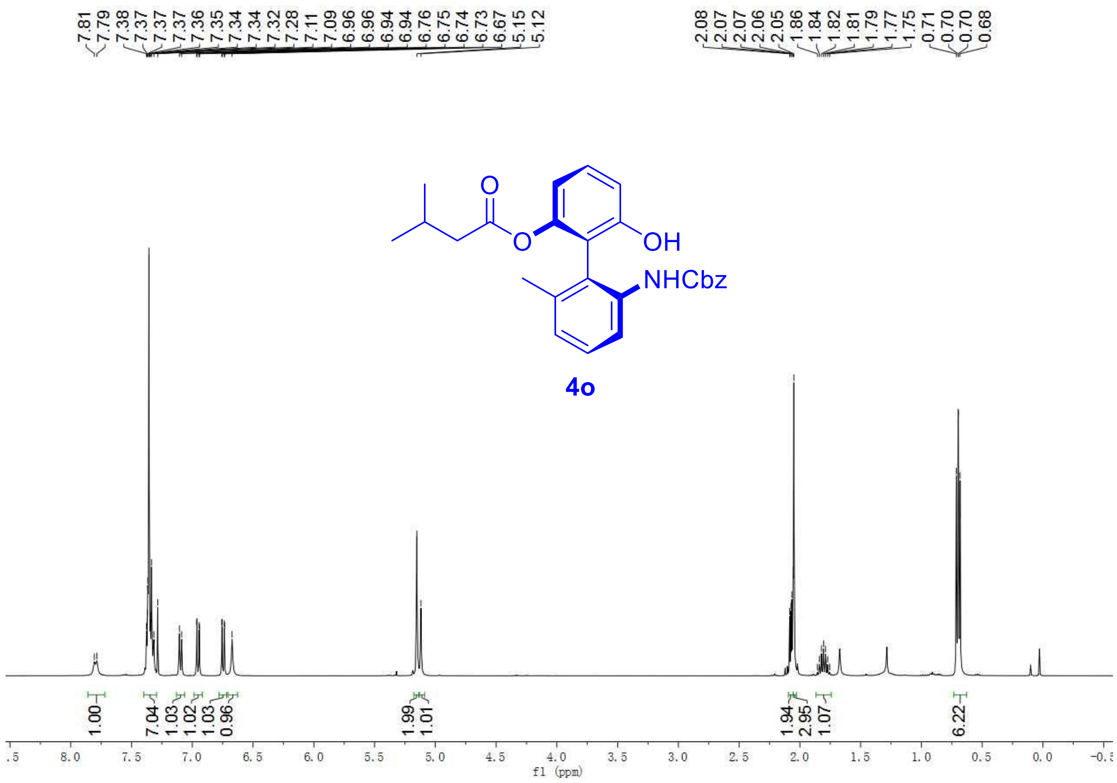

Supplementary Figure 106. ^{1}H NMR Spectrum of 4o.

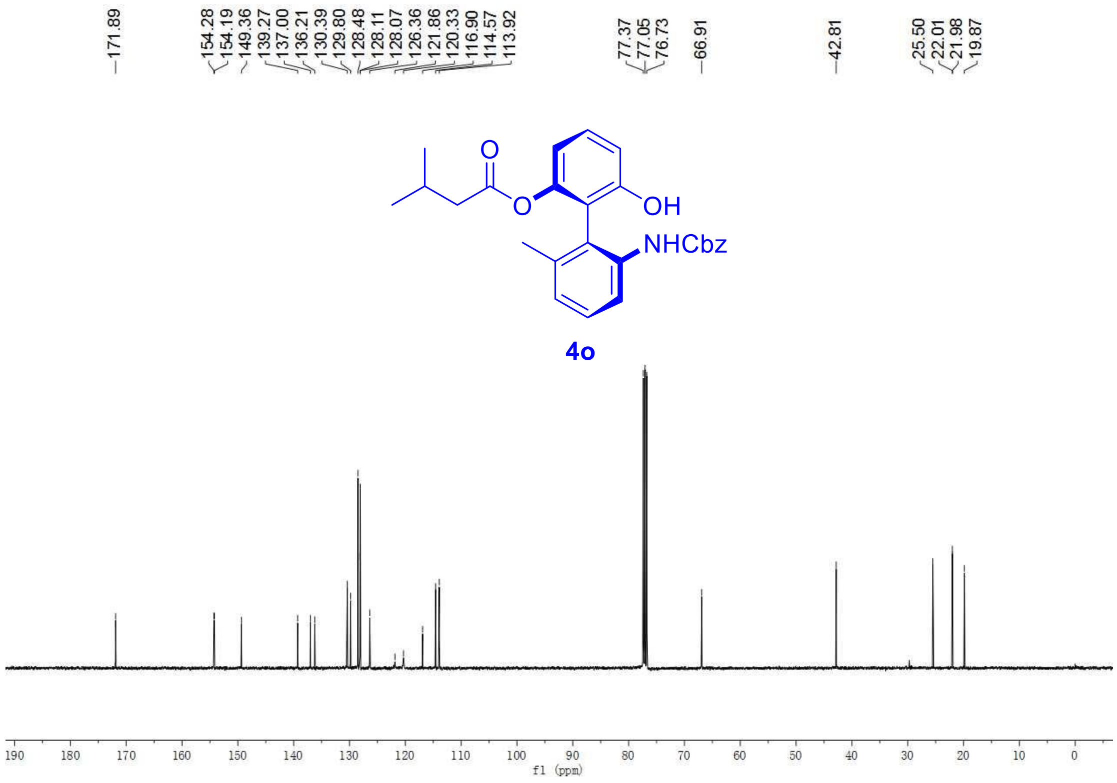

Supplementary Figure 107. ^{13}C NMR Spectrum of 4o.

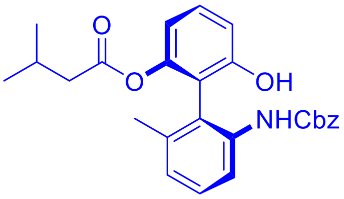

4o

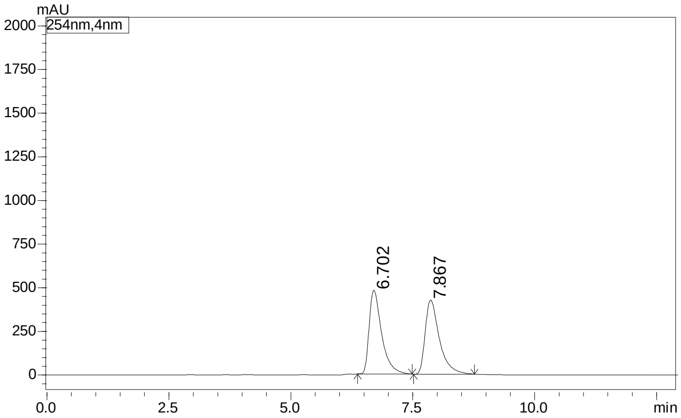

Peak	Ret. Time	Area	Height	Area%	Height%
1	6.702	8564833	480128	49.904	53.067
2	7.867	8597818	424628	50.096	46.933
Tolal		17162651	904755	100.000	100.000

Supplementary Figure 108. HPLC Spectrum of racemic 4o.

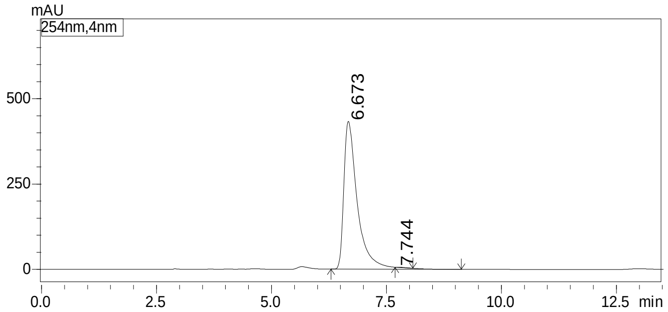

Peak	Ret. Time	Area	Height	Area%	Height%
1	6.673	8954670	433305	99.909	99.881
2	7.744	8153	514	0.091	0.119
Tolal		8962823	433819	100.000	100.000

Supplementary Figure 109. HPLC Spectrum of 4o.

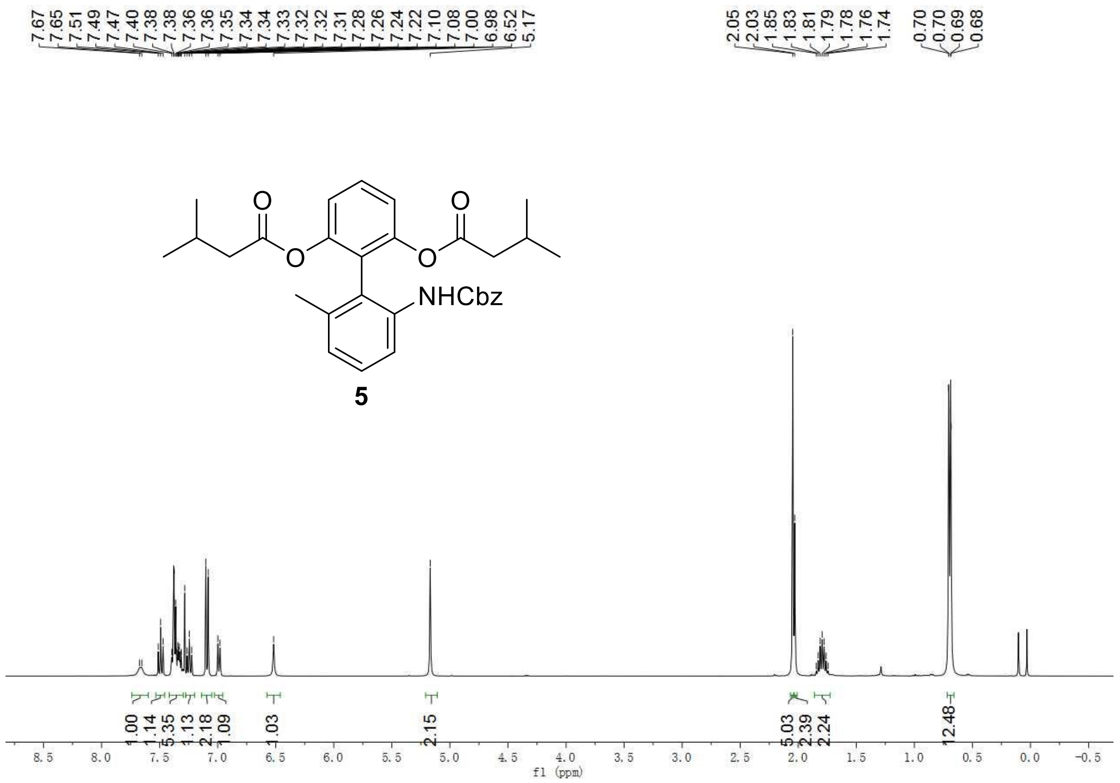

Supplementary Figure 110. ^{1}H NMR Spectrum of 5.

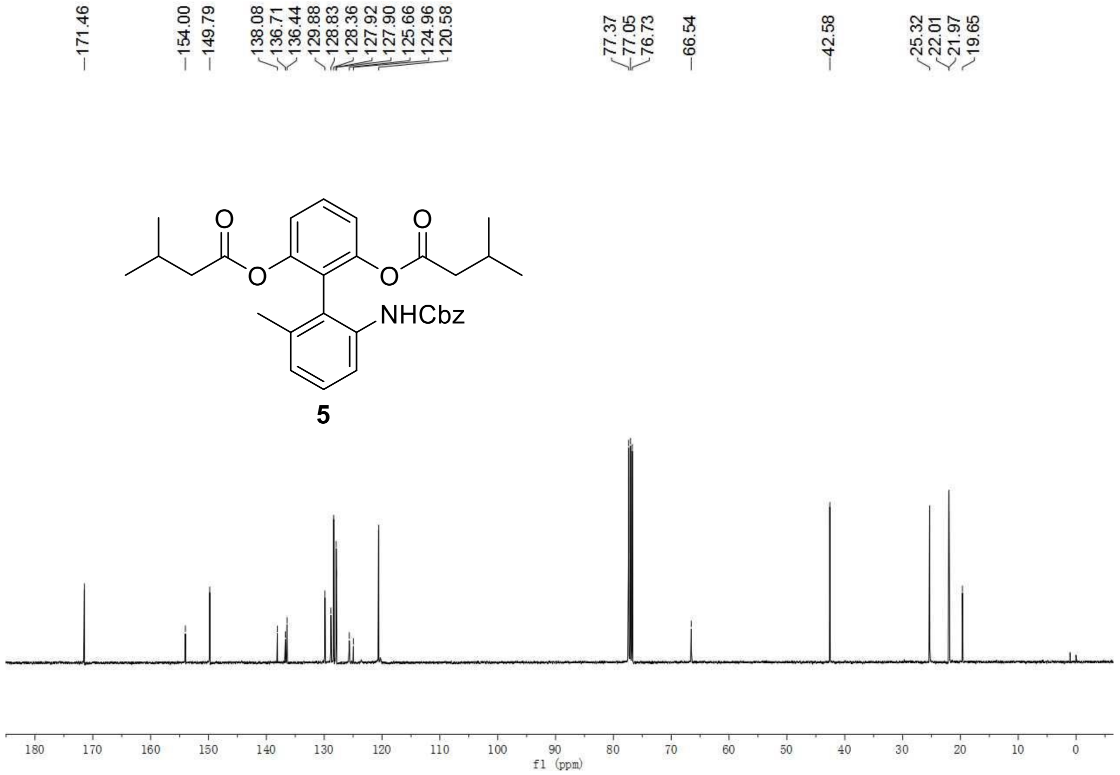

Supplementary Figure 111. ^{13}C NMR Spectrum of 5.

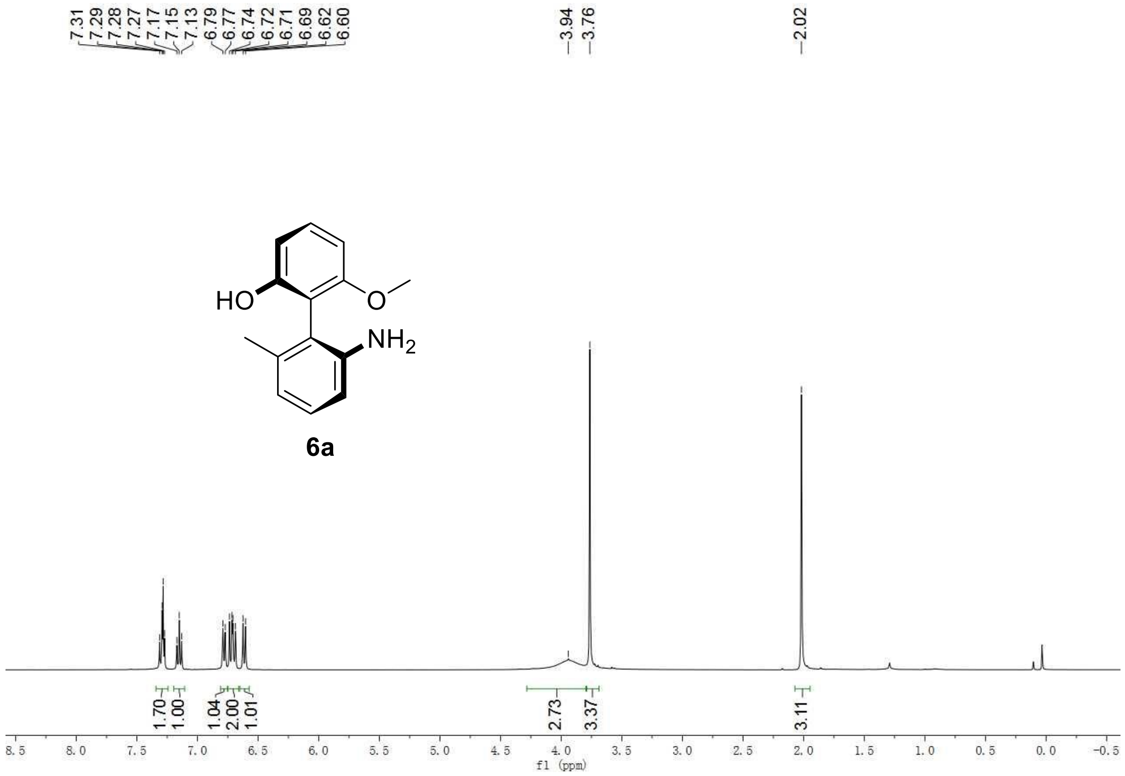

Supplementary Figure 112. ^{1}H NMR Spectrum of 6a.

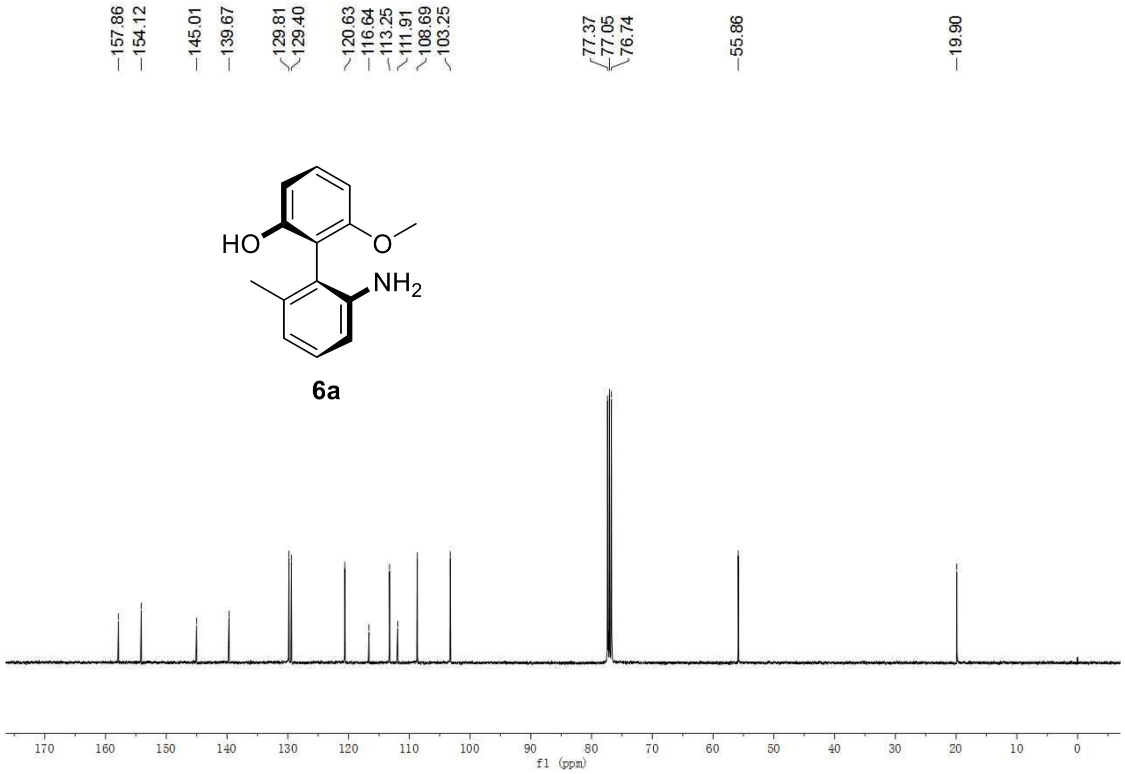

Supplementary Figure 113. ^{13}C NMR Spectrum of 6a.

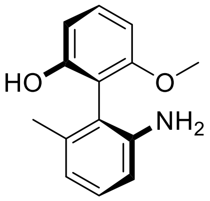

6a

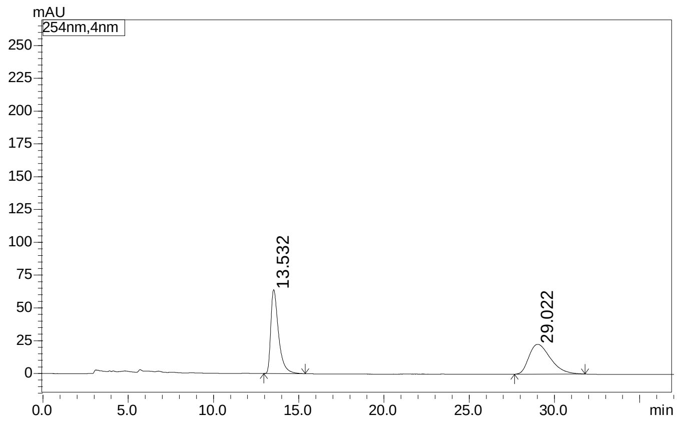

Peak	Ret. Time	Area	Height	Area%	Height%
1	13.532	2017140	63946	50.314	73.802
2	29.022	1991957	22699	49.686	26.198
Tolal		4009097	86645	100.000	100.000

Supplementary Figure 114. HPLC Spectrum of racemic 6a.

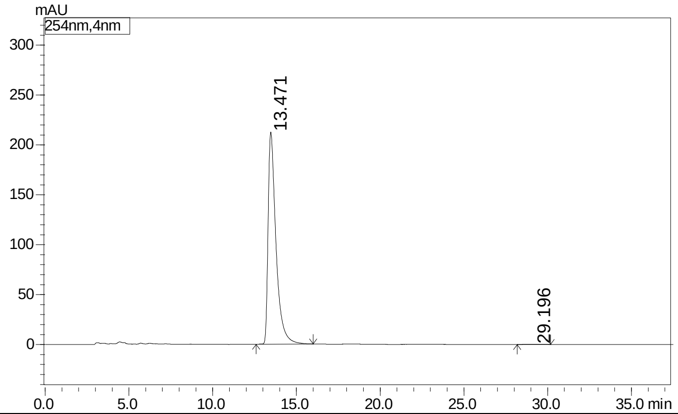

Peak	Ret. Time	Area	Height	Area%	Height%
1	13.471	6945081	212559	99.841	99.921
2	29.196	11063	168	0.159	0.079
Tolal		6956144	212726	100.000	100.000

Supplementary Figure 115. HPLC Spectrum of 6a.

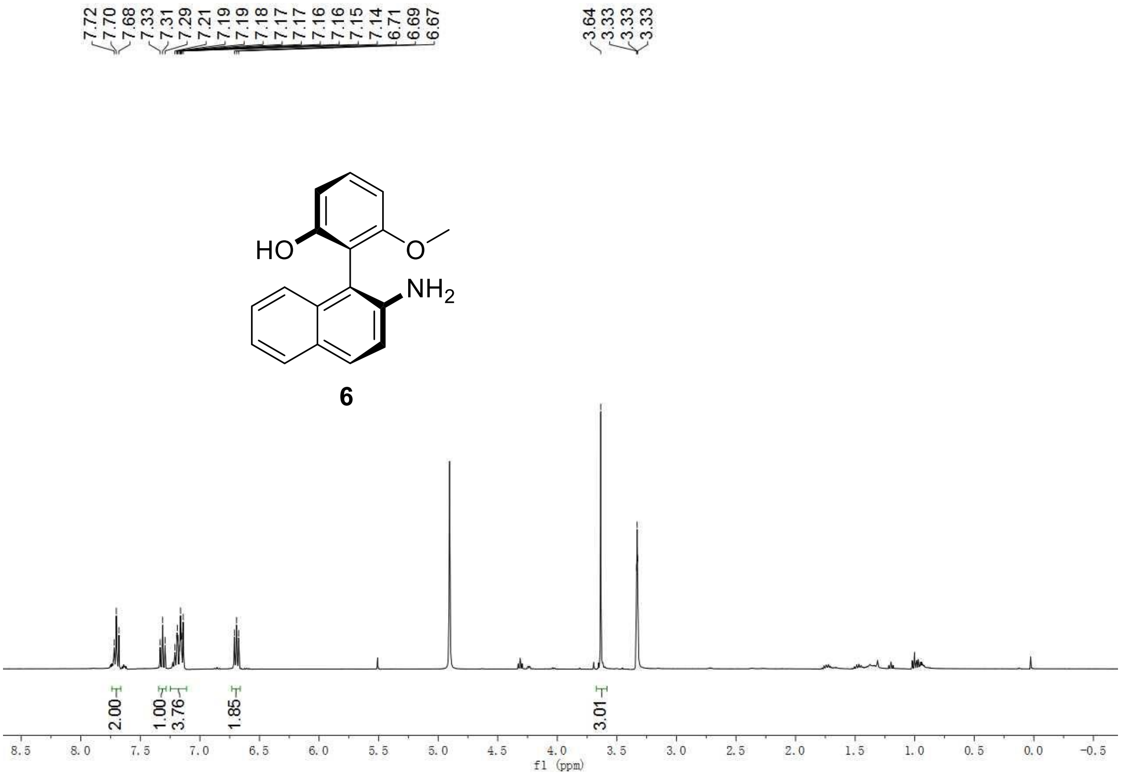

Supplementary Figure 116. ^{1}H NMR Spectrum of 6.

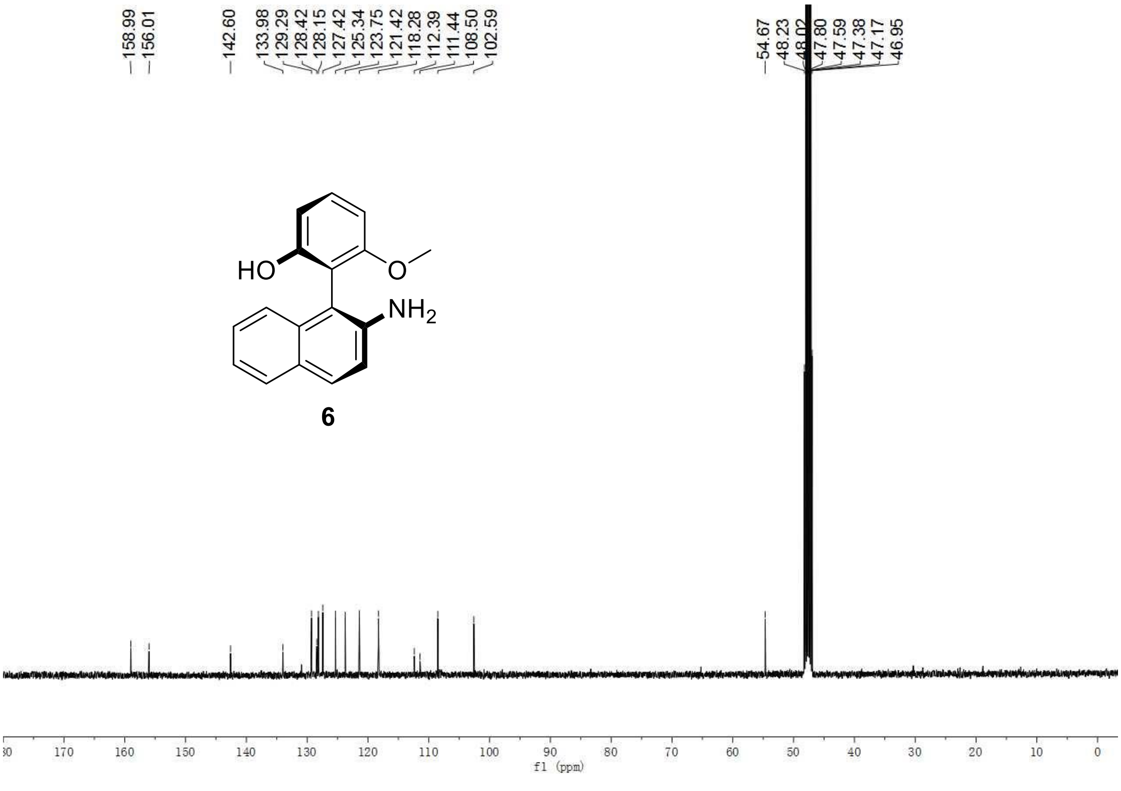

Supplementary Figure 117. ^{13}C NMR Spectrum of 6.

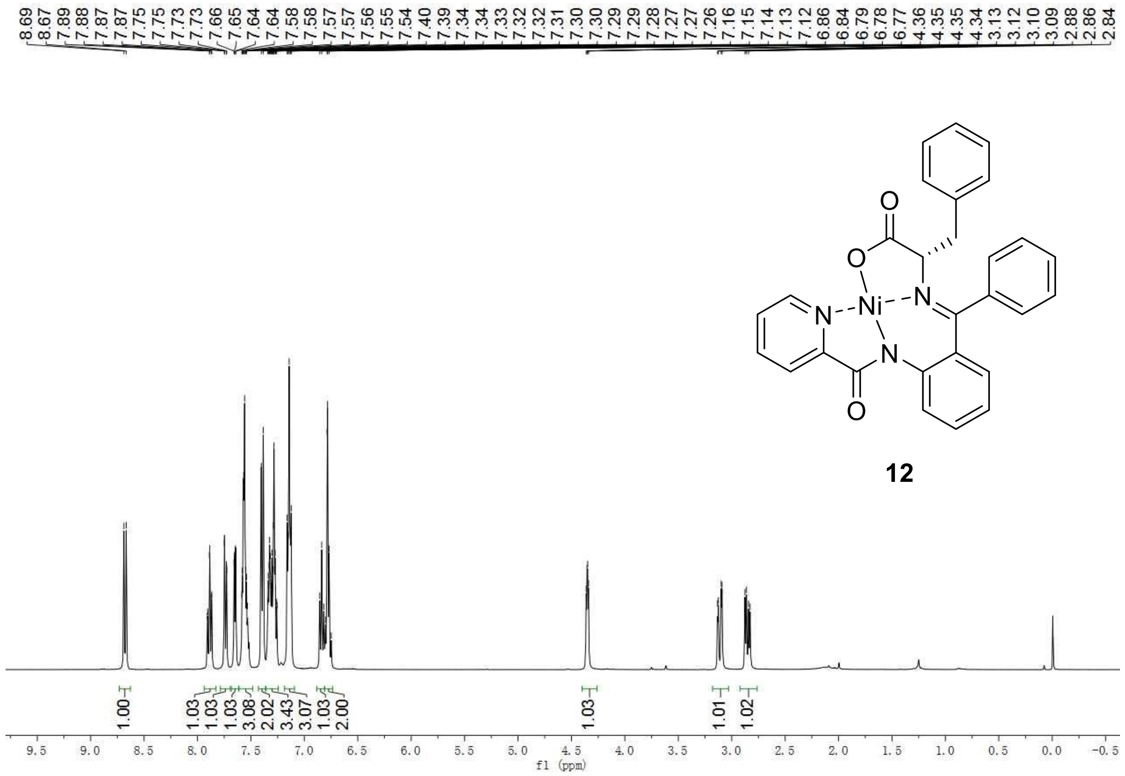

Supplementary Figure 118. ¹H NMR Spectrum of 12.

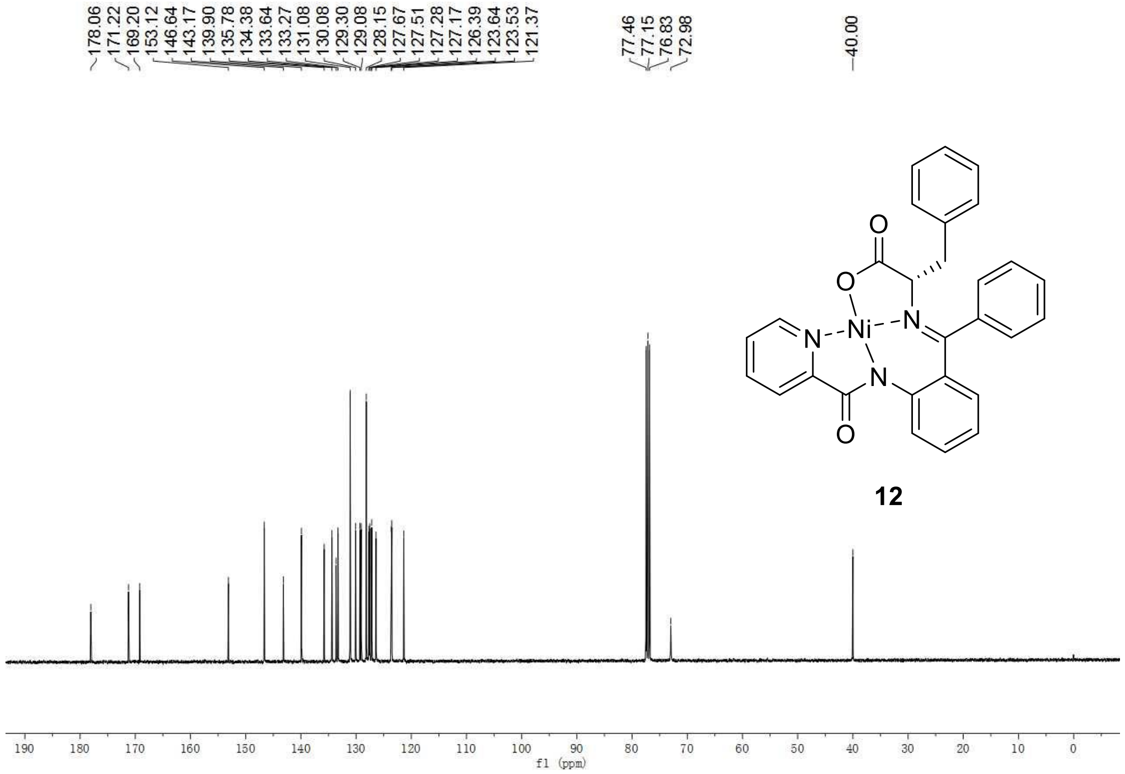

Supplementary Figure 119. ¹³C NMR Spectrum of 12.

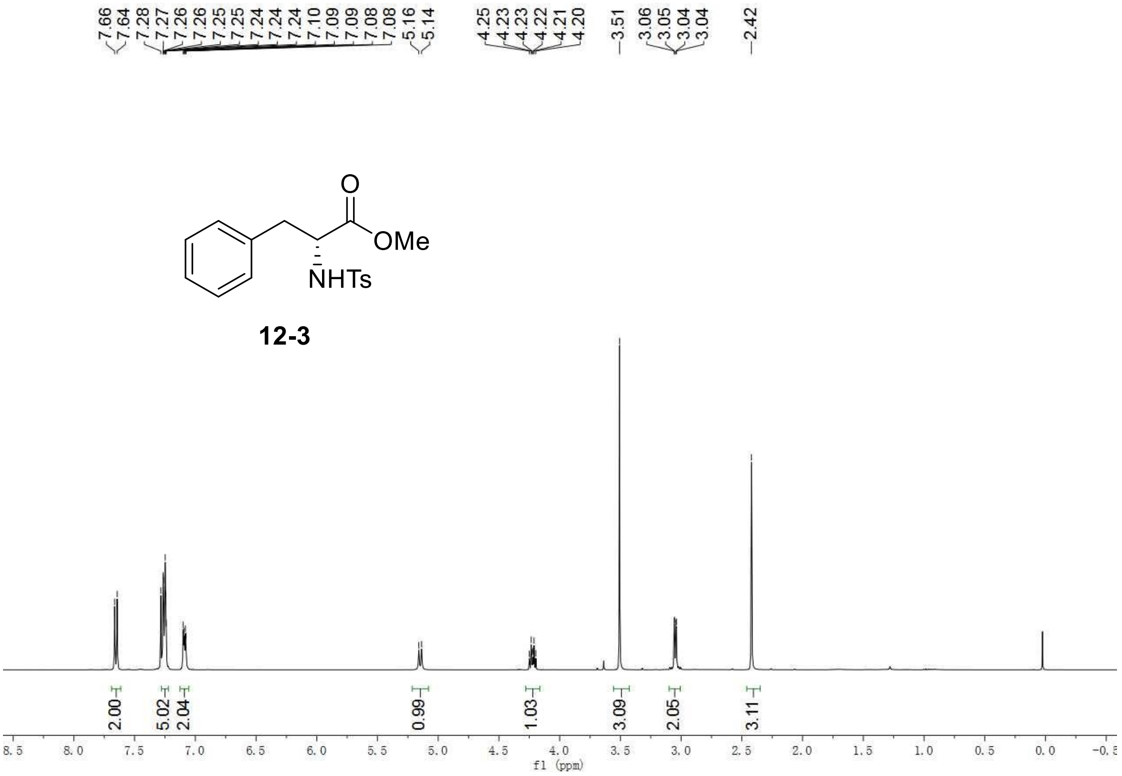

Supplementary Figure 120. ^{1}H NMR Spectrum of 12-3.

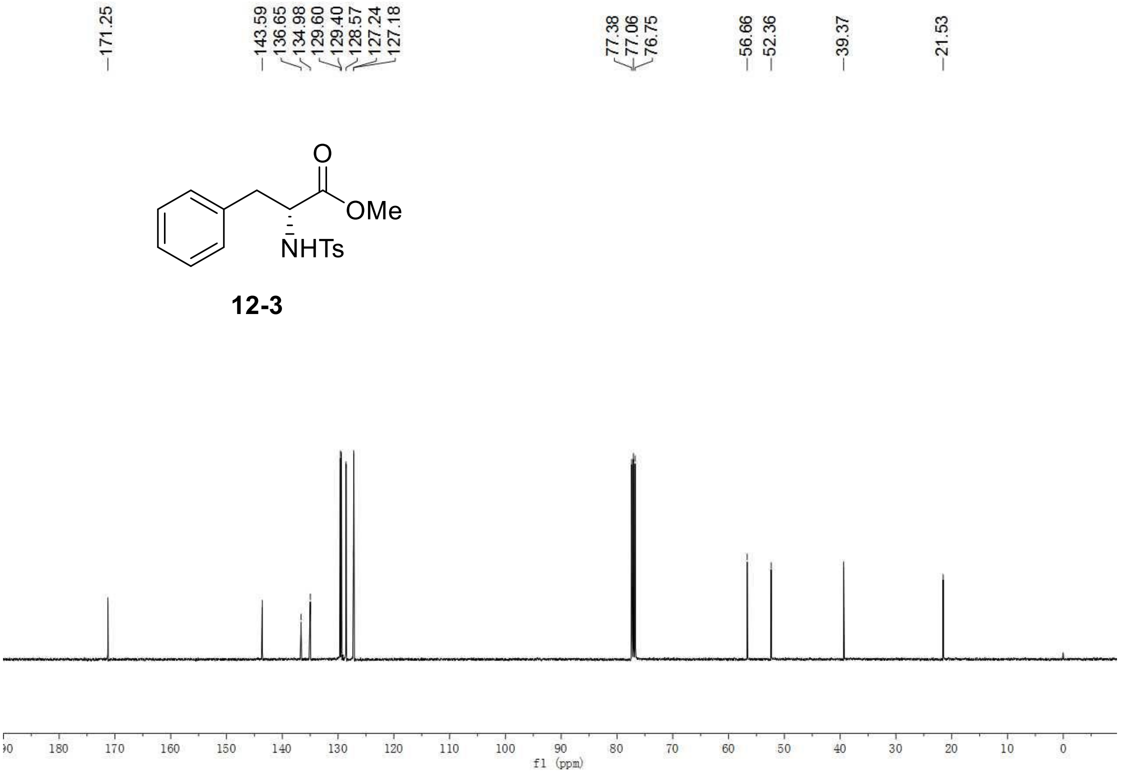

Supplementary Figure 121. ^{13}C NMR Spectrum of 12-3.

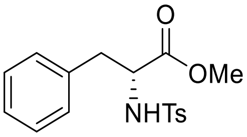

12-3

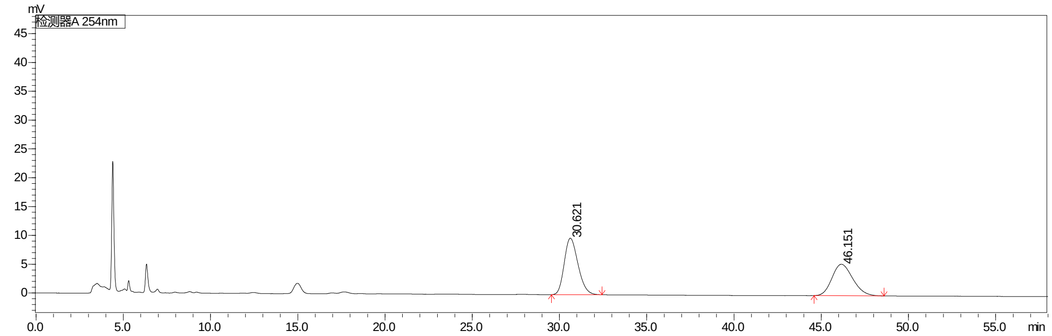

Peak	Ret. Time	Area	Height	Area%	Height%
1	30.621	539774	9813	54.358	64.248
2	46.151	453225	5460	45.642	35.752
Tolal		992998	15273	100.000	100.000

Supplementary Figure 122. HPLC Spectrum of racemic 12-3.

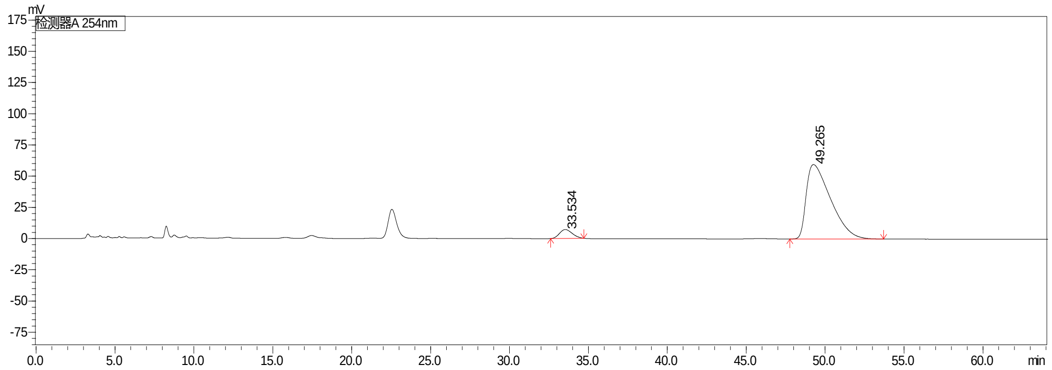

Peak	Ret. Time	Area	Height	Area%	Height%
1	33.534	400451	7152	5.889	10.712
2	49.265	6399649	59611	94.111	89.288
Tolal		6800100	66763	100.000	100.000

Supplementary Figure 123. HPLC Spectrum of 12-3.

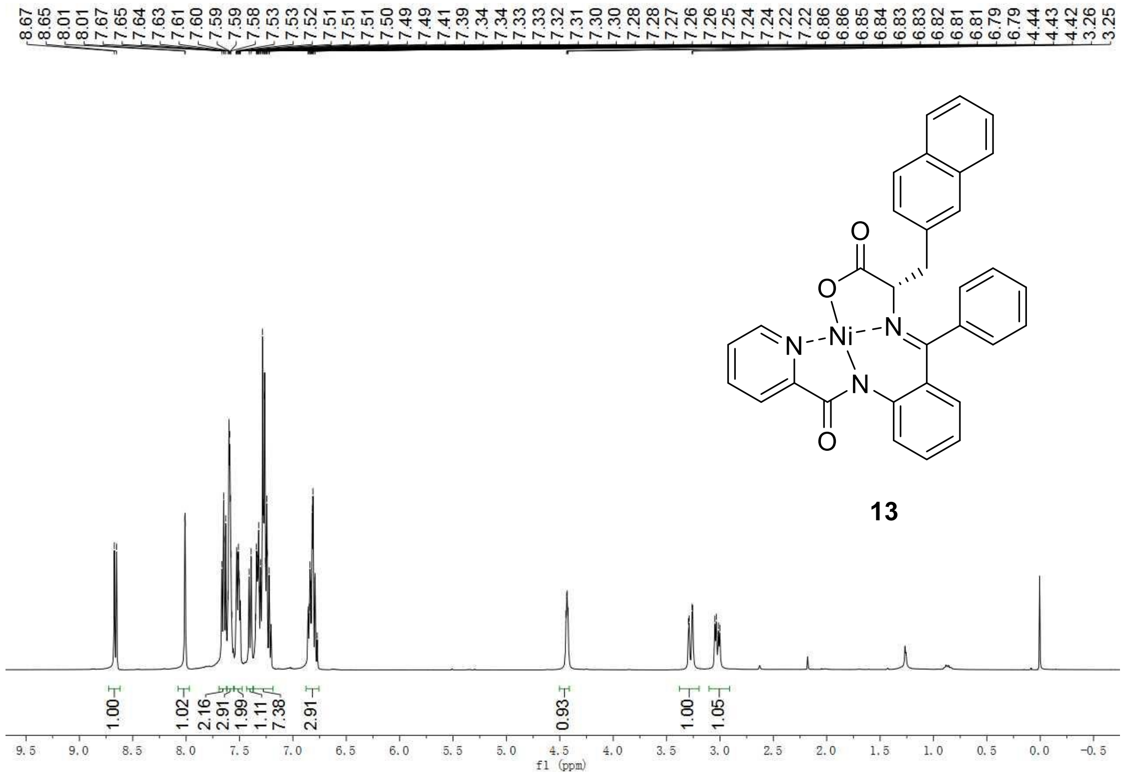

Supplementary Figure 124. ^{1}H NMR Spectrum of 13.

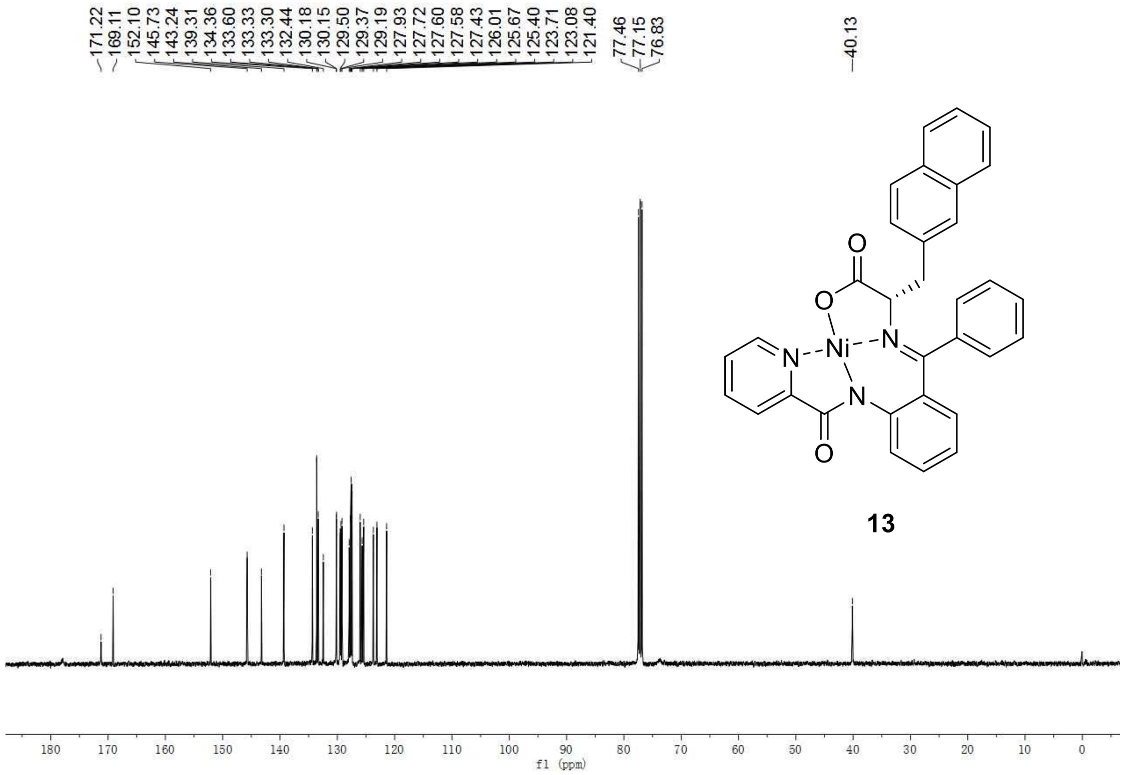

Supplementary Figure 125. ^{13}C NMR Spectrum of 13.

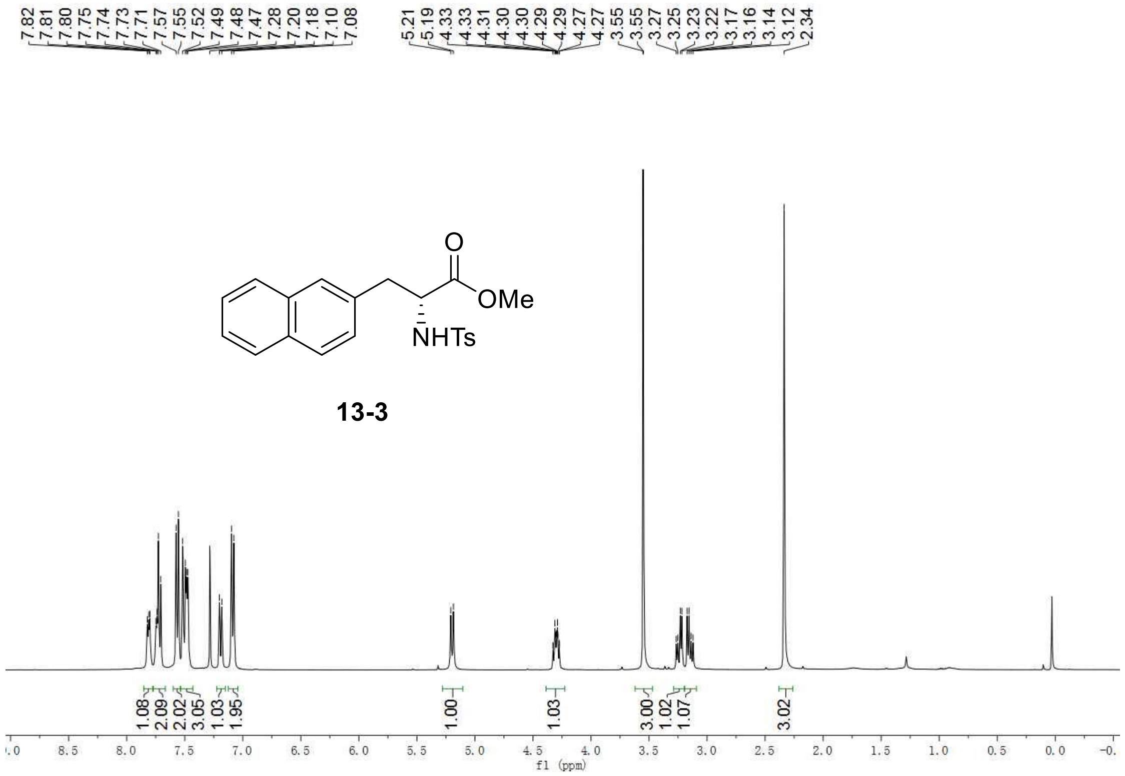

Supplementary Figure 126. ^{1}H NMR Spectrum of 13-3.

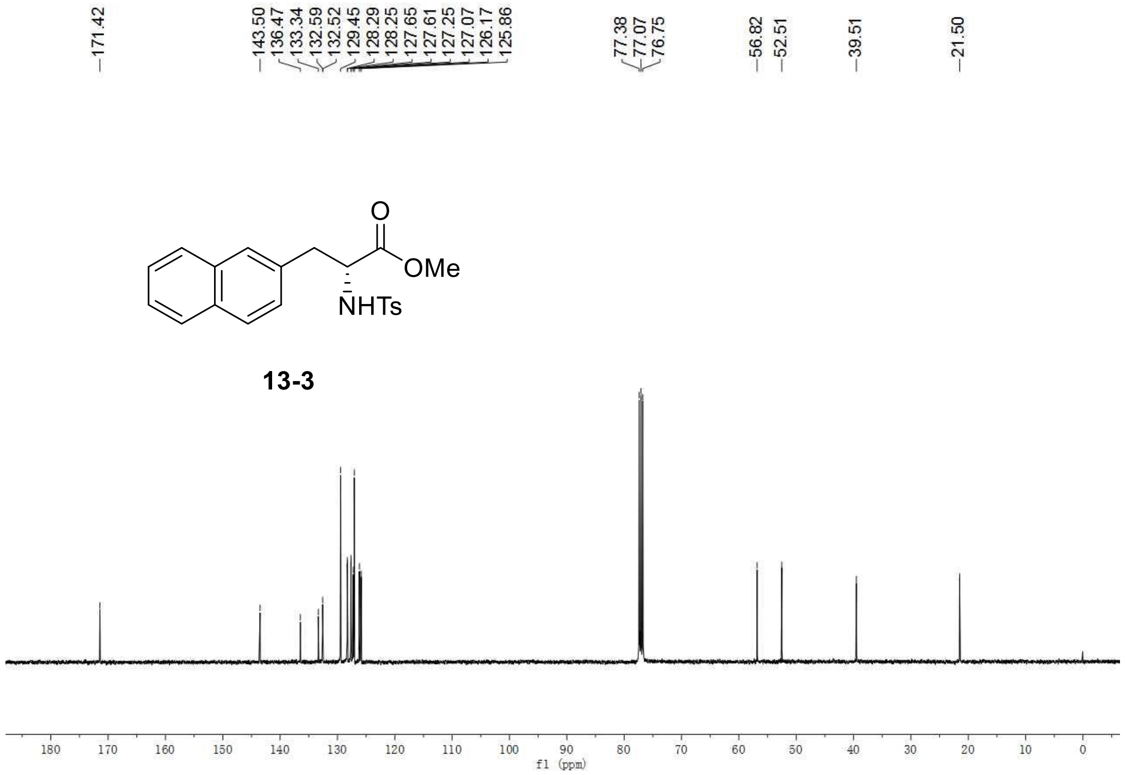

Supplementary Figure 127. ^{13}C NMR Spectrum of 13-3.

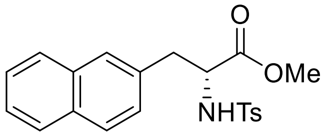

13-3

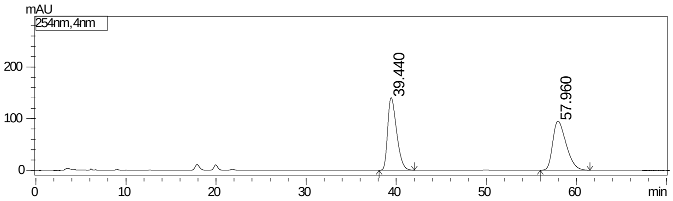

Peak	Ret. Time	Area	Height	Area%	Height%
1	39.440	9732149	140453	49.744	59.629
2	57.960	9832462	95093	50.256	40.371
Tolal		19564611	235546	100.000	100.000

Supplementary Figure 128. HPLC Spectrum of racemic 13-3.

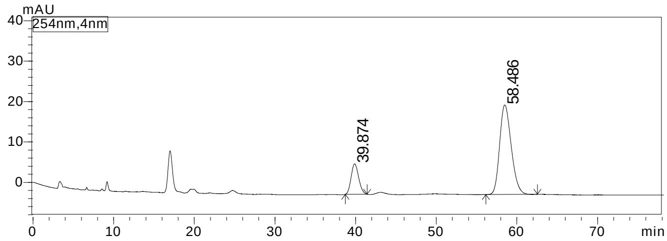

Peak	Ret. Time	Area	Height	Area%	Height%
1	39.874	491317	7538	18.125	25.358
2	58.486	2219393	22188	81.875	74.642
Tolal		2710710	29726	100.000	100.000

Supplementary Figure 129. HPLC Spectrum of 13-3.

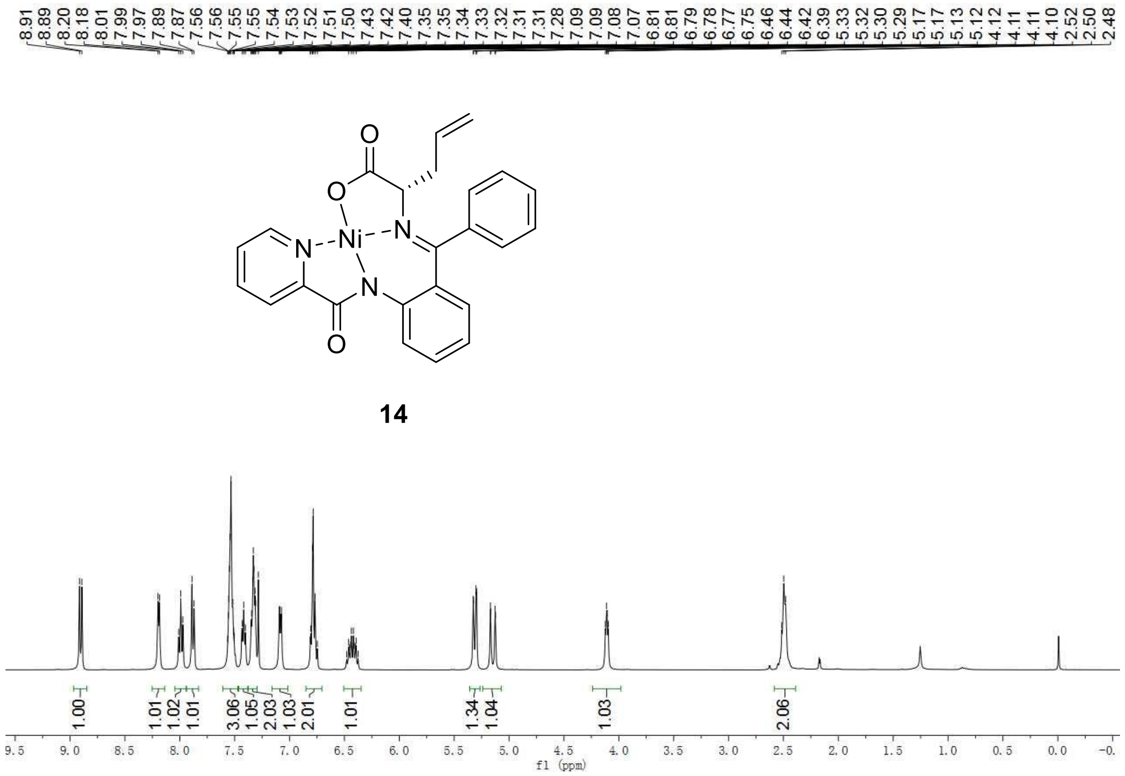

Supplementary Figure 130. ^{1}H NMR Spectrum of 14.

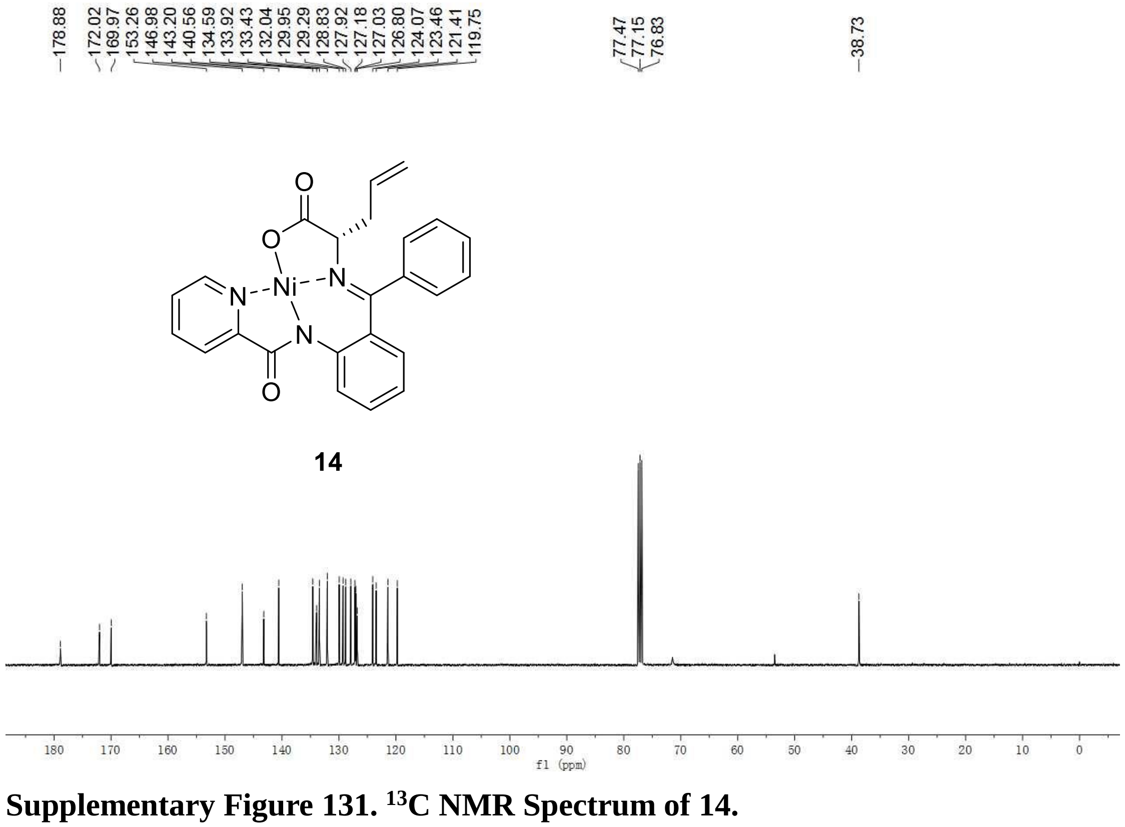

Supplementary Figure 131. ^{13}C NMR Spectrum of 14.

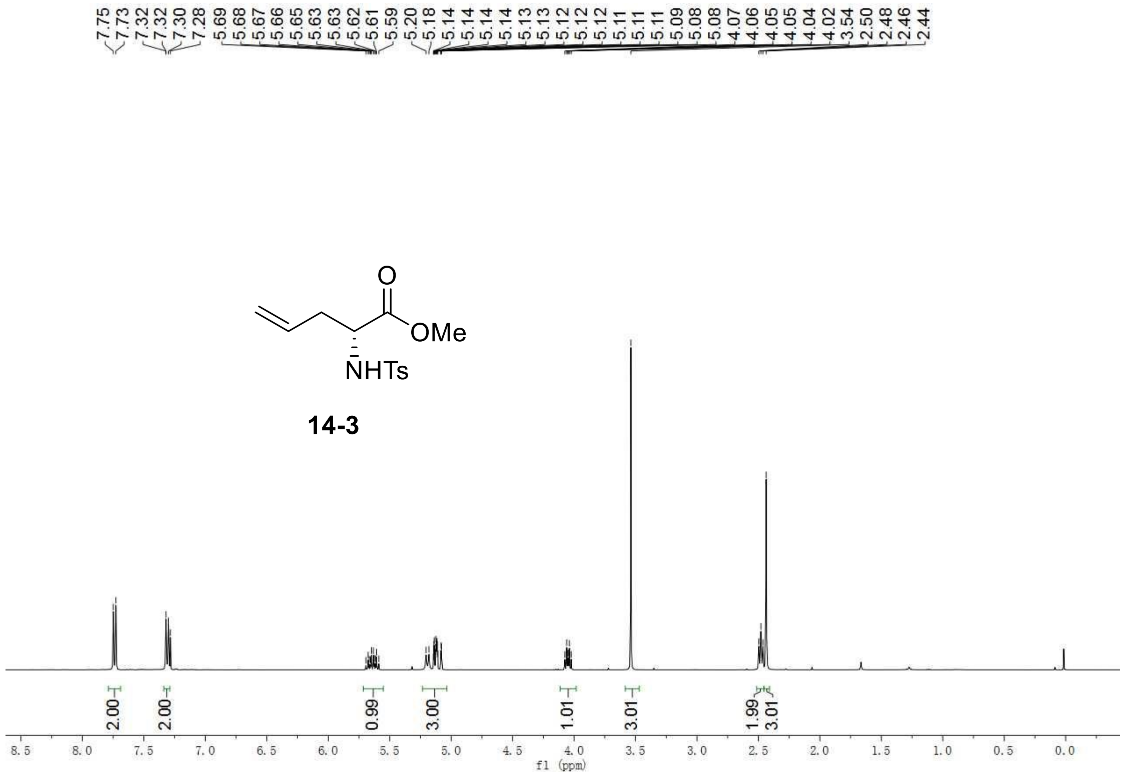

Supplementary Figure 132. ^{1}H NMR Spectrum of 14-3.

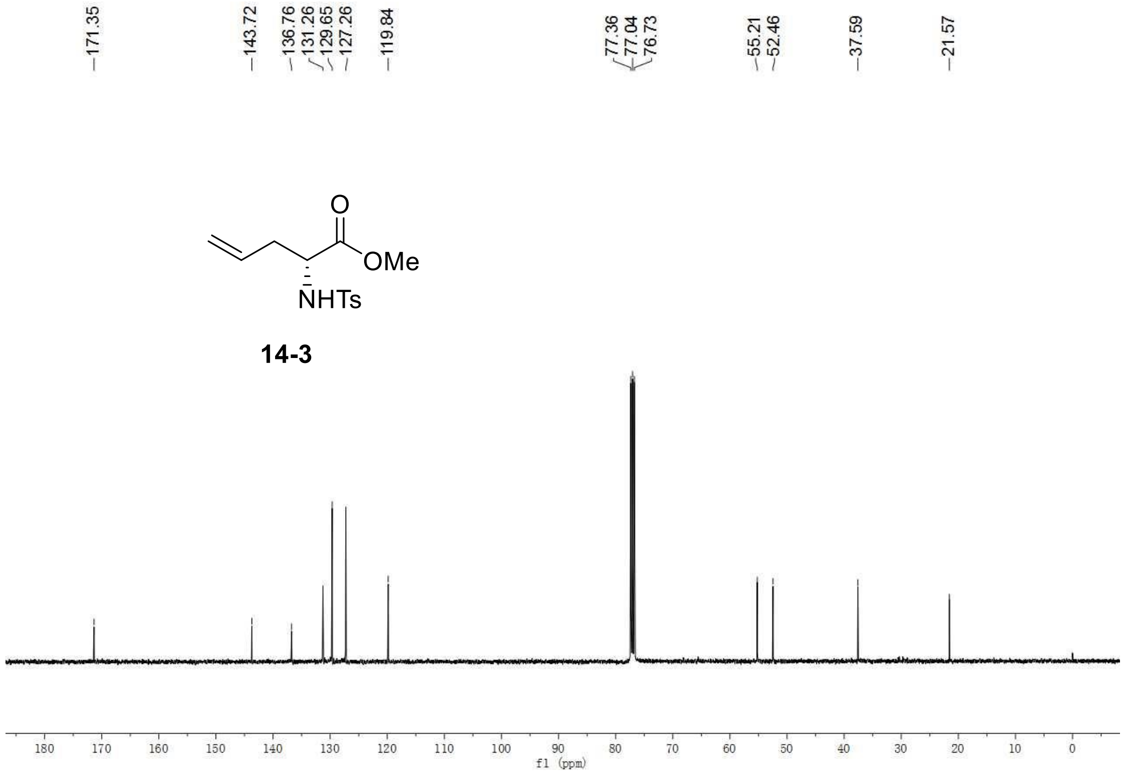

Supplementary Figure 133. ^{13}C NMR Spectrum of 14-3.

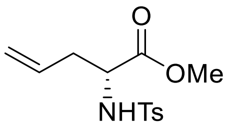

14-3

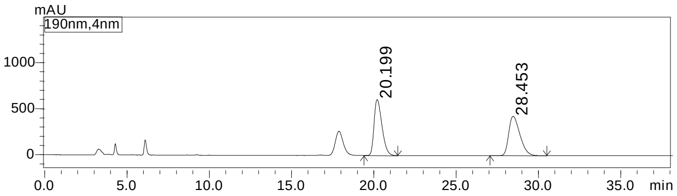

Peak	Ret. Time	Area	Height	Area%	Height%
1	20.199	20280550	608115	50.105	58.665
2	28.453	20195543	428467	49.895	41.335
Tolal		40476093	1036582	100.000	100.000

Supplementary Figure 134. HPLC Spectrum of racemic 14-3.

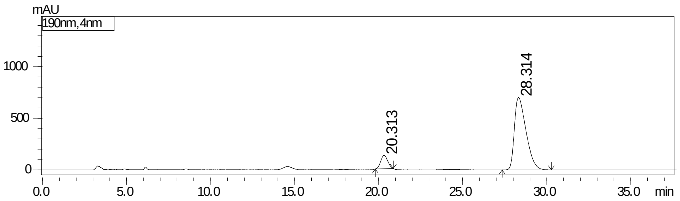

Peak	Ret. Time	Area	Height	Area%	Height%
1	20.313	3821599	131468	10.163	15.769
2	28.314	33779769	702261	89.837	84.231
Tolal		37601368	833730	100.000	100.000

Supplementary Figure 135. HPLC Spectrum of 14-3.

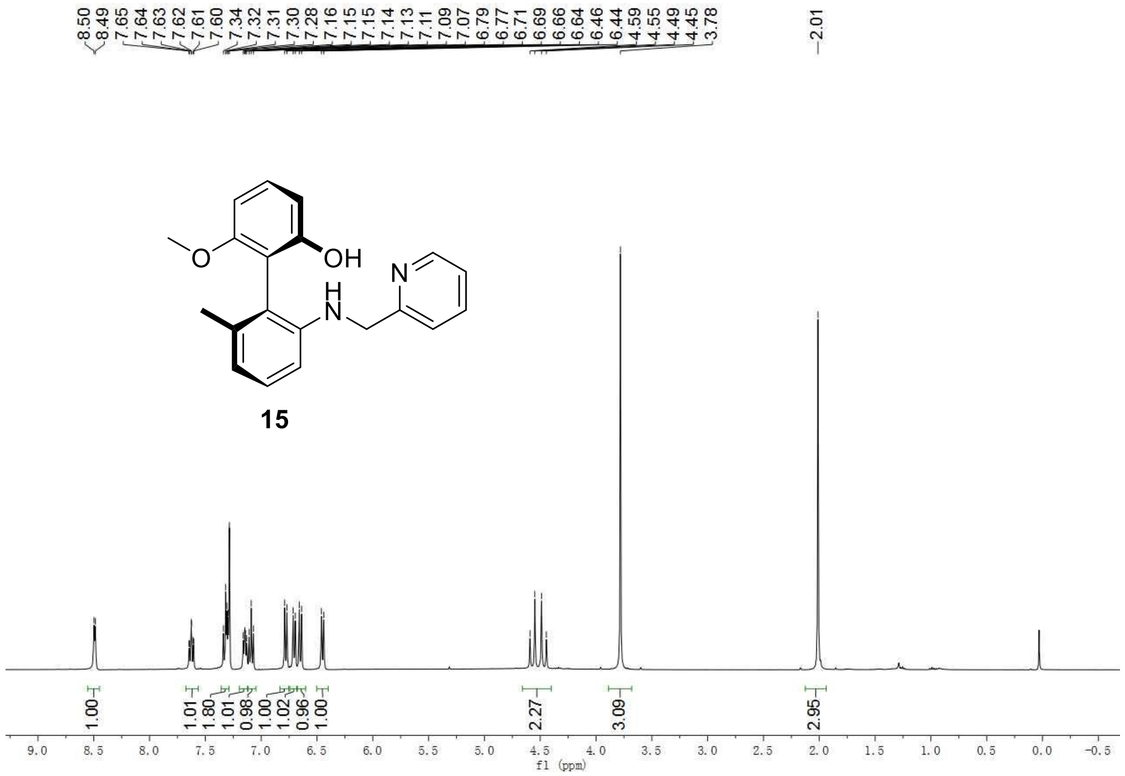

Supplementary Figure 136. ^{1}H NMR Spectrum of 15.

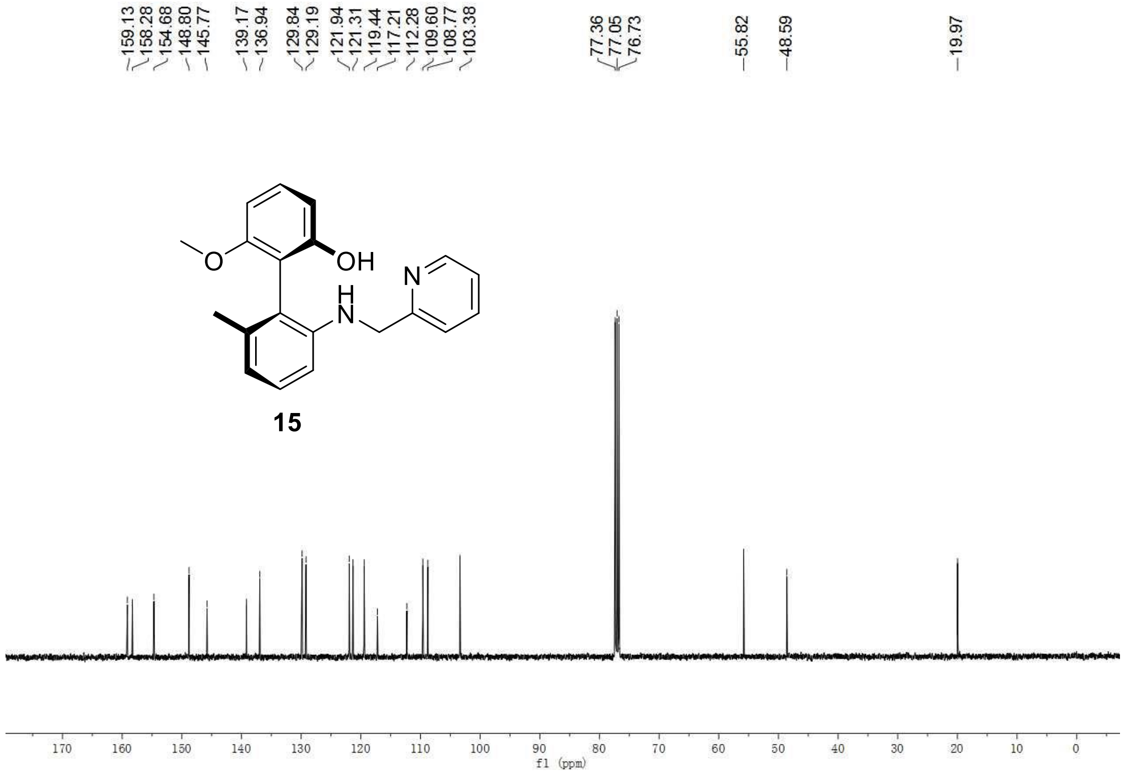

Supplementary Figure 137. ^{13}C NMR Spectrum of 15.

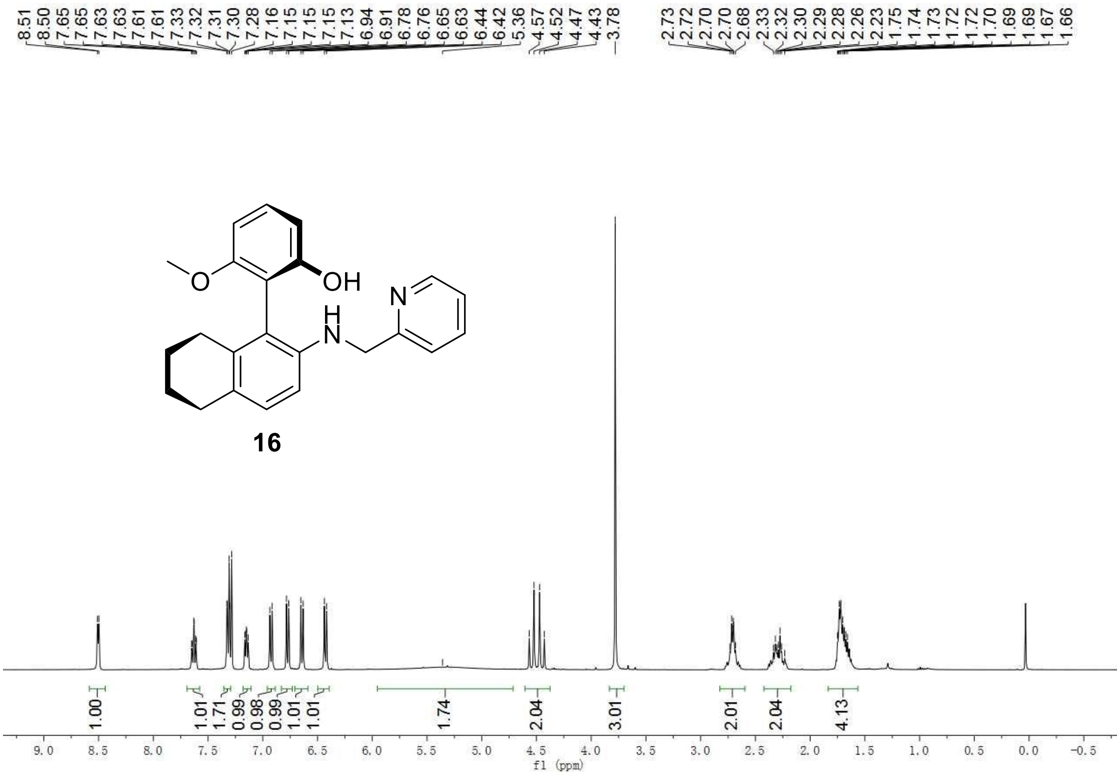

Supplementary Figure 138. ^{1}H NMR Spectrum of 16.

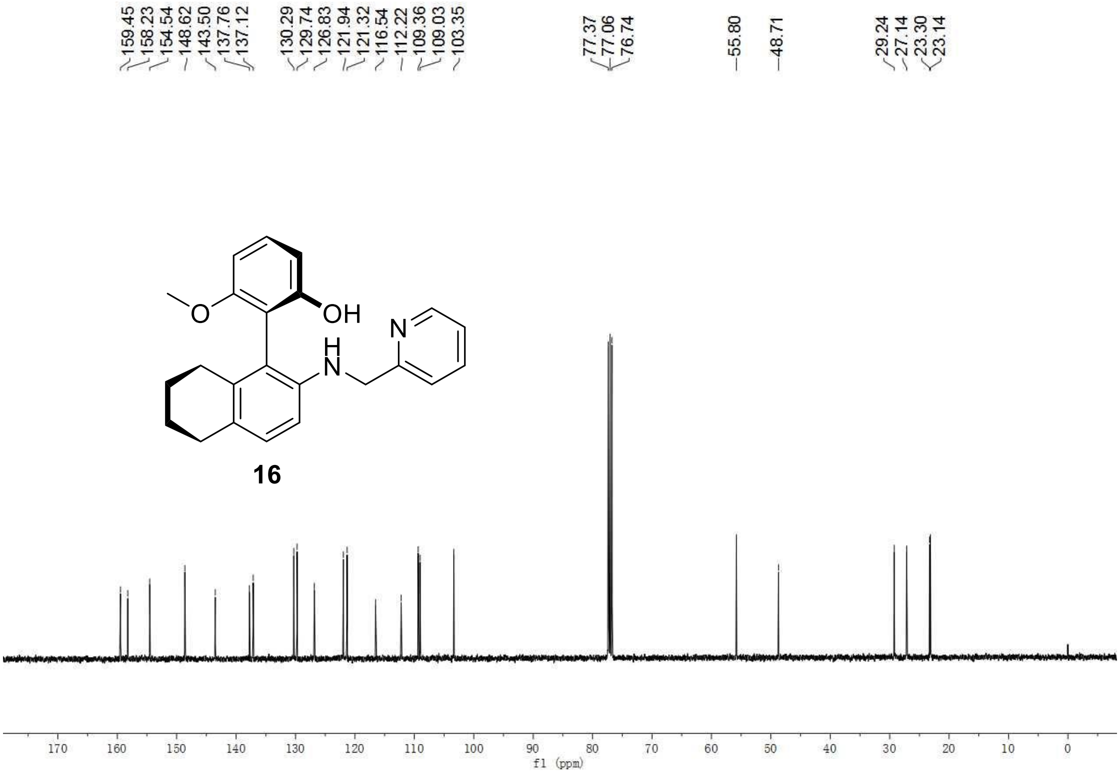

Supplementary Figure 139. ^{13}C NMR Spectrum of 16.

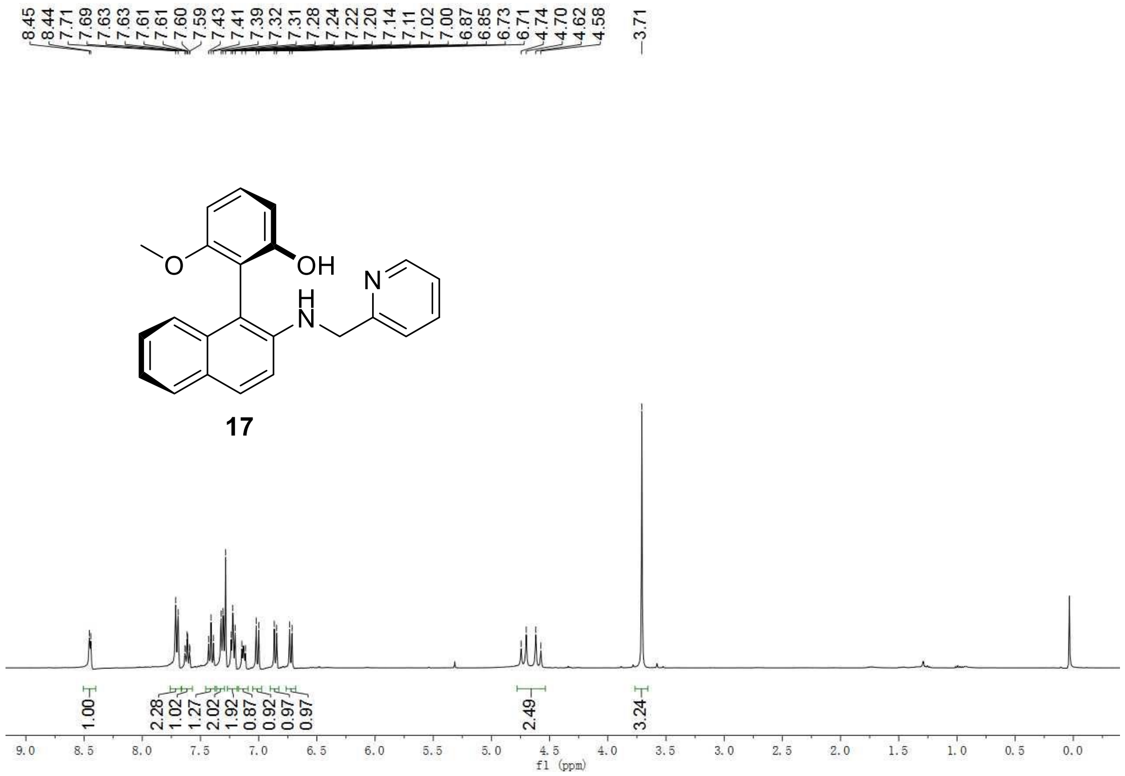

Supplementary Figure 140. ^{1}H NMR Spectrum of 17.

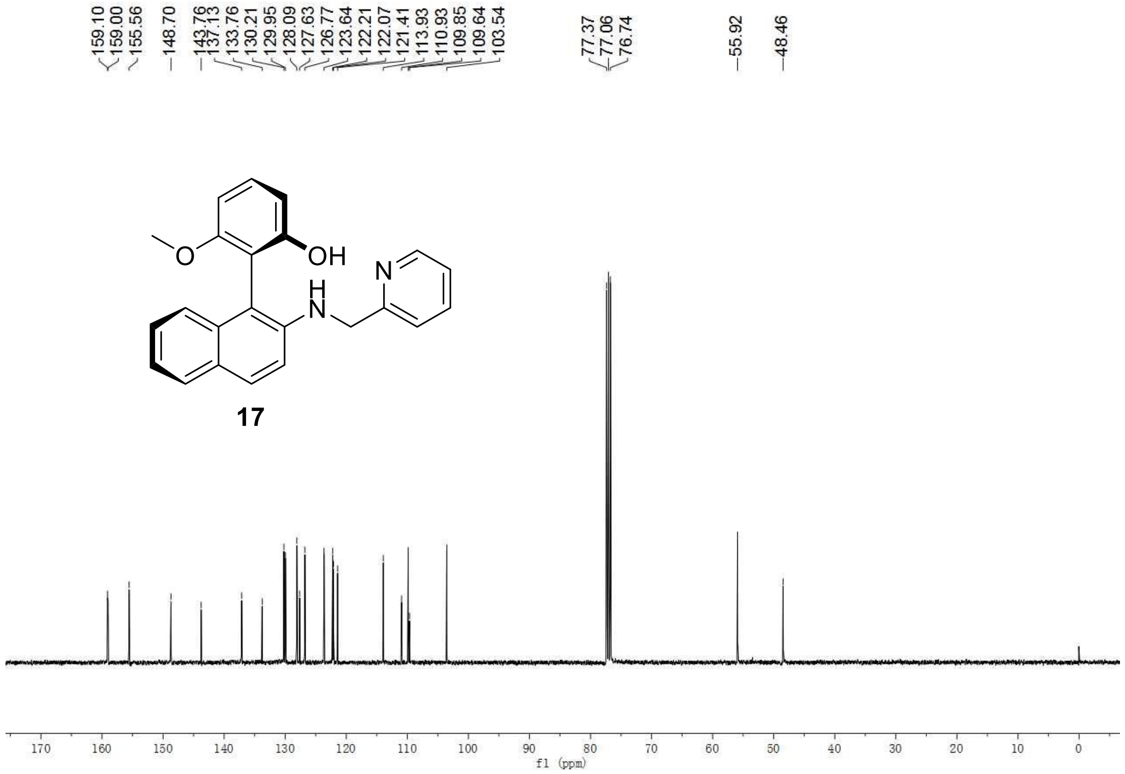

Supplementary Figure 141. ^{13}C NMR Spectrum of 17.

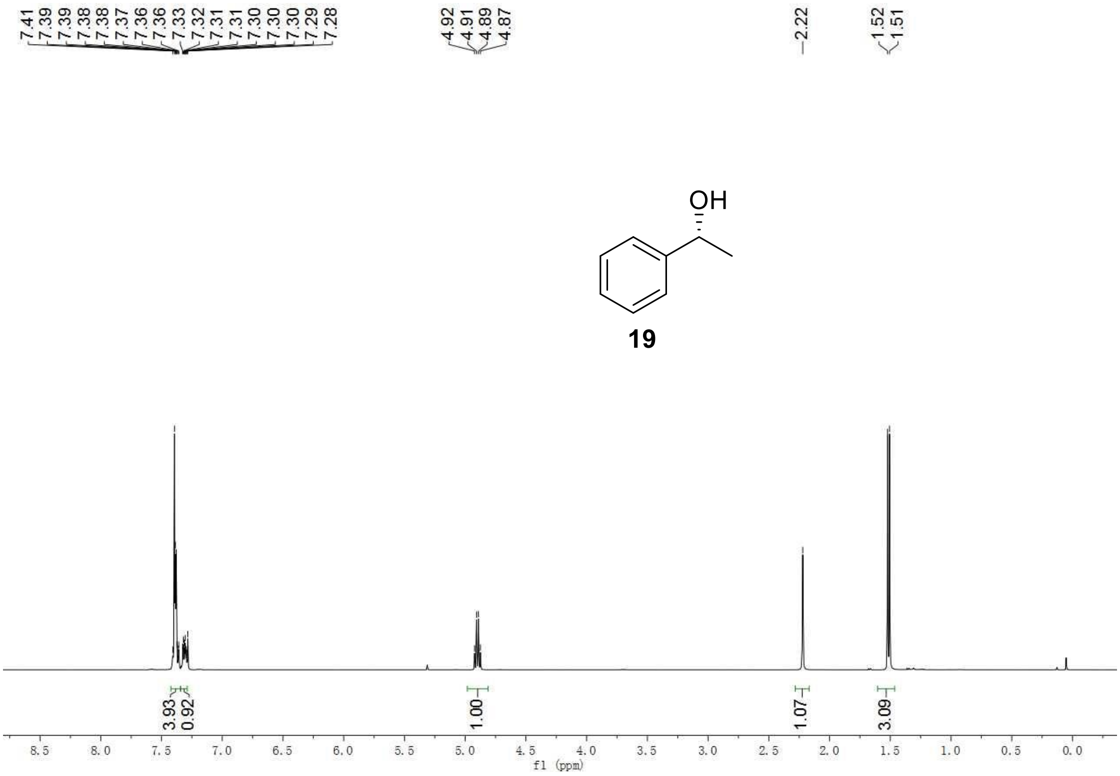

Supplementary Figure 142. ^{1}H NMR Spectrum of 19.

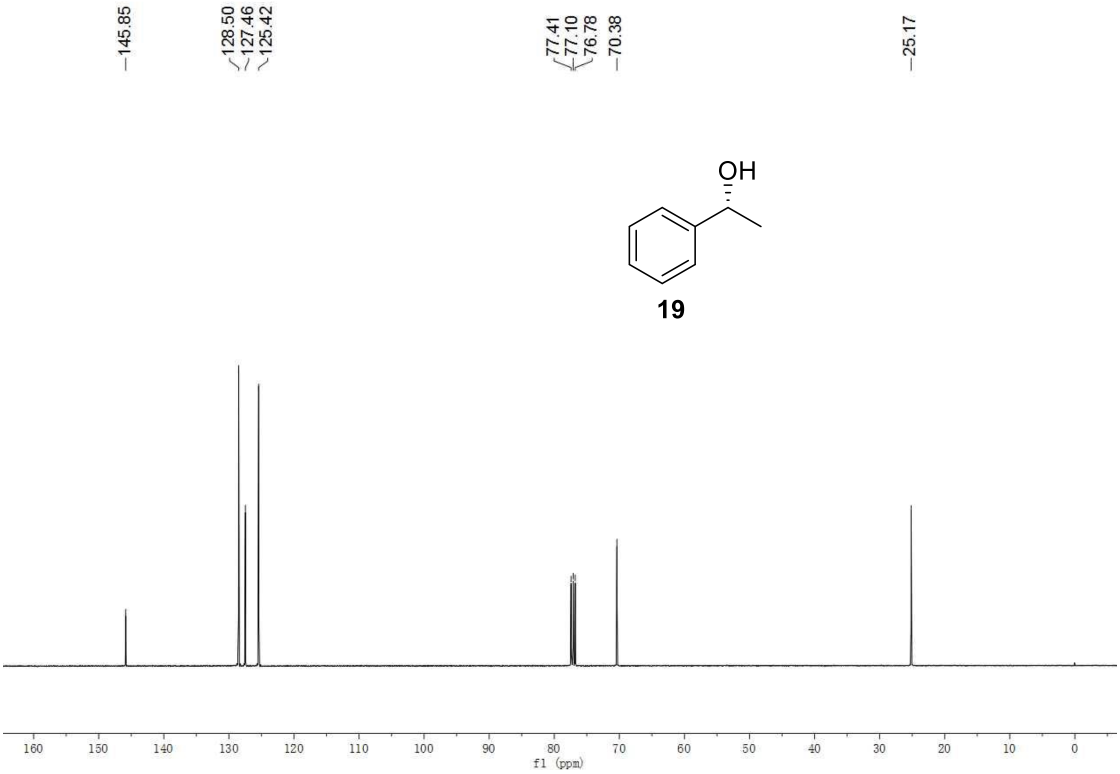

Supplementary Figure 143. ^{13}C NMR Spectrum of 19.

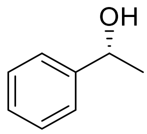

19 - L15

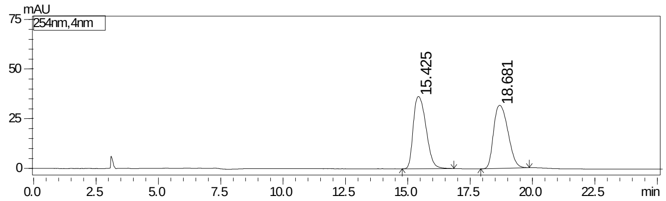

Peak	Ret. Time	Area	Height	Area%	Height%
1	15.425	1294250	36345	50.396	53.340
2	18.681	1273900	31793	49.604	46.660
Tolal		2568150	68138	100.000	100.000

Supplementary Figure 144. HPLC Spectrum of racemic 19-L15.

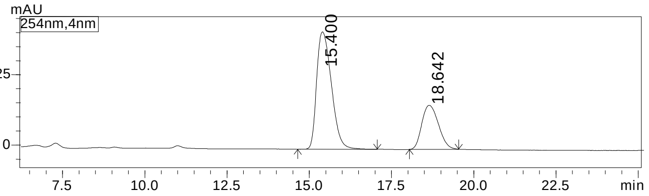

Peak	Ret. Time	Area	Height	Area%	Height%
1	15.400	1302531	41708	70.312	72.736
2	18.642	549961	15634	29.688	27.264
Tolal		1852492	57342	100.000	100.000

Supplementary Figure 145. HPLC Spectrum of 19-L15.

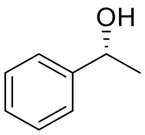

19 - L16

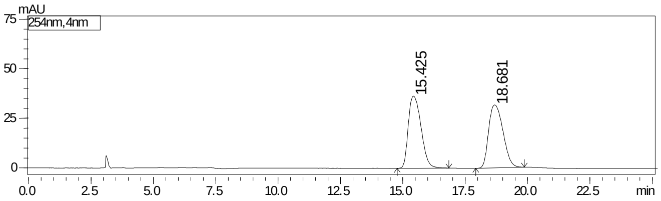

Peak	Ret. Time	Area	Height	Area%	Height%
1	15.425	1294250	36345	50.396	53.340
2	18.681	1273900	31793	49.604	46.660
Tolal		2568150	68138	100.000	100.000

Supplementary Figure 146. HPLC Spectrum of racemic 19-L16.

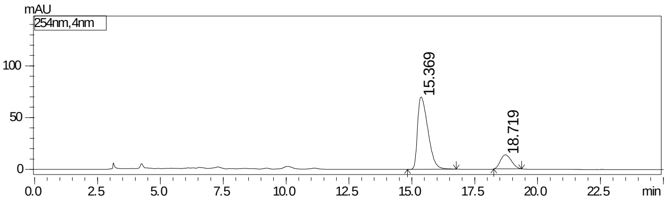

Peak	Ret. Time	Area	Height	Area%	Height%
1	15.369	2006950	69963	82.958	83.794
2	18.719	412292	13531	17.042	16.206
Tolal		2419242	83494	100.000	100.000

Supplementary Figure 147. HPLC Spectrum of 19-L16.

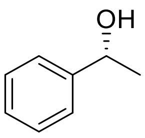

19 - L17

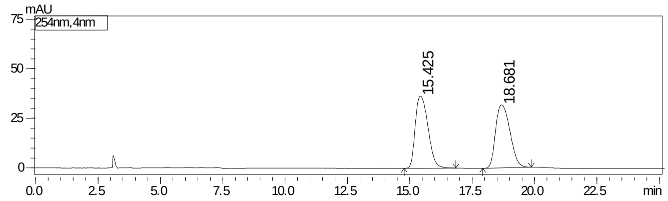

Peak	Ret. Time	Area	Height	Area%	Height%
1	15.425	1294250	36345	50.396	53.340
2	18.681	1273900	31793	49.604	46.660
Tolal		2568150	68138	100.000	100.000

Supplementary Figure 148. HPLC Spectrum of racemic 19-L17.

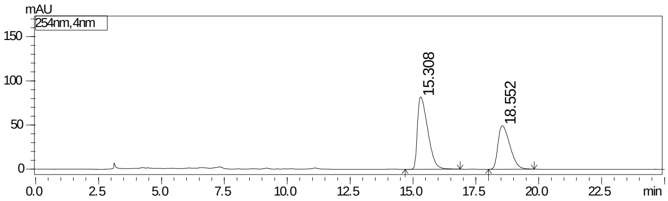

Peak	Ret. Time	Area	Height	Area%	Height%
1	15.308	2342081	81988	59.128	62.481
2	18.552	1618975	49233	40.872	37.519
Tolal		3961056	131220	100.000	100.000

Supplementary Figure 149. HPLC Spectrum of 19-L17.

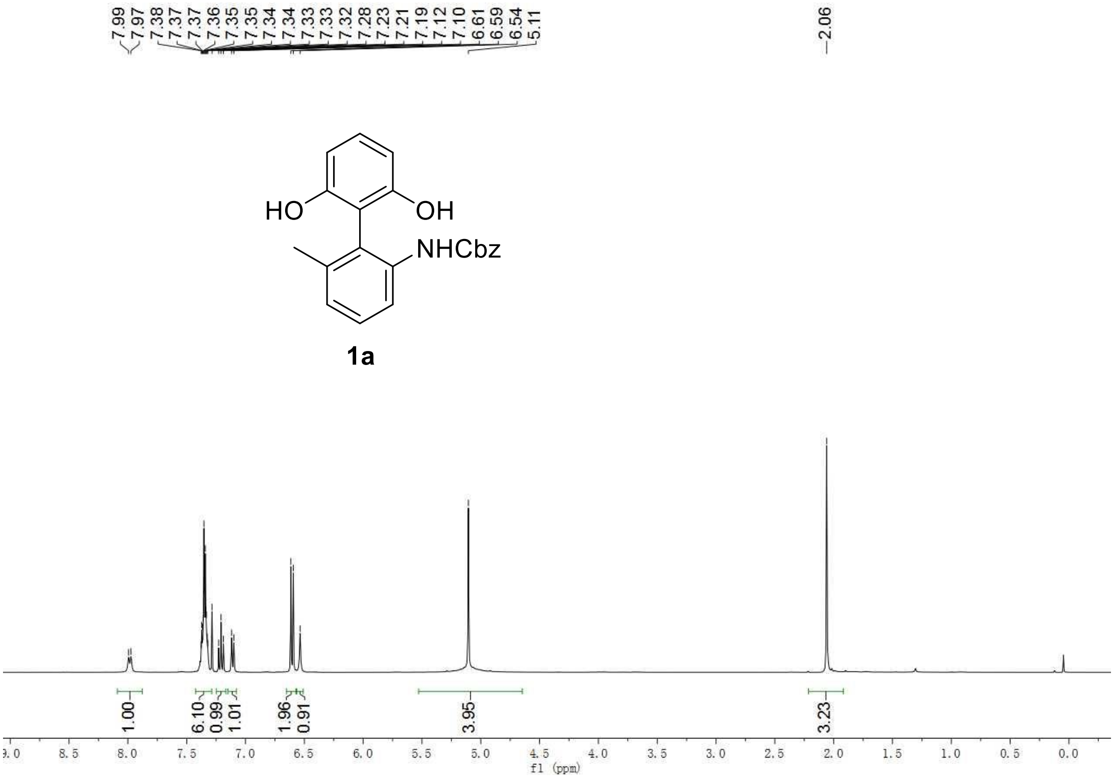

Supplementary Figure 150. ^{1}**H NMR Spectrum of 1a.**

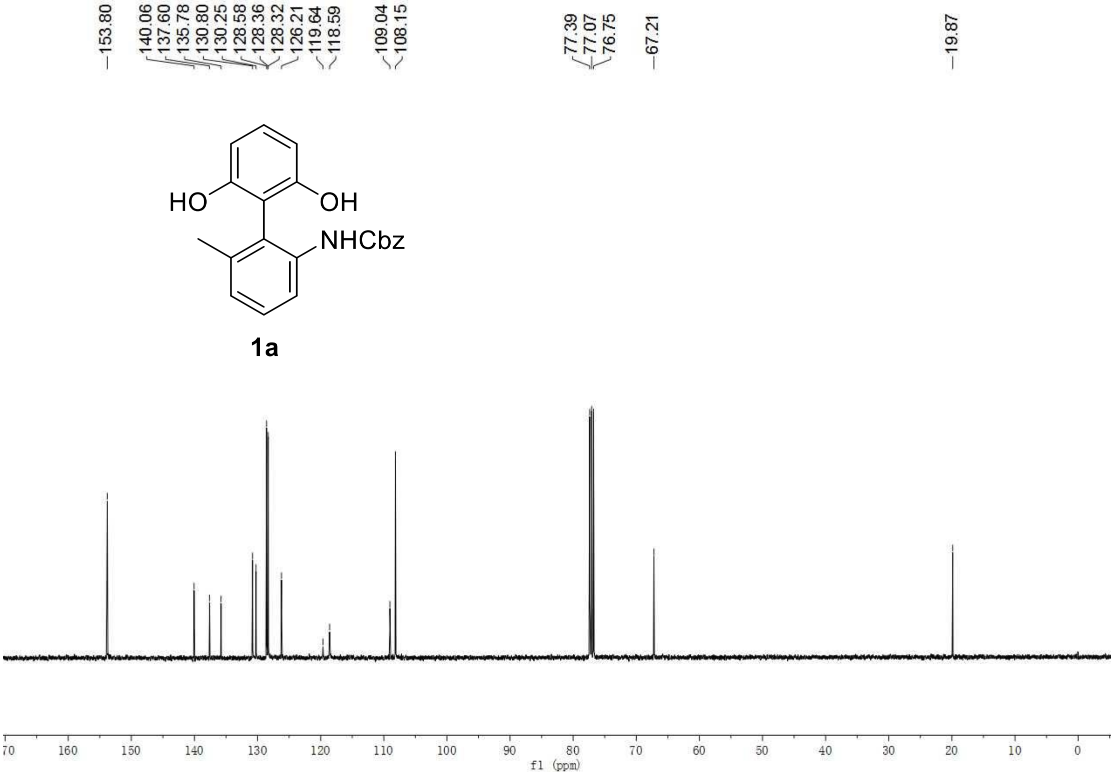

Supplementary Figure 151. ^{13}**C NMR Spectrum of 1a.**

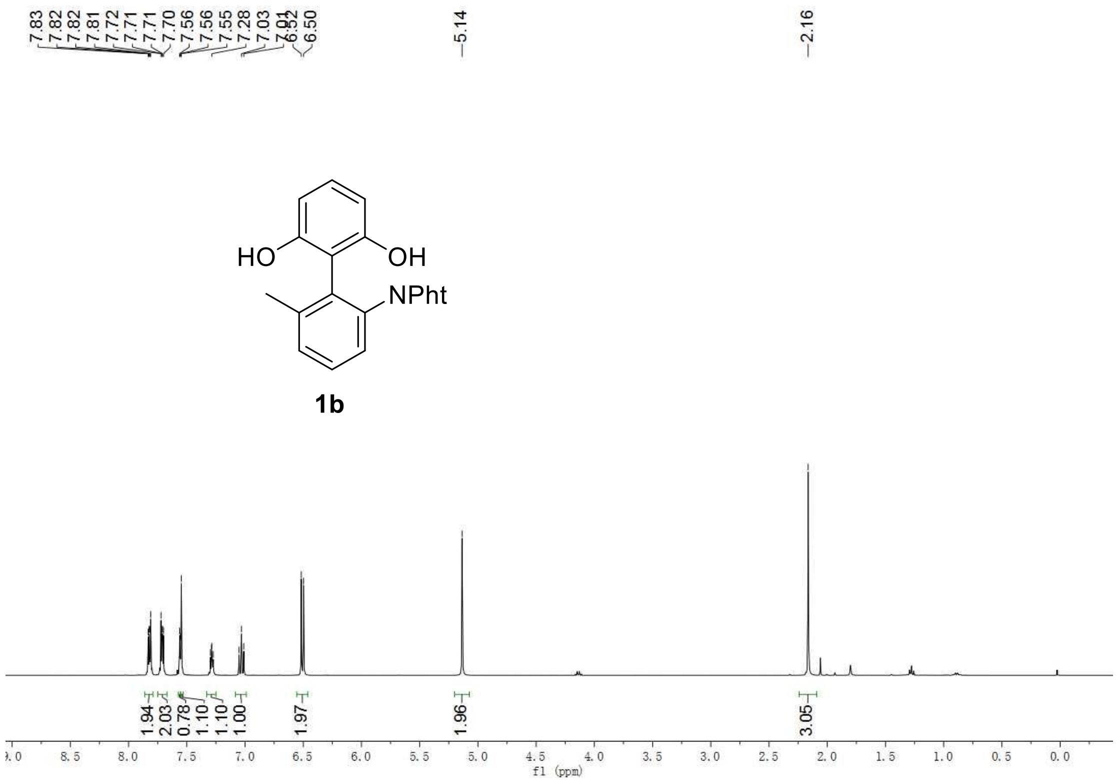

Supplementary Figure 152. ^{1}H NMR Spectrum of 1b.

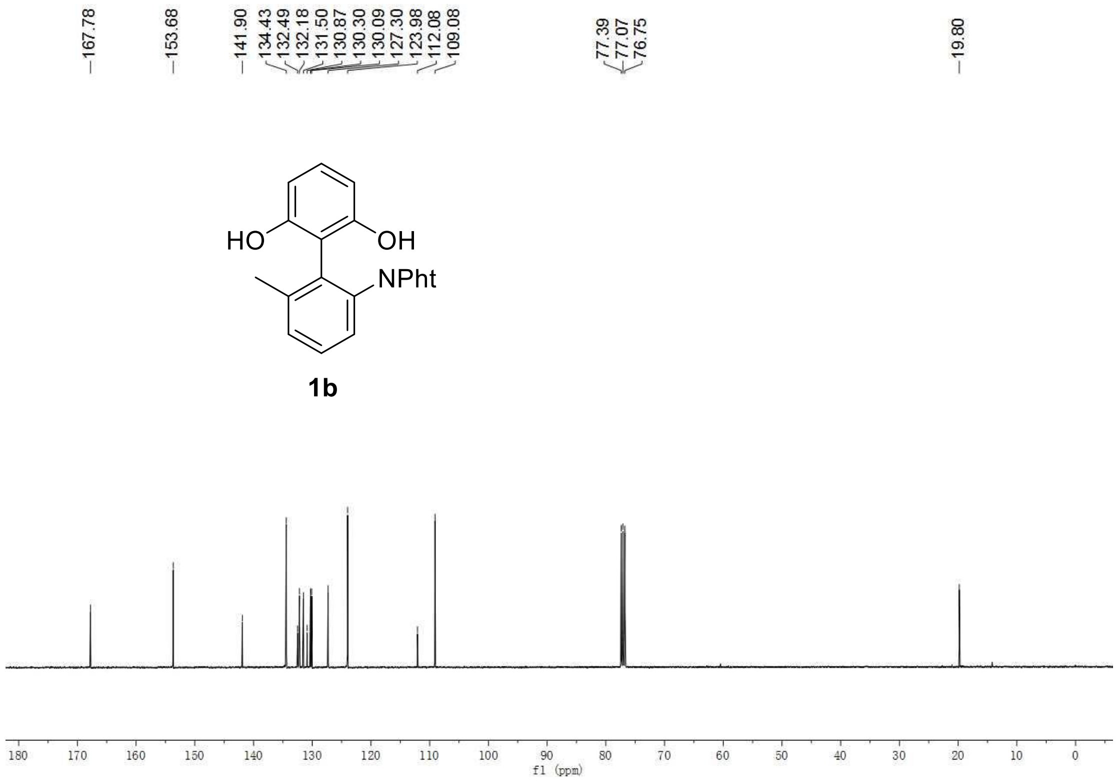

Supplementary Figure 153. ^{13}C NMR Spectrum of 1b.

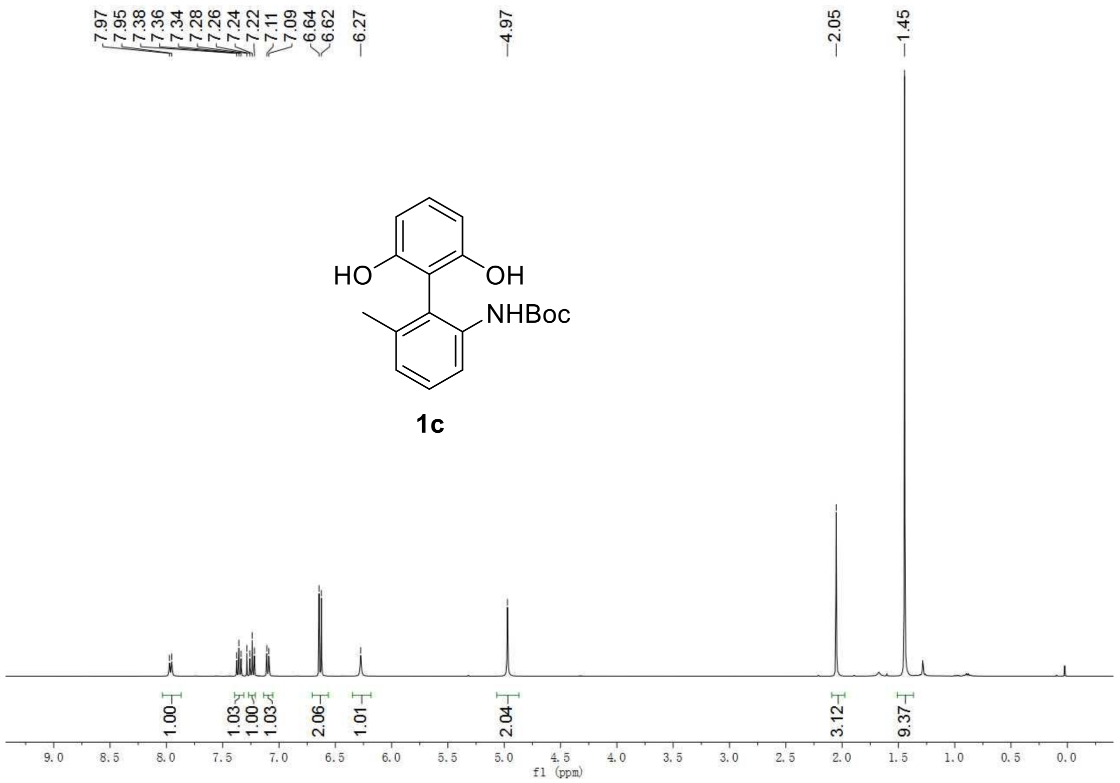

Supplementary Figure 154. ^{1}H NMR Spectrum of 1c.

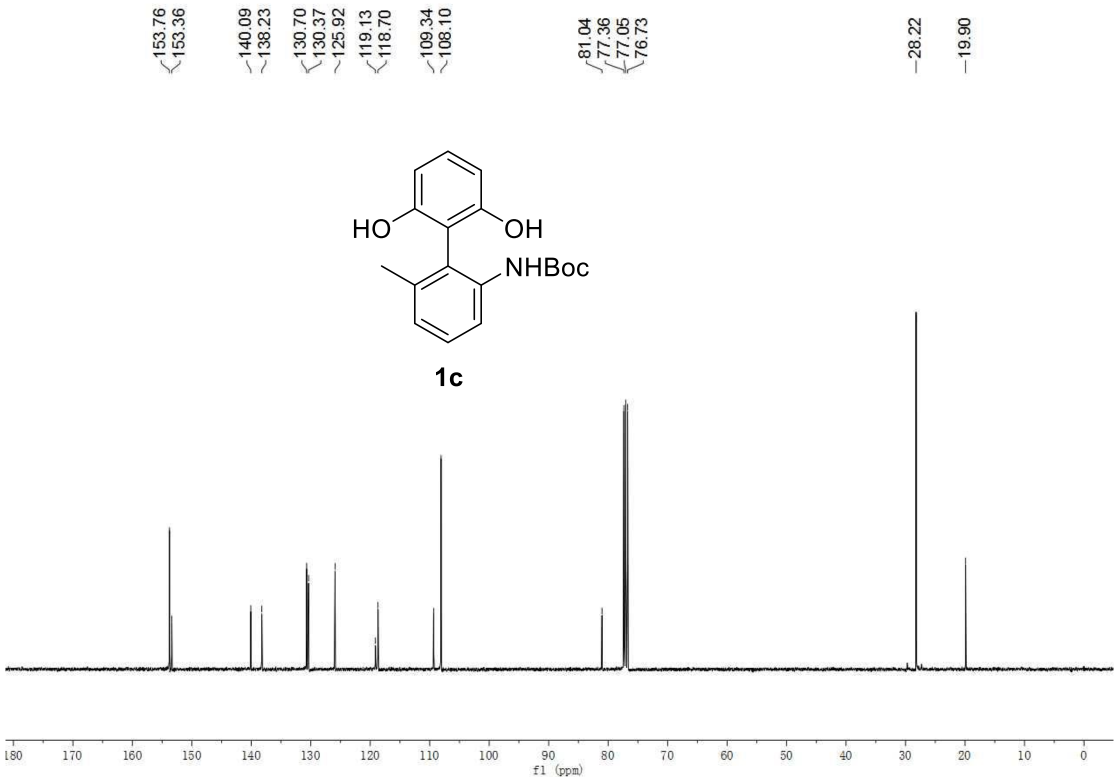

Supplementary Figure 155. ^{13}C NMR Spectrum of 1c.

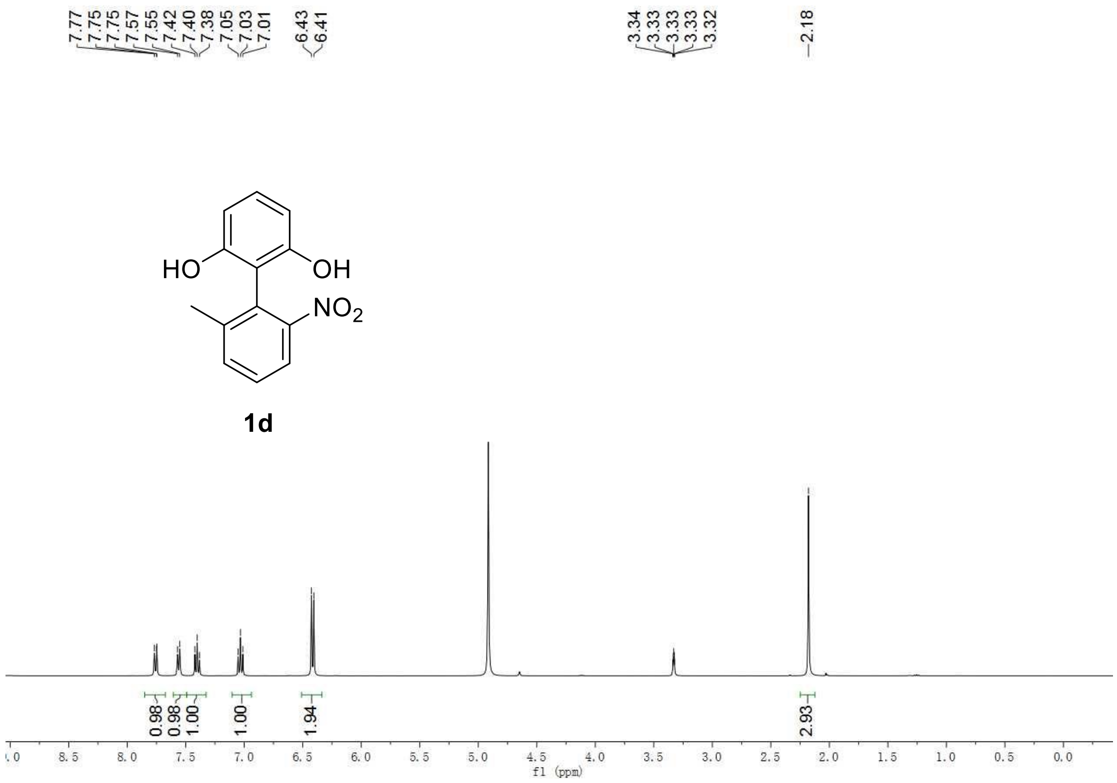

Supplementary Figure 156. ^{1}H NMR Spectrum of 1d.

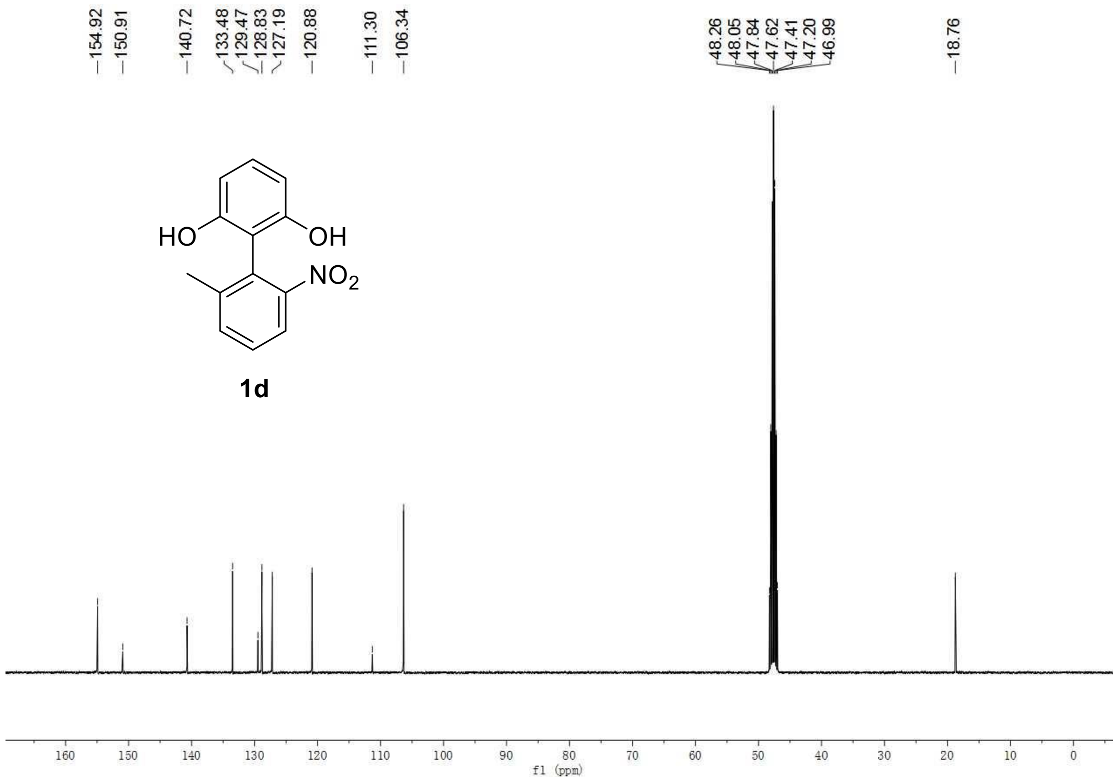

Supplementary Figure 157. ^{13}C NMR Spectrum of 1d.

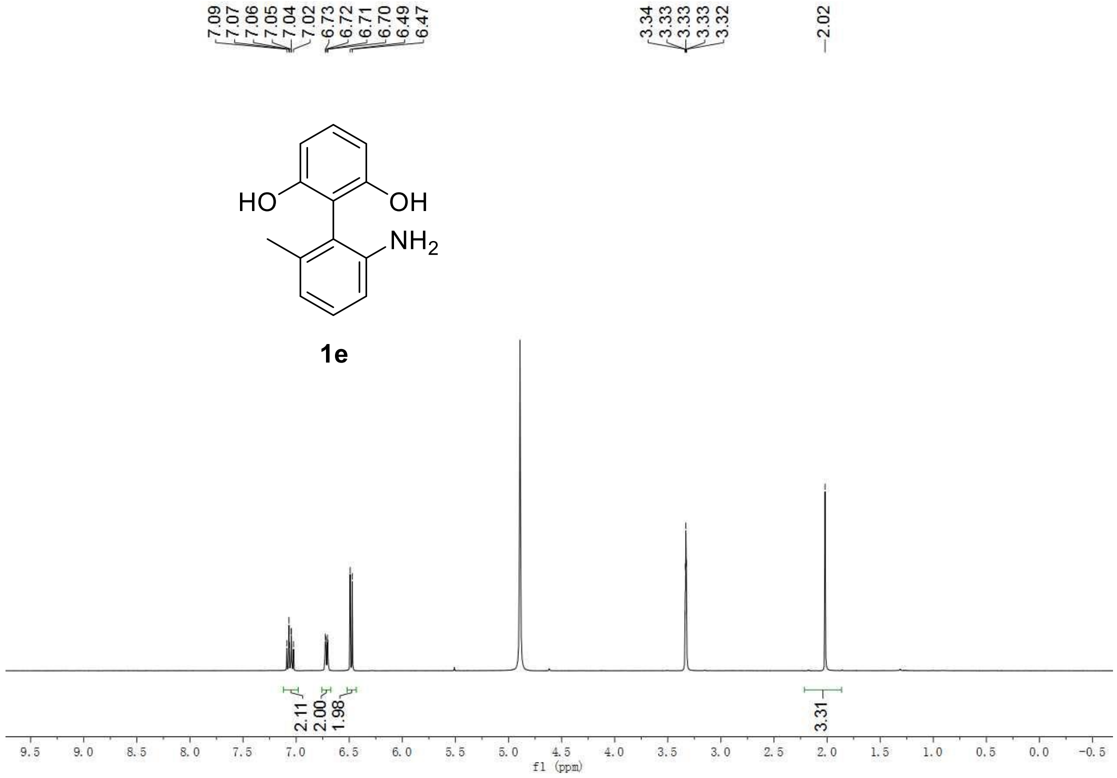

Supplementary Figure 158. ^{1}H NMR Spectrum of 1e.

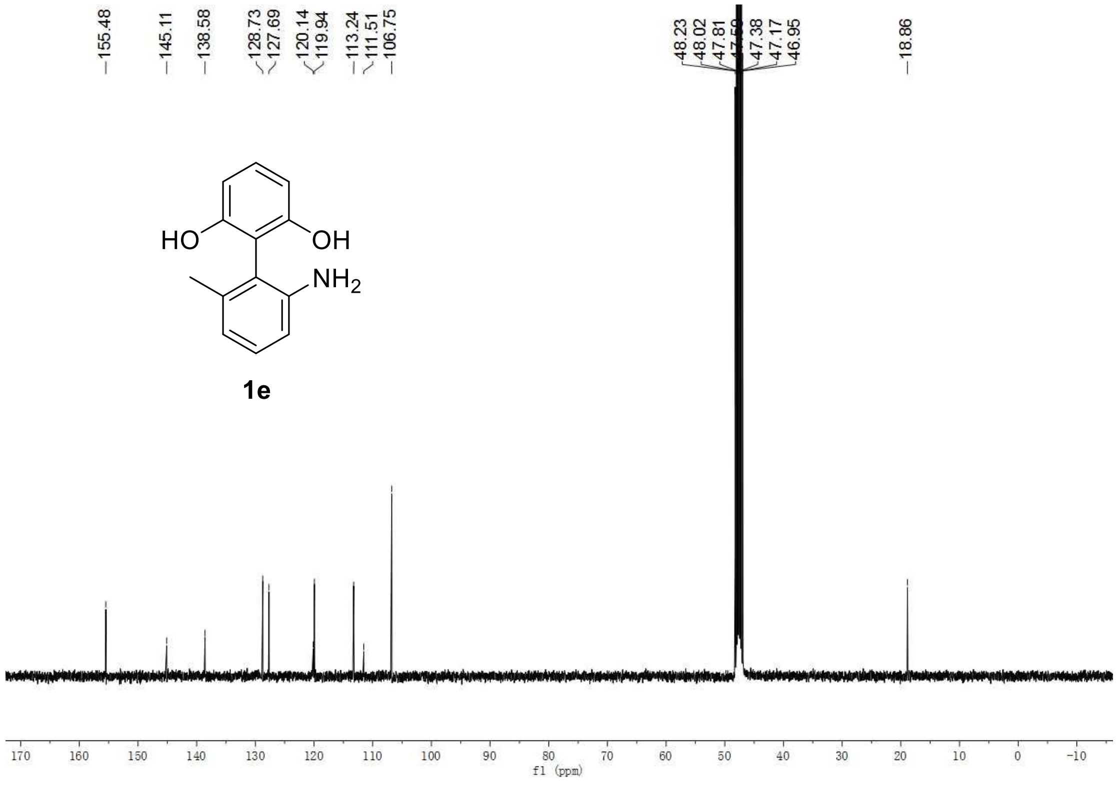

Supplementary Figure 159. ^{13}C NMR Spectrum of 1e.

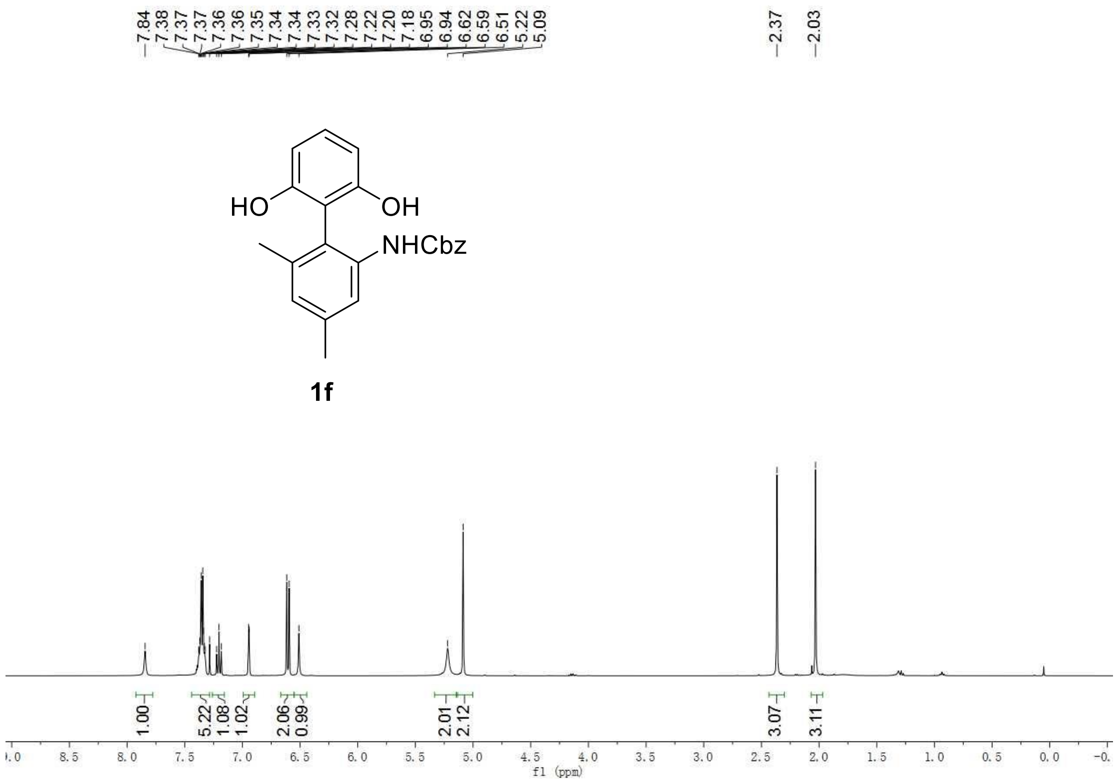

Supplementary Figure 160. ^{1}H NMR Spectrum of 1f.

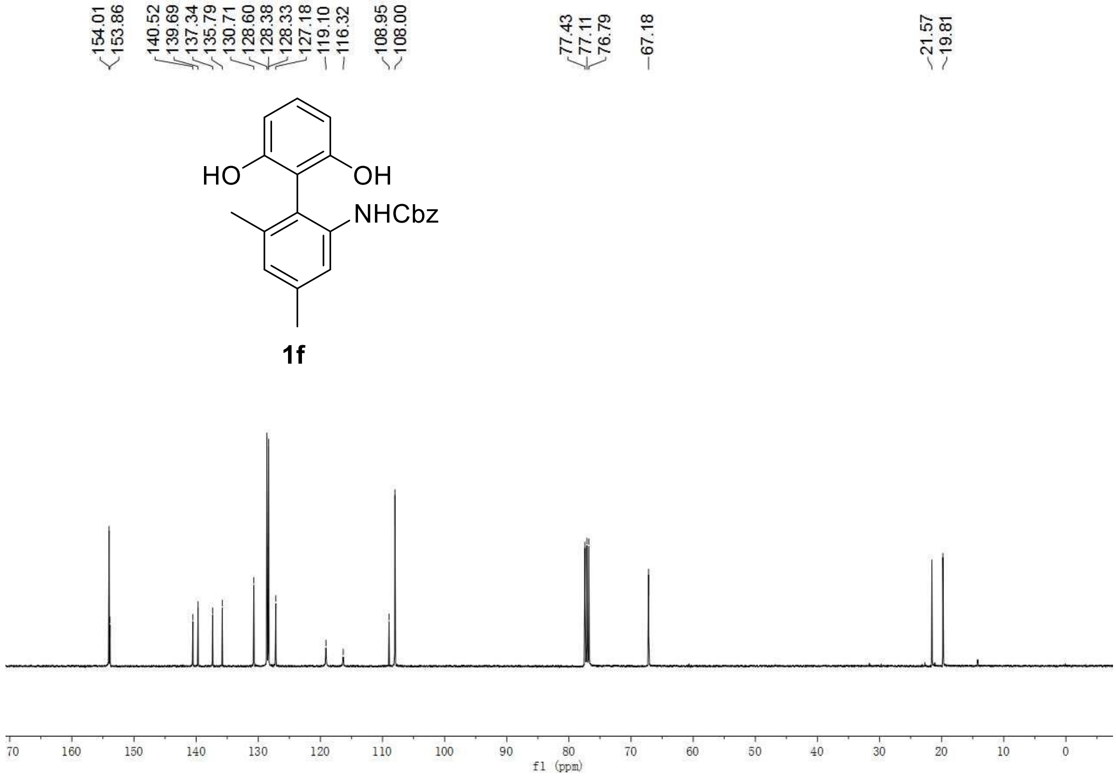

Supplementary Figure 161. ^{13}C NMR Spectrum of 1f.

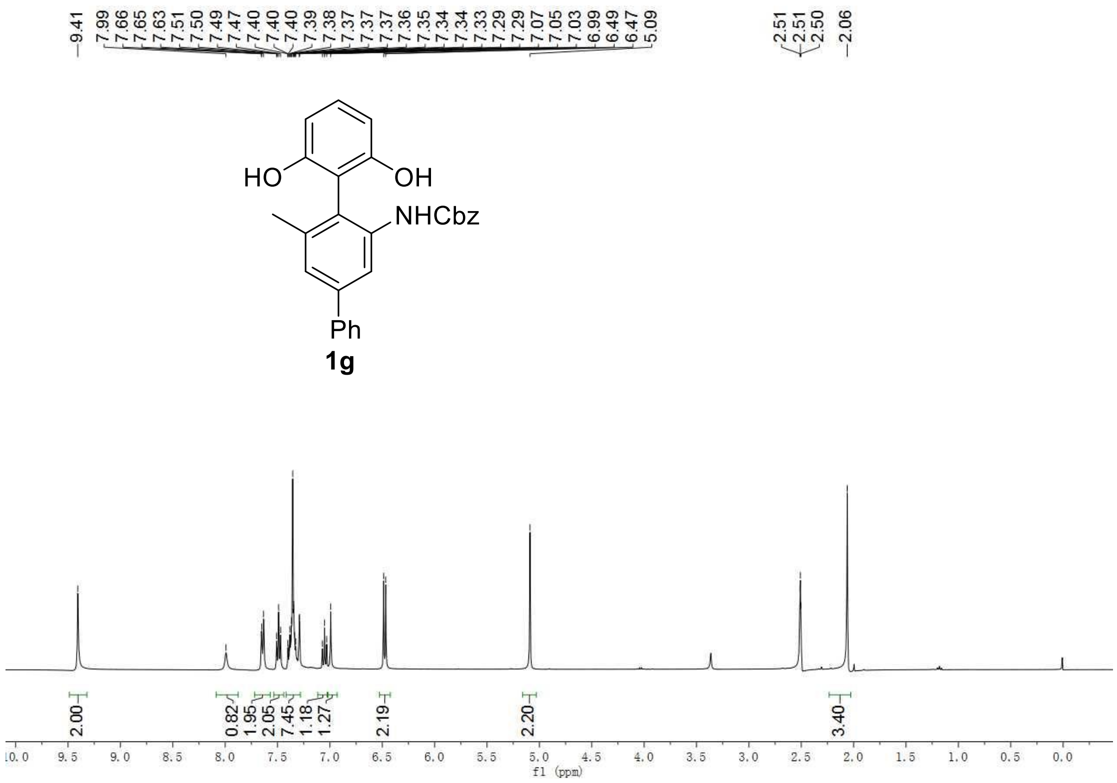

Supplementary Figure 162. ^{1}H NMR Spectrum of 1g.

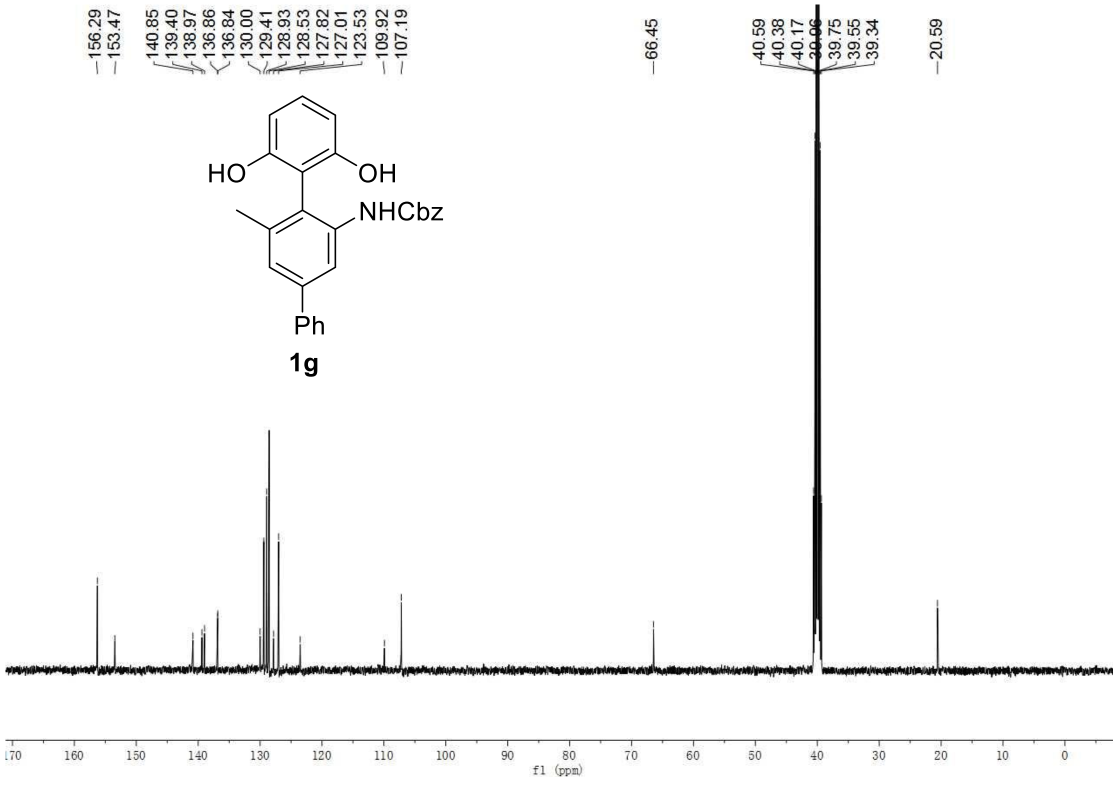

Supplementary Figure 163. ^{13}C NMR Spectrum of 1g.

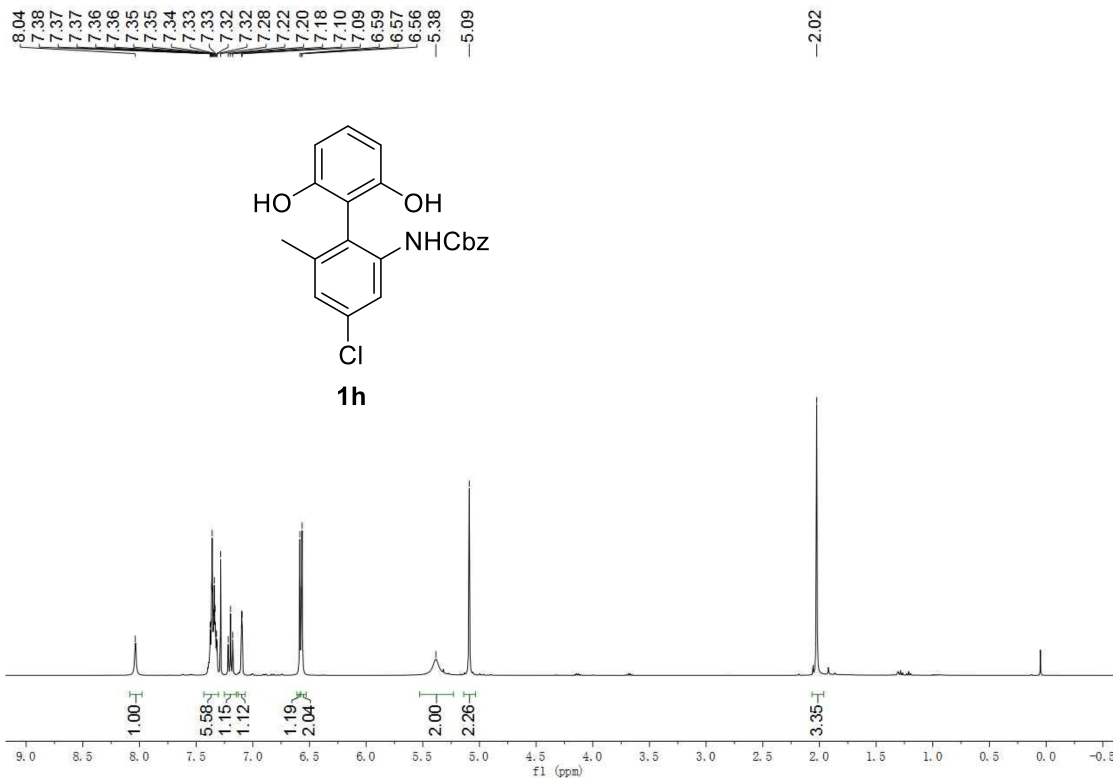

Supplementary Figure 164. ^{1}H NMR Spectrum of 1h.

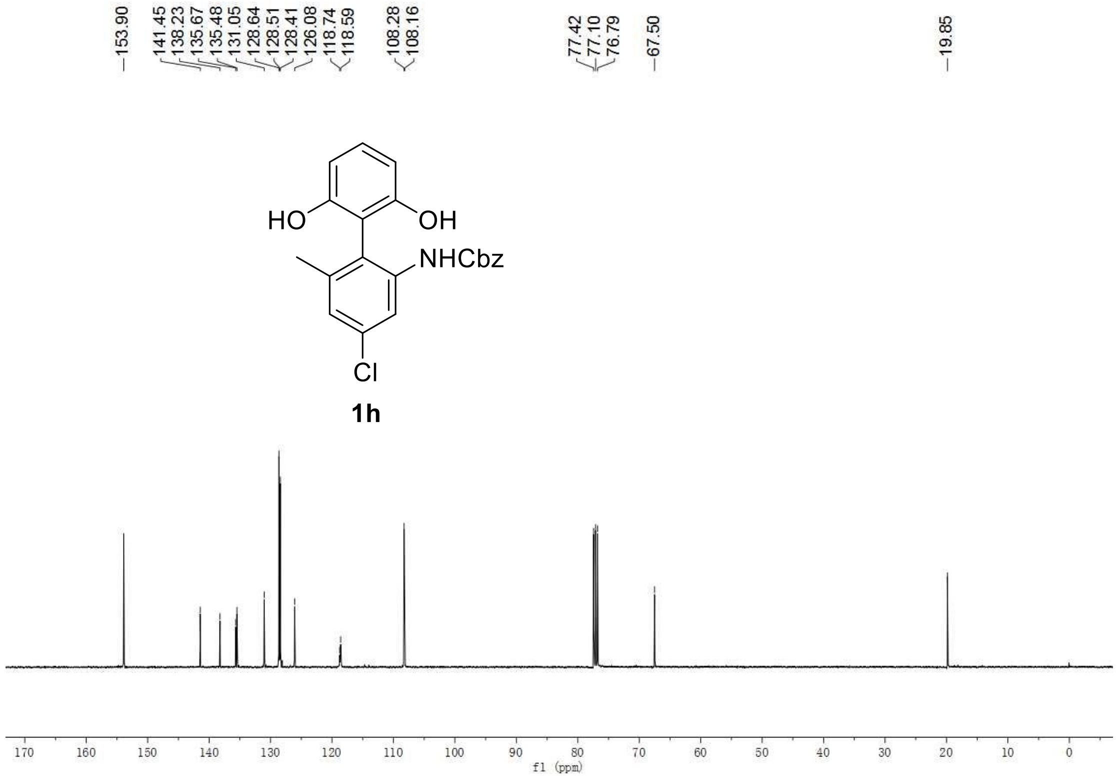

Supplementary Figure 165. ^{13}C NMR Spectrum of 1h.

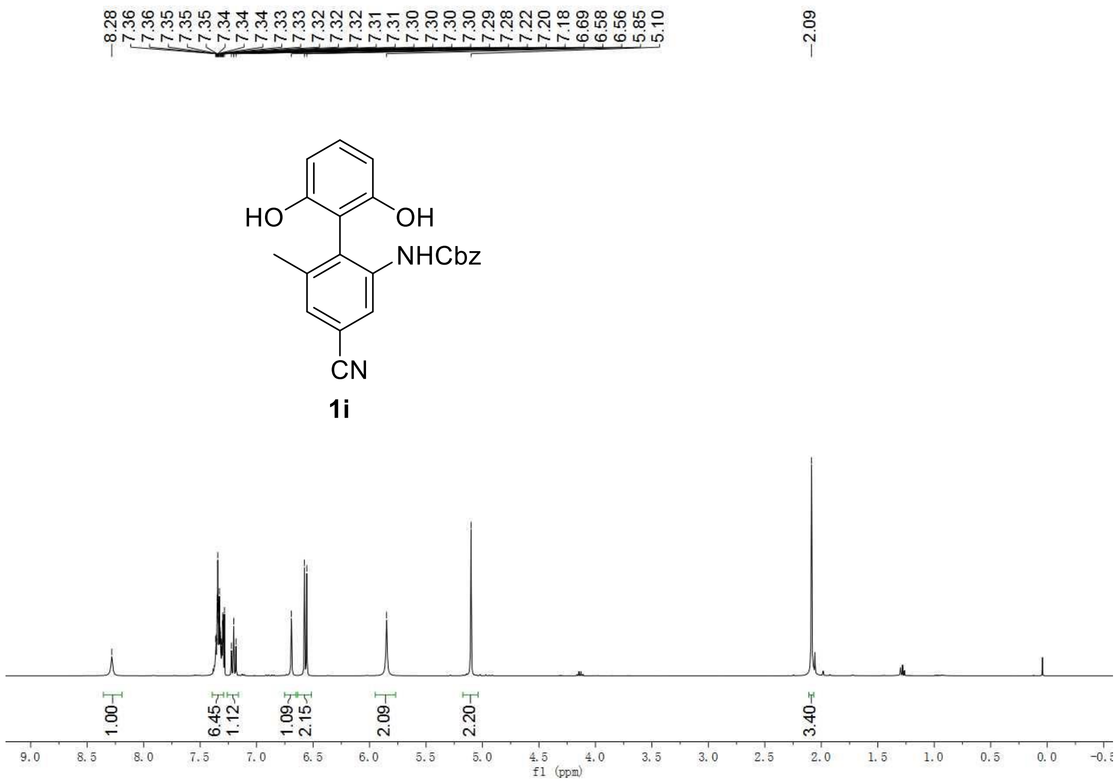

Supplementary Figure 166. ^{1}H NMR Spectrum of 1i.

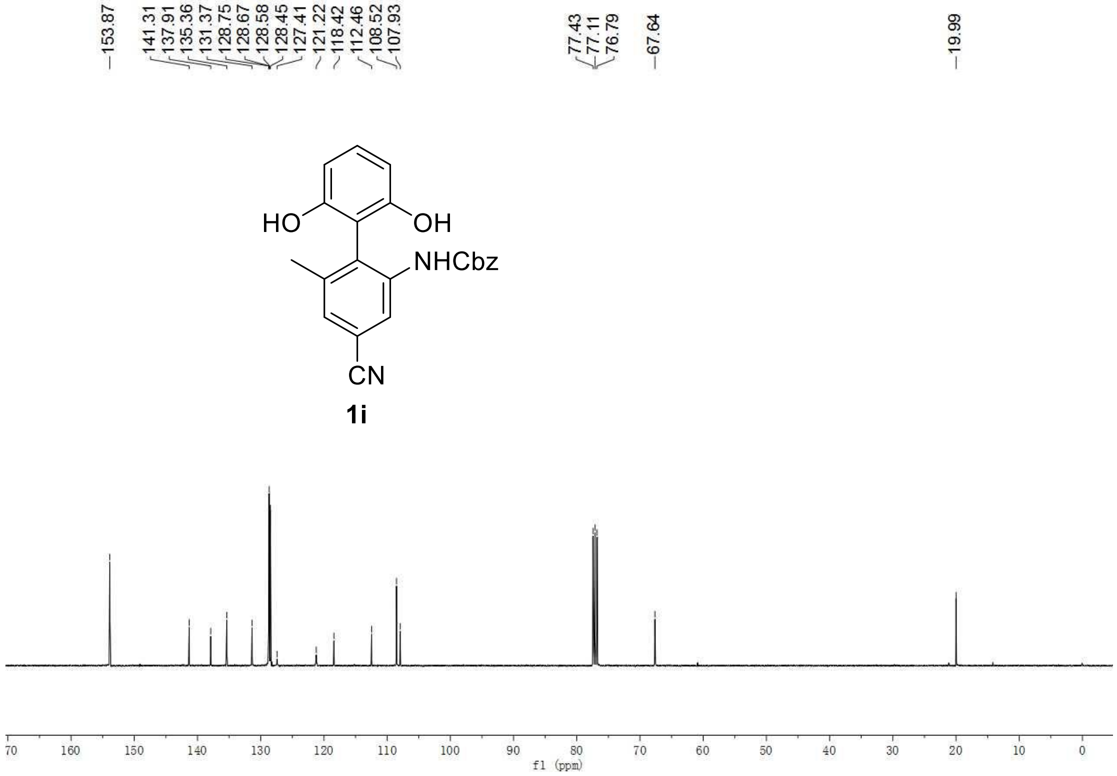

Supplementary Figure 167. ^{13}C NMR Spectrum of 1i.

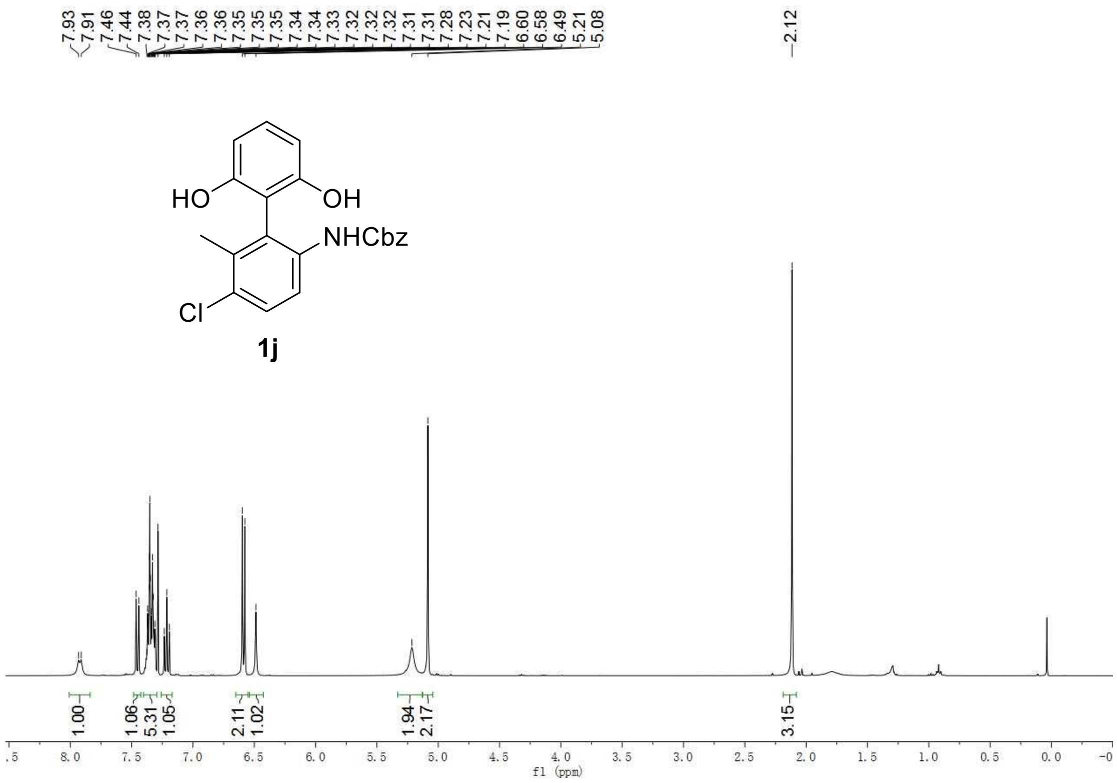

Supplementary Figure 168. ^{1}H NMR Spectrum of 1j.

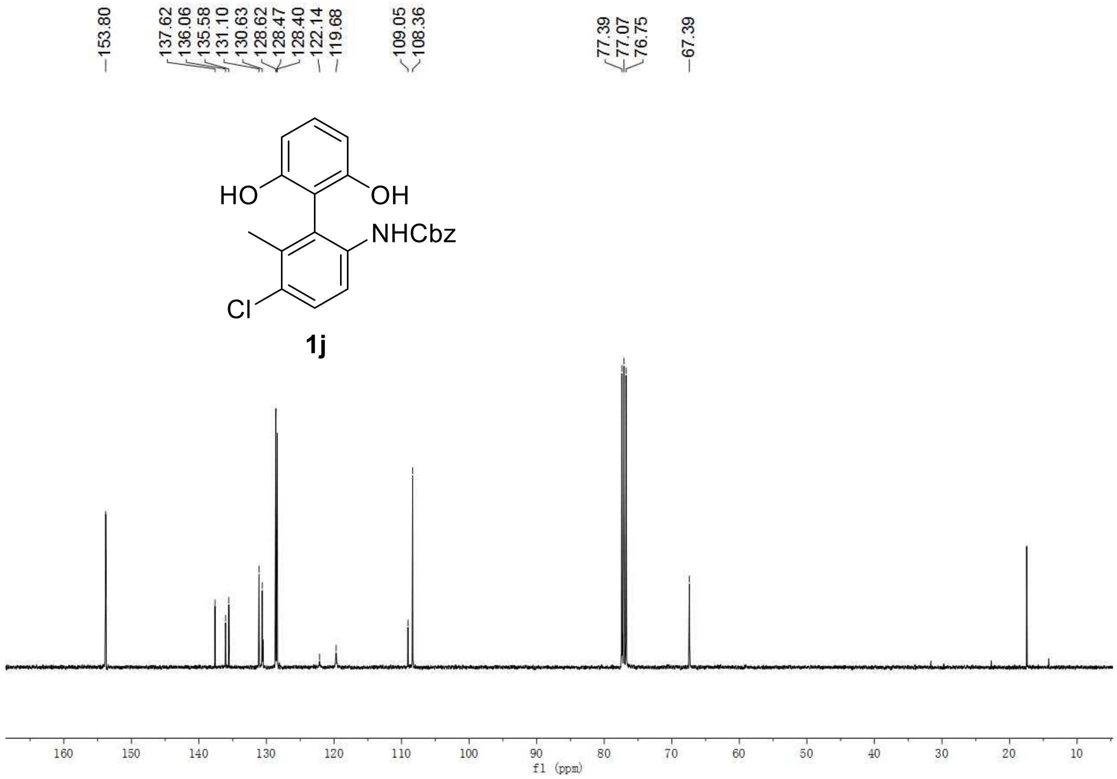

Supplementary Figure 169. ^{13}C NMR Spectrum of 1j.

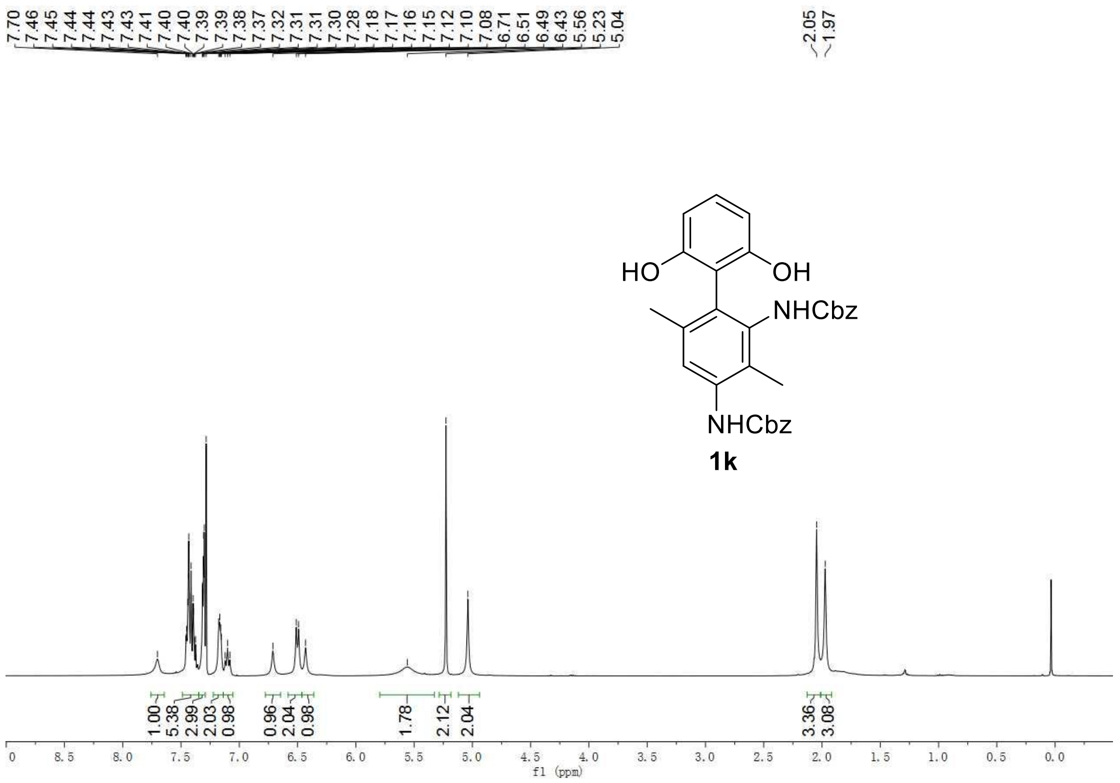

Supplementary Figure 170. ¹H NMR Spectrum of 1k.

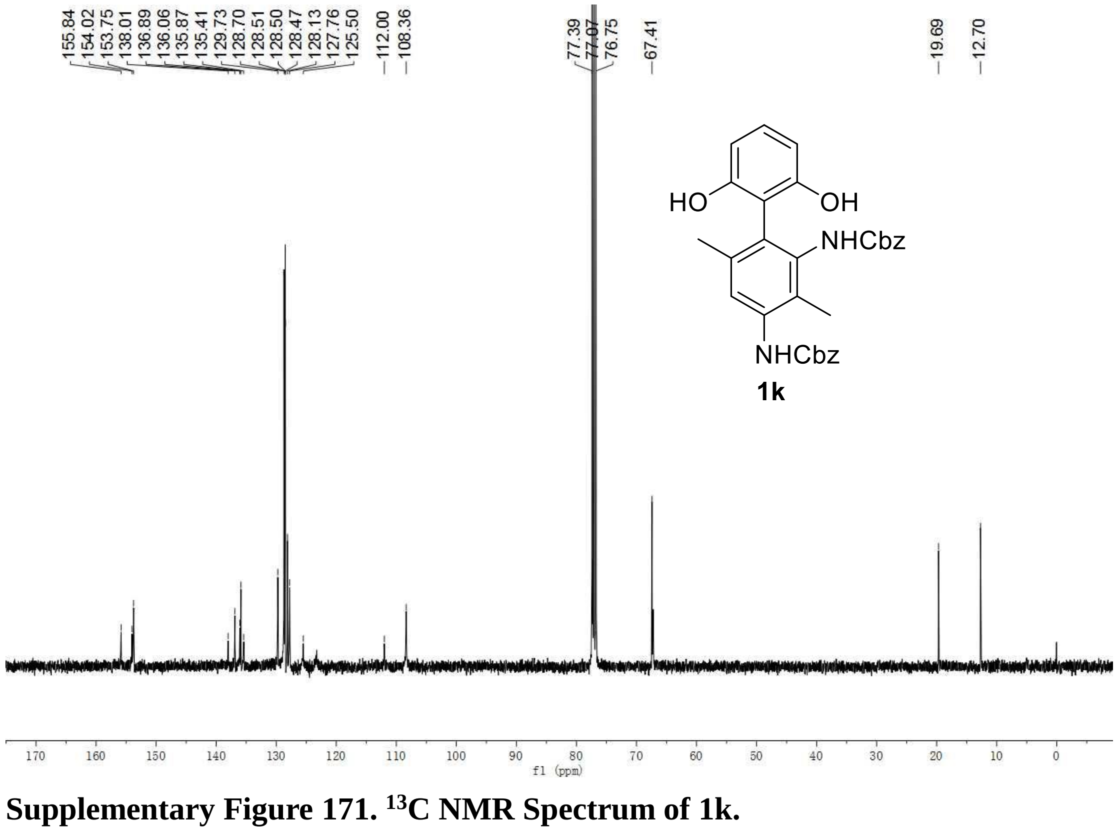

Supplementary Figure 171. ¹³C NMR Spectrum of 1k.

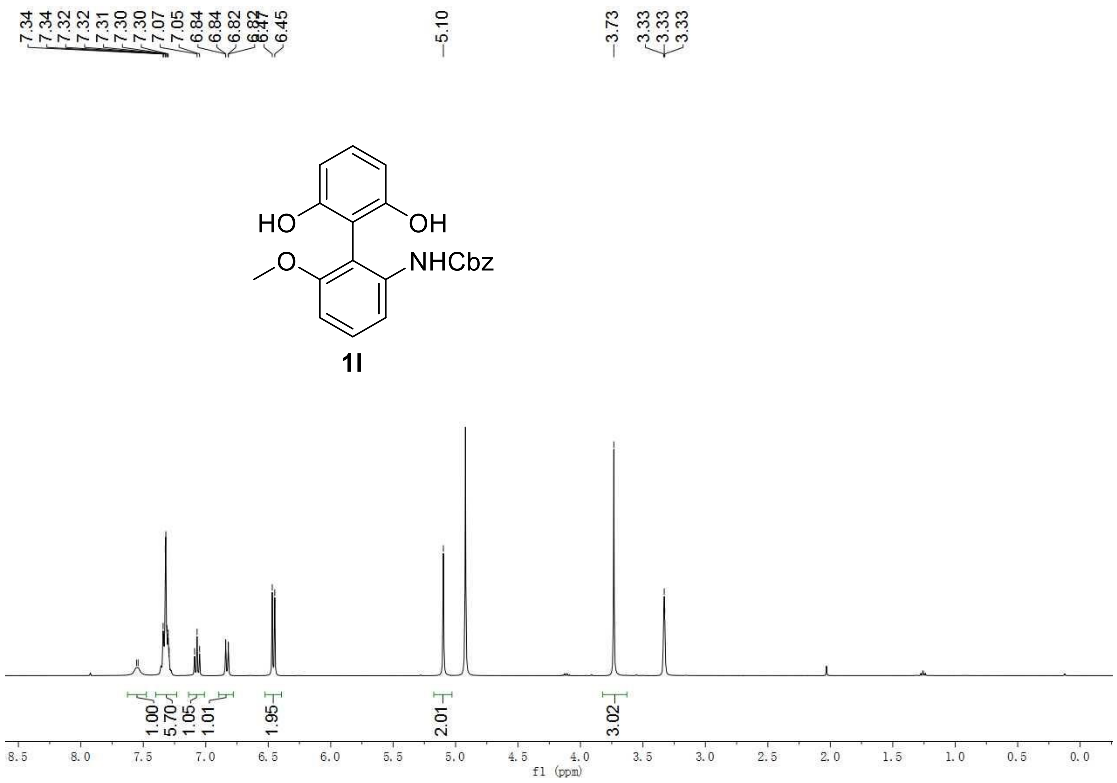

Supplementary Figure 172. ^{1}H NMR Spectrum of 1l.

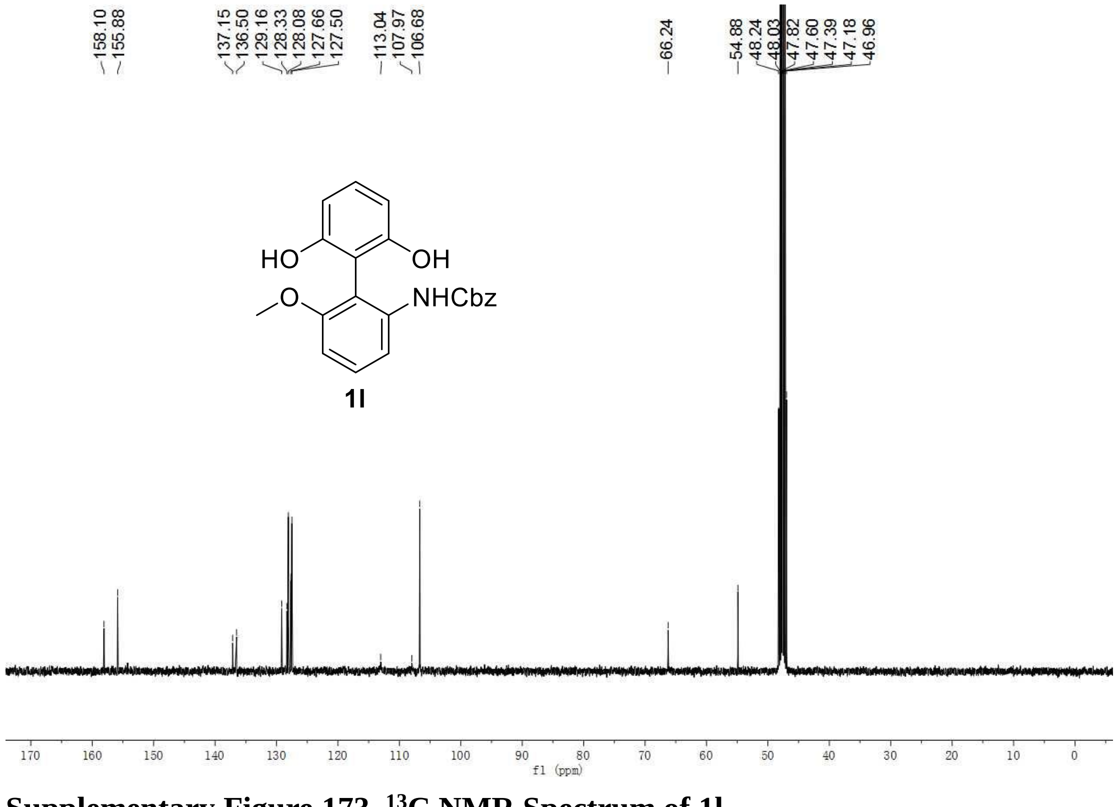

Supplementary Figure 173. ^{13}C NMR Spectrum of 1l.

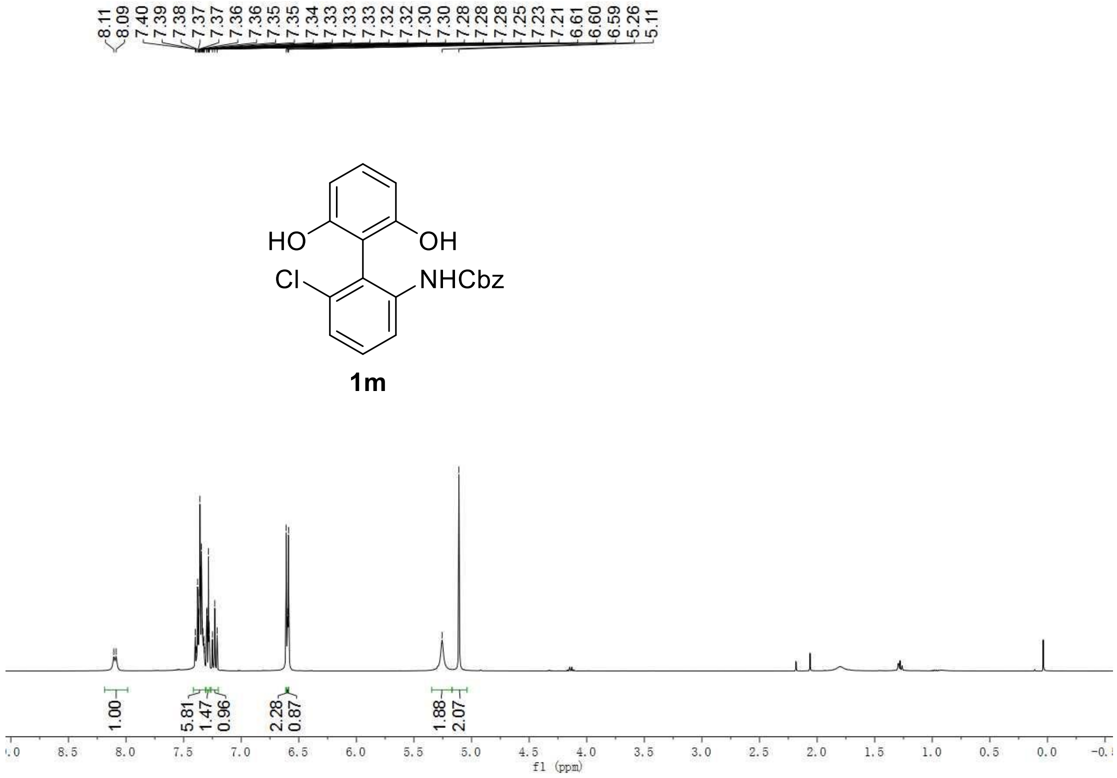

Supplementary Figure 174. ^{1}H NMR Spectrum of 1m.

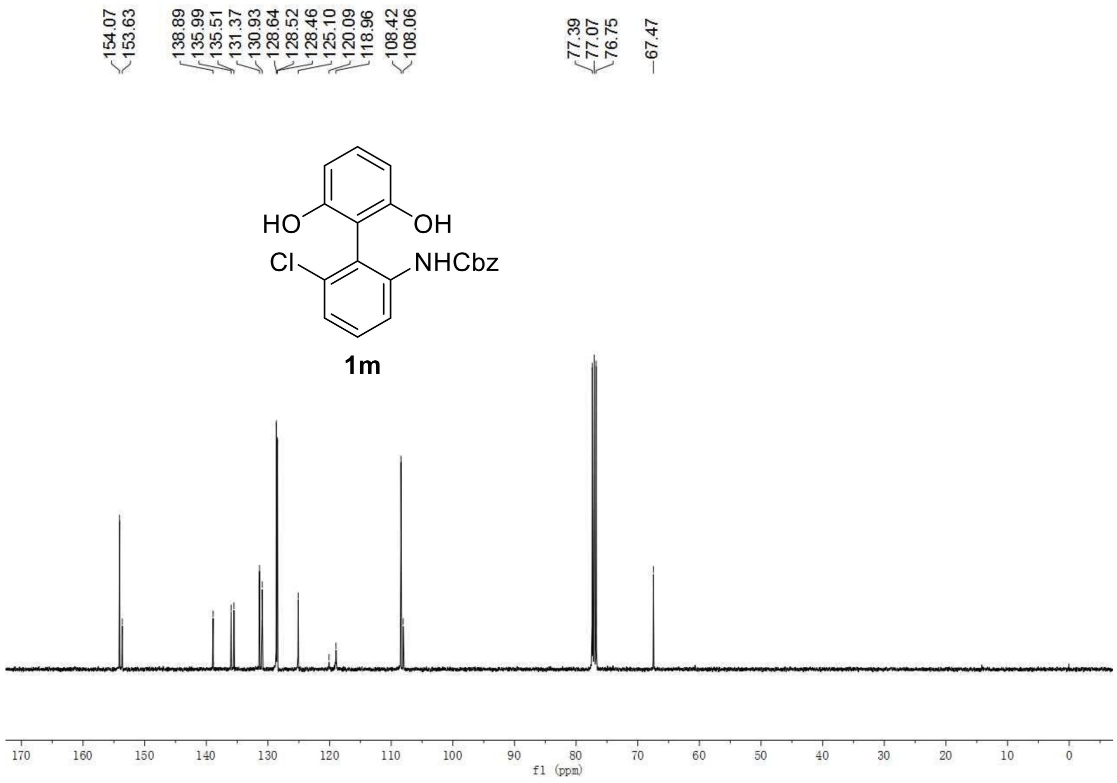

Supplementary Figure 175. ^{13}C NMR Spectrum of 1m.

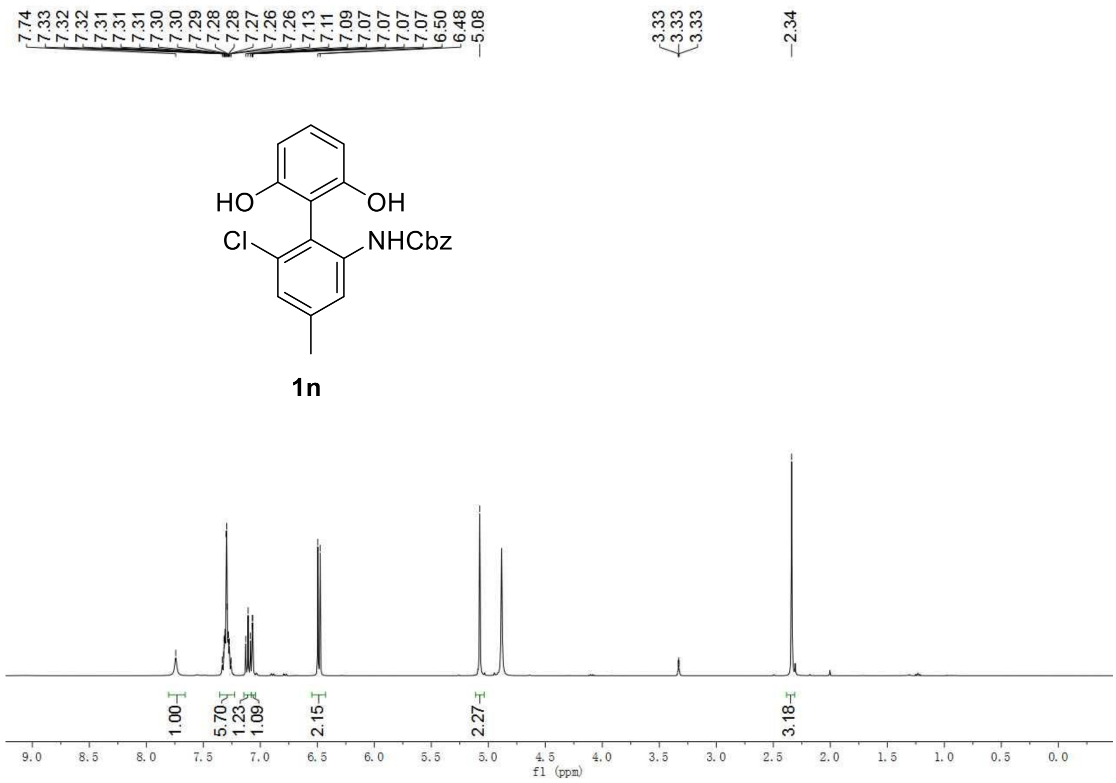

Supplementary Figure 176. ^{1}H NMR Spectrum of 1n.

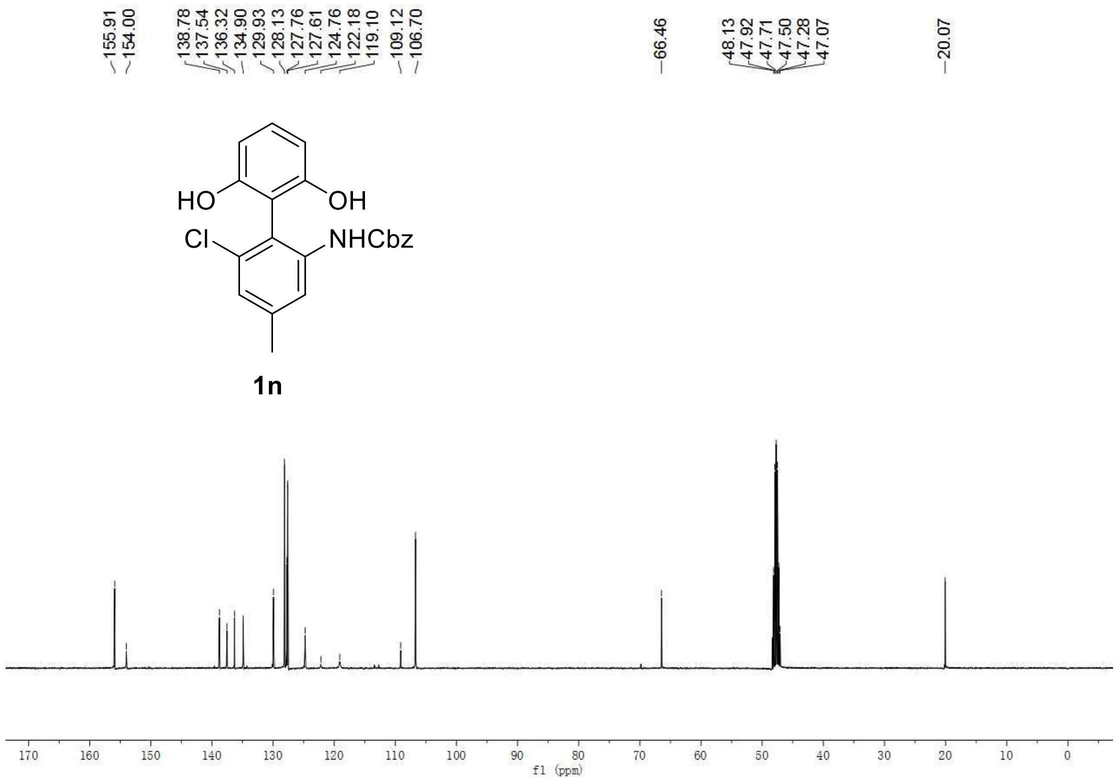

Supplementary Figure 177. ^{13}C NMR Spectrum of 1n.

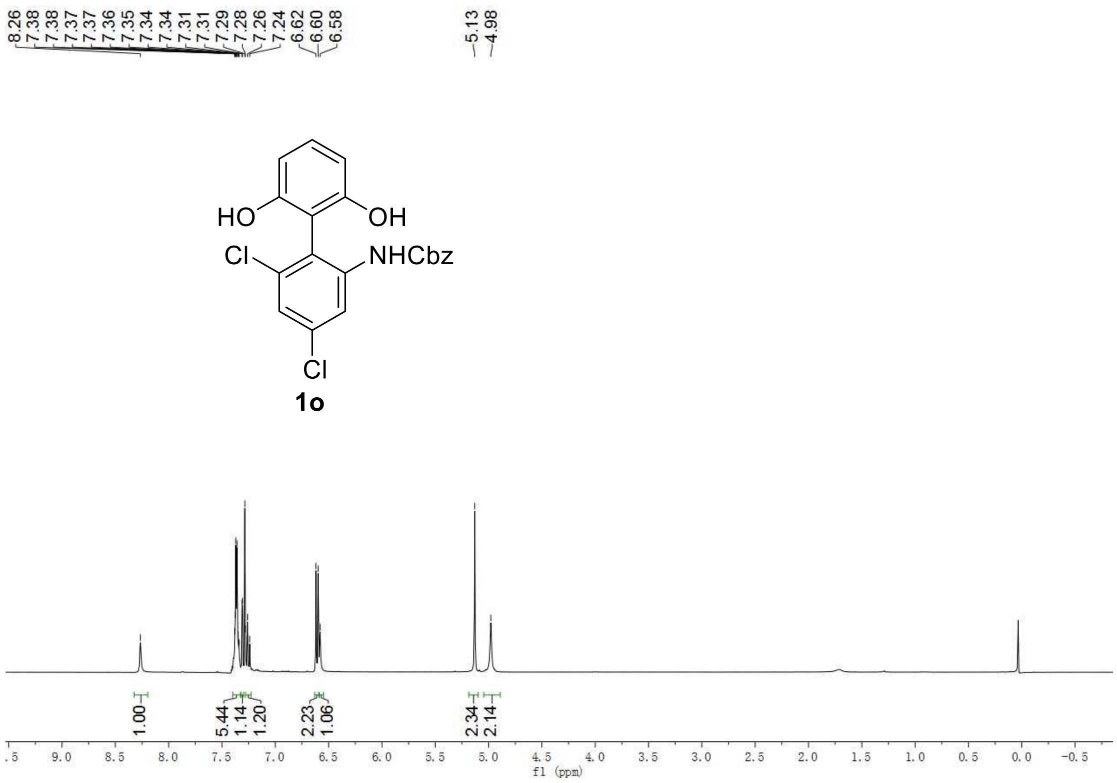

Supplementary Figure 178. ^{1}H NMR Spectrum of 1o.

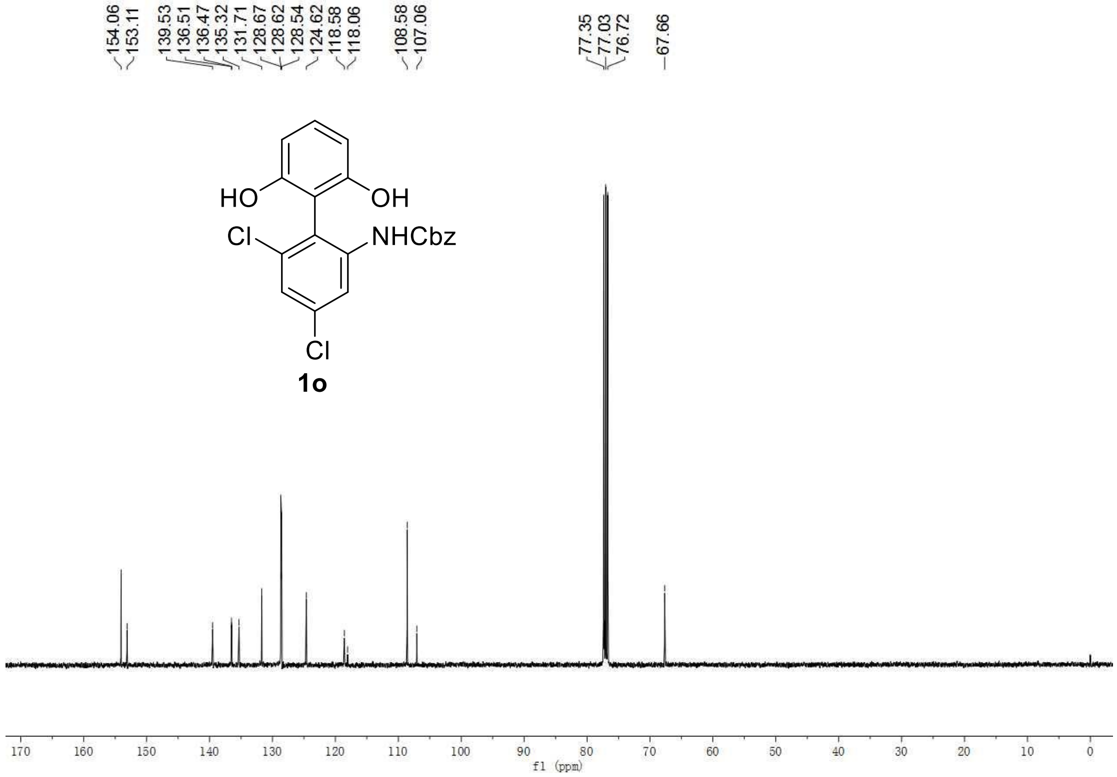

Supplementary Figure 179. ^{13}C NMR Spectrum of 1o.

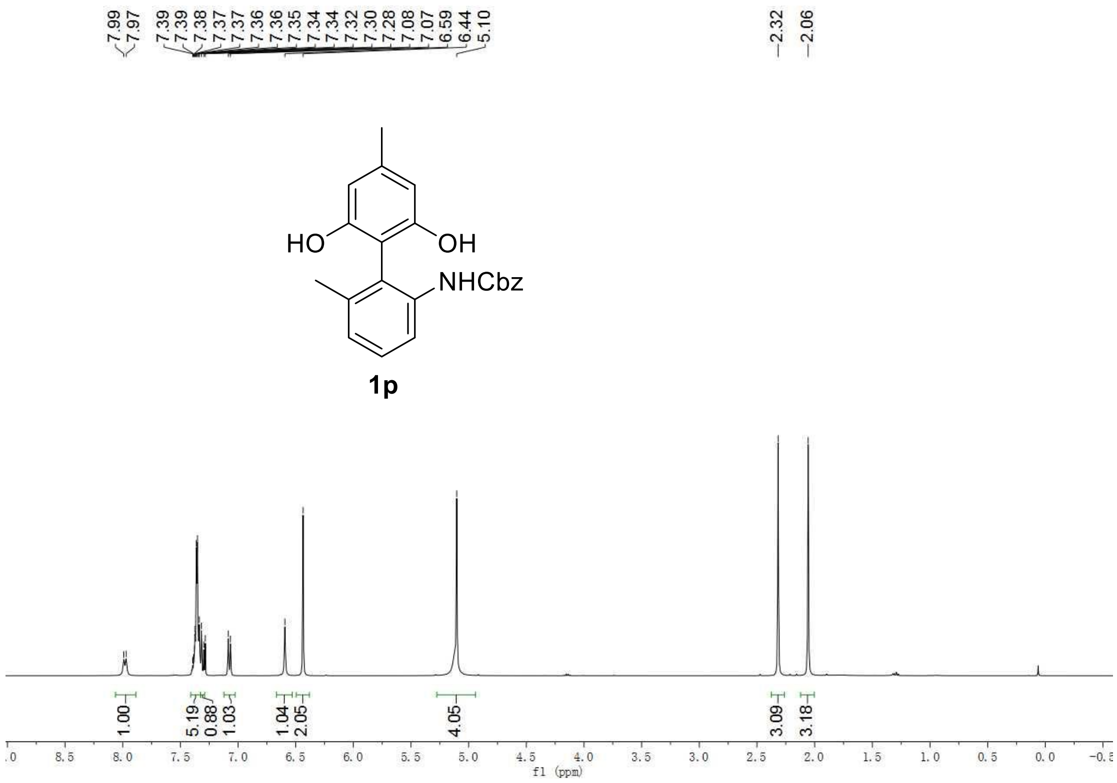

Supplementary Figure 180. ^{1}H NMR Spectrum of 1p.

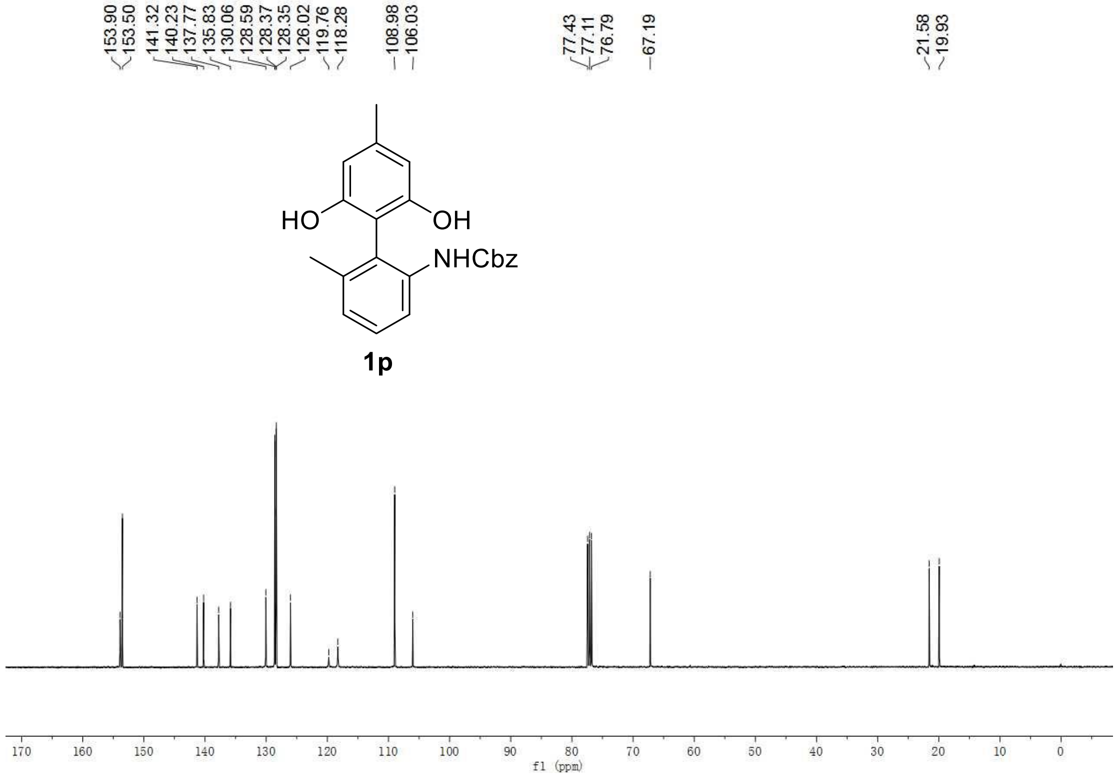

Supplementary Figure 181. ^{13}C NMR Spectrum of 1p.

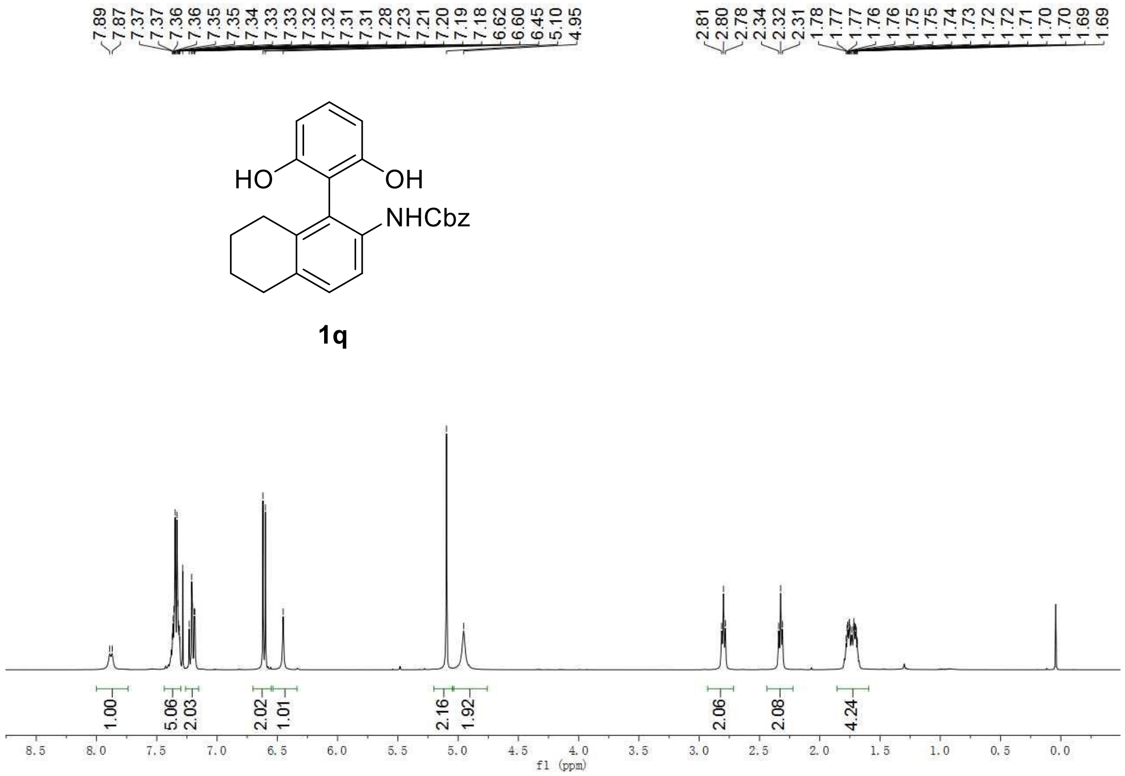

Supplementary Figure 182. ^{1}**H NMR Spectrum of 1q.**

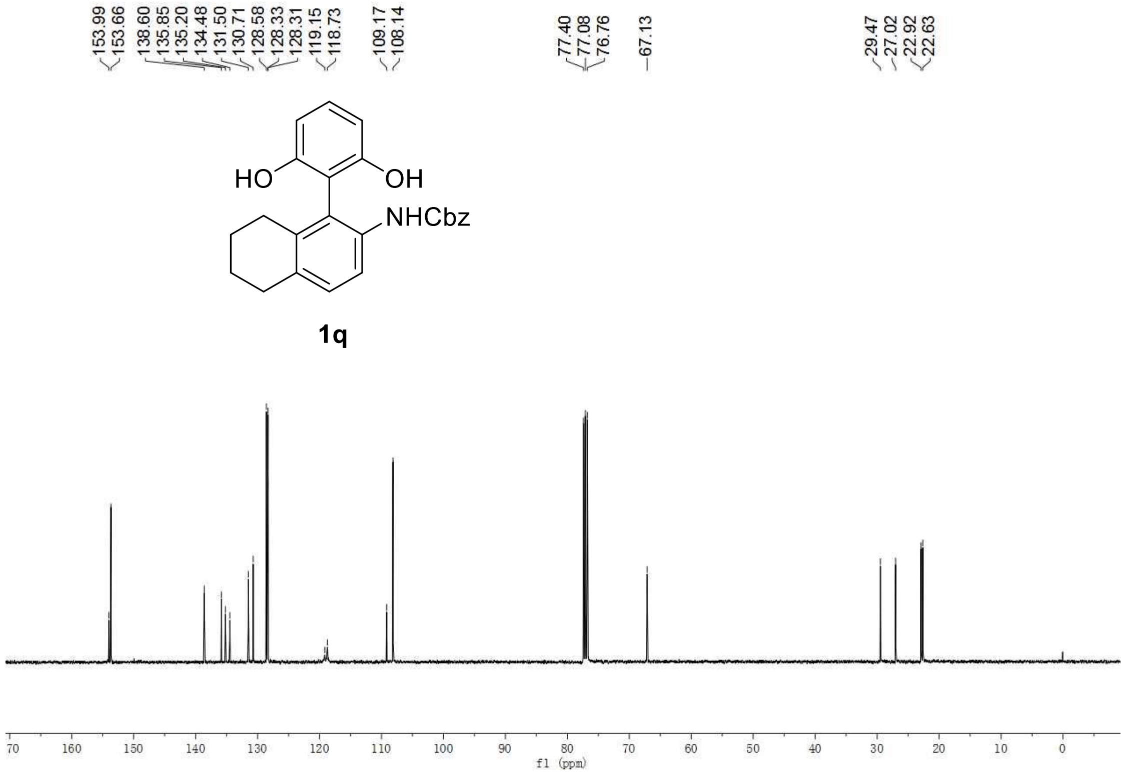

Supplementary Figure 183. ^{13}**C NMR Spectrum of 1q.**

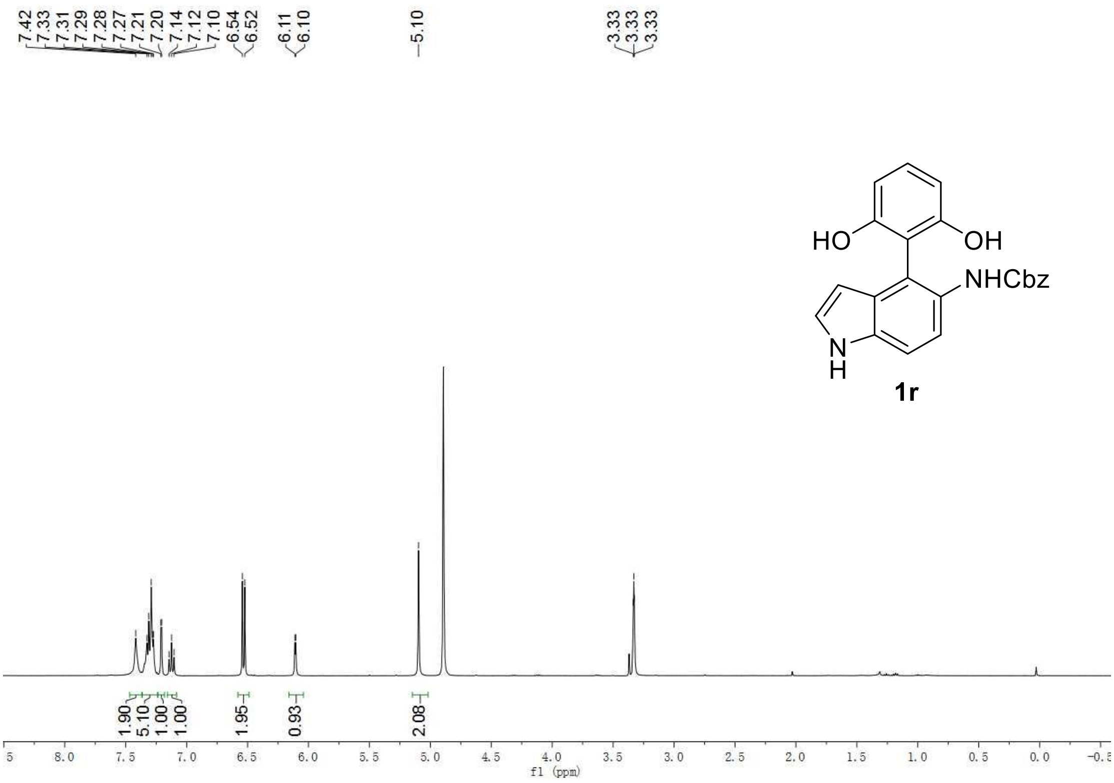

Supplementary Figure 184. ^{1}H NMR Spectrum of 1r.

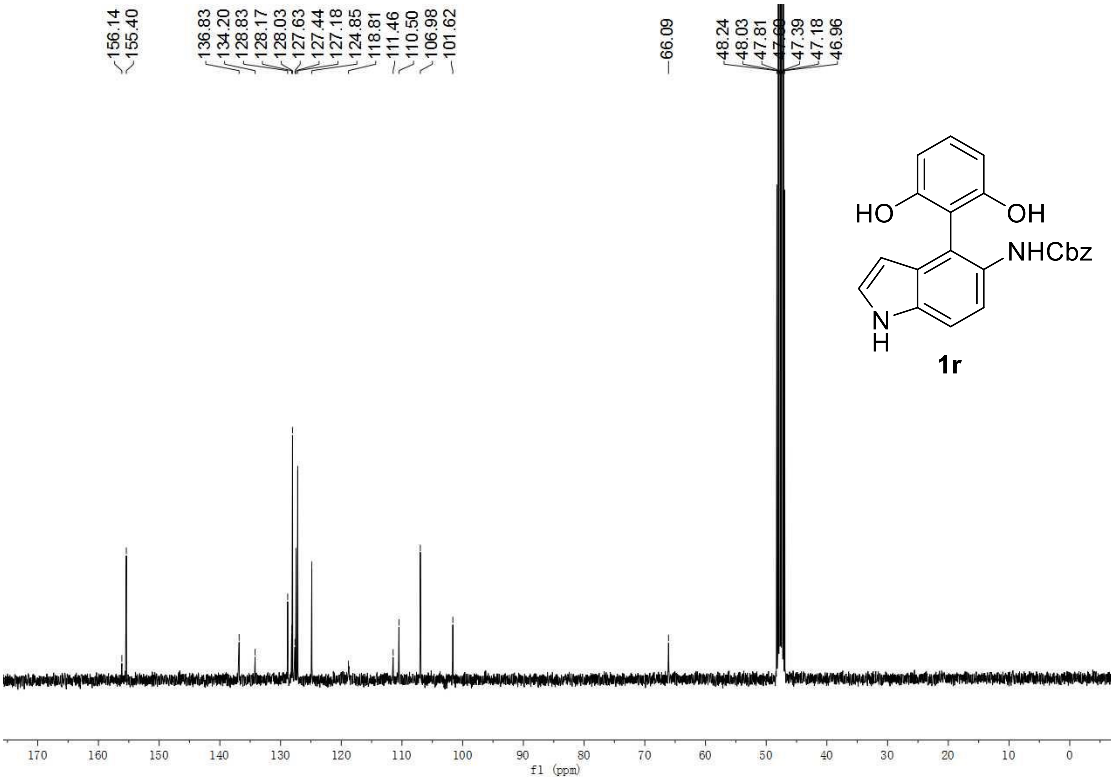

Supplementary Figure 185. ^{13}C NMR Spectrum of 1r.

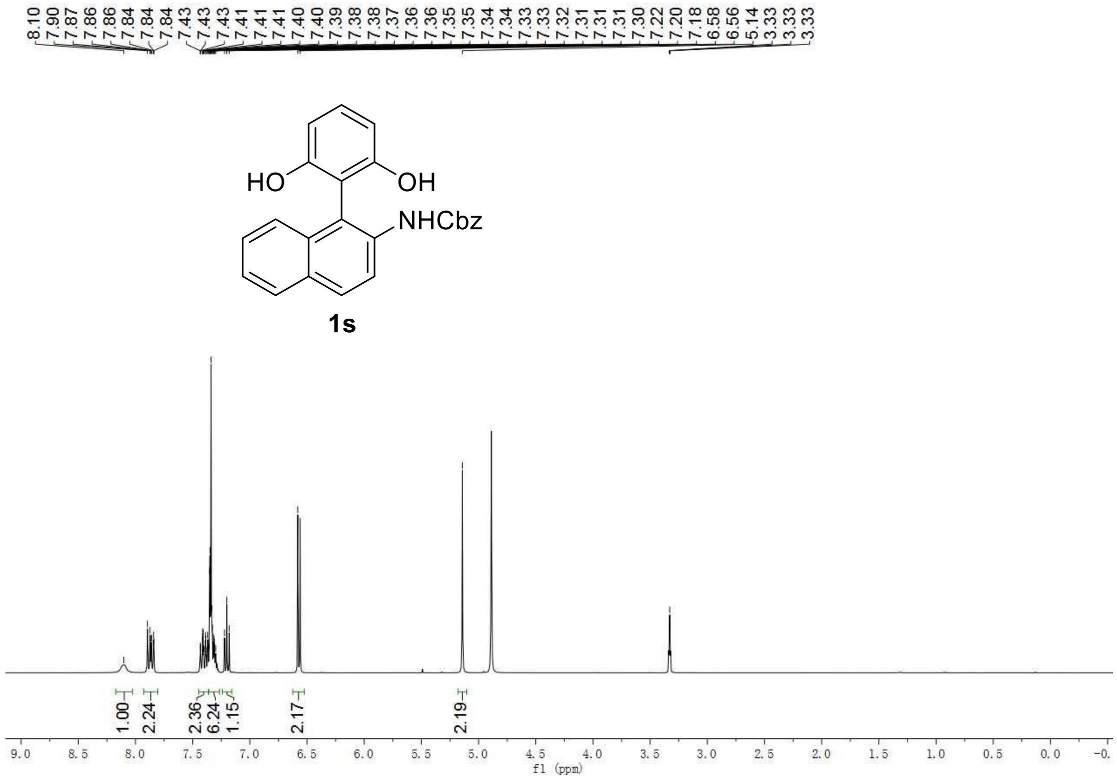

Supplementary Figure 186. ^{1}H NMR Spectrum of 1s.

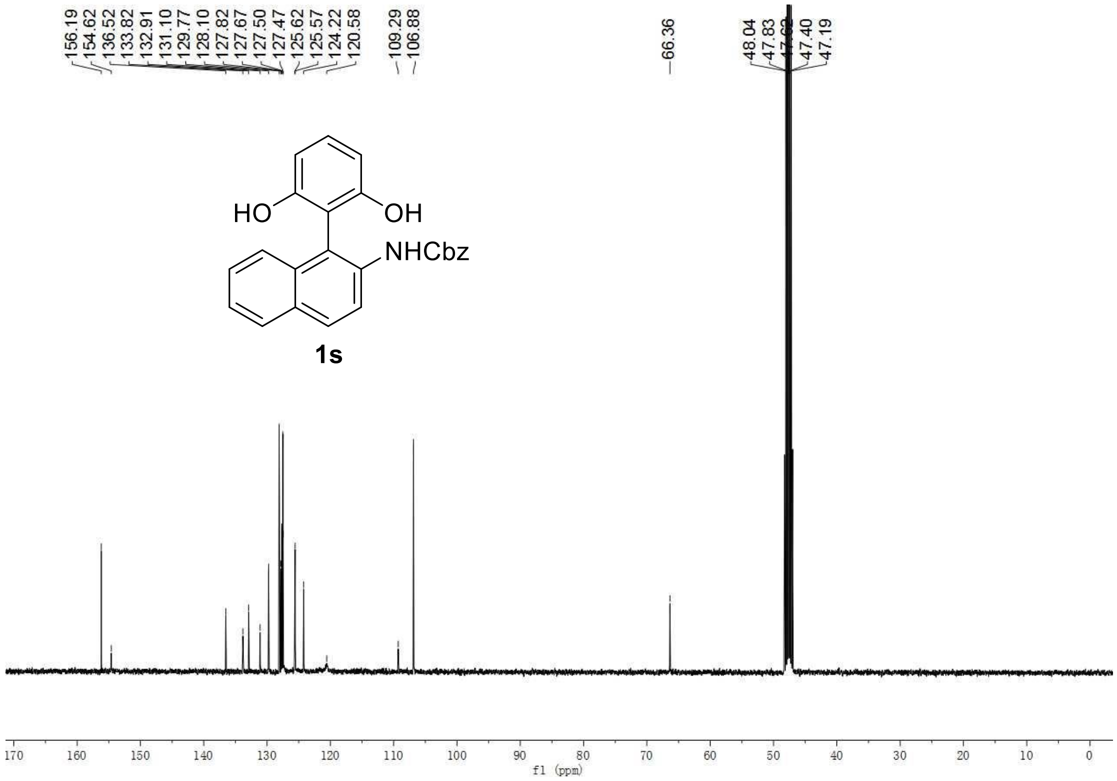

Supplementary Figure 187. ^{13}C NMR Spectrum of 1s.

Supplementary Tables

Supplementary Table 1. Crystal data and structure refinement for 6

Identification code	20181112yanggongming
Empirical formula	$C_{25}H_{21}NO_4$
Formula weight	399.43
Temperature/K	114.6(4)
Crystal system	monoclinic
Space group	$P2_1/c$
a/Å	11.8014(2)
b/Å	14.3726(2)
c/Å	12.4256(3)
α/°	90
β/°	111.048(2)
γ/°	90
Volume/Å^3	1966.97(7)
Z	4
ρ_{calc}g/cm^3	1.349
μ/mm^{-1}	0.742
F(000)	840.0
Crystal size/mm^3	0.3 × 0.25 × 0.22

Radiation	CuKα (λ = 1.54184)
2Θ range for data collection/°	8.028 to 142.824
Index ranges	-14 ≤ h ≤ 13, -12 ≤ k ≤ 17, -15 ≤ l ≤ 14
Reflections collected	12982
Independent reflections	3785 [R_{int} = 0.0275, R_{sigma} = 0.0247]
Data/restraints/parameters	3785/0/273
Goodness-of-fit on F^2	1.151
Final R indexes [I>=2σ (I)]	R_1 = 0.0592, wR_2 = 0.1447
Final R indexes [all data]	R_1 = 0.0627, wR_2 = 0.1472
Largest diff. peak/hole / e $Å^{-3}$	0.68/-0.34

Supplementary Table 2. Optimization of the reaction conditions

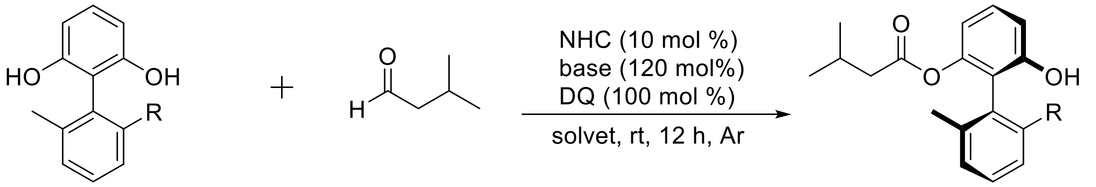

1 **2** **3**

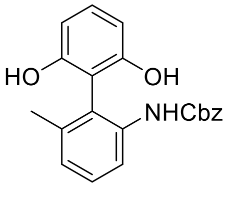

1a

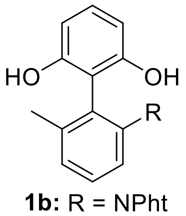

1b: R = NPht
1c: R = NHBoc

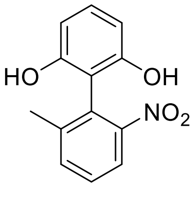

1d

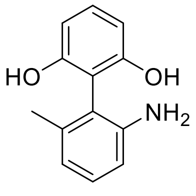

1e

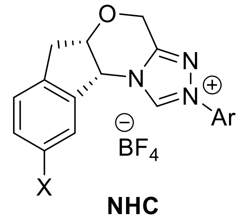

NHC

C1 Ar = 2,4,6-trimethylphenyl, X = H
C2 Ar = 2,4,6-trichlorophenyl, X = H
C3 Ar = C_6F_5, X = H
C4 Ar = 2,4,6-triisopropylphenyl, X = Br
C5 Ar = 2,4,6-triisopropylphenyl, X = NO_2

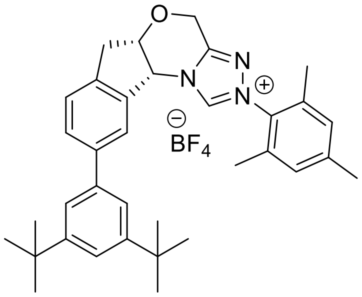

C6

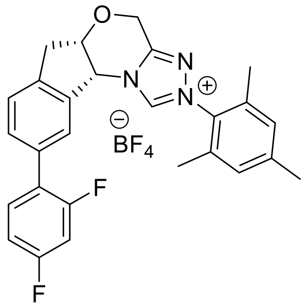

C7

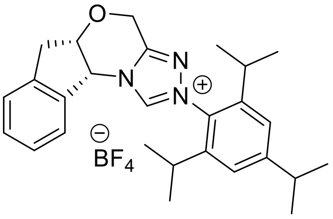

C8

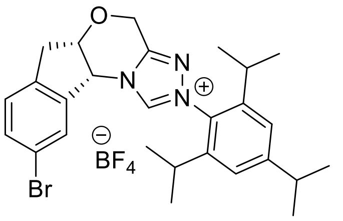

C9

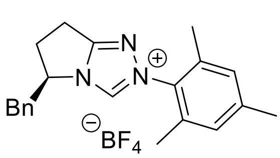

C10

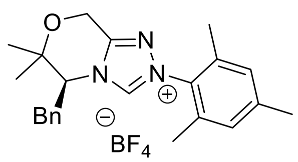

C11

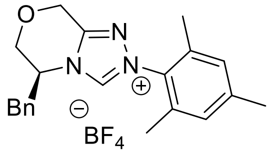

C12

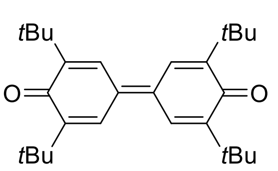

DQ

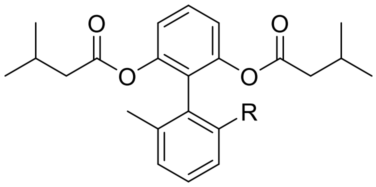

main byproduct

Entry[a]	NHC	Solvent	Base	Yield/%[b]	Ee/%[c]
1	**C1**	THF	K_2CO_3	63	85
2[d]	**C1**	THF	K_2CO_3	61	79
3[e]	**C1**	THF	K_2CO_3	50	93
4[f]	**C1**	THF	K_2CO_3	71	50
5[g]	**C1**	THF	K_2CO_3	mess.	--
6	**C2**	THF	K_2CO_3	23	23
7	**C3**	THF	K_2CO_3	< 5	--
8	**C4**	THF	K_2CO_3	74	90
9	**C5**	THF	K_2CO_3	80	95
10	**C6**	THF	K_2CO_3	59	67
11	**C7**	THF	K_2CO_3	70	67
12	**C8**	THF	K_2CO_3	70	90
13	**C9**	THF	K_2CO_3	74	90
14	**C10**	THF	K_2CO_3	70	- 89
15	**C11**	THF	K_2CO_3	46	- 63
16	**C12**	THF	K_2CO_3	70	- 77
17	**C5**	Tol.	K_2CO_3	90	96
18	**C5**	DCM	K_2CO_3	95	96
19	**C5**	MeCN	K_2CO_3	78	84
20	**C5**	MTBE	K_2CO_3	80	98
21	**C5**	Ether	K_2CO_3	81	96
22	**C5**	Dioxane	K_2CO_3	79	98
23	**C5**	$CHCl_3$	K_2CO_3	83	97
24	**C5**	Acetone	K_2CO_3	73	> 99
25	**C5**	DCM	Cs_2CO_3	85	92
26	**C5**	DCM	Na_2CO_3	39	96
27	**C5**	DCM	KOAc	39	96
28	**C5**	DCM	DBU	69	95
29	**C5**	DCM	Et_3N	76	97
30[h]	**C5**	DCM	K_2CO_3	78	93
31[i]	**C5**	DCM	K_2CO_3	92	99
32[j]	**C5**	DCM	K_2CO_3	87	96

[a] Conditions: **1a** (0.1 mmol), **2a** (0.15 mmol), catalyst (10 mol%), base (0.12 mmol) and DQ (0.1 mmol) in 1 mL of solvent. [b] Isolated yields after SiO_2 column chromatography. [c]

Enantiomeric ratio determined via chiral-phase HPLC analysis. [d] **1b** was use. [e] **1c** was used. [f] **1d** was used. [g] **1e** was used. [h] **2a** (0.12 mmol) was used, 15 h. [i] DQ (0.12 mmol) was used, 12 h. [j] **C5** (5 mol%) and DQ (0.12 mmol) was used, 24 h.

Supplementary Note 1

General Information

Chemicals and solvents were purchased from commercial suppliers and used as received. ^{1}H and ^{13}C NMR spectra were recorded on a Bruker ACF400 (400 MHz) spectrometer. Chemical shifts were reported in parts per million (ppm), and the residual solvent peak was used as an internal reference: proton (chloroform δ 7.26), carbon (chloroform δ 77.0) or tetramethylsilane (TMS δ 0.00) was used as a reference. Multiplicity was indicated as follows: s (singlet), d (doublet), t (triplet), q (quartet), m (multiplet), dd (doublet of doublet), bs (broad singlet). Coupling constants were reported in Hertz (Hz). Low resolution mass spectra were obtained on a Finnigan/MAT LCQ spectrometer in ESI mode, and a Finnigan/MAT 95XL-T mass spectrometer in EI mode. All high resolution mass spectra were obtained on a Finnigan/MAT 95XL-T spectrometer. For thin layer chromatography (TLC), Merck pre-coated TLC plates (Merck 60 F254) were used, and compounds were visualized with a UV light at 254 nm. Flash chromatography separations were performed on Merck 60 (0.040-0.063 mm) mesh silica gel. The enantiomeric excesses of products were determined by chiral phase HPLC analysis. Optical rotations were recorded on Jasco DIP-1000 polarimeter.

Supplementary Note 2

General procedure for the synthesis of 1a-s.[1-6] Preparation of 1a is shown as a representative example

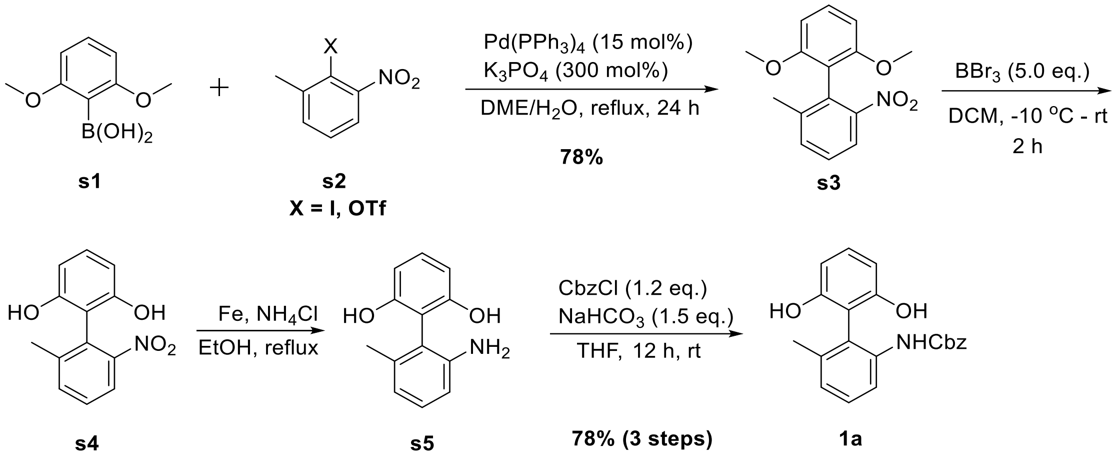

1a-p were synthesized from **s2** (X = I), **1q-s** were synthesized from **s2** (X = OTf).

Synthesis of 2,6-dimethoxy-2'-methyl-6'-nitro-1,1'-biphenyl (s3):[1-3]

The mixture of boronic acid **s1** (436.8 mg, 2.4 mmol), iodobenzene **s2** (526 mg, 2.0 mmol), $Pd(PPh_3)_4$ (346.7 mg, 0.3 mmol), K_3PO_4 (1.27 g, 6.0 mmol), DME (15.0 mL), and H_2O (5.0 mL) were heated at reflux for 24 h. After cooling to room temperature, the reaction was stopped by adding H_2O, The crude mixture was extracted with EtOAc (x3) and the combined organic extracts were washed with brine, dried (Na_2SO_4), and concentrated in vacuo. The residue was purified by column chromatography (silica gel, hexane/EtOAc = 10/1) to give **s3** (425.9 mg, 78%) as a yellow solid.

Synthesis of 2'-methyl-6'-nitro-[1,1'-biphenyl]-2,6-diol (s4):[4]

To a solution of 2'-methyl-6'-nitro-[1,1'-biphenyl]-2,6-diol **s3** (425.9 mg, 1.56 mmol, 1 equiv.) in CH_2Cl_2 (20 mL) at - 10 ºC, boron tribromide (7.8 mL, 1 M in CH_2Cl_2, 7.8 mmol) was added dropwise over 10 min via syringe. The reaction mixture was allowed to slowly warm to room temperature. After stirring for 2 h, methanol (1 mL) was added and the reaction was concentrated under reduced pressure to give the crude product. The product was purfied by column chromatography (silica gel, hexanes/EtOAc = 4/1) to give 2'-methyl-6'-nitro-[1,1'-biphenyl]-2,6-diol **s4**

(355.4 mg, 93%) as a colorless solid.

Synthesis of benzyl (2',6'-dihydroxy-6-methyl-[1,1'-biphenyl]-2-yl)carbamate (1a):[5-6]

To a solution of **s4** (355.4 mg , 1.45 mmol), iron powder (245 mg, 4.35 mmol), and NH_4Cl (155.2 mg, 2.9 mmol) in EtOH (15 mL) and H_2O (3 mL), and then the reaction mixture was heated to reflux for 2 h. After cooling to room temperature, the reaction mixture was filtered through Celite® pad and the resulting filtrate was concentrated in vacuo to give crude 2'-amino-6'-methyl-[1,1'-biphenyl]-2,6-diol **s5** (325.6 mg). The crude material was used for the next reaction without further purification.

To a solution of **s5** in dry THF (10 mL) were successively added $NaHCO_3$ (365.5 mg, 4.35 mmol) and CbzCl (297.5 mg, 1.74 mmol) at 0 °C. After being stirred for 12 h at room temperature, the reaction was stopped by adding water. The crude mixture was extracted with EtOAc (x3) and the combined organic extracts were washed with brine, dried ($NaSO_4$), and concentrated in vacuo. The residue was purified by column chromatography (silica gel, hexane/EtOAc = 3/1) to give **1a** (471.2 mg, 93% from **s4**) as a colorless oil.

Characterization data of the substrates

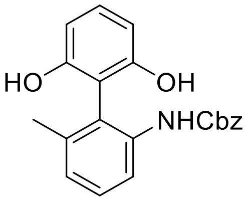

1a

1a: White solid; **^{1}H NMR** (400 MHz, Chloroform-*d*) δ 7.98 (d, *J* = 8.3 Hz, 1H), 7.35 (m, 6H), 7.21 (t, *J* = 8.2 Hz, 1H), 7.11 (d, *J* = 7.6 Hz, 1H), 6.60 (d, *J* = 8.2 Hz, 2H), 6.54 (s, 1H), 5.11 (s, 4H), 2.06 (s, 3H). **^{13}C NMR** (101 MHz, Chloroform-*d*) δ 153.80,

140.06, 137.60, 135.78, 130.80, 130.25, 128.58, 128.36, 128.32, 126.21, 118.59, 109.04, 108.15, 67.21, 19.87; **HRMS** (ESI): *m/z*: calculated for $C_{21}H_{19}NO_4$: [M + H]$^+$ 350.1392, found: 350.1394.

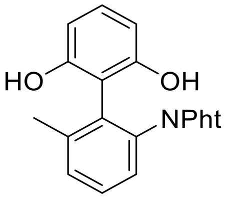

1b

1b: White solid; **^{1}H NMR** (400 MHz, Chloroform-*d*) δ 7.82 (dd, *J* = 5.5, 3.1 Hz, 2H), 7.71 (dd, *J* = 5.5, 3.1 Hz, 2H), 7.56 (d, *J* = 2.4 Hz, 1H), 7.55 (s, 1H), 7.29 (dd, *J* = 5.3, 3.9 Hz, 1H), 7.03 (t, *J* = 8.1 Hz, 2H), 6.51 (d, *J* = 8.2 Hz, 2H), 5.14 (s, 1H), 2.16 (s, 3H). **^{13}C NMR** (101 MHz, Chloroform-*d*) δ 167.78, 153.68, 141.90, 134.43, 132.49, 132.18, 131.50, 130.87, 130.30, 130.09, 127.30, 123.98, 112.08, 109.08, 19.80; **HRMS** (ESI): *m/z*: calculated for $C_{21}H_{17}NO_5$: [M + H]$^+$ 363.1107, found: 363.1110.

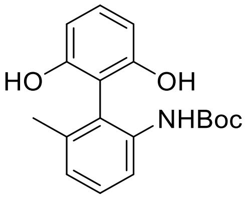

1c

1c: White solid; **^{1}H NMR** (400 MHz, Chloroform-*d*) δ 7.96 (d, *J* = 8.3 Hz, 1H), 7.36 (t, *J* = 7.9 Hz, 1H), 7.24 (t, *J* = 8.2 Hz, 1H), 7.10 (d, *J* = 7.6 Hz, 1H), 6.63 (d, *J* = 8.2 Hz, 2H), 6.27 (s, 1H), 4.97 (s, 2H), 2.05 (s, 3H), 1.45 (s, 9H). **^{13}C NMR** (101 MHz, Chloroform-*d*) δ 153.76, 153.36, 140.09, 138.23, 130.70, 130.37, 125.92, 119.13, 118.70, 109.34, 108.10, 81.04, 28.22, 19.90; **HRMS** (ESI): *m/z*: calculated for $C_{18}H_{22}NO_4$: [M + H]$^+$ 316.1471, found: 316.1473.

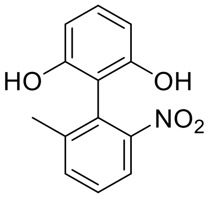

1d

1d: White solid; **^{1}H NMR** (400 MHz, Methanol-*d*$_4$) δ 7.85 – 7.67 (m, 1H), 7.56 (d, *J* = 7.5 Hz, 1H), 7.40 (t, *J* = 7.9 Hz, 1H), 7.03 (t, *J* = 8.1 Hz, 1H), 6.42 (d, *J* = 8.2 Hz,

2H), 2.18 (s, 3H). **^{13}C NMR** (101 MHz, Methanol-*d*$_4$) δ 154.92, 150.91, 140.72, 133.48, 129.47, 128.83, 127.19, 120.88, 111.30, 106.34, 18.76; **HRMS** (ESI): *m/z*: calculated for $C_{13}H_{12}NO_4$: $[M + H]^+$ 246.0766, found: 246.0761.

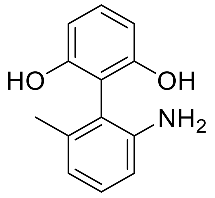

1e

1e: White solid; **^{1}H NMR** (400 MHz, Methanol-*d*$_4$) δ 7.09 – 7.02 (m, 2H), 6.71 (dd, *J* = 7.9, 2.2 Hz, 2H), 6.48 (d, *J* = 8.1 Hz, 2H), 2.02 (s, 3H). **^{13}C NMR** (101 MHz, Chloroform-*d*) δ 155.46, 145.14, 138.59, 128.73, 127.70, 119.91, 113.32, 106.77, 18.84; **HRMS** (ESI): *m/z*: calculated for $C_{13}H_{14}NO_2$: $[M + H]^+$ 216.0125, found: 216.0122.

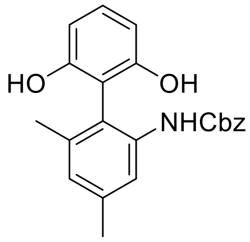

1f

1f: Colorless oil ; **^{1}H NMR** (400 MHz, Chloroform-*d*) δ 7.84 (s, 1H), 7.44 – 7.28 (m, 5H), 7.20 (t, *J* = 8.1 Hz, 1H), 6.94 (d, *J* = 1.6 Hz, 1H), 6.61 (d, *J* = 8.1 Hz, 2H), 6.51 (s, 1H), 5.22 (s, 2H), 5.09 (s, 2H), 2.37 (s, 3H), 2.03 (s, 3H). **^{13}C NMR** (101 MHz, Chloroform-*d*) δ 154.01, 153.86, 140.52, 139.69, 137.34, 135.79, 130.71, 128.60, 128.38, 128.33, 127.18, 108.95, 108.00, 67.18, 21.57, 19.81; **HRMS** (ESI): *m/z*: calculated for $C_{22}H_{22}NO_4$: $[M + H]^+$ 364.1549, found: 364.1546.

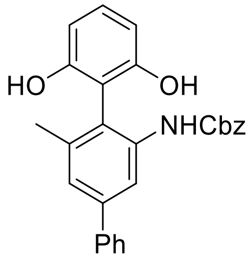

1g

1g: White solid; **^{1}H NMR** (400 MHz, DMSO-*d*$_6$) δ 9.41 (s, 2H), 7.99 (s, 1H), 7.64 (d,

J = 7.2 Hz, 2H), 7.49 (t, *J* = 7.6 Hz, 2H), 7.42 – 7.28 (m, 7H), 7.05 (t, *J* = 8.1 Hz, 1H), 6.99 (s, 1H), 6.48 (d, *J* = 8.1 Hz, 2H), 5.09 (s, 2H), 2.06 (s, 3H). **^{13}C NMR** (101 MHz, DMSO-d_6) δ 156.29, 153.47, 140.85, 139.40, 138.97, 136.86, 136.84, 130.00, 129.41, 128.93, 128.53, 127.82, 127.01, 123.53, 109.92, 107.19, 66.45, 20.59; **HRMS** (ESI): *m/z*: calculated for $C_{27}H_{24}NO_4$: $[M + H]^+$ 426.1705, found: 426.1707.

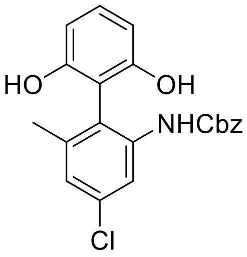

1h

1h: Colorless oil; **^{1}H NMR** (400 MHz, Chloroform-*d*) δ 8.04 (s, 1H), 7.43 – 7.30 (m, 5H), 7.20 (t, *J* = 8.2 Hz, 1H), 7.10 (d, *J* = 2.1 Hz, 1H), 6.59 (s, 1H), 6.57 (d, *J* = 2.2 Hz, 2H), 5.38 (s, 2H), 5.09 (s, 2H), 2.02 (s, 3H). **^{13}C NMR** (101 MHz, Chloroform-*d*) δ 153.90, 141.45, 138.23, 135.67, 135.48, 131.05, 128.64, 128.51, 128.41, 126.08, 118.74, 118.59, 108.28, 108.16, 67.50, 19.85; **HRMS** (ESI): *m/z*: calculated for $C_{21}H_{19}ClNO_4$: $[M + H]^+$ 384.1003, found: 384.1001.

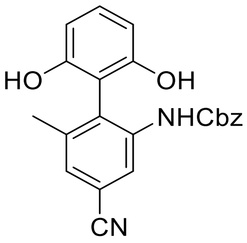

1i

1i: White solid; **^{1}H NMR** (400 MHz, Chloroform-*d*) δ 8.28 (s, 1H), 7.36 – 7.29 (m, 6H), 7.20 (t, *J* = 8.2 Hz, 1H), 6.69 (s, 1H), 6.57 (d, *J* = 8.2 Hz, 2H), 5.85 (s, 2H), 5.10 (s, 2H), 2.09 (s, 3H). **^{13}C NMR** (101 MHz, Chloroform-*d*) δ 153.87, 141.31, 137.91, 135.36, 131.37, 128.75, 128.67, 128.58, 128.45, 118.42, 112.46, 108.52, 107.93, 67.64, 19.99; **HRMS** (ESI): *m/z*: calculated for $C_{22}H_{19}N_2O_4$: $[M + H]^+$ 375.1345, found: 375.1343.

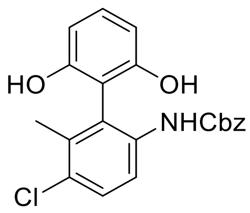

1j

1j: Colorless oil; **^{1}H NMR** (400 MHz, Chloroform-*d*) δ 7.92 (d, *J* = 8.8 Hz, 1H), 7.45 (d, *J* = 8.9 Hz, 1H), 7.40 – 7.29 (m, 5H), 7.21 (t, *J* = 8.2 Hz, 1H), 6.59 (d, *J* = 8.2 Hz, 2H), 6.49 (s, 1H), 5.21 (s, 2H), 5.08 (s, 2H), 2.12 (s, 3H). **^{13}C NMR** (101 MHz, Chloroform-*d*) δ 153.80, 137.62, 136.06, 135.58, 131.10, 130.63, 128.62, 128.47, 128.40, 109.05, 108.36, 67.39; **HRMS** (ESI): *m/z*: calculated for $C_{21}H_{19}ClNO_4$: [M + H]$^+$ 384.1003, found: 384.1006.

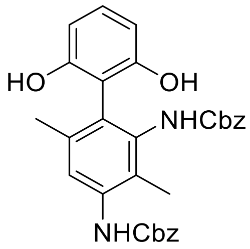

1k

1k: Colorless oil; **^{1}H NMR** (400 MHz, Chloroform-*d*) δ 7.70 (s, 1H), 7.46 – 7.37 (m, 5H), 7.32 – 7.28 (m, 3H), 7.18 – 7.15 (m, 2H), 7.10 (t, *J* = 8.1 Hz, 1H), 6.71 (s, 1H), 6.50 (d, *J* = 8.1 Hz, 2H), 6.43 (s, 1H), 5.56 (s, 2H), 5.23 (s, 2H), 5.04 (s, 2H), 2.05 (s, 3H), 1.97 (s, 3H). **^{13}C NMR** (101 MHz, Chloroform-*d*) δ 155.84, 154.02, 153.75, 138.01, 136.89, 136.06, 135.87, 135.41, 129.73, 128.70, 128.51, 128.50, 128.47, 128.13, 127.76, 125.50, 112.00, 108.36, 67.41, 19.69, 12.70; **HRMS** (ESI): *m/z*: calculated for $C_{30}H_{29}N_2O_6$: [M + H]$^+$ 513.2026, found: 513.2024.

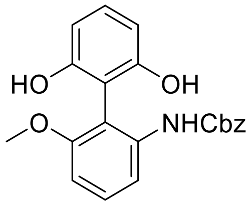

1l

1l: White solid; **^{1}H NMR** (400 MHz, Methanol-*d*$_4$) δ 7.55 (d, *J* = 8.1 Hz, 1H), 7.40 – 7.23 (m, 6H), 7.07 (t, *J* = 8.1 Hz, 1H), 7.64 (dd, *J* = 8.2 Hz, 1H), 6.46 (d, *J* = 8.2 Hz, 2H), 5.10 (s, 2H), 3.73 (s, 3H). **^{13}C NMR** (101 MHz, Methanol-*d*$_4$) δ 156.10, 155.88,

137.15, 136.50, 129.16, 128.33, 128.08, 127.66, 127.50, 113.04, 107.97, 106.68, 66.24, 54.88; **HRMS** (ESI): *m/z*: calculated for $C_{21}H_{20}NO_5$: $[M + H]^+$ 366.1341, found: 366.1342.

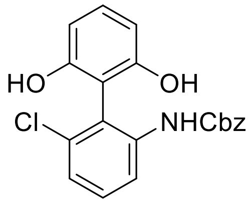

1m

1m: Colorless oil; **^{1}H NMR** (400 MHz, Chloroform-*d*) δ 8.10 (d, *J* = 8.3 Hz, 1H), 7.42 – 7.31 (m, 6H), 7.30 (dd, *J* = 8.2 Hz, 1H), 7.23 (t, *J* = 8.2 Hz, 1H), 6.61 (d, *J* = 8.2 Hz, 2H), 6.60 (s, 1H), 5.26 (s, 2H), 5.11 (s, 2H). **^{13}C NMR** (101 MHz, Chloroform-*d*) δ 154.07, 153.63, 138.89, 135.99, 135.51, 131.37, 130.93, 128.64, 128.52, 128.46, 125.10, 108.42, 108.06, 67.47; **HRMS** (ESI): *m/z*: calculated for $C_{20}H_{17}ClNO_4$: $[M + H]^+$ 370.0846, found: 370.0842.

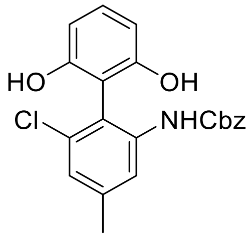

1n

1n: White solid; **^{1}H NMR** (400 MHz, Methanol-*d*$_4$) δ 7.74 (s, 1H), 7.36 – 7.23 (m, 6H), 7.11 (t, *J* = 8.2 Hz, 1H), 7.07 (dd, *J* = 1.7, 0.8 Hz, 1H), 6.49 (d, *J* = 8.2 Hz, 2H), 5.08 (s, 2H), 2.34 (s, 3H). **^{13}C NMR** (101 MHz, Methanol-*d*$_4$) δ 155.91, 154.00, 138.78, 137.54, 136.32, 134.90, 129.93, 128.13, 127.76, 127.61, 124.76, 109.12, 106.70, 66.46, 20.07; **HRMS** (ESI): *m/z*: calculated for $C_{21}H_{19}ClNO_4$: $[M + H]^+$ 384.1003, found: 384.1007.

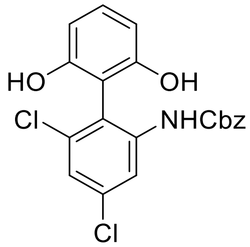

1o

1o: Colorless oil; **^{1}H NMR** (400 MHz, Chloroform-*d*) δ 8.26 (s, 1H), 7.37 (dd, *J* = 4.9,

3.5 Hz, 5H), 7.31 (d, *J* = 2.0 Hz, 1H), 7.28 (t, *J* = 8.2 Hz, 1H), 6.61 (d, *J* = 8.2 Hz, 2H), 6.58 (s, 1H), 5.13 (s, 2H), 4.98 (s, 2H). **^{13}C NMR** (101 MHz, Chloroform-*d*) δ 154.06, 153.11, 139.53, 136.51, 136.47, 135.32, 131.71, 128.67, 128.62, 128.54, 124.62, 118.58, 118.06, 108.58, 107.06, 67.66; **HRMS** (ESI): *m/z*: calculated for $C_{20}H_{16}Cl_2NO_4$: $[M + H]^+$ 404.0456, found: 404.0451.

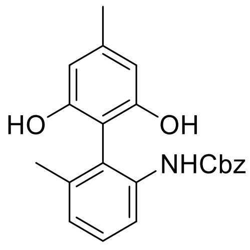

1p

1p: Colorless oil; **^{1}H NMR** (400 MHz, Chloroform-*d*) δ 7.98 (d, *J* = 8.2 Hz, 1H), 7.41 – 7.32 (m, 5H), 7.31 (d, *J* = 8.0 Hz, 1H), 7.08 (d, *J* = 7.6 Hz, 1H), 6.59 (s, 1H), 6.44 (s, 2H), 5.10 (s, 4H), 2.32 (s, 3H), 2.06 (s, 3H). **^{13}C NMR** (101 MHz, Chloroform-*d*) δ 153.90, 153.50, 141.32, 140.23, 137.77, 135.83, 130.06, 128.59, 128.37, 128.35, 126.02, 108.98, 106.03, 67.19, 21.58, 19.93; **HRMS** (ESI): *m/z*: calculated for $C_{22}H_{22}NO_4$: $[M + H]^+$ 364.1549, found: 364.1553.

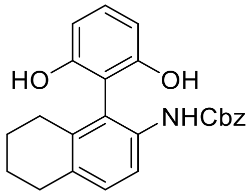

1q

1q: White solid ; **^{1}H NMR** (400 MHz, Chloroform-*d*) δ 7.88 (d, *J* = 8.4 Hz, 1H), 7.37 – 7.31 (m, 5H), 7.26 – 7.15 (m, 2H), 6.61 (d, *J* = 8.2 Hz, 2H), 6.45 (s, 1H), 5.10 (s, 2H), 4.95 (s, 2H), 2.80 (t, *J* = 6.2 Hz, 2H), 2.32 (t, *J* = 6.3 Hz, 2H), 1.86 – 1.59 (m, 4H). **^{13}C NMR** (101 MHz, Chloroform-*d*) δ 153.99, 153.66, 138.60, 135.85, 135.20, 134.48, 131.50, 130.71, 128.58, 128.33, 128.31, 109.17, 108.14, 67.13, 29.47, 27.02, 22.92, 22.63; **HRMS** (ESI): *m/z*: calculated for $C_{24}H_{24}NO_4$: $[M + H]^+$ 390.1705, found: 390.1708.

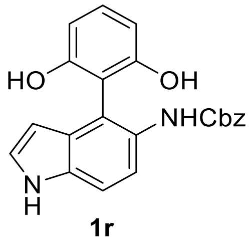

1r

1r: Colorless oil; **^{1}H NMR** (400 MHz, Methanol-d_4) δ 7.42 (s, 2H), 7.30 (q, *J* = 7.4, 6.6 Hz, 5H), 7.21 (d, *J* = 3.1 Hz, 1H), 7.12 (t, *J* = 8.1 Hz, 1H), 6.53 (d, *J* = 8.1 Hz, 2H), 6.11 (d, *J* = 3.2 Hz, 1H), 5.10 (s, 2H). **^{13}C NMR** (101 MHz, Methanol-d_4) δ 156.14, 155.40, 136.83, 134.20, 128.83, 128.17, 128.03, 127.63, 127.44, 127.18, 124.85, 118.81, 111.46, 110.50, 106.98, 101.62, 66.09; **HRMS** (ESI): *m/z*: calculated for $C_{22}H_{19}N_2O_4$: $[M + H]^+$ 375.1345, found: 375.1341.

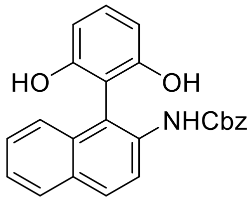

1s

1s: White solid; **^{1}H NMR** (400 MHz, Methanol-d_4) δ 8.10 (s, 1H), 7.90 – 7.84 (m, 2H), 7.43 – 7.36 (m, 2H), 7.36 – 7.30 (m, 6H), 7.22 (t, *J* = 8.2 Hz, 1H), 6.58 (d, *J* = 8.2 Hz, 2H), 5.14 (s, 2H). **^{13}C NMR** (101 MHz, Methanol-d_4) δ 156.19, 154.62, 136.52, 133.82, 132.91, 131.10, 129.77, 128.10, 127.82, 127.67, 127.50, 127.47, 125.62, 125.57, 124.22, 109.29, 106.88, 66.36; **HRMS** (ESI): *m/z*: calculated for $C_{24}H_{20}NO_4$: $[M + H]^+$ 386.1392, found: 386.1397.

Supplementary Note 3

General procedure for preparation of racemic compounds 3 and 4

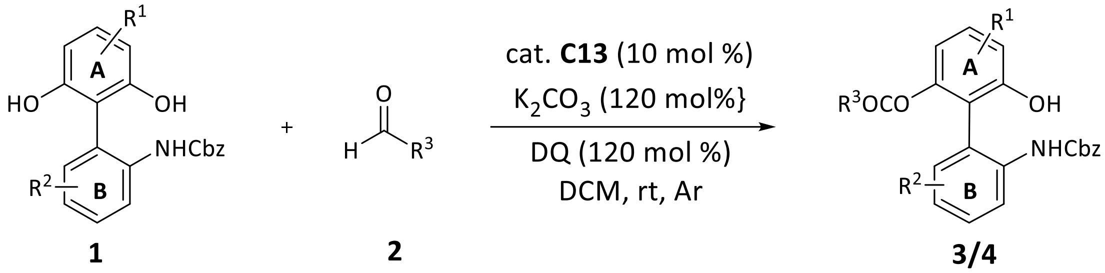

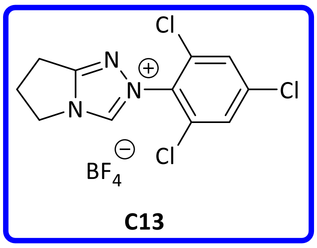

To a flame-dried Schlenk reaction tube equipped with a magnetic stir bar, was added the azolium precatalyst **C13** (3.8 mg, 0.01 mmol), K_2CO_3 (16.7 mg, 0.12 mmol), DQ (49.0 mg, 0.12 mmol) and **1** (0.10 mmol). The Schlenk tube was closed with a septum, evacuated and refilled with N_2. aldehyde **2** (13.0 mg, 0.15 mmol), and freshly distilled CH_2Cl_2 (1.0 mL) was added. The mixture was then stirred at room temperature and monitored by TLC until **1** was full consumed. The mixture was concentrated under reduced pressure and purified by via column chromatography on silica gel (hexanes/EtOAc = 5:1) to afford the racemic product **3/4**.

General procedure for preparation of compounds 3 and 4

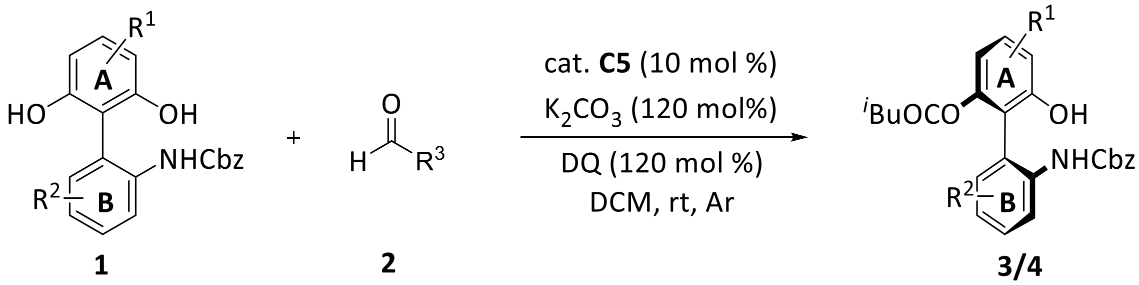

To a flame-dried Schlenk reaction tube equipped with a magnetic stir bar, was added the azolium precatalyst **C5** (5.5 mg, 0.01 mmol), K_2CO_3 (16.7 mg, 0.12 mmol), DQ

(49.0 mg, 0.12 mmol) and **1** (0.10 mmol). The Schlenk tube was closed with a septum, evacuated and refilled with N_2. aldehyde **2** (13.0 mg, 0.15 mmol), and freshly distilled CH_2Cl_2 (1.0 mL) was added. The mixture was then stirred at room temperature and monitored by TLC until **1** was full consumed. The mixture was concentrated under reduced pressure and purified by via column chromatography on silica gel (hexanes/EtOAc = 5:1) to afford the desired product **3/4**.

Characterization data of 3/4

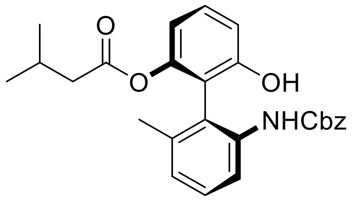

3a

3a: Colorless oil, 40 mg, 92%, 12 h; **^{1}H NMR** (400 MHz, Chloroform-*d*) δ 7.80 (d, *J* = 8.3 Hz, 1H), 7.43 – 7.31 (m, 7H), 7.0 (d, *J* = 8.2 Hz, 1H), 6.95 (dd, *J* = 8.3, 1.1 Hz, 1H), 6.75 (dd, *J* = 8.1, 1.1 Hz, 1H), 6.67 (s, 1H), 5.15 (s, 2H), 5.12 (s, 1H), 2.07 (dd, *J* = 7.1, 4.0 Hz, 2H), 2.05 (s, 3H), 1.88 – 1.75 (m, 1H), 0.71 (q, *J* = 3.6 Hz, 6H). **^{13}C NMR** (101 MHz, Chloroform-*d*) δ 171.89, 154.28, 154.19, 149.36, 139.27, 137.00, 136.21, 130.39, 129.80, 128.48, 128.11, 128.07, 126.36, 116.90, 114.57, 113.92, 66.91, 42.81, 25.50, 22.01, 21.98, 19.87; **HRMS** (ESI): *m/z*: calculated for $C_{26}H_{28}NO_5$: $[M + H]^+$ 434.1967, found: 434.1964; HPLC (Chiralpak AD-H, *i*-propanol/hexane = 10/90, flow rate 1.0 mL/min, λ = 254 nm): t_R (major) = 7.9 min, t_R (minor) = 6.8 min, *ee* = 99%; $[\alpha]^{25}_D$ = -135.7 (*c* = 1.0, $CHCl_3$).

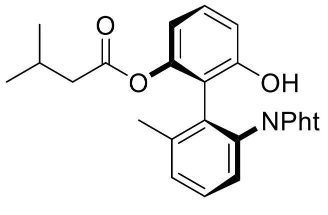

3ab

3ab: Colorless oil, 27 mg, 61%, 12 h; **^{1}H NMR** (400 MHz, Chloroform-*d*) δ 7.93 – 7.91 (m, 1H), 7.77 – 7.67 (m, 3H), 7.50 – 7.42 (m, 2H), 7.24 (dd, *J* = 7.9, 1.4 Hz, 1H),

7.19 (t, J = 8.2 Hz, 1H), 6.95 (dd, J = 8.3, 1.1 Hz, 1H), 6.55 (dd, J = 8.1, 1.1 Hz, 1H), 5.55 (s, 1H), 2.19 (s, 3H), 2.18 – 2.14 (m, 1H), 2.08 (dd, J = 15.1, 7.5 Hz, 1H), 1.92 – 1.82 (m, 1H), 0.85 (d, J = 6.7 Hz, 3H), 0.75 (d, J = 6.7 Hz, 3H). **^{13}C NMR** (101 MHz, Chloroform-*d*) δ 171.36, 167.61, 166.73, 154.72, 148.31, 134.29, 134.13, 131.98, 131.94, 131.36, 131.18, 130.62, 129.64, 129.44, 126.30, 123.70, 119.10, 114.97, 114.85, 42.33, 25.23, 22.17, 21.99, 19.57; **HRMS** (ESI): *m/z*: calculated for $C_{26}H_{24}NO_5$: [M + H]$^+$ 430.1654, found: 430.1657; HPLC (Chiralpak ID, *i*-propanol/hexane = 10/90, flow rate 1.0 mL/min, λ = 254 nm): t_R (major) = 27.1 min, t_R (minor) = 19.2 min, *ee* = 79%; $[\alpha]^{25}_D$ = - 122.0 (*c* = 1.0, $CHCl_3$).

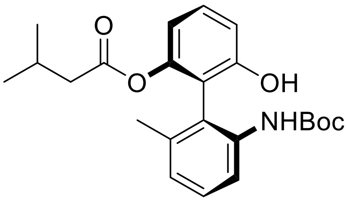

3ac

3ac: Colorless oil, 20 mg, 50%, 12 h; **^{1}H NMR** (400 MHz, Chloroform-*d*) δ 7.70 (d, J = 8.2 Hz, 1H), 7.36 (t, J = 8.2 Hz, 1H), 7.31 (t, J = 7.9 Hz, 1H), 7.08 (d, J = 7.5 Hz, 1H), 6.97 (dd, J = 8.3, 1.1 Hz, 1H), 6.75 (dd, J = 8.0, 1.1 Hz, 1H), 6.37 (s, 1H), 5.24 (s, 1H), 2.07 (dd, J = 7.1, 5.1 Hz, 2H), 2.04 (s, 3H), 1.85 – 1.78 (m, 1H), 1.45 (s, 9H), 0.71 (dd, J = 6.7, 5.2 Hz, 6H). **^{13}C NMR** (101 MHz, Chloroform-*d*) δ 171.85, 154.37, 154.05, 149.34, 139.18, 137.36, 130.17, 129.64, 126.24, 122.40, 121.01, 117.32, 114.45, 113.97, 80.51, 42.83, 28.25, 25.49, 22.03, 22.00, 19.87; **HRMS** (ESI): *m/z*: calculated for $C_{23}H_{30}NO_5$: [M + H]$^+$ 400.2046, found: 400.4025; HPLC (Chiralpak AD-H, *i*-propanol/hexane = 2.5/97.5, flow rate 1.0 mL/min, λ = 254 nm): t_R (major) = 11.1 min, t_R (minor) = 9.3 min, *ee* = 93%; $[\alpha]^{25}_D$ = - 142.0 (*c* = 1.0, $CHCl_3$).

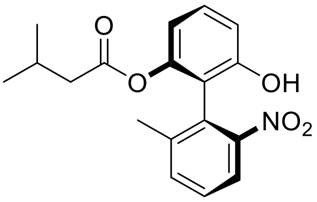

3ad

3ad: Colorless oil, 23.5 mg, 71%, 12 h; **^{1}H NMR** (400 MHz, Chloroform-*d*) δ 7.83

(dd, *J* = 8.2, 1.2 Hz, 1H), 7.56 (d, *J* = 7.6 Hz, 1H), 7.46 (t, *J* = 7.9 Hz, 1H), 7.32 (t, *J* = 8.2 Hz, 1H), 6.89 (dd, *J* = 8.2, 1.0 Hz, 1H), 6.77 (dd, *J* = 8.1, 1.0 Hz, 1H), 5.10 (s, 1H), 2.20 (s, 3H), 2.05 (dd, *J* = 7.1, 5.2 Hz, 2H), 1.85 – 1.78 (m, 1H), 0.72 (dd, *J* = 6.7, 2.3 Hz, 6H). **^{13}C NMR** (101 MHz, Chloroform-*d*) δ 170.86, 153.77, 150.87, 148.26, 141.64, 134.61, 129.92, 129.03, 126.05, 121.78, 117.31, 115.07, 113.52, 42.81, 25.37, 22.05, 21.97, 19.78; **HRMS** (ESI): *m/z*: calculated for $C_{18}H_{20}NO_5$: $[M + H]^+$ 330.1341, found: 330.1337; HPLC (Chiralpak ID, *i*-propanol/hexane = 10/90, flow rate 1.0 mL/min, λ = 254 nm): t_R (major) = 7.3 min, t_R (minor) = 6.7 min, *ee* = 50%; $[\alpha]^{25}_D$ = - 173.5 (*c* = 1.0, $CHCl_3$).

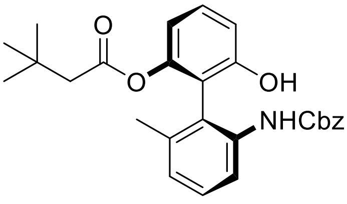

3b

3b: Colorless oil, 39 mg, 86%, 18 h; **^{1}H NMR** (400 MHz, Chloroform-*d*) δ 7.81 (d, *J* = 8.2 Hz, 1H), 7.38 – 7.32 (m, 7H), 7.09 (d, *J* = 7.6 Hz, 1H), 6.96 (dd, *J* = 8.3, 1.1 Hz, 1H), 6.74 (dd, *J* = 8.1, 1.1 Hz, 1H), 6.72 (s, 1H), 5.15 (s, 2H), 2.11 (d, *J* = 2.5 Hz, 1H), 2.04 (s, 3H), 0.80 (s, 9H). **^{13}C NMR** (101 MHz, Chloroform-*d*) δ 171.16, 154.23, 154.15, 149.46, 139.31, 137.13, 136.26, 130.39, 129.86, 128.46, 128.06, 128.00, 126.37, 121.83, 116.98, 114.69, 113.84, 66.84, 47.27, 30.53, 29.15, 19.88; **HRMS** (ESI): *m/z*: calculated for $C_{27}H_{30}NO_5$: $[M + H]^+$ 448.2124, found: 448.2127; HPLC (Chiralpak AD-H, *i*-propanol/hexane = 10/90, flow rate 1.0 mL/min, λ = 254 nm): t_R (major) = 7.0 min, t_R (minor) = 6.1 min, *ee* > 99%; $[\alpha]^{25}_D$ = - 62.6 (*c* = 1.0, $CHCl_3$).

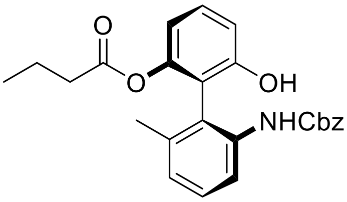

3c

3c: Colorless oil, 35 mg, 83%, 24 h; **^{1}H NMR** (400 MHz, Chloroform-*d*) δ 7.80 (d, *J* = 8.2 Hz, 1H), 7.40 – 7.30 (m, 7H), 7.10 (d, *J* = 7.6 Hz, 1H), 6.95 (dd, *J* = 8.3, 1.1 Hz,

1H), 6.76 (dd, J = 8.1, 1.1 Hz, 1H), 6.63 (s, 1H), 5.15 (d, J = 2.2 Hz, 2H), 5.12 (s, 1H), 2.17 – 2.12 (m, 2H), 2.05 (s, 3H), 1.49 – 1.21 (m, 2H), 0.69 (t, J = 7.4 Hz, 3H). **^{13}C NMR** (101 MHz, Chloroform-*d*) δ 172.41, 154.26, 154.17, 149.38, 139.26, 136.99, 136.21, 130.38, 129.78, 128.47, 128.10, 128.07, 126.30, 121.78, 120.24, 116.81, 114.59, 113.89, 66.91, 35.72, 19.83, 18.13, 13.26; **HRMS** (ESI): *m/z*: calculated for $C_{25}H_{26}NO_5$: $[M + H]^+$ 420.1811, found: 420.1815; HPLC (Chiralpak AD-H, *i*-propanol/hexane = 10/90, flow rate 1.0 mL/min, λ = 254 nm): t_R (major) = 8.9 min, t_R (minor) = 7.5 min, *ee* = 86%; $[\alpha]^{25}_D$ = -55.6 (*c* = 1.0, $CHCl_3$).

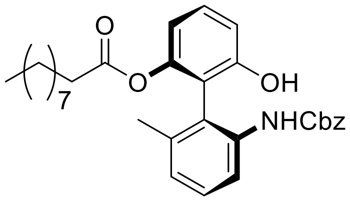

3d

3d: Colorless oil, 45 mg, 90%, 18 h; **^{1}H NMR** (400 MHz, Chloroform-*d*) δ 7.80 (d, J = 8.3 Hz, 1H), 7.40 – 7.30 (m, 7H), 7.10 (d, J = 7.6 Hz, 1H), 6.95 (dd, J = 8.3, 1.1 Hz, 1H), 6.75 (dd, J = 8.0, 1.1 Hz, 1H), 6.64 (s, 1H), 5.15 (d, J = 2.3 Hz, 3H), 2.18 – 2.14 (m, 2H), 2.05 (s, 3H), 1.42 – 1.10 (m, 12H), 1.06 – 1.00 (m, 2H), 0.92 (t, J = 7.0 Hz, 3H). **^{13}C NMR** (101 MHz, Chloroform-*d*) δ 172.63, 154.26, 154.16, 149.38, 139.28, 137.03, 136.22, 130.38, 129.81, 128.46, 128.09, 128.06, 126.31, 121.79, 120.33, 116.83, 114.58, 113.89, 66.90, 33.94, 31.88, 29.26, 29.14, 28.72, 24.65, 22.69, 19.84, 14.13; **HRMS** (ESI): *m/z*: calculated for $C_{31}H_{38}NO_5$: $[M + H]^+$ 504.2750, found: 504.2754; HPLC (Chiralpak AD-H, *i*-propanol/hexane = 10/90, flow rate 1.0 mL/min, λ = 254 nm): t_R (major) = 6.4 min, t_R (minor) = 5.4 min, *ee* = 89%; $[\alpha]^{25}_D$ = -48.2 (*c* = 1.0, $CHCl_3$).

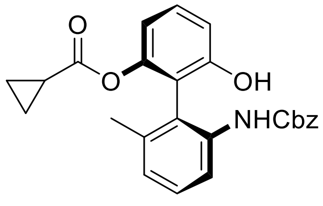

3e

3e: Colorless oil, 35 mg, 84%, 12 h; **^{1}H NMR** (400 MHz, Chloroform-*d*) δ 7.80 (d, J = 8.2 Hz, 1H), 7.41 – 7.32 (m, 7H), 7.12 (d, J = 7.6 Hz, 1H), 6.94 (dd, J = 8.3, 1.1 Hz,

1H), 6.78 (dd, *J* = 8.1, 1.1 Hz, 1H), 6.60 (s, 1H), 5.15 (d, *J* = 2.3 Hz, 2H), 5.01 (s, 1H), 2.05 (s, 3H), 1.64 – 1.37 (m, 1H), 0.79 – 0.44 (m, 4H). **^{13}C NMR** (101 MHz, Chloroform-*d*) δ 173.53, 154.27, 154.12, 149.33, 139.24, 136.99, 136.23, 130.33, 129.77, 128.09, 128.06, 126.31, 121.81, 120.26, 116.79, 114.61, 113.82, 66.87, 19.84, 12.59, 8.78, 8.45; **HRMS** (ESI): *m/z*: calculated for $C_{25}H_{24}NO_5$: $[M + H]^+$ 418.1654, found: 418.1658; HPLC (Chiralpak AD-H, *i*-propanol/hexane = 10/90, flow rate 1.0 mL/min, λ = 254 nm): t_R (major) = 11.6 min, t_R (minor) = 9.2 min, *ee* = 90%; $[\alpha]^{25}_D$ = -62.4 (*c* = 1.0, $CHCl_3$).

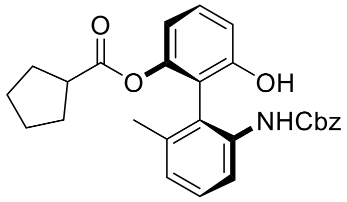

3f

3f: Colorless oil, 41 mg, 91%, 12 h; **^{1}H NMR** (400 MHz, Chloroform-*d*) δ 7.78 (d, *J* = 8.3 Hz, 1H), 7.37 – 7.33 (m, 7H), 7.10 (d, *J* = 7.6 Hz, 1H), 6.94 (dd, *J* = 8.3, 1.0 Hz, 1H), 6.76 (dd, *J* = 8.1, 1.0 Hz, 1H), 6.66 (s, 1H), 5.32 (s, 1H), 5.15 (s, 2H), 2.86 – 2.62 (m, 1H), 2.05 (s, 3H), 1.67 – 1.57 (m, 2H), 1.48 -1.42 (m, 5H), 1.24 – 1.18 (m, 1H). **^{13}C NMR** (101 MHz, Chloroform-*d*) δ 175.68, 154.36, 154.22, 149.43, 139.39, 137.03, 136.22, 130.35, 129.74, 128.48, 128.10, 128.05, 126.29, 122.05, 120.30, 116.82, 114.55, 113.89, 66.90, 43.33, 29.79, 29.26, 25.61, 25.58, 19.88.; **HRMS** (ESI): *m/z*: calculated for $C_{27}H_{28}NO_5$: $[M + H]^+$ 446.1967, found: 446.1970; HPLC (Chiralpak AD-H, *i*-propanol/hexane = 10/90, flow rate 1.0 mL/min, λ = 254 nm): t_R (major) = 8.9 min, t_R (minor) = 7.6 min, *ee* = 95%; $[\alpha]^{25}_D$ = - 44.1 (*c* = 1.0, $CHCl_3$).

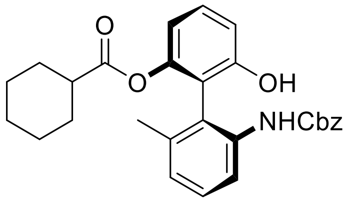

3g

3g: Colorless oil, 38 mg, 82%, 12 h; **^{1}H NMR** (400 MHz, Chloroform-*d*) δ 7.79 (d, *J* = 8.2 Hz, 1H), 7.43 – 7.31 (m, 7H), 7.10 (d, *J* = 7.6 Hz, 1H), 6.95 (dd, *J* = 8.3, 1.1 Hz,

1H), 6.74 (dd, *J* = 8.0, 1.1 Hz, 1H), 6.65 (s, 1H), 5.15 (s, 2H), 5.01 (s, 1H), 2.24 – 2.19 (m, 1H), 2.05 (s, 3H), 1.63 – 1.46 (m, 6H), 1.16 – 0.99 (m, 4H). **^{13}C NMR** (101 MHz, Chloroform-*d*) δ 174.92, 154.29, 154.15, 149.41, 139.35, 137.07, 136.25, 130.34, 129.76, 128.47, 128.08, 128.04, 126.26, 121.83, 120.27, 116.83, 114.61, 113.84, 66.87, 42.74, 28.38, 28.20, 25.49, 25.14, 24.99, 19.89; **HRMS** (ESI): *m/z*: calculated for $C_{28}H_{30}NO_5$: $[M + H]^+$ 460.2124, found: 460.2121; HPLC (Chiralpak AD-H, *i*-propanol/hexane = 10/90, flow rate 1.0 mL/min, λ = 254 nm): t_R (major) = 8.5 min, t_R (minor) = 7.0 min, *ee* = 98%; $[\alpha]^{25}_D$ = - 26.3 (*c* = 1.0, $CHCl_3$).

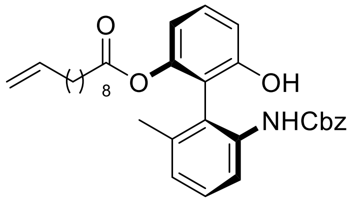

3h

3h: Colorless oil, 42 mg, 80%, 18 h; **^{1}H NMR** (400 MHz, Chloroform-*d*) δ 7.79 (d, *J* = 8.2 Hz, 1H), 7.37 – 7.32 (m, 7H), 7.10 (d, *J* = 7.5 Hz, 1H), 6.94 (dd, *J* = 8.3, 1.0 Hz, 1H), 6.75 (dd, *J* = 8.0, 1.0 Hz, 1H), 6.65 (s, 1H), 5.90 – 5.80 (m, 1H), 5.24 (s, 1H), 5.15 (s, 2H), 5.03 (dd, *J* = 17.2, 1.9 Hz, 1H), 4.99 – 4.96 (m, 1H), 2.18 – 2,14 (m, 2H), 2.10 – 2.06 (m, 2H), 2.05 (s, 3H), 1.43 – 1.36 (m, 2H), 1.30 – 1.13 (m, 8H), 1.07 – 1.01 (m, 2H). **^{13}C NMR** (101 MHz, Chloroform-*d*) δ 172.61, 154.31, 154.26, 149.39, 139.27, 139.15, 136.99, 136.23, 130.34, 129.70, 128.46, 128.09, 128.04, 126.28, 122.03, 120.31, 116.84, 114.52, 114.20, 113.94, 66.91, 33.92, 33.80, 29.13, 29.07, 29.05, 28.91, 28.69, 24.63, 19.84; **HRMS** (ESI): *m/z*: calculated for $C_{32}H_{38}NO_5$: $[M + H]^+$ 516.2750, found: 516.2754; HPLC (Chiralpak AD-H, *i*-propanol/hexane = 10/90, flow rate 1.0 mL/min, λ = 254 nm): t_R (major) = 7.1 min, t_R (minor) = 5.9 min, *ee* = 83%; $[\alpha]^{25}_D$ = - 81.7 (*c* = 1.0, $CHCl_3$).

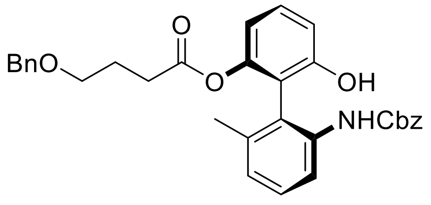

3i

3i: Colorless oil, 47 mg, 89%, 18 h; **^{1}H NMR** (400 MHz, Chloroform-*d*) δ 7.78 (d, *J* = 8.2 Hz, 1H), 7.38 – 7.34 (m, 7H), 7.33 – 7.30 (m, 4H), 7.09 (d, *J* = 7.6 Hz, 1H), 6.95 (dd, *J* = 8.3, 1.1 Hz, 1H), 6.73 (dd, *J* = 8.0, 1.0 Hz, 1H), 6.63 (s, 1H), 5.15 (d, *J* = 1.7 Hz, 2H), 5.12 (s, 1H), 4.41 (s, 2H), 3.26 – 3.22 (m, 2H), 2.30 (q, *J* = 7.2 Hz, 2H), 2.04 (s, 3H), 1.65 – 1.59 (m, 3H). **^{13}C NMR** (101 MHz, Chloroform-*d*) δ 172.27, 154.28, 154.19, 149.33, 139.30, 138.31, 137.02, 136.20, 130.39, 129.79, 128.47, 128.37, 128.11, 128.07, 127.59, 126.32, 121.91, 120.28, 116.77, 114.57, 113.97, 72.81, 68.65, 66.92, 30.67, 24.81, 19.84; **HRMS** (ESI): *m/z*: calculated for $C_{32}H_{32}NO_6$: $[M + H]^+$ 526.2230, found: 526.2232; HPLC (Chiralpak AD-H, *i*-propanol/hexane = 10/90, flow rate 1.0 mL/min, λ = 254 nm): t_R (major) = 24.0 min, t_R (minor) = 15.6 min, *ee* = 80%; $[\alpha]^{25}_D$ = - 23.8 (*c* = 1.0, $CHCl_3$).

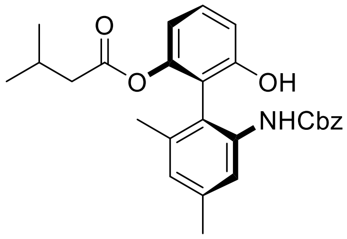

4a

4a: Colorless oil, 40 mg, 89%, 18 h; **^{1}H NMR** (400 MHz, Chloroform-*d*) δ 7.64 (s, 1H), 7.37 – 7.32 (m, 6H), 6.94 (dd, *J* = 8.4, 1.0 Hz, 1H), 6.93 (s, 1H), 6.73 (dd, *J* = 8.1, 1.0 Hz, 1H), 6.63 (s, 1H), 5.15 (s, 3H), 2.36 (s, 3H), 2.08 (dd, *J* = 7.1, 3.5 Hz, 2H), 2.01 (s, 3H), 1.88 – 1.77 (m, 1H), 0.71 (q, *J* = 8.0, 6H). **^{13}C NMR** (101 MHz, Chloroform-*d*) δ 171.87, 154.36, 154.28, 139.90, 138.91, 136.77, 136.28, 130.26, 128.47, 128.08, 128.04, 127.34, 120.86, 118.69, 116.94, 114.48, 113.75, 66.85, 42.84, 25.54, 21.97, 21.94, 21.41, 19.75; **HRMS** (ESI): *m/z*: calculated for $C_{27}H_{30}NO_5$: $[M + H]^+$ 448.2124, found: 448.2127; HPLC (Chiralpak AD-H, *i*-propanol/hexane = 5/95, flow rate 1.0 mL/min, λ = 254 nm): t_R (major) = 11.4 min, t_R (minor) = 11.0 min, *ee* > 99%; $[\alpha]^{25}_D$ = - 66.3 (*c* = 1.0, $CHCl_3$).

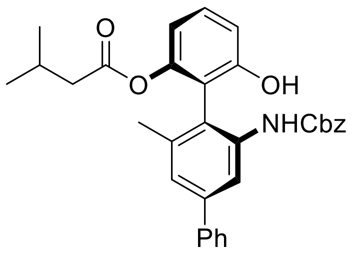

4b

4b: Colorless oil, 45 mg, 89%, 18 h; **^{1}H NMR** (400 MHz, Chloroform-*d*) δ 8.07 (s, 1H), 7.63 (d, *J* = 7.6 Hz, 2H), 7.47 (t, *J* = 7.4 Hz, 2H), 7.41 – 7.32 (m, 8H), 6.98 (dd, *J* = 8.3, 1.1 Hz, 1H), 6.77 (dd, *J* = 8.1, 1.0 Hz, 1H), 6.74 (s, 1H), 5.25 (s, 1H), 5.24 – 5.11 (m, 2H), 2.11 (s, 3H), 2.10 (d, *J* = 2.0 Hz, 2H), 1.85 – 1.77 (m, 1H), 0.68 (q, *J* = 4.0 Hz, 6H). **^{13}C NMR** (101 MHz, Chloroform-*d*) δ 171.91, 154.29, 149.48, 142.77, 140.40, 139.60, 137.38, 136.17, 130.49, 128.77, 128.52, 128.16, 128.09, 127.69, 127.21, 125.06, 120.69, 119.02, 116.71, 114.61, 113.95, 66.99, 42.90, 25.59, 21.96, 21.94, 20.08; **HRMS** (ESI): *m/z*: calculated for $C_{32}H_{32}NO_5$: $[M + H]^+$ 510.2280, found: 510.2283; HPLC (Chiralpak AD-H, *i*-propanol/hexane = 10/90, flow rate 1.0 mL/min, λ = 254 nm): t_R (major) = 20.5 min, t_R (minor) = 18.5 min, *ee* > 99%; $[\alpha]^{25}_D$ = - 73.1 (*c* = 1.0, $CHCl_3$).

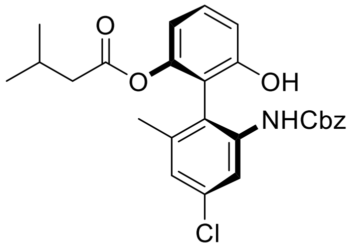

4c

4c: Colorless oil, 43 mg, 92%, 24 h; **^{1}H NMR** (400 MHz, Chloroform-*d*) δ 7.78 (s, 1H), 7.39 – 7.35 (m, 6H), 7.12 (s, 1H), 6.92 (dd, *J* = 8.3, 1.1 Hz, 1H), 6.77 (dd, *J* = 8.1, 1.0 Hz, 1H), 6.71 (s, 1H), 5.15 (s, 2H), 5.13 (s, 1H), 2.38 (s, 3H), 2.14 (dd, *J* = 7.1, 1.4 Hz, 2H), 1.92 – 1.85 (m, 1H), 0.75 (q, *J* = 4.2 Hz, 6H). **^{13}C NMR** (101 MHz, Chloroform-*d*) δ 171.89, 154.32, 153.96, 149.76, 141.30, 138.26, 136.03, 130.89, 128.50, 128.19, 128.11, 125.94, 121.23, 118.44, 115.40, 114.59, 113.92, 67.09, 42.87, 25.55, 22.01, 21.96, 21.37; **HRMS** (ESI): *m/z*: calculated for $C_{26}H_{27}ClNO_5$: $[M + H]^+$ 468.1578, found: 468.1581; HPLC (Chiralpak AD-H, *i*-propanol/hexane = 2/98, flow

rate 1.0 mL/min, λ = 254 nm): t_R (major) = 30.0 min, t_R (minor) = 27.3 min, *ee* = 98%; $[\alpha]^{25}_D$ = - 161.0 (*c* = 1.0, $CHCl_3$).

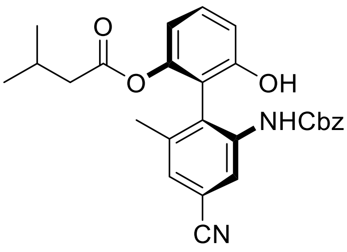

4d

4d: Colorless oil, 39 mg, 85%, 36 h; **^{1}H NMR** (400 MHz, Chloroform-*d*) δ 8.20 (s, 1H), 7.39 – 7.33 (m, 6H), 7.31 (d, *J* = 0.9 Hz, 1H), 6.89 (dd, *J* = 8.4, 1.0 Hz, 1H), 6.76 (dd, *J* = 8.2, 1.0 Hz, 1H), 6.73 (s, 1H), 5.76 (s, 1H), 5.15 (s, 2H), 2.10 – 2.08 (m, 2H), 2.07 (s, 3H), 1.87 – 1.76 (m, 1H), 0.72 (t, *J* = 6.3 Hz, 6H). **^{13}C NMR** (101 MHz, Chloroform-*d*) δ 171.75, 153.94, 153.89, 149.16, 140.75, 137.87, 135.72, 131.15, 128.60, 128.56, 128.36, 128.21, 127.52, 122.35, 118.38, 115.33, 114.95, 114.31, 112.89, 67.40, 42.75, 25.53, 21.91, 19.87; **HRMS** (ESI): *m/z*: calculated for $C_{27}H_{27}N_2O_5$: $[M + H]^+$ 459.1920, found: 459.1924; HPLC (Chiralpak AD-H, *i*-propanol/hexane = 10/90, flow rate 1.0 mL/min, λ = 254 nm): t_R (major) = 7.8 min, t_R (minor) = 8.9 min, *ee* = 98%; $[\alpha]^{25}_D$ = - 71.7 (*c* = 1.0, $CHCl_3$).

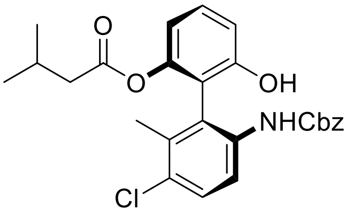

4e

4e: Colorless oil, 43 mg, 93%, 36 h; **^{1}H NMR** (400 MHz, Chloroform-*d*) δ 7.76 (d, *J* = 8.9 Hz, 1H), 7.43 (d, *J* = 8.8 Hz, 1H), 7.38 – 7.33 (m, 6H), 6.93 (dd, *J* = 8.3, 1.0 Hz, 1H), 6.75 (dd, *J* = 8.2, 1.0 Hz, 1H), 6.62 (s, 1H), 5.26 (s, 1H), 5.14 (s, 2H), 2.10 (d, *J* = 1.4 Hz, 2H), 2.08 (s, 3H), 1.85 – 1.78 (m, 1H), 0.72 (dd, *J* = 6.7, 1.9 Hz, 6H). **^{13}C NMR** (101 MHz, Chloroform-*d*) δ 171.81, 154.18, 154.12, 149.33, 136.95, 136.03, 135.71, 130.74, 130.29, 128.50, 128.19, 128.11, 123.99, 121.31, 116.69, 114.74, 114.16, 67.08, 25.54, 21.96, 21.90, 17.44; **HRMS** (ESI): *m/z*: calculated for $C_{26}H_{27}ClNO_5$: $[M + H]^+$ 468.1578, found: 468.1575; HPLC (Chiralpak AD-H,

i-propanol/hexane = 10/90, flow rate 1.0 mL/min, λ = 254 nm): t_R (major) = 10.2 min, t_R (minor) = 7.9 min, *ee* = 98%; $[\alpha]^{25}_D$ = - 19.1 (*c* = 1.0, $CHCl_3$).

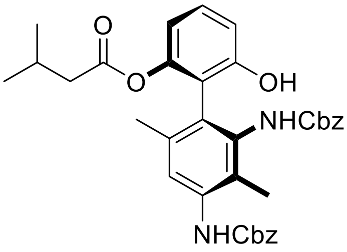

4f

4f: Colorless oil, 52 mg, 87%, 18 h; **^{1}H NMR** (400 MHz, Chloroform-*d*) δ 7.86 (s, 1H), 7.47 – 7.40 (m, 4H), 7.39 – 7.33 (m, 3H), 7.31 – 7.27 (m, 4H), 6.88 (dd, *J* = 8.3, 1.1 Hz, 1H), 6.71 (s, 1H), 6.68 (dd, *J* = 8.1, 1.1 Hz, 1H), 6.62 (s, 1H), 5.25 (s, 2H), 5.19 (d, *J* = 12.5 Hz, 1H), 5.07 (d, *J* = 12.4 Hz, 1H), 2.13 – 2.07 (m, 4H), 2.04 – 1.99 (m, 4H), 1.83 – 1.74 (m, 1H), 0.69 (q, *J* = 8.4, 6H). **^{13}C NMR** (101 MHz, Chloroform-*d*) δ 172.54, 155.43, 154.56, 153.50, 149.21, 137.30, 137.24, 136.43, 135.94, 135.25, 129.74, 128.68, 128.48, 128.42, 128.01, 127.92, 124.08, 121.57, 118.77, 114.31, 113.92, 67.28, 66.97, 42.74, 25.46, 21.87, 21.85, 19.83, 12.52; **HRMS** (ESI): *m/z*: calculated for $C_{35}H_{37}N_2O_7$: $[M + H]^+$ 597.2601, found: 597.2605; HPLC (Chiralpak AD-H, *i*-propanol/hexane = 20/80, flow rate 1.0 mL/min, λ = 254 nm): t_R (major) = 18.5 min, t_R (minor) = 9.4 min, *ee* > 99%; $[\alpha]^{25}_D$ = - 33.8 (*c* = 1.0, $CHCl_3$).

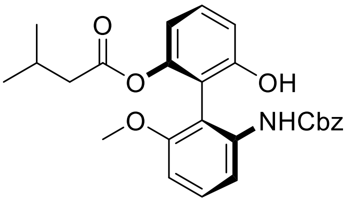

4g

4g: Colorless oil, 40 mg, 90%, 15 h; **^{1}H NMR** (400 MHz, Chloroform-*d*) δ 7.68 (d, *J* = 8.3 Hz, 1H), 7.42 – 7.32 (m, 7H), 6.95 (dd, *J* = 8.3, 1.1 Hz, 1H), 6.78 – 6.75 (m, 2H), 6.65 (s, 1H), 5.16 (s, 2H), 5.13 (s, 1H), 3.75 (s, 3H), 2.10 – 2.08 (m, 2H), 1.87 – 1.80 (m, 1H), 0.74 (q, *J* = 6.2, 6H). **^{13}C NMR** (101 MHz, Chloroform-*d*) δ 171.71, 157.93, 154.63, 153.94, 149.88, 138.12, 136.16, 130.82, 130.33, 128.48, 128.13, 128.10, 114.51, 114.01, 113.81, 110.43, 106.86, 66.93, 56.11, 42.85, 25.50, 22.06;

HRMS (ESI): *m/z*: calculated for $C_{26}H_{28}NO_6$: $[M + H]^+$ 450.1917, found: 450.1919; HPLC (Chiralpak AD-H, *i*-propanol/hexane = 20/80, flow rate 1.0 mL/min, λ = 254 nm): t_R (major) = 9.4 min, t_R (minor) = 6.0 min, *ee* = 98%; $[\alpha]^{25}_D$ = - 31.5 (*c* = 1.0, $CHCl_3$).

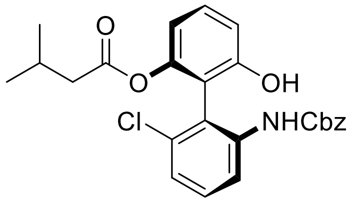

4h

4h: Colorless oil, 41 mg, 90%, 24 h; **^{1}H NMR** (400 MHz, Chloroform-*d*) δ 7.94 (d, *J* = 8.2 Hz, 1H), 7.40 – 7.34 (m, 7H), 7.28 (dd, *J* = 8.0, 1.3 Hz, 2H), 6.93 (dd, *J* = 8.3, 1.0 Hz, 1H), 6.78 (dd, *J* = 8.2, 1.0 Hz, 1H), 6.76 (s, 1H), 5.15 (s, 3H), 2.13 (dd, *J* = 7.1, 1.7 Hz, 2H), 1.89 – 1.83 (m, 1H), 0.73 (q, *J* = 4.0 Hz, 6H). **^{13}C NMR** (101 MHz, Chloroform-*d*) δ 171.95, 154.16, 153.96, 149.61, 138.66, 135.94, 135.49, 131.04, 130.60, 128.52, 128.24, 128.18, 125.22, 121.69, 120.55, 115.41, 114.66, 114.08, 67.17, 42.85, 25.53, 22.05, 22.01; **HRMS** (ESI): *m/z*: calculated for $C_{25}H_{25}ClNO_5$: $[M + H]^+$ 454.1421, found: 454.1418; HPLC (Chiralpak AD-H, *i*-propanol/hexane = 10/90, flow rate 1.0 mL/min, λ = 254 nm): t_R (major) = 11.0 min, t_R (minor) = 8.9 min, *ee* = 98%; $[\alpha]^{25}_D$ = - 15.3 (*c* = 1.0, $CHCl_3$).

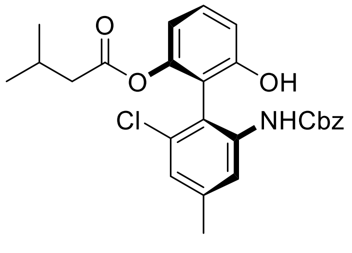

4i

4i: Colorless oil, 44 mg, 94%, 24 h; **^{1}H NMR** (400 MHz, Chloroform-*d*) δ 7.94 (s, 1H), 7.39 – 7.29 (m, 6H), 7.09 (s, 1H), 6.93 (d, *J* = 8.3 Hz, 1H), 6.75 (d, *J* = 8.1 Hz, 1H), 6.66 (s, 1H), 5.15 (s, 2H), 5.08 (s, 1H), 2.10 (dd, *J* = 7.3, 3.3 Hz, 2H), 2.02 (s, 3H), 1.92 – 1.85 (m, 1H), 0.75 (t, *J* = 5.8 Hz, 6H). **^{13}C NMR** (101 MHz, Chloroform-*d*) δ 171.78, 154.12, 153.81, 149.44, 140.78, 138.11, 135.97, 135.48, 130.77, 128.52, 128.23, 128.13, 125.96, 119.61, 115.80, 114.84, 114.01, 67.14, 42.81,

25.58, 21.94, 21.92, 19.78; **HRMS** (ESI): *m/z*: calculated for $C_{26}H_{27}ClNO_5$: $[M + H]^+$ 468.1578, found: 468.1574; HPLC (Chiralpak AD-H, *i*-propanol/hexane = 10/90, flow rate 1.0 mL/min, λ = 254 nm): t_R (major) = 8.3 min, t_R (minor) = 7.3 min, *ee* = 98%; $[\alpha]^{25}_D$ = - 17.8 (*c* = 1.0, $CHCl_3$).

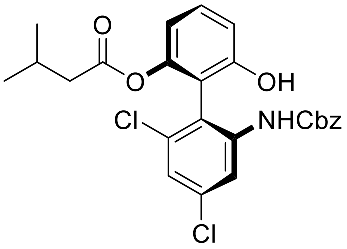

4j

4j: Colorless oil, 45 mg, 93%, 24 h; **^{1}H NMR** (400 MHz, Chloroform-*d*) δ 8.09 (s, 1H), 7.41 – 7.34 (m, 6H), 7.28 (s, 1H), 6.91 (dd, *J* = 8.3, 1.0 Hz, 1H), 6.79 (dd, *J* = 8.1, 1.0 Hz, 1H), 6.74 (s, 1H), 5.36 – 4.95 (m, 3H), 2.16 (dd, *J* = 7.2, 1.4 Hz, 2H), 1.93 – 1.86 (m, 1H), 0.78 (dd, *J* = 6.7, 2.9 Hz, 6H). **^{13}C NMR** (101 MHz, Chloroform-*d*) δ 171.86, 154.09, 153.49, 149.71, 139.34, 136.05, 135.70, 131.38, 128.56, 128.37, 128.26, 124.69, 119.92, 119.64, 114.93, 114.39, 114.11, 67.40, 42.86, 22.01, 21.96; **HRMS** (ESI): *m/z*: calculated for $C_{25}H_{24}Cl_2NO_5$: $[M + H]^+$ 488.1032, found: 488.1035; HPLC (Chiralpak AD-H, *i*-propanol/hexane = 4/96, flow rate 1.0 mL/min, λ = 254 nm): t_R (major) = 15.9 min, t_R (minor) = 13.8 min, *ee* = 98%; $[\alpha]^{25}_D$ = - 9.6 (*c* = 1.0, $CHCl_3$).

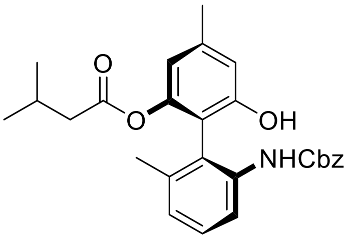

4k

4k: Colorless oil, 42 mg, 93%, 24 h; **^{1}H NMR** (400 MHz, Chloroform-*d*) δ 7.82 (d, *J* = 8.2 Hz, 1H), 7.38 – 7.30 (m, 6H), 7.07 (dd, *J* = 7.6, 1.0 Hz, 1H), 6.77 (dd, *J* = 1.5, 0.8 Hz, 1H), 6.69 (s, 1H), 6.57 (dd, *J* = 1.5, 0.7 Hz, 1H), 5.16 (s, 2H), 4.99 (s, 1H), 2.38 (s, 3H), 2.07 – 2.05 (m, 5H), 1.84 – 1.77 (m, 1H), 0.70 (dd, *J* = 6.7, 5.2 Hz, 6H). **^{13}C NMR** (101 MHz, Chloroform-*d*) δ 171.93, 154.19, 153.78, 149.05, 141.04, 139.43, 137.17, 136.26, 129.68, 128.48, 128.11, 126.15, 121.59, 119.85, 115.41,

114.52, 113.71, 66.88, 42.83, 25.53, 22.02, 22.00, 21.47, 19.92; **HRMS** (ESI): *m/z*: calculated for $C_{27}H_{30}NO_5$: [M + H]$^+$ 448.2124, found: 448.2126; HPLC (Chiralpak AD-H, *i*-propanol/hexane = 10/90, flow rate 1.0 mL/min, λ = 254 nm): t_R (major) = 5.1 min, t_R (minor) = 4.3 min, *ee* = 96%; $[\alpha]^{25}_D$ = - 129.6 (*c* = 1.0, $CHCl_3$).

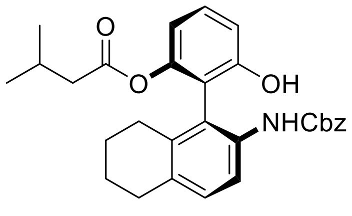

4l

4l: Colorless oil, 43 mg, 91%, 18 h; **^{1}H NMR** (400 MHz, Chloroform-*d*) δ 7.64 (d, *J* = 7.9 Hz, 1H), 7.36 – 7.32 (m, 6H), 7.16 (d, *J* = 8.4 Hz, 1H), 6.95 (dd, *J* = 8.3, 1.1 Hz, 1H), 6.73 (dd, *J* = 8.1, 1.1 Hz, 1H), 6.61 (s, 1H), 5.14 (d, *J* = 2.5 Hz, 2H), 5.03 (s, 1H), 2.79 – 2.77 (m, 2H), 2.45 – 2.38 (m, 1H), 2.25 – 2.17 (m, 1H), 2.09 (d, *J* = 7.1 Hz, 2H), 1.84 – 1.77 (m, 2H), 1.75 – 1.67 (m, 3H), 0.69 (q, *J* = 2.7 Hz, 6H). **^{13}C NMR** (101 MHz, Chloroform-*d*) δ 171.87, 154.52, 154.12, 149.30, 137.66, 136.33, 134.63, 134.44, 130.90, 130.18, 128.44, 128.03, 122.07, 120.91, 117.19, 114.52, 113.87, 66.81, 42.95, 29.54, 27.01, 25.60, 22.97, 22.75, 22.04, 21.96; **HRMS** (ESI): *m/z*: calculated for $C_{29}H_{32}NO_5$: [M + H]$^+$ 474.2280, found: 474.2277; HPLC (Chiralpak AD-H, *i*-propanol/hexane = 10/90, flow rate 1.0 mL/min, λ = 254 nm): t_R (major) = 12.5 min, t_R (minor) = 7.7 min, *ee* = 96%; $[\alpha]^{25}_D$ = - 46.2 (*c* = 1.0, $CHCl_3$).

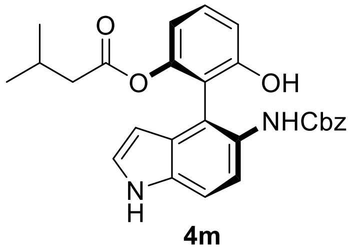

4m

4m: Colorless oil, 41 mg, 90%, 24 h; **^{1}H NMR** (400 MHz, Chloroform-*d*) δ 8.35 (s, 1H), 7.64 (s, 1H), 7.44 (d, *J* = 8.7 Hz, 1H), 7.40 – 7.32 (m, 6H), 7.19 (t, *J* = 2.8 Hz, 1H), 6.99 (dd, *J* = 8.3, 1.1 Hz, 1H), 6.78 (dd, *J* = 8.0, 1.0 Hz, 1H), 6.71 (s, 1H), 6.16 (s, 1H), 5.19 (s, 3H), 1,97 – 1.85 (m, 2H), 1.66 – 1.59 (m, 1H), 0.54 – 0.51 (m, 6H). **^{13}C NMR** (101 MHz, Chloroform-*d*) δ 171.72, 155.30, 154.71, 149.64, 136.42,

133.52, 130.04, 129.14, 128.47, 128.04, 127.96, 125.65, 119.56, 116.91, 114.19, 113.80, 112.43, 102.22, 66.87, 42.73, 25.25, 21.84, 21.81; **HRMS** (ESI): *m/z*: calculated for $C_{27}H_{27}N_2O_5$: $[M + H]^+$ 459.1920, found: 459.1918; HPLC (Chiralpak AD-H, *i*-propanol/hexane = 20/80, flow rate 1.0 mL/min, λ = 254 nm): t_R (major) = 14.2 min, t_R (minor) = 6.3 min, *ee* = 98%; $[\alpha]^{25}_D$ = - 130.5 (*c* = 1.0, $CHCl_3$).

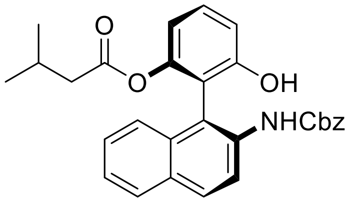

4n

4n: Colorless oil, 39 mg, 84%, 24 h; **^{1}H NMR** (400 MHz, Chloroform-*d*) δ 8.17 (d, *J* = 9.0 Hz, 1H), 7.95 (d, *J* = 9.0 Hz, 1H), 7.86 (d, *J* = 7.1 Hz, 1H), 7.48 – 7.33 (m, 9H), 7.03 (d, *J* = 8.2 Hz, 1H), 6.93 (s, 1H), 6.84 (d, *J* = 8.1 Hz, 1H), 5.20 (s, 2H), 4.99 (s, 1H), 1.94 – 1.81 (m, 2H), 1.57 – 1.50 (m, 1H), 0.47 (dd, *J* = 6.7, 4.9 Hz, 6H). **^{13}C NMR** (101 MHz, Chloroform-*d*) δ 171.81, 154.79, 154.24, 150.10, 136.10, 135.44, 132.36, 131.01, 130.79, 130.37, 128.52, 128.19, 128.15, 128.04, 127.24, 125.47, 124.88, 121.64, 116.94, 115.55, 114.75, 114.03, 67.10, 42.66, 25.28, 21.75; **HRMS** (ESI): *m/z*: calculated for $C_{29}H_{28}NO_5$: $[M + H]^+$ 470.1967, found: 470.1970; HPLC (Chiralpak AD-H, *i*-propanol/hexane = 20/80, flow rate 1.0 mL/min, λ = 254 nm): t_R (major) = 8.9 min, t_R (minor) = 6.5 min, *ee* > 99%; $[\alpha]^{25}_D$ = - 62.3 (*c* = 1.0, $CHCl_3$).

General procedure for preparation of compounds 4o and characterization data

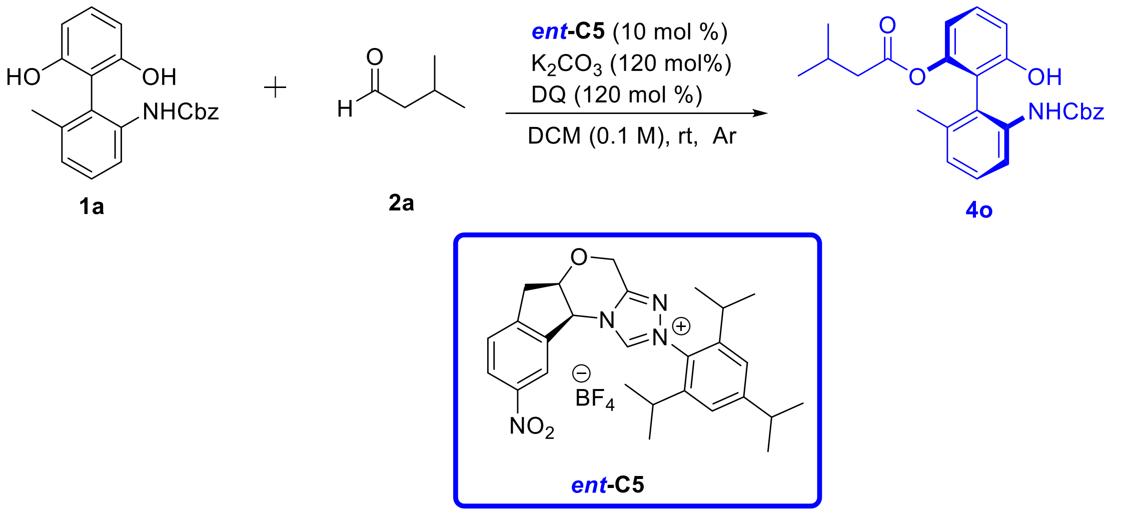

To a flame-dried Schlenk reaction tube equipped with a magnetic stir bar, was added the azolium precatalyst ***ent*-C5** (5.5 mg, 0.01 mmol), K_2CO_3 (16.7 mg, 0.12 mmol), DQ (49.0 mg, 0.12 mmol) and **1a** (35.0 mg, 0.10 mmol). The Schlenk tube was closed with a septum, evacuated and refilled with N_2. Aldehyde **2a** (13.0 mg, 0.15 mmol), and freshly distilled CH_2Cl_2 (1.0 mL) was added. The mixture was then stirred at room temperature and monitored by TLC until **1a** was full consumed. The mixture was concentrated under reduced pressure and purified by *via* column chromatography on silica gel (hexanes/EtOAc = 5:1) to afford the desired product **4o** in 90% yield with > 99% ee.

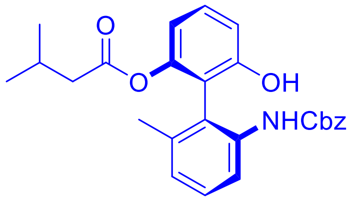

4o

4o: Colorless oil, 39 mg, 90%, 12 h; **^{1}H NMR** (400 MHz, Chloroform-*d*) δ 7.80 (d, *J* = 8.3 Hz, 1H), 7.43 – 7.31 (m, 7H), 7.0 (d, *J* = 8.2 Hz, 1H), 6.95 (dd, *J* = 8.3, 1.1 Hz, 1H), 6.75 (dd, *J* = 8.1, 1.1 Hz, 1H), 6.67 (s, 1H), 5.15 (s, 2H), 5.12 (s, 1H), 2.07 (dd, *J* = 7.1, 4.0 Hz, 2H), 2.05 (s, 3H), 1.88-1.75 (m, 1H), 0.71 (q, *J* = 3.6 Hz, 6H). **^{13}C NMR** (101 MHz, Chloroform-*d*) δ 171.89, 154.28, 154.19, 149.36, 139.27, 137.00, 136.21, 130.39, 129.80, 128.48, 128.11, 128.07, 126.36, 116.90, 114.57, 113.92, 66.91, 42.81, 25.50, 22.01, 21.98, 19.87; **HRMS** (ESI): *m/z*: calculated for $C_{26}H_{28}NO_5$: [M +

H]$^+$ 434.1967, found: 434.1964; HPLC (Chiralpak AD-H, *i*-propanol/hexane = 10/90, flow rate 1.0 mL/min, λ = 254 nm): t_R (major) = 6.6 min, t_R (minor) = 7.7 min, *ee* > 99%; $[\alpha]^{25}_D$ = +130.8 (*c* = 1.0, $CHCl_3$).

Gram scale reaction

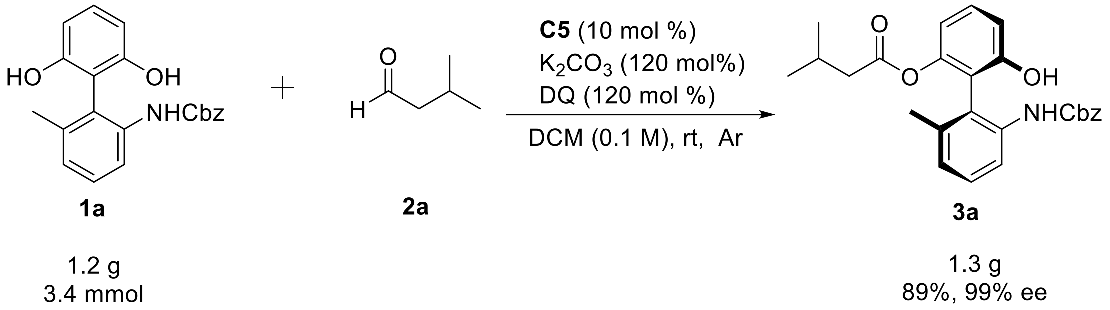

To a flame-dried 100 mL Schlenk reaction tube equipped with a magnetic stir bar, was added the azolium precatalyst **C5** (187 mg, 0.34 mmol), K_2CO_3 (567.8 mg, 4.08 mmol), DQ (1.66 g, 4.08 mmol) and **1a** (1.2 g, 3.4 mmol). The Schlenk tube was closed with a septum, evacuated and refilled with N_2. Aldehyde **2a** (442.0 mg, 5.1 mmol), and freshly distilled CH_2Cl_2 (34.0 mL) was added. The mixture was then stirred at room temperature and monitored by TLC until **1a** was full consumed. The mixture was concentrated under reduced pressure and purified by *via* column chromatography on silica gel (hexanes/EtOAc = 5:1) to afford 1.3 g product **3a** in 89% yield with 99% ee.

Supplementary Note 4

General procedure for for NHC-catalyzed kinetic resolution (KR) of *rac*-3a with 2a and characterization data

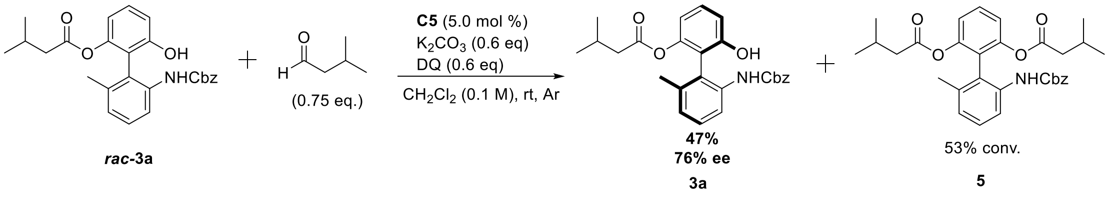

To a flame-dried Schlenk reaction tube equipped with a magnetic stir bar, was added the azolium precatalyst **C5** (2.7 mg, 0.05 mmol), K_2CO_3 (8.3 mg, 0.06 mmol), DQ (24.5 mg, 0.06 mmol) and **1a** (43.3 mg, 0.10 mmol). The Schlenk tube was closed with a septum, evacuated and refilled with N_2. Aldehyde **2a** (6.5 mg, 0.075 mmol), and freshly distilled CH_2Cl_2 (1.0 mL) was added. The mixture was then stirred at room temperature and monitored by TLC until DQ was full consumed. The mixture was concentrated under reduced pressure and purified by *via* column chromatography on silica gel (hexanes/EtOAc = 10:1 to 5:1) to recovered **3a** in 47% yield with 76% ee and **4aa** with 53% conversion.

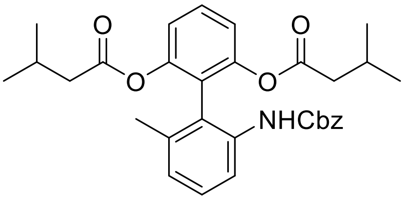

5

5: Colorless oil, 27 mg, 53%, 12 h; **^{1}H NMR** (400 MHz, Chloroform-*d*) δ 7.66 (d, J = 8.2 Hz, 1H), 7.49 (t, J = 8.1 Hz, 1H), 7.40 – 7.31 (m, 5H), 7.24 (t, J = 7.9 Hz, 1H), 7.09 (d, J = 8.2 Hz, 2H), 6.99 (d, J = 7.5 Hz, 1H), 6.52 (s, 1H), 5.17 (s, 2H), 2.05 (s, 5H), 2.03 (s, 2H), 1.83 – 1.76 (m, 2H), 0.69 (dd, J = 6.7, 1.4 Hz, 12H). **^{13}C NMR** (101 MHz, Chloroform-*d*) δ 171.46, 154.00, 149.79, 138.08, 136.71, 136.44, 129.88, 128.83, 128.36, 127.92, 127.90, 125.66, 124.96, 120.58, 66.54, 42.58, 25.32, 22.01, 21.97, 19.65; **HRMS** (ESI): *m/z*: calculated for $C_{31}H_{36}NO_6$: $[M + H]^+$ 518.2543, found: 518.2547.

Procedure for NHC-catalyzed desymmetrization of bisphenol 1a with cat. C5 without subsequent kinetic resolution (KR) reaction

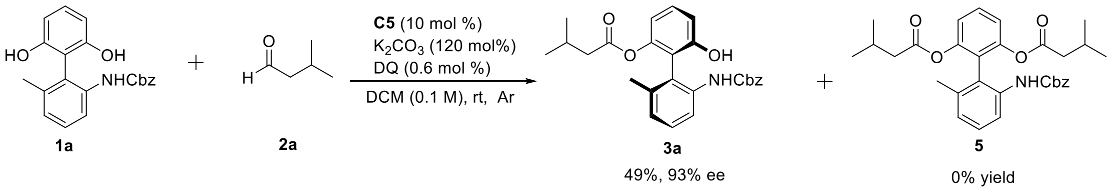

To a flame-dried Schlenk reaction tube equipped with a magnetic stir bar, was added the azolium precatalyst **C5** (5.5 mg, 0.01 mmol), K_2CO_3 (16.7 mg, 0.12 mmol), DQ (24.5.0 mg, 0.06 mmol) and **1a** (35.0 mg, 0.10 mmol). The Schlenk tube was closed with a septum, evacuated and refilled with N_2. Aldehyde **2a** (13.0 mg, 0.15 mmol), and freshly distilled CH_2Cl_2 (1.0 mL) was added. The mixture was then stirred at room temperature and monitored by TLC. The mixture was concentrated under reduced pressure and purified by *via* column chromatography on silica gel (hexanes/EtOAc = 5:1) to afford the desired product **3a** in 49% yield with 93% ee.

NHC-catalyzed tandem desymmetrization-kinetic resolution

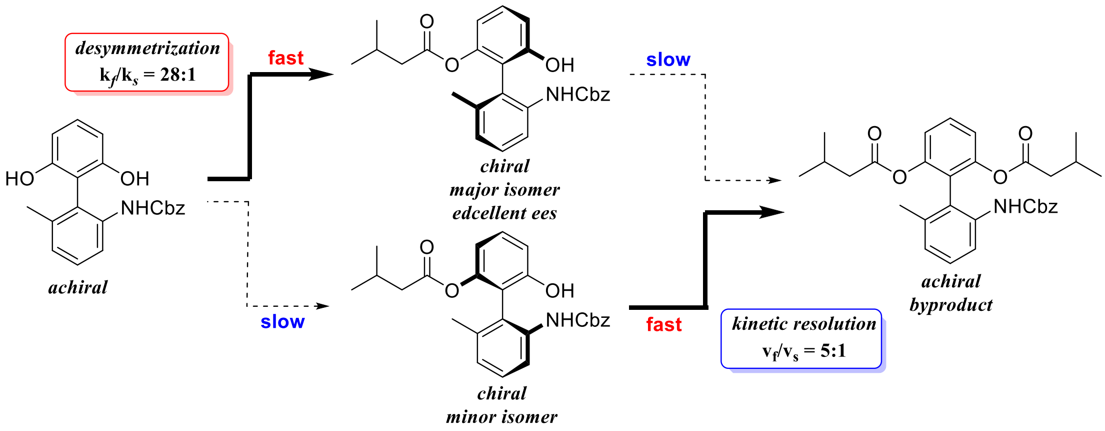

Supplementary Note 5

General Procedure for synthesis of biaryl amino-alcohol 7 and characterization data

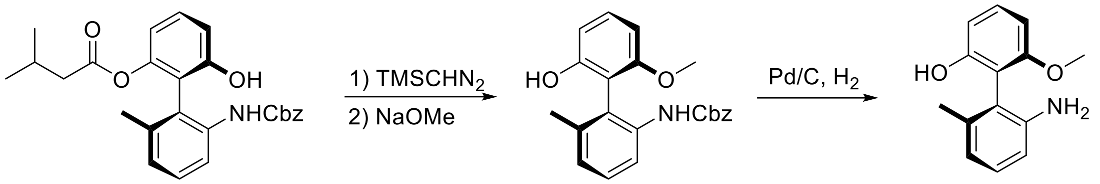

3a > 99% ee **6a** **7a** **> 99% ee** 87% yield in total

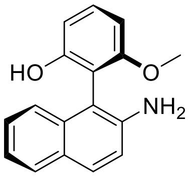

7

To a 50 mL RBF equipped with a magnetic stir bar, was added **3a** (1.0 mmol) and TMSCHN$_2$ (0.6 mL, 2 M in hexane, 1.2 equiv.) in CHCl$_3$ (10.0 mL) and MeOH (2.0 mL). The reaction was stirred for 24 hours in room temperature. After the reaction completed, the mixture was concentrated to give the crude product. The crude material was used for the next reaction without further purification.

To a 50 mL RBF equipped with a magnetic stir bar, was added crude material (1.0 mmol)**,** MeONa (1.5 mmol), MeOH (10.0 mL). The reaction was stirred 1 hour in room temperature. Quenched the reaction with 20 mL sat. NH$_4$Cl, and extracted with EA (30mL*3). Combined the organic layer and dried with MgSO$_4$. The organic solvent was concentrated under vacuum to give crude product **6a**. The crude material was used for the next reaction without further purification.

To a stirred solution of crude **6a** in methanol (10.0 mL) was added 5% Pd/C (50.0 mg). The reaction mixture was thoroughly purged with nitrogen and then stirred under 1 atm of hydrogen at room temperature for 18 h. The mixture was filtered through a pad of Celite and the filtrate was concentrated under reduced pressure. The crude residue was purified *via* column chromatography on silica gel to afford the desired product **7a** (198.5 mg, 87% yield in total).

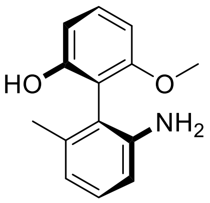

7a

7a: White solid, 42 mg, 87% yield in total; **^{1}H NMR** (400 MHz, Chloroform-*d*) δ 7.29 (t, *J* = 8.2 Hz, 2H), 7.15 (t, *J* = 7.8 Hz, 1H), 6.78 (d, *J* = 7.5 Hz, 1H), 6.71 (dd, *J* = 11.4, 8.1 Hz, 2H), 6.61 (d, *J* = 8.3 Hz, 1H), 3.94 (s, 3H), 3.76 (s, 3H), 2.02 (s, 3H). **^{13}C NMR** (101 MHz, Chloroform-*d*) δ 157.86, 154.12, 145.01, 139.67, 129.81, 129.40, 120.63, 116.64, 113.25, 111.91, 108.69, 103.25, 55.86, 19.90; **HRMS** (ESI): *m/z*: calculated for $C_{14}H_{16}NO_2$: $[M + H]^+$ 230.1181, found: 230.1183; HPLC (Chiralpak OJ-H, *i*-propanol/hexane = 20/80, flow rate 1.0 mL/min, λ = 254 nm): t_R (major) = 13.4 min, t_R (minor) = 29.1 min, *ee* > 99%; $[\alpha]^{25}_D$ = - 4.8 (*c* = 1.0, MeOH).

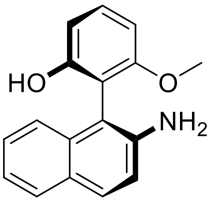

7

7: White solid, 49 mg, 89% yield in total; **^{1}H NMR** (400 MHz, Methanol-d_4) δ 7.70 (t, *J* = 7.9 Hz, 2H), 7.31 (t, *J* = 8.3 Hz, 1H), 7.21 – 7.14 (m, 4H), 6.69 (t, *J* = 8.3 Hz, 2H), 3.64 (s, 3H). **^{13}C NMR** (101 MHz, Methanol-d_4) δ 158.99, 156.01, 142.60, 133.98, 129.29, 128.42, 128.15, 125.34, 123.75, 121.42, 118.28, 112.39, 111.44, 108.50, 102.59; **HRMS** (ESI): *m/z*: calculated for $C_{17}H_{16}NO_2$: $[M + H]^+$ 66.1181, found: 266.1184; $[\alpha]^{25}_D$ = - 14.7 (*c* = 1.0, MeOH).

Application of 7 as catalyst and characterization data

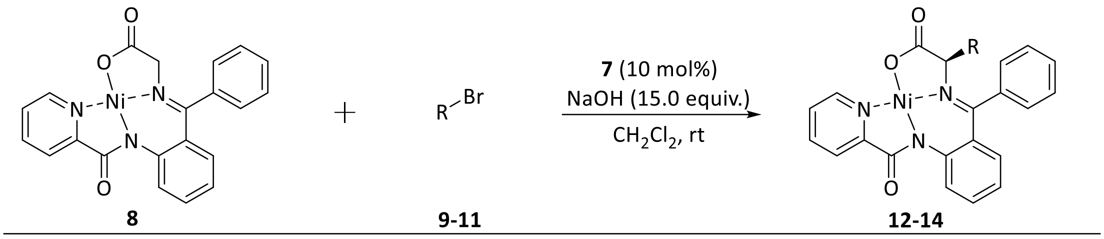

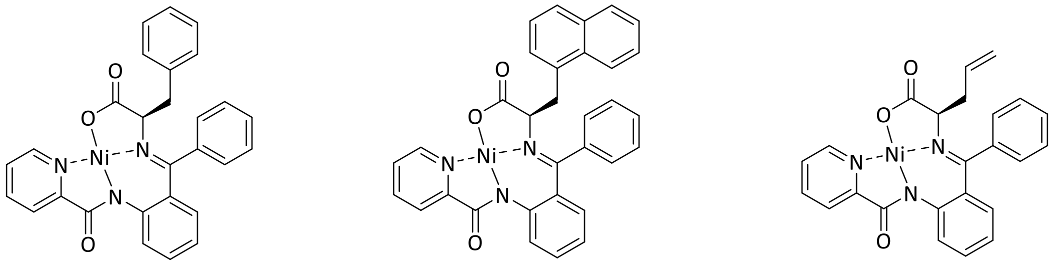

12	13	14
t = 3 min	t = 3 min	t = 5 min
95%, 94:6 er	94%, 86:14 er	89%, 90:10 er

A mixture of finely ground NaOH (120.0 mg, 3.0 mmol), complex (83.0 mg, 0.2 mmol), and **7** (5.3 mg, 0.02 mmol) in anhydrous CH_2Cl_2 (1.5 mL) was stirred under Ar for 3 minutes at ambient temperature. Bromine compound (0.4 mmol) was then added under Ar and the mixture was stirred for an additional 3-5 min. The reaction was quenched by the addition of 10% aqueous AcOH (5 mL), and the mixture was diluted with CH_2Cl_2 (15 mL). The organic layer was separated and the mixture was concentrated under vacuum to give crude product**.** The crude residue was purified *via* column chromatography on silica gel to afford the desired product **12** (95 mg, 93% yield). The er of the product was determined by the corresponding derivative.

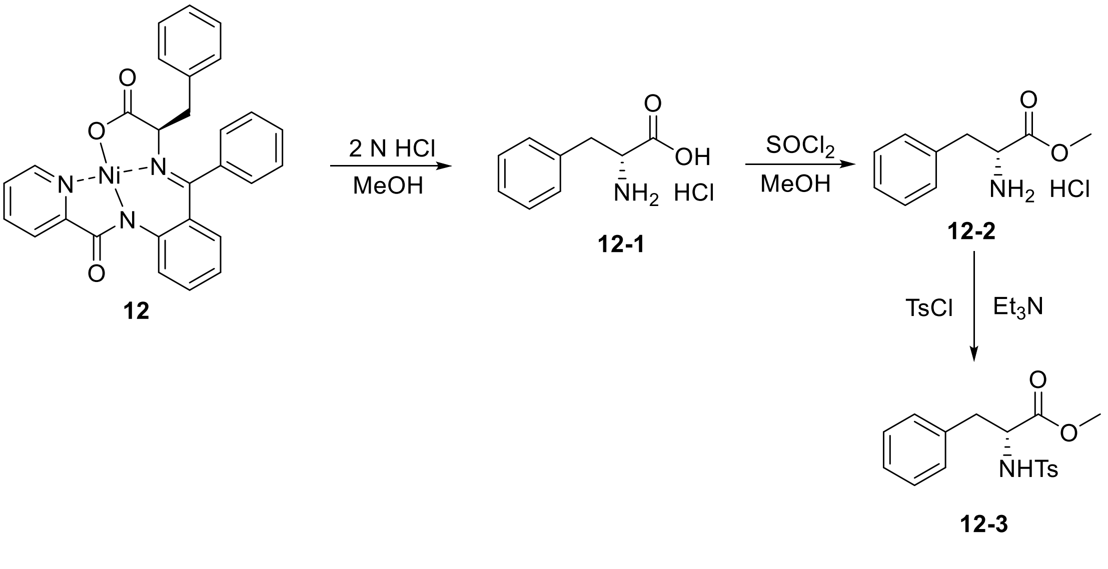

To a solution of **12** in 2N HCl in MeOH (2.0 mL), and the reaction mixture was

heat to 50 ^oC and stirred at this temperature for 5 min. The solvent was concentrated under vacuum to give the crude product **12-1**. The crude material was used for the next reaction without further purification.

To a solution of the crude material **12-1** in MeOH (4.0 mL), the reaction mixture was cooled to 0 ^oC, then the $SOCl_2$ (1.5 equiv.) was added and the reaction mixture was then warm to room temperature and heated to reflux for 12 h. The solvent was concentrated under vacuum to give the crude product **12-2**. The crude material was used for the next reaction without further purification.

To a solution of crude **12-2**, and Et_3N (3.0 equiv.) in dry CH_2Cl_2 (1.0 mL), then added TsCl (1.2 equiv.) to the reaction mixture and stirred at room temperature for 12 h. The reaction was quenched by the addition of sat. NH_4Cl aqueous (5 mL), and the mixture was diluted with CH_2Cl_2 (15 mL*3). The organic layer was separated and the mixture was concentrated under vacuum to give crude product**.** The crude residue was purified *via* column chromatography on silica gel to afford the desired product **12-3** (28 mg, 86% yield).

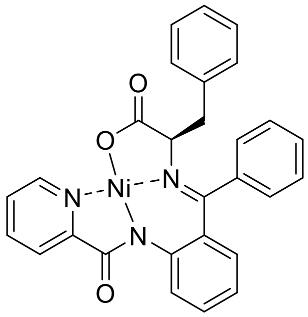

12

12: 48 mg, 95%, 3 min, red solid; **^{1}H NMR** (400 MHz, Chloroform-*d*) δ 8.68 (d, *J* = 8.6 Hz, 1H), 7.91 – 7.87 (m, 1H), 7.74 (dd, *J* = 7.8, 1.3 Hz, 1H), 7.65 (dd, *J* = 5.5, 1.4 Hz, 1H), 7.58 – 7.53 (m, 3H), 7.39 (d, *J* = 7.1 Hz, 2H), 7.34 – 7.26 (m, 3H), 7.16 – 7.12 (m, 3H), 6.84 (t, *J* = 7.5 Hz, 1H), 6.80 – 6.75 (m, 2H), 4.35 (dd, *J* = 5.7, 3.1 Hz, 1H), 3.11 (dd, *J* = 13.5, 3.1 Hz, 1H), 2.85 (dd, *J* = 13.5, 5.7 Hz, 1H). **^{13}C NMR** (101 MHz, Chloroform-*d*) δ 178.06, 171.22, 169.20, 153.12, 146.64, 143.17, 139.90, 135.78, 134.38, 133.64, 133.27, 131.08, 130.08, 129.30, 129.08, 128.15, 127.67, 127.51, 127.28, 127.17, 126.39, 123.64, 123.53, 121.37, 72.98, 40.00; er = 94:6

(detected by **12-3**).

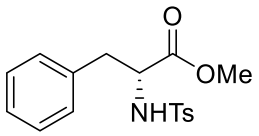

12-3

12-3: White solid, 28 mg, 86% yield in total; **^{1}H NMR** (400 MHz, Chloroform-*d*) δ 7.65 (d, *J* = 8.3 Hz, 2H), 7.27 – 7.24 (m, 5H), 7.09 (dd, *J* = 7.2, 2.4 Hz, 2H), 5.15 (d, *J* = 9.1 Hz, 1H), 4.25 – 4.20 (m, 1H), 3.51 (s, 3H), 3.05 (dd, *J* = 6.0, 1.6 Hz, 2H), 2.42 (s, 3H). **^{13}C NMR** (101 MHz, Chloroform-*d*) δ 171.25, 143.59, 136.65, 134.98, 129.60, 129.40, 128.57, 127.24, 127.18, 56.66, 52.36, 39.37, 21.53; **HRMS** (ESI): *m/z*: calculated for $C_{17}H_{20}NO_4S$: $[M + H]^+$ 334.1113, found: 334.1117; HPLC (Chiralpak IC, *i*-propanol/hexane = 20/80, flow rate 1.0 mL/min, λ = 254 nm): t_R (major) = 49.2 min, t_R (minor) = 33.5 min, er = 94:6; $[\alpha]^{25}_D$ = - 6.7 (*c* = 1.0, $CHCl_3$).

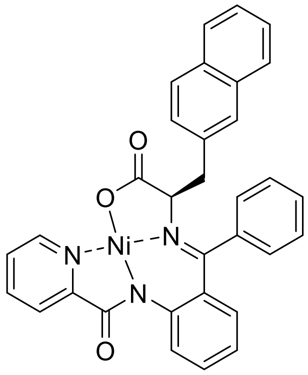

13

13: 53 mg, 92%, 3 min, red solid; **^{1}H NMR** (400 MHz, Chloroform-*d*) δ 8.66 (d, *J* = 8.6 Hz, 1H), 8.01 (d, *J* = 1.6 Hz, 1H), 7.65 (dd, *J* = 8.2, 6.4 Hz, 2H), 7.61 – 7.51 (m, 3H), 7.53 – 7.49 (m, 2H), 7.40 (d, *J* = 8.0 Hz, 1H), 7.35 – 7.20 (m, 7H), 6.77 – 6.86 (m, 3H), 4.43 (dd, *J* = 5.3, 3.0 Hz, 1H), 3.27 (dd, *J* = 13.5, 2.9 Hz, 1H), 3.02 (dd, *J* = 13.4, 5.2 Hz, 1H). **^{13}C NMR** (101 MHz, Chloroform-*d*) δ 171.22, 169.11, 152.10, 145.73, 143.24, 139.31, 134.36, 133.60, 133.33, 133.30, 132.44, 130.18, 130.15, 129.50, 129.37, 129.19, 127.93, 127.72, 127.43, 126.01, 125.67, 125.40, 123.71, 123.08, 121.40, 40.13; er = 82:18 (detected by **13-3**).

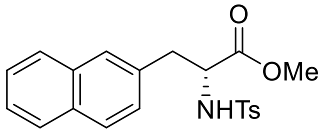

13-3

13-3: White solid, 33 mg, 87% yield in total; **^{1}H NMR** (400 MHz, Chloroform-*d*) δ 7.81 (t, *J* = 4.1 Hz, 1H), 7.72 (t, *J* = 6.5 Hz, 2H), 7.56 (d, *J* = 7.9 Hz, 2H), 7.52 – 7.47 (m, 3H), 7.19 (d, *J* = 8.4 Hz, 1H), 7.09 (d, *J* = 8.0 Hz, 2H), 5.20 (d, *J* = 9.1 Hz, 1H), 4.33 – 4.27 (m, 1H), 3.55 (d, *J* = 1.1 Hz, 3H), 3.24 (dd, *J* = 13.8, 5.6 Hz, 1H), 3.15 (dd, *J* = 13.8, 6.8 Hz, 1H), 2.34 (s, 3H). **^{13}C NMR** (101 MHz, Chloroform-*d*) δ 171.42, 143.50, 136.47, 133.34, 132.59, 132.52, 129.45, 128.29, 128.25, 127.65, 127.61, 127.25, 127.07, 126.17, 125.86, 56.82, 52.51, 39.51, 21.50; **HRMS** (ESI): *m/z*: calculated for $C_{21}H_{22}NO_4S$: $[M + H]^+$ 384.1270, found: 384.1268; HPLC (Chiralpak IC, *i*-propanol/hexane = 20/80, flow rate 1.0 mL/min, λ = 254 nm): t_R (major) = 58.4 min, t_R (minor) = 39.8 min, er = 82:18; $[\alpha]^{25}_D$ = - 3.6 (*c* = 1.0, $CHCl_3$).

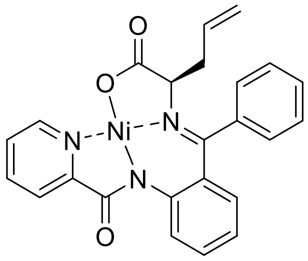

14

14: 41 mg, 89%, 5 min, red solid; **^{1}H NMR** (400 MHz, Chloroform-*d*) δ 8.90 (d, *J* = 8.6 Hz, 1H), 8.19 (d, *J* = 5.4 Hz, 1H), 7.99 (t, *J* = 7.5 Hz, 1H), 7.88 (d, *J* = 7.6 Hz, 1H), 7.54 (q, *J* = 6.8, 5.7 Hz, 3H), 7.42 (t, *J* = 6.1 Hz, 1H), 7.35 – 7.31 (m, 2H), 7.09 – 7.07 (m, 1H), 6.81 – 6.75 (m, 2H), 6.48 – 6.38 (m, 1H), 5.31 (dd, *J* = 11.2, 2.3 Hz, 1H), 5.15 (d, *J* = 16.0 Hz, 1H), 4.11 (dd, *J* = 6.5, 4.2 Hz, 1H), 2.50 (t, *J* = 7.0 Hz, 2H). **^{13}C NMR** (101 MHz, Chloroform-*d*) δ 178.88, 172.02, 169.97, 153.26, 146.98, 143.20, 140.56, 134.59, 133.92, 133.43, 132.04, 129.95, 129.29, 128.83, 127.92, 127.18, 127.03, 126.80, 124.07, 123.46, 121.41, 119.75, 38.73; e.r. = 90:10 (detected by **14-3**).

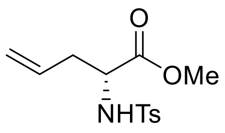

14-3

14-3: Colorless oil, 23 mg, 81% yield in total; **^{1}H NMR** (400 MHz, Chloroform-*d*) δ 7.74 (d, *J* = 8.4 Hz, 2H), 7.31 (d, *J* = 8.4 Hz, 2H), 5.69 – 5.59 (m, 1H), 5.20 – 5.08 (m, 3H), 4.08 – 4.03 (m, 1H), 3.54 (s, 3H), 2.48 (t, *J* = 6.6 Hz, 2H), 2.44 (s, 3H). **^{13}C NMR** (101 MHz, Chloroform-*d*) δ 171.35, 143.72, 136.76, 131.26, 129.65, 127.26, 119.84, 55.21, 52.46, 37.59, 21.57; **HRMS** (ESI): *m/z*: calculated for $C_{13}H_{18}NO_4S$: $[M + H]^+$ 284.0957, found: 284.0956; HPLC (Chiralpak IC, *i*-propanol/hexane = 20/80, flow rate 1.0 mL/min, λ = 254 nm): t_R (major) = 28.3 min, t_R (minor) = 20.3 min, er = 90:10; $[\alpha]^{25}_D$ = - 6.7 (*c* = 1.0, $CHCl_3$).

General Procedure for synthesis of biaryl amino-alcohol derivatives 15 - 17 and characterization data

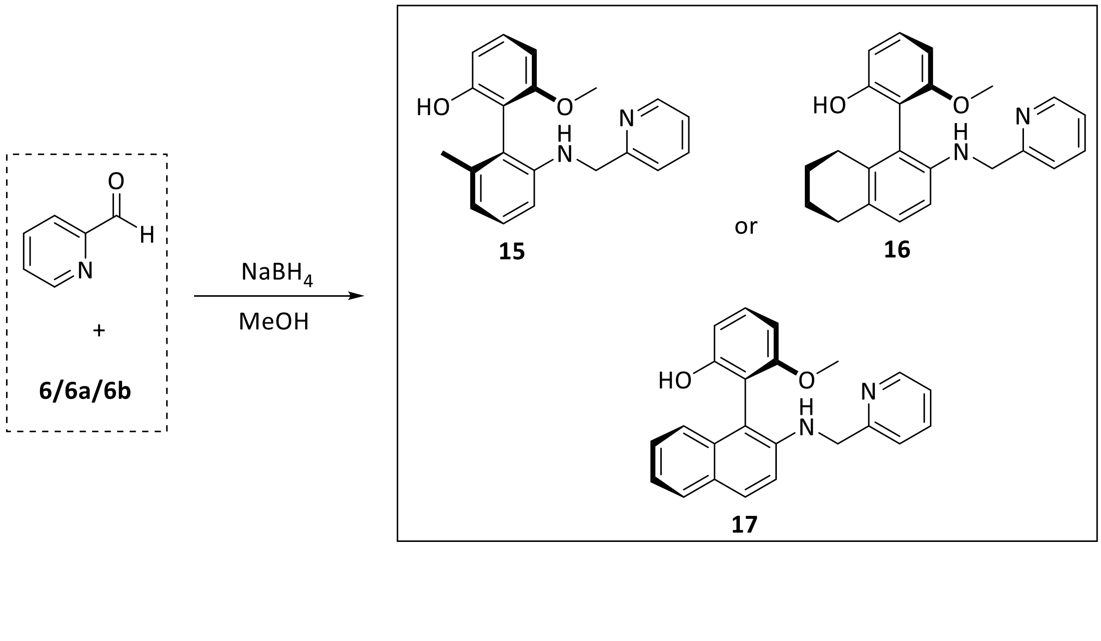

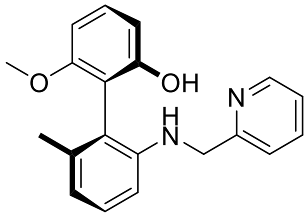

15

15: Colorless oil, 28 mg, 86%, 24 h; **^{1}H NMR** (400 MHz, Chloroform-*d*) δ 8.49 (d, *J* = 4.6 Hz, 1H), 7.85 – 7.60 (m, 1H), 7.34 – 7.28 (m, 2H), 7.16 – 7.13 (m, 1H), 7.09 (t, *J* = 7.8 Hz, 1H), 6.78 (d, *J* = 8.2 Hz, 1H), 6.70 (d, *J* = 7.5 Hz, 1H), 6.65 (d, *J* = 8.3 Hz, 1H), 6.45 (d, *J* = 8.2 Hz, 1H), 4.59 – 4.45 (m, 2H), 3.78 (s, 3H), 2.01 (s, 3H). **^{13}C NMR** (101 MHz, Chloroform-*d*) δ 159.13, 158.28, 154.68, 148.80, 145.77, 139.17, 136.94, 129.84, 129.19, 121.94, 121.31, 119.44, 117.21, 112.28, 109.60, 108.77, 103.38, 55.82, 48.59, 19.97; **HRMS** (ESI): *m/z*: calculated for $C_{20}H_{21}N_2O_2$: $[M + H]^+$ 321.1603, found: 321.1606; $[\alpha]^{25}_D$ = + 46.6 (*c* = 1.0, $CHCl_3$).

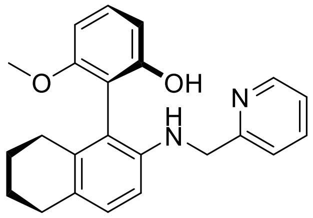

16

16: Colorless oil, 30 mg, 84%, 24 h; **^{1}H NMR** (400 MHz, Chloroform-*d*) δ 8.50 (d, *J* = 4.7 Hz, 1H), 7.65 – 7.61 (m, 1H), 7.33 – 7.28 (m, 2H), 7.16 – 7.13 (m, 1H), 6.92 (d, *J* = 8.3 Hz, 1H), 6.77 (d, *J* = 8.2 Hz, 1H), 6.64 (d, *J* = 8.2 Hz, 1H), 6.43 (d, *J* = 8.3 Hz, 1H), 5.36 (s, 2H), 4.57 – 4.43 (m, 2H), 3.78 (s, 3H), 2.73 – 2.68 (m, 2H), 2.33 – 2.23 (m, 2H), 1.75 – 1.66 (m, 4H). **^{13}C NMR** (101 MHz, Chloroform-*d*) δ 159.45, 158.23, 154.54, 148.62, 143.50, 137.76, 137.12, 130.29, 129.74, 126.83, 121.94, 121.32, 116.54, 109.36, 109.03, 103.35, 55.80, 48.71, 29.24, 27.14, 23.30, 23.14; **HRMS** (ESI): *m/z*: calculated for $C_{23}H_{25}N_2O_2$: $[M + H]^+$ 361.1916, found: 361.1912; $[\alpha]^{25}_D$ = + 17.8 (*c* = 1.0, $CHCl_3$).

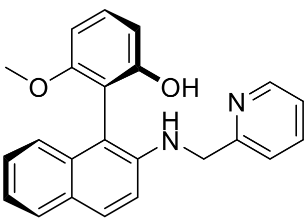

17

17: White solid, 31 mg, 87%, 24 h; **^{1}H NMR** (400 MHz, Chloroform-*d*) δ 8.45 (d, *J* = 4.5 Hz, 1H), 7.70 (d, *J* = 8.8 Hz, 2H), 7.63 – 7.59 (m, 1H), 7.41 (t, *J* = 8.2 Hz, 1H), 7.31 (d, *J* = 7.0 Hz, 2H), 7.22 (t, *J* = 6.8 Hz, 2H), 7.14 – 7.11 (m, 1H), 7.01 (d, *J* = 8.9 Hz, 1H), 6.86 (d, *J* = 8.2 Hz, 1H), 6.72 (d, *J* = 8.3 Hz, 1H), 4.74 – 4.58 (m, 2H), 3.71 (s, 3H). **^{13}C NMR** (101 MHz, Chloroform-*d*) δ 159.10, 159.00, 155.56, 148.70, 143.76, 137.13, 133.76, 130.21, 129.95, 128.09, 127.63, 126.77, 123.64, 122.21, 122.07, 121.41, 113.93, 110.93, 109.85, 109.64, 103.54, 55.92, 48.46; **HRMS** (ESI): *m/z*: calculated for $C_{23}H_{21}N_2O_2$: $[M + H]^+$ 375.1603, found: 375.1601; $[\alpha]^{25}_D$ = + 35.1 (*c* = 1.0, $CHCl_3$).

Application of biaryl amino-alcohol derivatives 15 - 17 as ligands

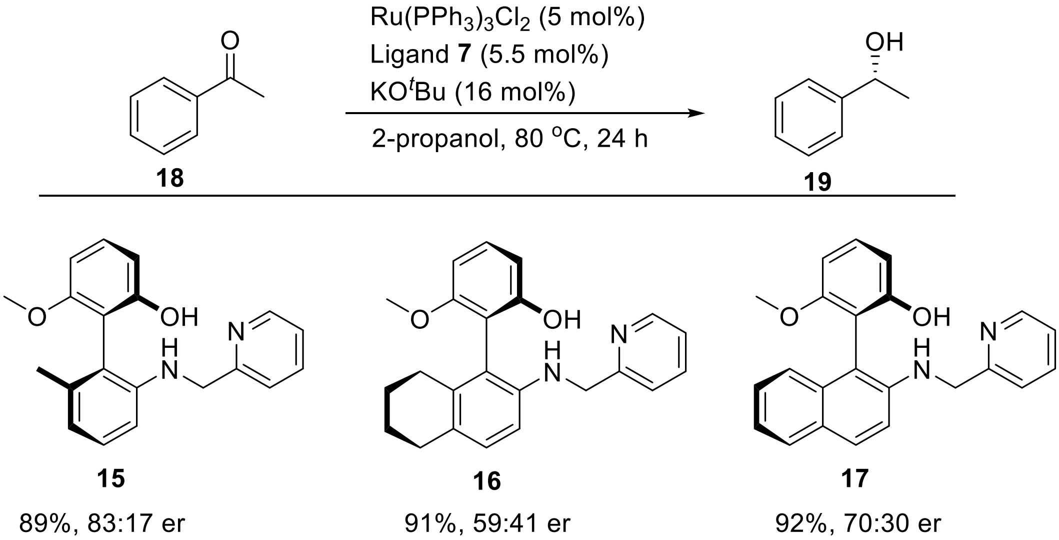

The ligand (0.055 mol), KO*ᵗ*Bu (11 mol%) and $Ru(PPh_3)_3Cl_2$ (5 mol%) was dissolved in 2-propanol (1.0 mL) under N_2 and the solution was stirred at room temperature for 1.5 h to accomplish the formation of the catalyst. Then, acetophenone (0.1 mol) and additional KO^{t}Bu (5 mol%) were added. The reaction mixture was stirred at 80 °C for

24 h. After 24 h the reaction was stopped by addition of 0.1 mol/L solution of acetic acid in 2-propanol (0.30 mL). After removal of the solvent, the crude residue was purified *via* column chromatography on silica gel to afford the desired product **19**.

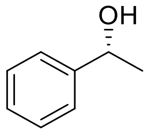

19

19: Colorless oil; **^{1}H NMR** (400 MHz, Chloroform-*d*) δ 7.41 – 7.36 (m, 4H), 7.33 – 7.29 (m, 1H), 4.90 (q, *J* = 6.4 Hz, 1H), 2.22 (s, 1H), 1.51 (d, *J* = 6.4 Hz, 3H). **^{13}C NMR** (101 MHz, Chloroform-*d*) δ 145.85, 128.50, 127.46, 125.42, 70.38, 25.17; $[\alpha]^{25}_{D}$ = + 15.2; + 9.6; + 12.9 (*c* = 1.0, $CHCl_3$).

Supplementary References:

1. Yasui, Y., Suzuki, K., Matsumoto, T. & Konegawa, T. Transmission of axial chirality to spiro center chirality, enabling enantiospecific access to erythrinan alkaloids. *Synlett*, **4**, 619-622 (2004).
2. Okuyama, K. et al. Enantiodivergent synthesis of tetra-ortho-substituted biphenyls by enzymatic desymmetrization. *Synlett*, **6**, 941-944 (2009).
3. Mori, K. et al. Enantioselective synthesis of multisubstituted biaryl skeleton by chiral phosphoric acid catalyzed desymmetrization/kinetic resolution sequence. *J. Am. Chem. Soc.* **10**, 3964-3970 (2013).
4. Milne, J. E. & Buchwald, S. L. An extremely active catalyst for the Negishi cross-coupling reaction. *J. Am. Chem. Soc*. **126**, 13028 – 13032 (2004).
5. Lai, M. J. et al. N-Sulfonyl-aminobiaryls as antitubulin agents and inhibitors of signal transducers and activators of transcription 3 (STAT3) signaling. *J. Med. Chem*. **58**, 6549 – 6558 (2015).
6. Yang, B. et al. Microwave-assisted expeditious synthesis of 5-fluoroalkyl-3-(aryl/alkyl)-oxazolidin-2-ones. *Tetrahedron*, **69**, 3331-3337 (2013).
